# Theoretical and Experimental Study of the Effect of Functional Groups on the Thiazole-5H Proton Chemical Shift in ^1^H NMR Spectroscopy

**DOI:** 10.3390/ma19112400

**Published:** 2026-06-04

**Authors:** Angelika Baranowska-Łączkowska, Krzysztof Z. Łączkowski

**Affiliations:** 1 Department of Physics, Kazimierz Wielki University, Al. Powstańców Wielkopolskich 2, PL-85090 Bydgoszcz, Poland; 2 Department of Chemical Technology and Pharmaceuticals, Faculty of Pharmacy, Nicolaus Copernicus University, 2 Jurasz St., PL-85089 Bydgoszcz, Poland; krzysztof.laczkowski@cm.umk.pl

**Keywords:** thiazole, ^1^H NMR chemical shift, DFT calculation, frontier orbitals, molecular electrostatic potential surface

## Abstract

The relationship between the position of the thiazole-5H proton signal and the presence of various substituents in the molecule was investigated in detail from an experimental and theoretical point of view. For this purpose, twenty 2,4-disubstituted thiazole derivatives were carefully chosen and synthesized, and their NMR spectra were recorded. Density functional theory calculations of ^1^H NMR chemical shifts, frontier molecular orbitals, and molecular electrostatic potential surfaces were performed. The position of the thiazole-5H proton signal in the NMR spectrum is shown to strongly depend on the type and position of substituents in the molecule. Based on the obtained results, we can conclude that compounds with the smallest values of thiazole-5H proton shift are simultaneously those with small electron affinity, ionization potential and molecular electronegativity values, while compounds with the largest values of thiazole-5H proton shift have large electron affinity, ionization potential, and molecular electronegativity and a small HOMO-LUMO energy gap. These relationships become less clear in the case of compounds with intermediate values of the proton shift. Present research is a step towards an easy-to-use tool for predicting electronic effects in materials containing thiazole, based on the position of the thiazole-5H proton NMR signal.

## 1. Introduction

**Figure 1:**
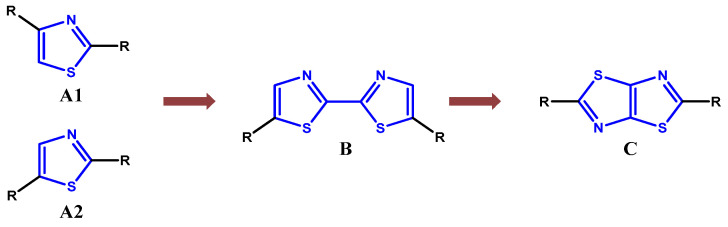
Thiazole derivatives types **A1**, **A2**, **B** and **C** reported in the literature.

In recent years, there has been a rapid increase in interest in materials with unique electronic properties, driven by the development of organic field-effect transistors [1,2], organic photovoltaics [3], and organic light-emitting diodes [4]. Heterocyclic compounds with their very strongly π-conjugated electron systems are irreplaceable structures for the abovementioned applications. Modern methods of organic synthesis, while demanding and expensive, allow for rational modification of their properties by introducing appropriate strong electron-donor and electron-acceptor groups, as well as through the formation of conjugated heterocyclic aromatic systems. The presence of these groups or conjugated units causes such materials to have high second- and third-order optical nonlinearities [5] and a significant electrooptic coefficient [6]. Some of the most frequently chosen systems include benzothiadiazole [7], carbazole [8], thiophene [9], and phenanthroline [10]. The family of optical materials also includes thiazoles with various substituents (**A1** and **A2**) [11,12,13,14,15,16], coplanar bithiazoles [17,18,19] (**B**), and a large group of compounds called thiazolo [5,4-d]thiazoles (**C**) [20,21,22] (Figure 1). Experimental and theoretical studies have shown that replacing the benzene ring with a thiazole ring with acceptor properties leads to an increase in the hyperpolarizability and the photochemical and thermal stability of the molecules [23,24].

In our earlier works, we have investigated thiazole derivatives of biological importance [25,26,27] and of possible applications as optoelectronic materials [28,29]. Presently, we focus on the influence of the type and position of substituents within the molecule on the position of the thiazole-5H proton signal in the ^1^H NMR spectrum, investigating it from an experimental and theoretical point of view. Understanding of this relationship would make thiazole-5H proton a good marker for predicting the impact of electronic effects in molecules, resulting from the presence of simple substituents, such as the -Cl, -NO_2_, -CN or -N(CH_3_)_2_, as well as more complex heterocyclic rings. Both theoretical calculations and experimental measurements of the NMR proton shifts are easily accessible and relatively inexpensive, and thus an understanding of the abovementioned relationship could lead to a widely available tool.

In this work, twenty **A1**-type 2,4-disubstituted thiazole derivatives were carefully chosen and synthesized. Both electron-donating and electron-withdrawing groups were introduced into the molecules. To confirm the structure of the synthesized compounds, their ^1^H, ^13^C NMR and HRMS spectra were recorded. Next, the density functional theory (DFT) calculations were performed in order to investigate the electronic structure of the investigated compounds. The ^1^H NMR chemical shifts, frontier molecular orbitals, global reactivity descriptors, and molecular electrostatic potential (MEP) surfaces were evaluated. The presented approach is universal and can be used for any system containing thiazole as long as proton atoms are present, e.g., for **A2**-type and **B**-type derivatives.

## 2. Materials and Methods

### 2.1. Chemistry

All experiments were carried out under an air atmosphere. Reagents were generally the best quality commercial-grade products and were used without further purification. ^1^H NMR (400 MHz) and ^13^C NMR (100 MHz) spectra were recorded on a Bruker Avance III multinuclear instrument (Bruker, Zurich, Switzerland). The chemical shift value of a multiplet (doublet, triplet, etc.) is given by reading the position of its center. This is achieved by calculating the average of the chemical shifts in the two outermost peaks (ppm) or by directly identifying the exact center peak in symmetrical multiplets. For very broad, irregular signals, their range is given. MS spectra were recorded on a triple quadrupole mass spectrometer detector LCMS-8040 (Shimadzu, Kyoto, Japan). Melting points were determined in open glass capillaries and are uncorrected. Analytical TLC was performed using Macherey-Nagel Polygram Sil G/UV_254_ 0.2 mm plates. All thioureas and appropriate bromoketones were commercial materials of 99% purity (Merck, Darmstadt, Germany).

#### 2.1.1. *N*,4-Diphenylthiazol-2-amine (**3a**)—Typical Procedure

*N*-Phenylthiourea **1a** (0.152 g, 1.0 mmol) was added to a stirred solution of 2-bromoacetophenone (0.199 g, 1.0 mmol) in absolute ethanol (20 mL). The reaction mixture was stirred under reflux for 20 h, cooled to room temperature and reaction mixture was added to water (50 mL). The separate precipitate was collected by filtration and washed with ethanol, to afford the desired white product to yield 0.25 g (99%); mp 130–132 °C, lit. [30] 136–137 °C; eluent: dichloromethane/methanol (95:5), R_f_ = 0.69. ^1^H NMR (400 MHz, DMSO-d_6_), δ (ppm): 6.950 (t, 1H, CH, J = 7.2 Hz); 7.268–7.368 (m, 3H, 3CH); 7.322 (s, 1H, CH); 7.420 (t, 2H, 2CH, J = 8.8 Hz); 7.715 (d, 2H, 2CH, J = 8.0 Hz); 7.910 (d, 2H, 2CH, J = 7.6 Hz); 10.246 (bs, 1H, NH). ^13^C NMR (100 MHz, DMSO-d_6_), δ (ppm): 103.367; 117.305 (2C); 121.677; 126.146 (2C); 128.047; 129.115 (2C); 129.487 (2C); 135.031; 141.711; 150.590; 163.572. LC-ESI-HRMS (*m*/*z*) calculated for C_15_H_13_N_2_S: 253.0799 [M + H]^+^. Found: 253.0798 [M + H]^+^.

#### 2.1.2. 4-(4-Fluorophenyl)-*N*-phenylthiazol-2-amine (**3b**)

White, yield 0.24 g (89%); (dichloromethane/methanol, 95:5, R_f_ = 0.88); mp 127–129 °C, lit. [30] 126–127 °C. ^1^H NMR (400 MHz, DMSO-d_6_), δ (ppm): 6.952 (t, 1H, CH, J = 7.2 Hz); 7.250 (t, 2H, 2CH, J = 8.8 Hz); 7.300 (s, 1H, CH); 7.332 (t, 2H, 2CH, J = 7.6 Hz); 7.706 (d, 2H, 2CH, J = 8.8 Hz); 7.945 (q, 2H, 2CH, J = 8.8 Hz); 10.256 (bs, 1H, NH). ^13^C NMR (100 MHz, DMSO-d_6_), δ (ppm): 103.080; 115.941 (d, 2C, J_C-F_ = 21.4 Hz); 117.324 (2C); 121.719; 128.117 (d, 2C, J_C-F_ = 7.9 Hz); 129.485 (2C); 131.634; 141.665; 149.547; 162.118 (d, J_C-F_ = 241.8 Hz); 163.708. LC-ESI-HRMS (*m*/*z*) calculated for C_15_H_12_FN_2_S: 271.0705 [M + H]^+^. Found: 271.0710 [M + H]^+^.

#### 2.1.3. 4-(4-Chlorophenyl)-*N*-phenylthiazol-2-amine (**3c**)

White, yield 0.22 g (77%); (dichloromethane/methanol, 95:5, R_f_ = 0.91); mp 141–143 °C, lit. [30] 141–142 °C. ^1^H NMR (400 MHz, DMSO-d_6_), δ (ppm): 6.980 (t, 1H, CH, J = 7.0 Hz); 7.357 (t, 2H, 2CH, J = 7.7 Hz); 7.424 (d, 1H, CH, J = 1.4 Hz); 7.503 (d, 2H, 2CH, J = 8.4 Hz); 7.746 (d, 2H, 2CH, J = 7.7 Hz); 7.951 (d, 2H, 2CH, J = 8.4 Hz); 10.310 (bs, 1H, NH). ^13^C NMR (100 MHz, DMSO-d_6_), δ (ppm): 104.212; 117.352 (2C); 121.781; 127. 837 (2C); 129.137 (2C); 129.502 (2C); 132.455; 133.867; 141.609; 149.335; 163.737. LC-ESI-HRMS (*m*/*z*) calculated for C_15_H_12_ClN_2_S: 287.0410 [M + H]^+^. Found: 287.0406 [M + H]^+^.

#### 2.1.4. 4-(2-Chlorophenyl)-*N*-phenylthiazol-2-amine (**3d**)

White, yield 0.21 g (91%); (dichloromethane/methanol, 95:5, R_f_ = 0.90); mp 118–120 °C, lit. [30] 130–131 °C. ^1^H NMR (400 MHz, DMSO-d_6_), δ (ppm): 6.937 (tt, 1H, CH, J_1_ = 0.8 Hz, J_2_ = 7.6 Hz); 7.272–7.338 (m, 2H, 2CH); 7.331 (s, 1H, CH); 7.344–7.378 (m, 1H, CH); 7.431 (td, 1H, CH, J_1_ = 1.2 Hz, J_2_ = 7.6 Hz); 7.529 (dd, 1H, CH, J = 1.6 Hz, J = 8.0 Hz); 7.648–7.696 (m, 2H, 2CH); 7.915 (dd, 1H, CH, J = 1.6 Hz, J = 7.6 Hz); 10.258 (bs, 1H, NH). ^13^C NMR (100 MHz, DMSO-d_6_), δ (ppm): 108.320; 117.308 (2C); 121.708; 127.816; 129.460; 129.516 (2C); 130.893; 131.262; 131.676; 133.664; 141.634; 147.195; 162.682.

#### 2.1.5. 4-(4-Bromophenyl)-*N*-phenylthiazol-2-amine (**3e**)

White, yield 0.29 g (88%); (dichloromethane/methanol, 95:5, R_f_ = 0.87); mp 144–146 °C, lit. [30] 137–138 °C. ^1^H NMR (400 MHz, DMSO-d_6_), δ (ppm): 6.980 (t, 1H, CH, J = 7.7 Hz); 7.357 (t, 2H, 2CH, J = 7.0 Hz); 7.436 (d, 1H, CH, J = 2.1 Hz); 7.637 (d, 2H, 2CH, J = 9.1 Hz); 7.721 (d, 2H, 2CH, J = 8.4 Hz); 7.886 (d, 2H, 2CH, J = 8.4 Hz); 10.308 (bs, 1H, NH).^13^C NMR (100 MHz, DMSO-d_6_), δ (ppm): 104.298; 117.365 (2C); 121.056; 121.791; 128.148 (2C); 129.502 (2C); 132.044 (2C); 134.213; 141.608; 149.397; 163.761. LC-ESI-HRMS (*m*/*z*) calculated for C_15_H_12_BrN_2_S: 330.9905 [M + H]^+^. Found: 330.9908 [M + H]^+^.

#### 2.1.6. 4-(3-Bromophenyl)-*N*-phenylthiazol-2-amine (**3f**)

Light yellow, yield 0.30 g (91%); (dichloromethane/methanol, 95:5, R_f_ = 0.88); mp 86–87 °C, lit. [31] oil. ^1^H NMR (400 MHz, DMSO-d_6_), δ (ppm): 6.986 (t, 1H, CH, J = 7.7 Hz); 7.366 (t, 2H, 2CH, J = 7.7 Hz); 7.414 (t, 1H, CH, J = 8.4 Hz); 7.511 (s, 1H, CH); 7.517 (d, 1H, CH, J = 1.4 Hz); 7.706 (d, 2H, 2CH, J = 8.4 Hz); 7.948 (d, 1H, CH, J = 7.7 Hz); 8.102 (s, 1H, CH); 10.326 (bs, 1H, NH). ^13^C NMR (100 MHz, DMSO-d_6_), δ (ppm): 105.028; 117.393 (2C); 121.861; 126.604; 125.139; 128.578; 129.529 (2C); 130.682; 131.325; 137.287; 141.561; 148.871; 163.782. LC-ESI-HRMS (*m*/*z*) calculated for C_15_H_12_BrN_2_S: 330.9905 [M + H]^+^. Found: 330.9911 [M + H]^+^.

#### 2.1.7. *N*-Phenyl-4-*p*-tolylthiazol-2-amine (**3g**)

Sandy, yield 0.21 g (78%); (dichloromethane/methanol, 95:5, R_f_ = 0.95); mp 91–93 °C, lit. [30] 94–95 °C. ^1^H NMR (400 MHz, DMSO-d_6_), δ (ppm): 2.314 (s, 3H, CH_3_); 6.946 (t, 1H, CH, J = 7.2 Hz); 7.222 (d, 2H, 2CH, J = 8.0 Hz); 7.232 (s, 1H, CH); 7.332 (t, 2H, 2CH, J = 7.6 Hz); 7.708 (d, 2H, 2CH, J = 8.8 Hz); 7.796 (d, 2H, 2CH, J = 8.4 Hz); 10.222 (bs, 1H, NH). ^13^C NMR (100 MHz, DMSO-d_6_), δ (ppm): 21.311; 102.416; 117.277 (2C); 121.626; 126.087 (2C); 129.472 (2C); 129.669 (2C); 132.410; 137.314; 141.756; 150.673; 163.494. LC-ESI-HRMS (*m*/*z*) calculated for C_16_H_15_N_2_S: 267.0956 [M + H]^+^. Found: 267.0956 [M + H]^+^.

#### 2.1.8. 4-(4-Methoxyphenyl)-*N*-phenylthiazol-2-amine (**3h**)

Sandy, yield 0.28 g (99%); (dichloromethane/methanol, 95:5, R_f_ = 0.71); mp 142–143 °C, lit. [30] 142–143 °C. ^1^H NMR (400 MHz, DMSO-d_6_), δ (ppm): 3.776 (s, 3H, CH_3_); 6.943 (t, 1H, CH, J = 7.2 Hz); 6.979 (d, 2H, 2CH, J = 8.8 Hz); 7.140 (s, 1H, CH); 7.326 (t, 2H, 2CH, J = 7.6 Hz); 7.703 (d, 2H, 2CH, J = 8.4 Hz); 7.834 (d, 2H, 2CH, J = 8.8 Hz); 10.215 (bs, 1H, NH). ^13^C NMR (100 MHz, DMSO-d_6_), δ (ppm): 55.617; 101.237; 114.488 (2C); 117.369 (2C); 121.710; 127.495 (2C); 127.787; 129.470 (2C); 141.694; 150.151; 159.328; 163.566. LC-ESI-HRMS (*m*/*z*) calculated for C_16_H_15_N_2_OS: 283.0905 [M + H]^+^. Found: 283.0905 [M + H]^+^.

#### 2.1.9. *N*-Phenyl-4-(4-(trifluoromethyl)phenyl)thiazol-2-amine (**3i**)

White, yield 0.26 g (81%); (dichloromethane/methanol, 95:5, R_f_ = 0.88); mp 148–149 °C. ^1^H NMR (400 MHz, DMSO-d_6_), δ (ppm): 6.969 (t, 1H, CH, J = 7.6 Hz); 7.342 (t, 2H, 2CH, J = 7.6 Hz); 7.567 (s, 1H, CH); 7.714 (d, 2H, 2CH, J = 8.8 Hz); 7.777 (d, 2H, 2CH, J = 8.4 Hz); 8.117 (d, 2H, 2CH, J = 8.0 Hz); 10.329 (bs, 1H, NH). ^13^C NMR (100 MHz, DMSO-d_6_), δ (ppm): 106.202; 117.428 (2C); 121.875; 123.486; 126.100 (2C); 126.666 (2C); 128.092 (q, J_C-F_ = 6.3 Hz); 129.502 (2C); 138.675; 141.545; 149.050; 163.909. LC-ESI-HRMS (*m*/*z*) calculated for C_16_H_12_F_3_N_2_S: 321.0673 [M + H]^+^. Found: 321.0674 [M + H]^+^.

#### 2.1.10. *N*-Phenyl-4-(4-(trifluoromethoxy)phenyl)thiazol-2-amine (**3j**)

White, yield 0.28 g (99%); (dichloromethane/methanol, 95:5, R_f_ = 0.90); mp 129–130 °C. ^1^H NMR (400 MHz, DMSO-d_6_), δ (ppm): 6.959 (t, 1H, CH, J = 7.2 Hz); 7.333 (t, 2H, 2CH, J = 7.6 Hz); 7.405 (s, 1H, CH); 7.414 (d, 2H, 2CH, J = 7.2 Hz); 7.685–7.729 (m, 2H, 2CH); 8.020 (d, 2H, 2CH, J = 8.8 Hz); 10.289 (bs, 1H, NH). ^13^C NMR (100 MHz, DMSO-d_6_), δ (ppm): 104.425; 117.363 (2C); 121.706 (2C); 121.779; 121.873; 127.896 (2C); 129.476 (2C); 134.295; 141.605; 148.045; 149.140; 163.840. LC-ESI-HRMS (*m*/*z*) calculated for C_16_H_12_F_3_N_2_OS: 337.0622 [M + H]^+^. Found: 337.0624 [M + H]^+^.

#### 2.1.11. 4-(4-Nitrophenyl)-*N*-phenylthiazol-2-amine (**3k**)

Orange, yield 0.29 g (98%); (dichloromethane/methanol, 95:5, R_f_ = 0.73); mp 204–205 °C, lit. [32] 206–207 °C. ^1^H NMR (400 MHz, DMSO-d_6_), δ (ppm): 7.006 (t, 1H, CH, J = 7.7 Hz); 7.374 (t, 2H, 2CH, J = 8.4 Hz); 7.742 (d, 2H, 2CH, J = 9.1 Hz); 7.748 (s, 1H, CH); 8.192 (d, 2H, 2CH, J = 9.1 Hz); 8.311 (d, 2H, 2CH, J = 8.4 Hz); 10.405 (bs, 1H, NH).^13^C NMR (100 MHz, DMSO-d_6_), δ (ppm): 108.327; 117.504 (2C); 122.000; 124.578 (2C); 126.939 (2C); 129.529 (2C); 140.931; 141.437; 146.711; 148.550; 164.016. LC-ESI-HRMS (*m*/*z*) calculated for C_15_H_12_N_3_O_2_S: 298.0650 [M + H]^+^. Found: 298.0649 [M + H]^+^.

#### 2.1.12. 4-(2-(Phenylamino)thiazol-4-yl)benzonitrile (**3l**)

White, yield 0.26 g (94%); (dichloromethane/methanol, 95:5, R_f_ = 0.54); mp 201–203 °C, lit. [33] 210–212 °C. ^1^H NMR (400 MHz, DMSO-d_6_), δ (ppm): 6.969 (t, 1H, CH, J = 7.2 Hz); 7.343 (t, 2H, 2CH, J = 8.0 Hz); 7.642 (s, 1H, CH); 7.709 (d, 2H, 2CH, J = 8.4 Hz); 7.882 (d, 2H, 2CH, J = 8.8 Hz); 8.092 (d, 2H, 2CH, J = 8.4 Hz); 10.341 (bs, 1H, NH). ^13^C NMR (100 MHz, DMSO-d_6_), δ (ppm): 107.314; 110.096; 117.445 (2C); 119.497; 121.932; 126.728 (2C); 129.518 (2C); 133.180 (2C); 139.045; 141.465; 148.850; 163.905. LC-ESI-HRMS (*m*/*z*) calculated for C_16_H_12_N_3_S: 278.0752 [M + H]^+^. Found: 278.0748 [M + H]^+^.

#### 2.1.13. 4-(4-(Dimethylamino)phenyl)-*N*-phenylthiazol-2-amine (**3m**)

Light brown, yield 0.21 g (71%); (dichloromethane/methanol, 95:5, R_f_ = 0.70); mp 168–169 °C. ^1^H NMR (400 MHz, DMSO-d_6_), δ (ppm): 2.922 (s, 6H, 2CH_3_); 6.750 (d, 2H, 2CH, J = 9.2 Hz); 6.934 (t, 1H, CH, J = 7.2 Hz); 6.972 (s, 1H, CH); 7.319 (t, 2H, 2CH, J = 8.0 Hz); 7.683–7.750 (m, 4H, 4CH); 10.156 (bs, 1H, NH).^13^C NMR (100 MHz, DMSO-d_6_), δ (ppm): 40.494 (2C); 99.095; 112.589 (2C); 117.185 (2C); 121.473; 123.512; 127.081 (2C); 129.438 (2C); 141.876; 150.323; 151.211; 163.197. LC-ESI-HRMS (*m*/*z*) calculated for C_17_H_18_N_3_S: 296.1221 [M + H]^+^. Found: 296.1222 [M + H]^+^.

#### 2.1.14. *N*-(4-Fluorophenyl)-4-(4-methoxyphenyl)thiazol-2-amine (**3n**)

Light yellow, yield 0.27 g (90%); (dichloromethane/methanol, 95:5, R_f_ = 0.61); mp 139–140 °C, lit. [34] 100 °C. ^1^H NMR (400 MHz, DMSO-d_6_), δ (ppm): 3.775 (s, 3H, CH_3_); 6.971 (d, 2H, 2CH, J = 8.8 Hz); 7.129 (s, 1H, CH); 7.170 (t, 2H, 2CH, J = 8.8 Hz); 7.734 (q, 2H, 2CH, J = 9.6 Hz); 7.828 (d, 2H, 2CH, J = 8.8 Hz); 10.231 (bs, 1H, NH). ^13^C NMR (100 MHz, DMSO-d_6_), δ (ppm): 55.571; 101.129; 114.456 (2C); 115.557 (d, 2C, J = 21.9 Hz); 118.757 (2C, J = 7.6 Hz); 127.479 (2C); 127.898; 138.307; 150.397; 157.303 (d, J = 236.0 Hz); 159.327; 163.570. LC-ESI-HRMS (*m*/*z*) calculated for C_16_H_14_FN_2_OS: 301.0811 [M + H]^+^. Found: 301.0814 [M + H]^+^.

#### 2.1.15. 4-(4-Fluorophenyl)-*N*-(4-methoxyphenyl)thiazol-2-amine (**3o**)

Off-white, yield 0.26 g (87%); (dichloromethane/methanol, 95:5, R_f_ = 0.64); mp 185–186 °C. ^1^H NMR (400 MHz, DMSO-d_6_), δ (ppm): 3.720 (s, 3H, CH_3_); 6.927 (d, 2H, 2CH, J = 8.8 Hz); 7.222 (s, 1H, CH); 7.235 (t, 2H, 2CH, J = 8.8 Hz); 7.607 (d, 2H, 2CH, J = 9.2 Hz); 7.923 (q, 2H, 2CH, J = 9.2 Hz); 10.039 (bs, 1H, NH). ^13^C NMR (100 MHz, DMSO-d_6_), δ (ppm): 55.663; 102.351; 114.734 (2C); 115.884 (d, 2C, J = 21.5 Hz); 119.163 (2C); 128.070 (d, 2C, J = 7.8 Hz); 131.730; 135.185; 149.479; 154.624; 162.071 (d, J_C-F_ = 245.3 Hz); 164.368. LC-ESI-HRMS (*m*/*z*) calculated for C_16_H_14_FN_2_OS: 301.0811 [M + H]^+^. Found: 301.0812 [M + H]^+^.

#### 2.1.16. *N*,4-bis(4-Fluorophenyl)thiazol-2-amine (**3p**)

White, yield 0.25 g (87%); (dichloromethane/methanol, 95:5, R_f_ = 0.69); mp 155–157 °C, lit. [35] 127 °C. ^1^H NMR (400 MHz, DMSO-d_6_), δ (ppm): 7.172 (t, 2H, 2CH, J = 8.8 Hz); 7.243 (t, 2H, 2CH, J = 8.8 Hz); 7.296 (s, 1H, CH); 7.728 (q, 2H, 2CH, J = 9.2 Hz); 7.937 (q, 2H, 2CH, J = 8.8 Hz); 10.276 (bs, 1H, NH). ^13^C NMR (100 MHz, DMSO-d_6_), δ (ppm): 103.097; 115.845 (d, 2C, J = 4.7 Hz); 116.064 (d, 2C, J = 5.8 Hz); 118.846 (d, 2C, J = 7.7 Hz); 128.116 (d, 2C, J = 7.7 Hz); 131.599; 138.166; 149.476; 157.348 (d, J = 235.3 Hz); 162.122 (d, J = 242.1 Hz); 163.785. LC-ESI-HRMS (*m*/*z*) calculated for C_15_H_11_F_2_N_2_S: 289.0611 [M + H]^+^. Found: 289.0612 [M + H]^+^.

#### 2.1.17. *N*,4-bis(4-Methoxyphenyl)thiazol-2-amine (**3q**)

White, yield 0.24 g (89%); (dichloromethane/methanol, 95:5, R_f_ = 0.55); mp 182–184 °C, lit. [36] 178–180 °C. ^1^H NMR (400 MHz, DMSO-d_6_), δ (ppm): 3.720 (s, 3H, CH_3_); 3.773 (s, 3H, CH_3_); 6.923 (d, 2H, 2CH, J = 9.2 Hz); 6.965 (d, 2H, 2CH, J = 8.8 Hz); 7.058 (s, 1H, CH); 7.608 (d, 2H, 2CH, J = 9.2 Hz); 7.815 (d, 2H, 2CH, J = 8.8 Hz); 9.988 (bs, 1H, NH). ^13^C NMR (100 MHz, DMSO-d_6_), δ (ppm): 55.584; 55.678; 100.427; 114.428 (2C); 114. 732 (2C); 119.085 (2C); 127.447 (2C); 128.034; 135.309; 150.380; 154.547; 159.265; 164.153. LC-ESI-HRMS (*m*/*z*) calculated for C_17_H_17_N_2_O_2_S: 313.1011 [M + H]^+^. Found: 313.1011 [M + H]^+^.

#### 2.1.18. 4-(4-Methoxyphenyl)-*N*-(4-nitrophenyl)thiazol-2-amine (**3r**)

Dark orange, yield 0.31 g (95%); (dichloromethane/methanol, 95:5, R_f_ = 0.64); mp 197–199 °C, lit. [37] 184–186 °C. ^1^H NMR (400 MHz, DMSO-d_6_), δ (ppm): 3.788 (s, 3H, CH_3_); 6.991 (d, 2H, 2CH, J = 9.2 Hz); 7.357 (s, 1H, CH); 7.884 (d, 2H, 2CH, J = 9.2 Hz); 7.922 (d, 2H, 2CH, J = 9.2 Hz); 8.246 (d, 2H, 2CH, J = 9.2 Hz); 11.028 (bs, 1H, NH). ^13^C NMR (100 MHz, DMSO-d_6_), δ (ppm): 55.602; 103.520; 114.514 (2C); 116.601 (2C); 126.007 (2C); 127.497; 127.647 (2C); 140.623; 147.409; 150.760; 159.511; 162.044. LC-ESI-HRMS (*m*/*z*) calculated for C_16_H_14_N_3_O_3_S: 328.0756 [M + H]^+^. Found: 328.0758 [M + H]^+^.

#### 2.1.19. *N*-(4-Methoxyphenyl)-4-(4-nitrophenyl)thiazol-2-amine (**3s**)

Yellow, yield 0.32 g (97%); (dichloromethane/methanol, 95:5, R_f_ = 0.55); mp 171–173 °C, lit. [36] 166–168 °C. ^1^H NMR (400 MHz, DMSO-d_6_), δ (ppm): 3.729 (s, 3H, CH_3_); 6.939 (d, 2H, 2CH, J = 9.2 Hz); 7.614 (d, 2H, 2CH, J = 9.2 Hz); 7.645 (s, 1H, CH); 8.141 (d, 2H, 2CH, J = 8.8 Hz); 8.270 (d, 2H, 2CH, J = 8.8 Hz); 10.163 (bs, 1H, NH). ^13^C NMR (100 MHz, DMSO-d_6_), δ (ppm): 55.679; 107.672; 114.767 (2C); 119.390 (2C); 124.551 (2C); 126.897 (2C); 134.909; 141.021; 146.658; 148.509; 154.809; 164.684. LC-ESI-HRMS (*m*/*z*) calculated for C_16_H_14_N_3_O_3_S: 328.0756 [M + H]^+^. Found: 328.0760 [M + H]^+^.

#### 2.1.20. *N*,4-bis(4-Nitrophenyl)thiazol-2-amine (**3t**)

Orange, yield 0.34 g (99%); (dichloromethane/methanol, 95:5, R_f_ = 0.72); mp > 290 °C, lit. [37] 306–308 °C. ^1^H NMR (400 MHz, DMSO-d_6_), δ (ppm): 7.918 (s, 1H, CH); 7.942 (d, 2H, 2CH, J = 9.6 Hz); 8.223 (d, 2H, 2CH, J = 9.2 Hz); 8.257 (d, 2H, 2CH, J = 9.2 Hz); 8.288 (d, 2H, 2CH, J = 9.2 Hz); 11.166 (bs, 1H, NH). ^13^C NMR (100 MHz, DMSO-d_6_), δ (ppm): 110.539; 116.913 (2C); 124.597 (2C); 126.023 (2C); 127.161 (2C); 140.498; 140.924; 146.928; 147.075; 148.717; 162.715.

### 2.2. Computational Details

As theoretical results are compared to the experimental ^1^H NMR spectra recorded in DMSO-d_6_, accounting for the solvent effects was crucial. This was done through employing the Polarizable Continuum Model (PCM) [38,39] at all calculation stages reported in this work.

**Figure 2:**
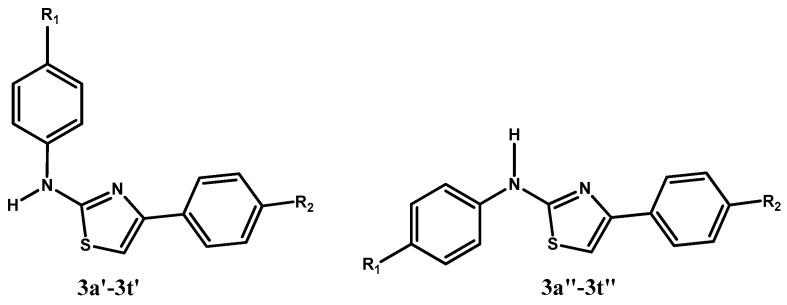
The two conformers of compounds **3a**–**3t** employed in geometry optimization calculations, obtained by the rotation around the C–N bond formed between the nitrogen atom of the amine group and the C2 carbon atom of the thiazole ring.

For each of the investigated systems **3a** through **3t**, two starting point geometries were used, obtained by the rotation around the C–N bond formed between the nitrogen atom of the amine group and the C2 carbon atom of the thiazole ring by 180 degrees, as shown in Figure 2. Geometrical parameters of the starting structures were optimized using the B3LYP [40,41] exchange-correlation functional and the 6-311++G** basis set [42,43]. Next, vibrational frequencies were calculated for the optimized structures, confirming that all geometries correspond to real minima on the potential energy surfaces.

For all identified stable structures, the energy of the highest occupied molecular orbital (HOMO), denoted as $E_{HOMO}$, and of the lowest unoccupied molecular orbital (LUMO), denoted as $E_{LUMO}$, were evaluated. The HOMO–LUMO energy gap is calculated as

$$∆E=E_{LUMO}-E_{HOMO},$$

and informs on the molecule’s electronic excitation energy. The ionization potential IP, reflecting the molecule’s tendency to donate electrons, and electron affinity EA, reflecting the tendency to accept electrons, are the negative of the HOMO and of the LUMO energy, respectively. The electronegativity χ is defined as their arithmetic mean:

$$\chi =\frac{IP+EA}{2},$$

and chemical hardness η and softness S as:

$$\eta =\frac{IP-EA}{2}=\frac{1}{S}$$

Small values of S (large values of η) indicate large resistance to changing the number of electrons, and low chemical reactivity. Thus, analysis of the above given properties allow us to evaluate the chemical stability of the investigated compounds. Additional information on molecular reactivity can be provided through analysis of the MEP surfaces, which give a prediction of regions susceptible to nucleo- and electrophilic attacks.

Evaluation of the proton chemical shift δ needs calculations of the chemical shielding constants of the investigated compound $\sigma_{sample}$ and of the reference $\sigma_{ref}$, that is, tetramethylsilane (TMS), using the equation:

(1) $$\delta =\sigma_{ref}-\sigma_{sample}.$$

The shielding constant is defined as one third of the trace of the shielding tensor, evaluated as a mixed second derivative of electronic energy with respect to the components of the external magnetic field and the nuclear magnetic moment [44]. To avoid the dependence of results on the choice of the Cartesian coordinate system origin, known in the literature as the gauge-origin problem, the gauge-including atomic orbitals (GIAO) have to be used in the calculations [45,46].

To properly select the level of approximation for the ^1^H NMR shielding calculations, we used a small test system resembling the investigated systems, namely the 4-phenylthiazole molecule. Its geometrical parameters were optimized at the B3LYP/6-311++G** level of approximation, and the vibrational frequency calculation was performed to confirm that the optimized structure corresponds to the real minimum on the potential energy surface. Based on our earlier ^1^H NMR investigation carried out for the 4-formylbenzoic acid-based thiazoles [47], we used the BLYP [40,41], B3LYP [40,41], M06 [48] and M06-2X [48] exchange-correlation functionals, and eleven basis sets of different size, namely the 6-31G**, 6-31++G**, 6-311G**, 6-311++G** basis sets of Pople [42,43,49,50], aug-cc-pVXZ (X = D, T and Q) basis sets of Dunning and co-workers [51,52], and the aug-pcS-*n* [53] and aug-pcSseg-*n* (*n* = 1, 2) [54] basis sets of Jensen. The aug-pcS-*n* and aug-pcSseg-*n* basis sets were downloaded from the Basis Set Exchange library [55]. The same levels of approximation were used for the TMS molecule, which was the standard (reference molecule) during all experimental measurements of proton shifts. Based on the basis set scan, the M06 functional and the aug-pcS-1 basis set were chosen for the calculation of ^1^H NMR chemical shifts in the investigated systems **3a**–**3t**. All calculations were carried out using the Gaussian 16 package [56]. Default Gaussian 16 PCM parameters were used for DMSO.

## 3. Results and Discussion

### 3.1. Chemistry

**Figure 3:**
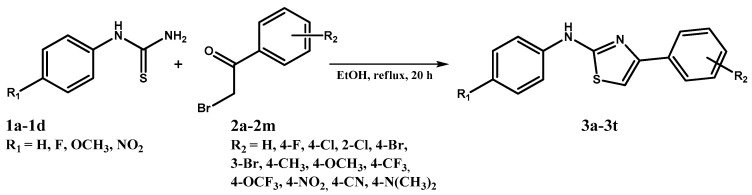
Chemical synthesis route of 2,4-disubstituted thiazole derivatives **3a**–**3t**.

The target 2,4-disubstituted thiazoles **3a**–**3t** were conveniently synthesized by the Hantzsch reaction involving the condensation of the resulting phenylthioureas **1a**–**1d** with substituted bromoacetophenones **2a**–**2m** in absolute ethanol and under reflux for 20 h (Figure 3). The twenty products were obtained with a high 77–99% yield, and with high purity (Table 1). The structures of all compounds were characterized on the basis of spectroscopic methods, ^1^H NMR (400 MHz), ^13^C NMR (100 MHz) and ESI-HRMS analyses.

### 3.2. Geometry Optimization

For each of the investigated **3a**–**3t** compounds, two stable conformers were found. Their optimized Cartesian coordinates are reported in Supplementary Materials (see Table S1). In all cases, the lower energy structure was the bent one, denoted as structure **3a′** through **3t′**. Using Boltzmann statistics, one can evaluate the population fractions $x_{i}$ for each of the compounds as:

$$x_{i}=\frac{\exp(-\Delta_{i}E/kT)}{\exp(-\Delta_{i}E/kT)+\exp(-\Delta_{j}E/kT)},$$

with $-{∆}_{i}E$ being the energy difference between the given conformer and the lower energy one, k denoting the Boltzmann constant, and T—the temperature. The values of the population fractions $x_{i}$ for the twenty studied compounds are reported in the Supplementary Materials (see Table S2). It can be seen that, depending on the system, the lower energy conformer accounts for approximately 75–95% of the population, with the most often occurring value being around 85%. The lower energy conformer has the largest values of population fraction for compounds **3t** and **3r**, while the lowest values are found for compounds **3s**, **3o** and **3q**. Although the **3a′**–**3t′** structures dominate the population, the contribution from the **3a″**–**3t″** conformers should not be neglected because molecular property values strongly depend on the system’s structure. Thus, the NMR proton shift values reported for compounds **3a**–**3t** in the following are evaluated as a weighted average of contributions from both stable structures.

### 3.3. NMR Chemical Shift Parameters

**Figure 4:**
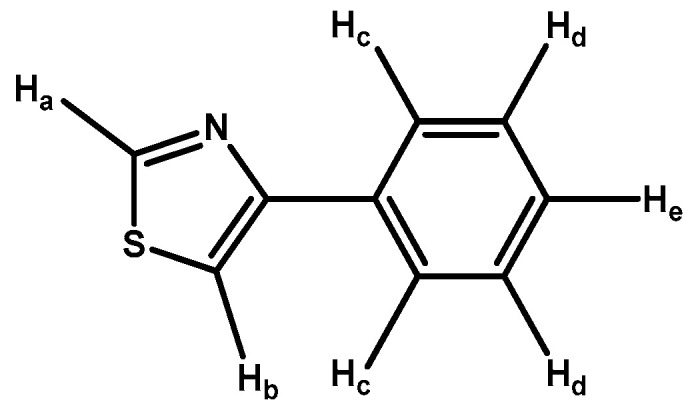
4-phenylthiazole atom numbering.

The 4-phenylthiazole atom numbering is presented in Figure 4. The calculated results are shown in Table 2, together with the values measured in DMSO-d6, and the root mean square errors (rmse) calculated with respect to experimental values. Analyzing the results obtained in the Dunning basis sets, one can see that, regardless of the exchange-correlation functional, the aug-cc-pVTZ results are close to converged. Increasing the basis set size from the aug-cc-pVDZ (320 basis functions for 4-phenylthaizole) to aug-cc-pVTZ (671 basis functions, respectively) has a positive impact on the agreement of results with the experimental values, leading to a decrease in rmse values. A further increase in the basis set size when going to aug-cc-pVQZ (1206 functions for 4-phenylthaizole) has very little or no effect on the results, proving that the use of the aug-cc-pVQZ basis set in regular calculation of proton chemical shifts would mean unnecessary additional computational cost. Considering the size of the investigated molecules, the aug-cc-pVTZ basis set can already be demanding due to the relatively large number of functions. A reduction in this cost can be done through employing Pople or Jensen basis sets. From Table 2, we can see that the exceptionally small Pople basis sets (from 204 basis functions for 4-phenylthiazole in the case of 6-31G** set to 299 functions in the case of 6-311++G** set) can yield better agreement with the experimental values than the Dunning basis sets, in particular in the case of Minnesota functionals. This observation is in agreement with our earlier work, where in vacuum calculations of proton chemical shifts were carried out for small test systems: methane, benzene and thiazole using different levels of approximation [47], and supports literature findings that the aug-pcS-*n* basis sets outperform the all-purpose basis sets of similar size in NMR shielding calculations, yielding accurate results at a relatively low computing cost. Analyzing the performance of employed exchange-correlation functionals, we notice that M06-2X gives much larger rmse values than the remaining three functionals. The BLYP and B3LYP outperform M06 for the three tested Dunning basis sets; however, in the case of the Pople and Jensen sets, all three functionals yield results of similar accuracy. Among all investigated approximations, the smallest rmse value was obtained for the M06/aug-pcS-1 combination. Simultaneously, this level of approximation yielded the best agreement between the theoretical and experimental chemical shifts in the H_b_ proton, which is the one whose ^1^H NMR signal position is investigated in the remaining compounds. It is worth noting that the M06/aug-pcS-1 approximation was also the one yielding the smallest rmse values in reference [47]. Taking the above observations into account, the M06/aug-pcS-1 approximation was chosen for the regular calculation of proton shieldings in compounds **3a**–**3t**.

**Figure 5:**
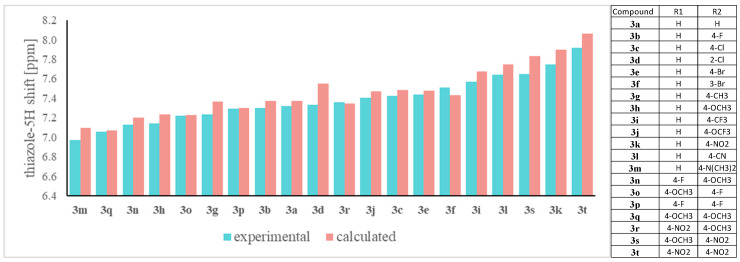
The M06/aug-pcS-1 and the experimental chemical shifts in the thiazole-5H proton in compounds **3a**–**3t**.

Based on the population fraction values (see Table S2 in Supplementary Materials), both optimized structures of compounds **3a**–**3t** have a significant percentage of the total population. Thus, the ^1^H NMR chemical shifts for the thiazole-5H proton were evaluated for both structures, and the total chemical shift was calculated as a weighted average of contributions from both stable conformers. Theoretical results are presented in Figure 5 and compared to the experimental results. Additionally, their values are also reported in the Supplementary Materials (see Table S2).

Compound **3a** does not contain substituents in the phenyl rings, and it is our reference molecule in the following, with the experimental thiazole-5H signal at 7.32 ppm. We start the discussion with the ten compounds containing a single substituent in position 4 of the phenyl ring attached to the thiazole molecule, that is, compounds **3b**, **3c**, **3e**, and **3g**–**3m**. Halogen substituents have weak electron-withdrawing character, and thus are anticipated to cause some deshielding of the thiazole-5H proton and a shift in the experimental signal to higher ppm values. This is true for chlorine and bromine substituents, where we observe a shift in the signal to 7.42 and 7.44 ppm, respectively. Nevertheless, an opposite shift is observed for compound **3b**, for which the experimental thiazole-5H signal is at 7.30 ppm, 0.02 ppm to the left of the corresponding signal in the unsubstituted **3a** compound. It is also reflected in the theoretical values, with the thiazole-5H signal positioned at 7.38 ppm for both **3a** and **3b**. This observation can be explained by the resonance effect, leading to electron density donation from the fluorine atom to the phenyl ring, opposed to the fluorine inductive effect.

Regarding compounds **3g**, **3h** and **3m**, all containing electron-donating groups, we observe a change in the thiazole-5H signal position towards low ppm (high field), with the smallest effect in the case of **3g** (change of 0.09 ppm) molecule, followed by **3h** (0.18 ppm), and the largest change observed for **3m** (0.35 ppm). This is in line with the known electron-donating character sequence of the three substituents involved, namely methyl, methoxy and dimethylamino groups. The value of proton shift measured for **3m** (6.97 ppm) is the lowest among all twenty investigated compounds. Considering theoretical values for these three compounds, we can see good qualitative agreement with the experiment (change of 0.01, 0.14 and 0.28 ppm, respectively).

Analysis of compounds containing single electron withdrawing groups, that is, compounds **3i**–**3l**, shows that in all cases there is a shift in the thiazole-5H signal toward low field (high ppm). The smallest change is observed in the case of molecule **3j** (0.08 ppm), followed by **3i** (0.25 ppm) and **3l** (0.32 ppm), while the largest change occurs for **3k** (0.43 ppm). Theoretical values yield the same picture (change of 0.09, 0.30, 0.37 and 0.52 ppm, respectively). From the above observations, we can conclude that in the investigated compounds, the nitro group is the one with the strongest electron-withdrawing character, followed closely by the cyano group.

Next, we analyze the compounds with a single substituent in different positions of the phenyl ring, that is, molecules **3c**–**3f**. Upon moving the chlorine atom from position 4 to position 2 of the phenyl ring (compounds **3c** and **3d**), we observe a shift of approximately 0.09 ppm toward high field (lower ppm values), corresponding to an increase in the electron shielding. When the bromine atom is moved from position 4 to position 3 in the phenyl ring (compounds **3e** and **3f**), we see opposite behaviour, with a change in thiazole-5H signal position by approximately 0.08 ppm toward low field, which means a decrease in electron shielding. This observation can be explained by the fact that the chlorine atom in the *ortho* position can donate electrons in a resonance effect, increasing the shielding. This effect is slightly smaller than in the case of the fluorine atom in the *para* position (see compound **3b**). Since the bromine atom can increase electron density through the resonance effect in the *ortho* and *para* positions, but not in the *meta* position, electron shielding around the thiazole-5H proton in **3f** is smaller than in **3e**.

For the two pairs: **3c**–**3d** and **3e**–**3f**, the opposite effect is obtained from calculations, namely a shift in the signal by 0.07 ppm towards low field when going from **3c** to **3d**, and by 0.05 ppm towards high field when going from **3e** to **3f**. This can be better understood when looking at the rmse value obtained for 4-phenylthiazole. While M06/aug-pcS-1 proved to be the most accurate among the tested methods, the error with respect to the experimental proton shifts exceeds 0.2 ppm, and thus, the prediction of changes as small as 0.1 ppm using this method can be difficult.

Finally, we turn our attention to the compounds with two substituents influencing the position of the thiazole-5H proton signal, that is, compounds **3n**–**3t**. Both experimental and theoretical results indicate that compound **3t**, containing two strongly electron-withdrawing groups, has the largest value of thiazole-5H proton shift. The thiazole-5H signal of compound **3q** with two methoxy groups is on the other end, with the value only slightly larger than that of **3m**. As could be anticipated, the signal for **3p**, that is, a compound with two fluorine substituents, is close to the signal of unsubstituted **3a**. It is interesting to investigate the position of the thiazole-5H proton NMR signal in pairs **3n**–**3o** and **3r**–**3s**. We can see that it depends strongly on the character of the substituents in the phenyl rings, and that the effect of the group in the phenyl ring attached to the thiazole is dominating. If a strong electron-withdrawing nitro group is present in this ring (compound **3s**), the observed proton shift is among the largest values reported here (7.65 ppm), despite the presence of an electron-donating group in the other phenyl ring. On the contrary, the presence of a nitro group attached to the other phenyl ring does not lead to a large value of this proton shift if a methoxy substituent is present in the phenyl ring attached to thiazole (compound **3r**, 7.36 ppm). A similar situation is observed for the pair **3n**–**3o**, with the corresponding proton shifts equal to 7.13 and 7.22 ppm, respectively.

The overall agreement between measured and calculated chemical shifts is good and confirms the usefulness of the quantum mechanical prediction of ^1^H NMR parameters. Nevertheless, theoretical results have to be taken with some caution, and conclusions based on computations should be drawn carefully, especially if the change in the signal position is subtle.

### 3.4. The Frontier Orbitals and Molecular Electrostatic Potential Surfaces

Molecular properties evaluated based on the energies of the highest occupied and lowest unoccupied molecular orbitals are presented in Table 3. The contours of the HOMO and LUMO orbitals, and the MEP surfaces for three representative molecules are presented in Table 4, and the complete set can be found in the Supplementary Materials (see Table S3).

We start the discussion with the HOMO-LUMO energy gap. Its value ranges from around 2.4 eV to around 4.3 eV, and divides investigated compounds into the following groups: compound **3s** with an energy gap of 2.4 eV, compounds **3k**, **3r** and **3t** with an energy gap in the range 2.7–3.0 eV, compound **3l** with an energy gap of 3.6 eV, and the remaining 15 compounds with an energy gap in the range 4.0–4.3 eV. All compounds containing a nitro group have an energy gap in the range of 2.4–3.0 eV, which corresponds to an electronic transition in the range of 415–515 nm and to the observed colour between red (smaller energy gap) and yellow (larger energy gap). This is consistent with the experimentally observed colour of compounds **3k** and **3r**–**3t**, all appearing from yellow to dark orange. For the gap of 3.6 eV and more, absorbed wavelengths are outside the visible region, and the compounds should be colourless, which is confirmed by experimental observations showing the remaining sixteen compounds to be either colourless or pale yellow.

Considering the electron affinity, its largest values correspond to containing nitro group compounds **3t**, **3k**, **3s** and **3r**, followed by compound **3l** containing cyano group. This indicates that the LUMOs in these species are low-energy and have a tendency to accept electrons. The smallest EAs are observed for compounds **3m**, **3q**, **3h** and **3n**, all containing strong electron-donating dimethylamino or methoxy groups. It is worth noting that exchanging the position of the methoxy and fluoro substituents (**3n** and **3o** compounds) increases the electron affinity value by 0.1 eV and substantially shifts it towards the EA of the reference compound **3a**. The ionization potential for most of the investigated compounds has values in the range 5.5–5.8 eV. The smallest (5.17 eV) and the largest (6.15 eV) IP values correspond to compounds **3m** and **3t**, respectively.

The largest values of chemical softness (the smallest hardness) correspond to the four compounds containing a nitro group, and the compound **3l**, containing a cyano group. This suggests that the chemical reactivity of these derivatives is the highest among the investigated systems. Moreover, these five compounds have the largest electronegativity, with the maximum value of 4.658 eV observed for **3t**, which contains two strongly electron-withdrawing nitro groups. The lowest value of χ corresponds to **3m**, i.e., to the derivative with two strongly electron-donating dimethylamino substituents. The electronegativity of **3q**, **3h**, **3n** and **3o**—all containing an electron-donating methoxy group—is only slightly larger.

An attempt was made to find a correlation between the calculated molecular parameters discussed in this section and the experimental chemical shifts (see Figure S2 in the Supplementary Materials). The results obtained for the compounds containing a single substituent in the 4-position show that there is a possible quadratic relationship between the HOMO-LUMO energy gap, ionization potential, electron affinity, electronegativity or chemical hardness, and the experimental values of chemical shift in the thiazole-5H proton. The R^2^ values are in the order of 0.93–0.95. Further investigation, covering a larger set of compounds, is necessary for molecules containing two substituents in the phenyl rings.

Analysis of the MEP surfaces reveals large differences in the charge distribution between the studied compounds. The presence of substituents with strong electron-withdrawing character or with electron-donating character leads to larger differences in charge distribution, see MEP surfaces of **3g**, **3h**, **3k**, **3l**, **3m**, and **3q**–**3t**. In these compounds, the electron-rich regions are located around the aromatic rings if electron donors are present (compounds **3g**, **3h**, **3m** and **3q**), or around the phenyl ring with strong electron-withdrawing substituents (compounds **3r**–**3t**). On the contrary, halogens’ presence results in more uniform charge distribution regardless of the substituent position in the molecule, see MEP surfaces of **3c**–**3f**. Surprisingly, the **3i** and **3j** compounds’ MEP surfaces also show relatively uniform charge distribution despite the strong electron-withdrawing character of the substituents. Finally, when both electron-withdrawing and -donating groups are present, the effect depends on the location of substituents, see **3n**, **3o**, **3r** and **3s** molecules, leading to substantial shifts in charge distribution. All the above observations can be of help when rationally designing materials with semiconducting or optical properties.

## 4. Conclusions

Thiazole derivatives are widely studied because of their possible applications as optoelectronic materials, with numerous interesting candidates reported in the previous literature. In particular, there is a focus on designing thiazole compounds with specific optical properties. In this work, we investigated the dependence between the position of the thiazole-5H proton signal in the NMR spectrum and the type and position of substituents present in the molecule. We have carefully chosen twenty 2,4-disubstituted thiazole derivatives with different substituents and performed DFT calculations to investigate their electronic structure in detail. To better understand the dependence of the chemical shift value on the type of substituents present in the molecule, we additionally calculated frontier molecular orbitals, global reactivity descriptors, and molecular electrostatic potential surfaces. Based on the obtained results we can conclude that the compounds with the smallest values of thiazole-5H proton shift (**3h**, **3m**, **3n** and **3q**) are simultaneously those with small electron affinity, ionization potential and molecular electronegativity, while compounds with the largest values of thiazole-5H proton shift (**3k**, **3l**, **3s** and **3t**) have large electron affinity, ionization potential, molecular electronegativity and small HOMO-LUMO energy gap. However, these relationships become less clear in the case of other investigated compounds, in particular those containing more than one substituent in two independent phenyl rings at 2,4-positions. Present research allows us to make a step towards a promising and easy-to-use tool for predicting global electronic effects in molecules containing thiazole, based on the position of the thiazole-5H proton NMR signal. The authors trust that such predictions could be helpful in the future design of organic optoelectronic materials.

## Figures and Tables

**Table 1:** Structures and selected physical properties of 2,4-disubstituted thiazole derivatives **3a**–**3t**.

Compound	Yield (%)	mp (°C)	R_f_	Colour	Thiazole-5H Proton Shift (ppm)
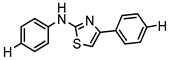 **3a**	99	130–132	0.69	white	7.32
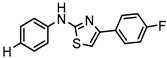 **3b**	89	127–129	0.88	white	7.30
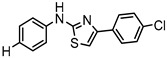 **3c**	77	141–143	0.91	white	7.42
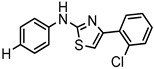 **3d**	91	118–120	0.90	white	7.33
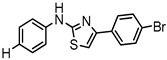 **3e**	88	144–146	0.87	white	7.44
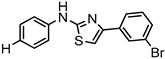 **3f**	91	86–87	0.88	light yellow	7.51
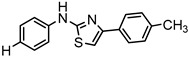 **3g**	78	91–93	0.95	sandy	7.23
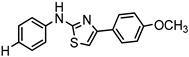 **3h**	99	142–143	0.71	sandy	7.14
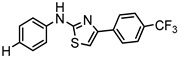 **3i**	81	148–149	0.88	white	7.57
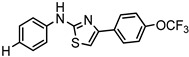 **3j**	99	129–130	0.90	white	7.41
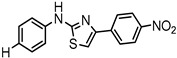 **3k**	98	204–205	0.73	orange	7.75
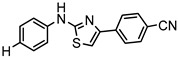 **3l**	94	201–203	0.54	white	7.64
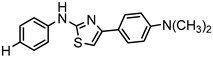 **3m**	71	168–169	0.70	light brown	6.97
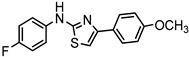 **3n**	90	139–140	0.61	light yellow	7.13
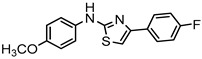 **3o**	87	185–186	0.64	off-white	7.22
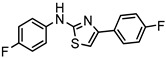 **3p**	87	155–157	0.69	white	7.30
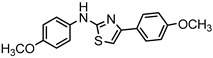 **3q**	89	182–184	0.55	white	7.06
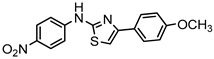 **3r**	95	197–199	0.64	dark orange	7.36
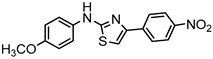 **3s**	97	171–173	0.55	yellow	7.65
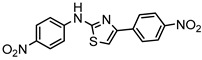 **3t**	99	>290	0.72	orange	7.92

**Table 2:** Proton chemical shifts of 4-phenylthiazole calculated at different levels of approximation. Experimental ^1^H NMR (400 MHz) values measured in DMSO-d_6_ for commercially available 4-phenylthiazole. All results in ppm. See Figure 4 for atom numbering.

Functional	BLYP						B3LYP					
Basis set	H_a_	H_b_	H_c_	H_d_	H_e_	rmse	H_a_	H_b_	H_c_	H_d_	H_e_	rmse
aug-cc-pVDZ	9.01	8.03	8.41	7.81	7.67	0.31	9.18	8.13	8.50	7.91	7.78	0.37
aug-cc-pVTZ	8.95	7.94	8.36	7.74	7.60	0.28	9.12	8.04	8.46	7.84	7.71	0.33
aug-cc-pVQZ	8.93	7.95	8.36	7.74	7.61	0.29	9.10	8.04	8.46	7.83	7.72	0.33
6-31G**	8.54	7.54	8.00	7.49	7.34	0.40	8.78	7.70	8.16	7.63	7.50	0.30
6-31++G**	8.70	7.77	8.28	7.70	7.56	0.34	8.90	7.90	8.42	7.82	7.68	0.35
6-311G**	8.62	7.68	8.17	7.62	7.48	0.36	8.83	7.82	8.30	7.74	7.61	0.32
6-311++G**	8.73	7.85	8.32	7.69	7.54	0.32	8.93	7.97	8.44	7.80	7.66	0.33
aug-pcS-1	8.69	7.80	8.21	7.55	7.38	0.30	8.87	7.90	8.32	7.66	7.49	0.26
aug-pcS-2	8.93	7.94	8.37	7.76	7.64	0.30	9.10	8.04	8.47	7.86	7.75	0.35
aug-pcSseg-1	8.70	7.81	8.25	7.58	7.41	0.30	8.88	7.92	8.35	7.68	7.52	0.28
aug-pcSseg-2	8.93	7.93	8.36	7.74	7.60	0.29	9.10	8.03	8.46	7.83	7.71	0.33
**Functional**	**M06**						**M06-2X**					
Basis set	H_a_	H_b_	H_c_	H_d_	H_e_	rmse	H_a_	H_b_	H_c_	H_d_	H_e_	rmse
aug-cc-pVDZ	9.60	8.38	8.55	8.09	7.94	0.52	9.78	8.69	9.13	8.41	8.17	0.85
aug-cc-pVTZ	9.57	8.29	8.53	7.99	7.74	0.43	9.73	8.63	9.10	8.42	8.24	0.84
aug-cc-pVQZ	9.55	8.30	8.55	8.05	7.72	0.45	9.70	8.63	9.05	8.43	8.30	0.84
6-31G**	9.26	7.99	8.28	7.81	7.62	0.26	9.49	8.39	8.86	8.33	8.18	0.70
6-31++G**	9.30	8.14	8.52	7.95	7.74	0.38	9.52	8.54	9.11	8.46	8.33	0.84
6-311G**	9.23	8.06	8.35	7.84	7.67	0.29	9.51	8.47	8.97	8.43	8.29	0.79
6-311++G**	9.32	8.16	8.53	7.91	7.61	0.35	9.58	8.57	9.11	8.46	8.25	0.83
aug-pcS-1	9.25	8.16	8.33	7.74	7.52	0.22	9.45	8.50	8.96	8.32	8.10	0.71
aug-pcS-2	9.57	8.40	8.62	8.07	8.00	0.54	9.73	8.59	9.06	8.43	8.41	0.87
aug-pcSseg-1	9.25	8.17	8.37	7.77	7.55	0.25	9.47	8.52	9.00	8.34	8.12	0.73
aug-pcSseg-2	9.62	8.42	8.62	8.06	7.86	0.52	9.71	8.57	9.05	8.43	8.31	0.84
Exp.	9.18	8.15	7.98	7.44	7.33							

**Table 3:** Predicted molecular properties of compounds **3a**–**3t** calculated for the lower energy conformer within the B3LYP/6-311++G** approximation. Calculations in DMSO employing PCM. Chemical softness in eV^−1^, all other values in eV.

System	∆E	IP	EA	χ	η	S
**3a**	4.26	5.66	1.39	3.52	2.13	0.47
**3b**	4.27	5.67	1.40	3.53	2.13	0.47
**3c**	4.15	5.68	1.53	3.61	2.07	0.48
**3d**	4.31	5.72	1.41	3.57	2.15	0.46
**3e**	4.14	5.69	1.55	3.62	2.07	0.48
**3f**	4.16	5.72	1.56	3.64	2.08	0.48
**3g**	4.28	5.60	1.32	3.46	2.14	0.47
**3h**	4.26	5.52	1.26	3.39	2.13	0.47
**3i**	3.96	5.75	1.80	3.77	1.98	0.51
**3j**	4.15	5.72	1.57	3.64	2.07	0.48
**3k**	2.69	5.82	3.13	4.48	1.34	0.74
**3l**	3.64	5.77	2.13	3.95	1.82	0.55
**3m**	4.02	5.17	1.15	3.16	2.01	0.50
**3n**	4.27	5.54	1.26	3.40	2.14	0.47
**3o**	4.09	5.45	1.36	3.41	2.04	0.49
**3p**	4.27	5.67	1.40	3.54	2.14	0.47
**3q**	4.15	5.36	1.21	3.29	2.08	0.48
**3r**	2.78	5.75	2.96	4.36	1.39	0.72
**3s**	2.43	5.56	3.13	4.34	1.22	0.82
**3t**	2.99	6.15	3.16	4.66	1.49	0.67

**Table 4:** The HOMO, LUMO and the MEP surfaces for three representative 2,4-disubstituted thiazole derivatives calculated at the B3LYP/6-311++G** level of approximation. PCM used to account for the solvent effects.

	HOMO	LUMO	MEP Surface
**3a**	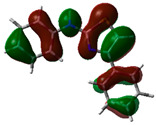	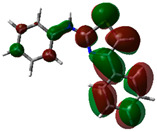	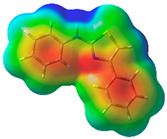
**3m**	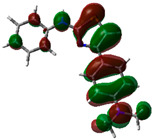	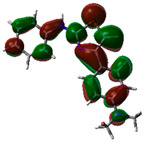	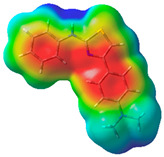
**3t**	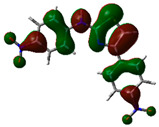	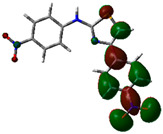	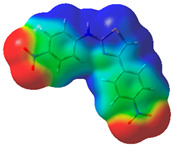

## Data Availability

The original contributions presented in this study are included in the article/Supplementary Material. Further inquiries can be directed to the corresponding author.

## Supplementary Materials

The following supporting information can be downloaded at: https://www.mdpi.com/article/10.3390/ma19112400/s1, Table S1: The B3LYP/6-311++G** optimized Cartesian coordinates of 4-phenylthiazole, tetramethylsilane and investigated compounds **3a**–**3t**. Calculations carried out in DMSO employing the polarizable continuum model. All values in Å; Table S2: Compounds **3a**–**3t**. The PCM B3LYP/6-311++G** relative energies [${∆}_{i}E$ (kcal mol^−1^)] and population fractions [$x_{i}$ (%)], and the PCM M06/aug-pcS-1 chemical shift in thiazole-5H proton: in i-th conformer [$\delta_{i}$ (ppm)], total calculated [Calcd.δ (ppm)], and experimental [Exp.δ (ppm)]; Table S3: The HOMO and LUMO contours, and the MEP surfaces for the investigated 2,4-disubstituted thiazole derivatives **3a**–**3t** calculated at the B3LYP/6-311++G** level of approximation for the lower energy structures. In the MEP surface, red colour denotes the electron-rich regions, and the blue colour denotes the electron-deficient regions; Figure S1: ^1^H and ^13^C NMR, and HRMS spectra of 4-phenylthiazole and the investigated compounds **3a**–**3t**; Figure S2: Correlation between calculated molecular properties and the experimental chemical shifts δ of thiazole-5H proton in compounds **3a**–**3m**. Symbol ∆E denotes HOMO–LUMO energy gap, IP—ionization potential, EA—electron affinity, χ—electronegativity, and η—chemical hardness.

## Abbreviations

The following abbreviations are used in this manuscript:

NMR	nuclear magnetic resonance
DMSO-d_6_	deuterated dimethyl sulfoxide
TMS	tetramethylsilane
J	spin-spin coupling constant
TLC	thin layer chromatography
HOMO	highest occupied molecular orbital
LUMO	lowest unoccupied molecular orbital
DFT	density functional theory
MEP	molecular electrostatic potential
mp	melting point
R_f_	retention factor
ESI	electrospray ionization
HRMS	high-resolution mass spectrometry
PCM	polarizable continuum model
IP	ionization potential
EA	electron affinity
χ	electronegativity
η	chemical hardness
S	chemical softness
GIAO	gauge-including atomic orbital
