# Supplementary material for: Theoretical and Experimental Study of the Effect of Functional Groups on the Thiazole-5H Proton Chemical Shift in 1H NMR Spectroscopy
Source: Materials (Basel). 2026 Jun 4;19(11):2400. doi: 10.3390/ma19112400 (PMC13257839; doi:10.3390/ma19112400)
Supplement: Supplementary file 1 [file materials-19-02400-s001.zip › materials-4310569-supplementary.pdf]

# Theoretical and Experimental Study of the Effect of Functional Groups on the Thiazole-5H Proton Chemical Shift in $^1$H NMR Spectroscopy

## Supplementary Materials

Angelika Baranowska-Łączkowska,[1] Krzysztof Z. Łączkowski[2]

[1]Department of Physics, Kazimierz Wielki University, Al. Powstańców Wielkopolskich 2, PL-85090 Bydgoszcz, Poland

[2]Department of Chemical Technology and Pharmaceuticals, Faculty of Pharmacy, Nicolaus Copernicus University, 2 Jurasz St., PL-85089 Bydgoszcz, Poland

**Contents:**

**Table S1.** The B3LYP/6-311++G** optimized Cartesian coordinates of 4-phenylthiazole, tetramethylsilane and investigated compounds **3a**–**3t**. Calculations carried out in DMSO employing the polarizable continuum model. All values in Å.

**Table S2.** Compounds **3a**-**3t**. The PCM B3LYP/6-311++G** relative energies [$\Delta_i E$ (kcal mol$^{-1}$)] and population fractions [$x_i$ (%)], and the PCM M06/aug-pcS-1 chemical shift in thiazole-5H proton: in $i$-th conformer [$\delta_i$ (ppm)], total calculated [$\mathrm{Calcd.}\,\delta$ (ppm)], and experimental [$\mathrm{Exp.}\,\delta$ (ppm)].

**Table S3.** The HOMO and LUMO contours, and the MEP surfaces for the investigated 2,4-disubstituted thiazole derivatives **3a**-**3t** calculated at the B3LYP/6-311++G** level of approximation for the lower energy structures. In the MEP surface, red colour denotes the electron-rich regions, and the blue colour denotes the electron-deficient regions.

**Figure S1.** $^1$H and $^{13}$C NMR, and HRMS spectra of 4-phenylthiazole and the investigated compounds **3a**–**3t**.

**Figure S2.** Correlation between calculated molecular properties and the experimental chemical shifts $\delta$ of thiazole-5H proton in compounds **3a**-**3m**. Symbol $\Delta E$ denotes HOMO–LUMO energy gap, $IP$ – ionization potential, $EA$ – electron affinity, $\chi$ – electronegativity, and $\eta$ – chemical hardness.

**Table S1.** The B3LYP/6-311++G** optimized Cartesian coordinates of 4-phenylthiazole, tetramethylsilane and investigated compounds **3a**–**3t**. Calculations carried out in DMSO employing the polarizable continuum model. All values in Å.

Phenylthiazole

| | | | |
|---|---|---|---|
| C | -2.613546 | 1.110127 | 0.180104 |
| C | -0.673106 | 0.044184 | 0.016052 |
| C | -1.515840 | -1.022842 | -0.170147 |
| C | 0.803386 | 0.002252 | 0.013158 |
| C | 1.499536 | -1.209480 | 0.149646 |
| C | 2.891078 | -1.237014 | 0.130048 |
| C | 3.617299 | -0.054470 | -0.020846 |
| C | 2.936491 | 1.155859 | -0.150234 |
| C | 1.543672 | 1.185550 | -0.132452 |
| N | -1.324257 | 1.252953 | 0.210086 |
| S | -3.165378 | -0.517992 | -0.094246 |
| H | -3.321819 | 1.916402 | 0.315235 |
| H | 4.700997 | -0.076975 | -0.033408 |
| H | -1.270642 | -2.055210 | -0.360863 |
| H | 0.955151 | -2.136594 | 0.286009 |
| H | 3.409331 | -2.182911 | 0.240546 |
| H | 3.490254 | 2.080816 | -0.266930 |
| H | 1.018755 | 2.126680 | -0.235219 |

TMS

| | | | |
|---|---|---|---|
| Si | 0.000000 | 0.000000 | 0.000000 |
| C | 1.091786 | 1.091786 | 1.091786 |
| C | -1.091786 | -1.091786 | 1.091786 |
| C | -1.091786 | 1.091786 | -1.091786 |
| C | 1.091786 | -1.091786 | -1.091786 |
| H | 1.736468 | 1.736468 | 0.485855 |
| H | 1.736468 | 0.485855 | 1.736468 |
| H | 0.485855 | 1.736468 | 1.736468 |
| H | -1.736468 | -1.736468 | 0.485855 |
| H | -1.736468 | -0.485855 | 1.736468 |
| H | -0.485855 | -1.736468 | 1.736468 |
| H | -1.736468 | 0.485855 | -1.736468 |
| H | -0.485855 | 1.736468 | -1.736468 |
| H | -1.736468 | 1.736468 | -0.485855 |
| H | 0.485855 | -1.736468 | -1.736468 |
| H | 1.736468 | -1.736468 | -0.485855 |
| H | 1.736468 | -0.485855 | -1.736468 |

Molecule **3a’**

| | | | |
|---|---|---|---|
| C | -0.668719 | 1.330078 | 0.035478 |
| S | 0.062362 | 2.948441 | 0.069608 |
| C | 1.625738 | 2.182504 | 0.052211 |

| | | | |
|---|---|---|---|
| C | 1.499025 | 0.823560 | 0.017713 |
| N | 0.190609 | 0.354367 | 0.013119 |
| C | 2.609994 | -0.148955 | 0.001536 |
| C | 3.925924 | 0.247581 | -0.289830 |
| C | 4.966057 | -0.676642 | -0.285163 |
| C | 4.715458 | -2.019614 | 0.004466 |
| C | 3.412204 | -2.426759 | 0.287145 |
| C | 2.369249 | -1.502384 | 0.284589 |
| N | -2.032866 | 1.229034 | 0.029914 |
| C | -3.254861 | -2.295185 | -0.105780 |
| C | -4.632052 | -2.086449 | -0.091606 |
| C | -5.118000 | -0.777833 | -0.037137 |
| C | -4.240787 | 0.298501 | 0.002827 |
| C | -2.850875 | 0.086196 | -0.011299 |
| C | -2.359144 | -1.225695 | -0.065997 |
| H | 2.517943 | 2.785970 | 0.087922 |
| H | 4.139689 | 1.281316 | -0.535205 |
| H | 5.973744 | -0.349417 | -0.515702 |
| H | 5.526393 | -2.739008 | 0.004637 |
| H | 3.204724 | -3.467277 | 0.511297 |
| H | 1.359317 | -1.821965 | 0.506838 |
| H | -2.532581 | 2.105598 | 0.059155 |
| H | -2.860806 | -3.304691 | -0.148181 |
| H | -5.316878 | -2.925575 | -0.122540 |
| H | -6.186037 | -0.591858 | -0.025432 |
| H | -4.628694 | 1.311247 | 0.044837 |
| H | -1.294065 | -1.396621 | -0.077495 |

Molecule **3a''**

| | | | |
|---|---|---|---|
| C | -0.527355 | -0.071809 | 0.003305 |
| S | -0.562950 | 1.690825 | -0.108141 |
| C | 1.178448 | 1.678359 | -0.115699 |
| C | 1.651146 | 0.398771 | -0.041925 |
| N | 0.674854 | -0.580408 | 0.032745 |
| C | 3.074064 | 0.002409 | -0.011720 |
| C | 4.091103 | 0.901211 | -0.373284 |
| C | 5.428701 | 0.520318 | -0.324592 |
| C | 5.780627 | -0.768851 | 0.080523 |
| C | 4.779211 | -1.672419 | 0.434344 |
| C | 3.439226 | -1.292345 | 0.387876 |
| N | -1.635071 | -0.877251 | -0.013699 |
| C | -3.003936 | -0.564671 | 0.014187 |
| C | -3.897700 | -1.513398 | -0.510441 |
| C | -5.267164 | -1.278468 | -0.489105 |
| C | -5.775327 | -0.092100 | 0.044656 |
| C | -4.890774 | 0.845694 | 0.572039 |
| C | -3.514943 | 0.615667 | 0.569985 |
| H | 1.717608 | 2.611356 | -0.131574 |

| | | | |
|---|---|---|---|
| H | 3.838696 | 1.900691 | -0.707953 |
| H | 6.197869 | 1.228956 | -0.610672 |
| H | 6.822846 | -1.065053 | 0.115164 |
| H | 5.040279 | -2.676898 | 0.748724 |
| H | 2.664219 | -1.995394 | 0.664765 |
| H | -1.404101 | -1.849200 | -0.173191 |
| H | -3.511027 | -2.432678 | -0.937104 |
| H | -5.939194 | -2.023613 | -0.899450 |
| H | -6.842674 | 0.093440 | 0.053382 |
| H | -5.267787 | 1.765115 | 1.005529 |
| H | -2.859974 | 1.343482 | 1.028432 |

Molecule **3b’**

| | | | |
|---|---|---|---|
| C | 1.154447 | 1.379177 | -0.036623 |
| S | 0.608442 | 3.069147 | -0.057933 |
| C | -1.030763 | 2.483079 | -0.041092 |
| C | -1.056096 | 1.118551 | -0.014881 |
| N | 0.190906 | 0.505615 | -0.016710 |
| C | -2.266522 | 0.273785 | -0.000764 |
| C | -3.534858 | 0.813354 | 0.269730 |
| C | -4.673338 | 0.014360 | 0.265621 |
| C | -4.530950 | -1.337732 | -0.006454 |
| C | -3.301092 | -1.915366 | -0.270460 |
| C | -2.170745 | -1.101078 | -0.264707 |
| N | 2.498286 | 1.124803 | -0.037387 |
| C | 3.316929 | -2.514180 | 0.116327 |
| C | 4.708675 | -2.461811 | 0.092123 |
| C | 5.338276 | -1.216613 | 0.024823 |
| C | 4.587354 | -0.048746 | -0.018044 |
| C | 3.182521 | -0.103133 | 0.006156 |
| C | 2.546916 | -1.350991 | 0.073671 |
| F | -5.643017 | -2.126270 | -0.008209 |
| H | -1.849279 | 3.183539 | -0.070349 |
| H | -3.642824 | 1.866462 | 0.498690 |
| H | -5.651896 | 0.426998 | 0.477036 |
| H | -3.230567 | -2.976216 | -0.476475 |
| H | -1.200665 | -1.532951 | -0.471074 |
| H | 3.094641 | 1.938528 | -0.072368 |
| H | 2.812157 | -3.472636 | 0.168736 |
| H | 5.295004 | -3.372403 | 0.125238 |
| H | 6.420333 | -1.151974 | 0.005102 |
| H | 5.086691 | 0.913348 | -0.070281 |
| H | 1.469605 | -1.401483 | 0.092676 |

Molecule **3b’’**

| | | | |
|---|---|---|---|
| C | -0.928910 | -0.032564 | 0.000503 |
| S | -1.020872 | 1.728052 | -0.105990 |
| C | 0.720200 | 1.771716 | -0.117983 |

| | | | |
|---|---|---|---|
| C | 1.232688 | 0.507488 | -0.049330 |
| N | 0.289276 | -0.502745 | 0.025082 |
| C | 2.666082 | 0.152897 | -0.025011 |
| C | 3.656741 | 1.082798 | -0.379445 |
| C | 5.006263 | 0.747552 | -0.339933 |
| C | 5.356888 | -0.534932 | 0.052898 |
| C | 4.415063 | -1.487368 | 0.402611 |
| C | 3.067996 | -1.135107 | 0.360008 |
| N | -2.009964 | -0.872798 | -0.014824 |
| C | -3.388270 | -0.604018 | 0.016175 |
| C | -4.251923 | -1.578950 | -0.510542 |
| C | -5.628195 | -1.388194 | -0.485820 |
| C | -6.172984 | -0.220722 | 0.053398 |
| C | -5.318007 | 0.743123 | 0.582585 |
| C | -3.935507 | 0.557341 | 0.577229 |
| F | 6.677629 | -0.870466 | 0.090161 |
| H | 1.228698 | 2.721861 | -0.132363 |
| H | 3.379736 | 2.078533 | -0.703475 |
| H | 5.772550 | 1.461145 | -0.615779 |
| H | 4.729258 | -2.479561 | 0.702075 |
| H | 2.315671 | -1.863816 | 0.631019 |
| H | -1.748571 | -1.836814 | -0.175600 |
| H | -3.836651 | -2.483477 | -0.941783 |
| H | -6.276797 | -2.152941 | -0.897780 |
| H | -7.245708 | -0.069541 | 0.064868 |
| H | -5.723574 | 1.648347 | 1.020129 |
| H | -3.303253 | 1.304065 | 1.037226 |

Molecule **3c'**

| | | | |
|---|---|---|---|
| C | 1.628708 | 1.381313 | -0.024037 |
| S | 1.237343 | 3.114194 | -0.042885 |
| C | -0.447093 | 2.678897 | -0.029870 |
| C | -0.596310 | 1.321843 | -0.009112 |
| N | 0.590148 | 0.598586 | -0.009053 |
| C | -1.877435 | 0.590088 | 0.001406 |
| C | -3.102536 | 1.251877 | 0.184084 |
| C | -4.305835 | 0.555912 | 0.180995 |
| C | -4.290431 | -0.824271 | -0.002844 |
| C | -3.095780 | -1.511742 | -0.180571 |
| C | -1.898037 | -0.801239 | -0.176128 |
| N | 2.943922 | 1.007437 | -0.023669 |
| C | 3.435982 | -2.691666 | 0.077395 |
| C | 4.827060 | -2.761985 | 0.061748 |
| C | 5.564680 | -1.576436 | 0.017310 |
| C | 4.920009 | -0.346327 | -0.010825 |
| C | 3.515842 | -0.277061 | 0.004954 |
| C | 2.771960 | -1.464454 | 0.049170 |
| Cl | -5.814458 | -1.714072 | -0.005489 |

| | | | |
|---|---|---|---|
| H | -1.198017 | 3.451590 | -0.051017 |
| H | -3.129092 | 2.323398 | 0.339728 |
| H | -5.242023 | 1.079838 | 0.325044 |
| H | -3.095648 | -2.585162 | -0.321201 |
| H | -0.964987 | -1.330509 | -0.315648 |
| H | 3.611554 | 1.764194 | -0.046672 |
| H | 2.848246 | -3.602445 | 0.111850 |
| H | 5.330424 | -3.721255 | 0.083694 |
| H | 6.648316 | -1.607452 | 0.004423 |
| H | 5.501988 | 0.569112 | -0.045032 |
| H | 1.694339 | -1.419836 | 0.061315 |

Molecule **3c''**

| | | | |
|---|---|---|---|
| C | -1.336495 | -0.011350 | 0.016605 |
| S | -1.478911 | 1.742447 | -0.141991 |
| C | 0.259410 | 1.834576 | -0.164111 |
| C | 0.808553 | 0.588142 | -0.057765 |
| N | -0.105718 | -0.445658 | 0.050653 |
| C | 2.250634 | 0.273394 | -0.033475 |
| C | 3.221720 | 1.250806 | -0.303261 |
| C | 4.578196 | 0.948082 | -0.270273 |
| C | 4.975253 | -0.351639 | 0.032536 |
| C | 4.040349 | -1.344871 | 0.299326 |
| C | 2.685059 | -1.026741 | 0.263736 |
| N | -2.394143 | -0.880373 | 0.034929 |
| C | -3.779350 | -0.644075 | 0.044159 |
| C | -4.613841 | -1.646168 | -0.477838 |
| C | -5.994470 | -1.486864 | -0.470483 |
| C | -6.571493 | -0.324518 | 0.045609 |
| C | -5.744988 | 0.666636 | 0.569779 |
| C | -4.358743 | 0.512239 | 0.582511 |
| Cl | 6.695447 | -0.743381 | 0.073961 |
| H | 0.739186 | 2.797915 | -0.221061 |
| H | 2.926448 | 2.262839 | -0.551753 |
| H | 5.315430 | 1.711752 | -0.482353 |
| H | 4.361014 | -2.352336 | 0.532188 |
| H | 1.951465 | -1.794504 | 0.470750 |
| H | -2.107722 | -1.841717 | -0.096467 |
| H | -4.172847 | -2.546473 | -0.892171 |
| H | -6.620954 | -2.272000 | -0.878285 |
| H | -7.647473 | -0.197994 | 0.043383 |
| H | -6.176245 | 1.568296 | 0.989709 |
| H | -3.748072 | 1.278879 | 1.039170 |

Molecule **3d'**

| | | | |
|---|---|---|---|
| C | -1.036998 | -1.261688 | -0.257758 |
| S | -0.206933 | -2.810558 | -0.511116 |
| C | 1.304405 | -1.948397 | -0.468268 |

| | | | |
|---|---|---|---|
| C | 1.094136 | -0.615574 | -0.271992 |
| N | -0.240062 | -0.238721 | -0.159887 |
| C | 2.110946 | 0.459533 | -0.247081 |
| C | 3.408210 | 0.350559 | 0.281764 |
| C | 4.307288 | 1.414516 | 0.253576 |
| C | 3.929645 | 2.629905 | -0.308073 |
| C | 2.645533 | 2.776107 | -0.830757 |
| C | 1.758452 | 1.708303 | -0.793716 |
| N | -2.402288 | -1.252868 | -0.174596 |
| C | -3.825144 | 2.143745 | 0.437190 |
| C | -5.184735 | 1.844110 | 0.482641 |
| C | -5.592184 | 0.519221 | 0.309124 |
| C | -4.655312 | -0.483620 | 0.094309 |
| C | -3.283289 | -0.179510 | 0.048036 |
| C | -2.870134 | 1.149047 | 0.221815 |
| Cl | 3.976152 | -1.130027 | 1.065525 |
| H | 2.228613 | -2.480588 | -0.612412 |
| H | 5.294740 | 1.287333 | 0.678235 |
| H | 4.633804 | 3.453174 | -0.328402 |
| H | 2.335844 | 3.718163 | -1.267615 |
| H | 0.760250 | 1.818465 | -1.197225 |
| H | -2.850519 | -2.150744 | -0.282427 |
| H | -3.491764 | 3.167162 | 0.570151 |
| H | -5.915990 | 2.625952 | 0.650142 |
| H | -6.645124 | 0.262843 | 0.341090 |
| H | -4.982603 | -1.509801 | -0.038446 |
| H | -1.819363 | 1.391021 | 0.187359 |

Molecule **3d''**

| | | | |
|---|---|---|---|
| C | -0.958307 | 0.226267 | -0.012326 |
| S | -0.902845 | -1.365805 | 0.748293 |
| C | 0.834525 | -1.249366 | 0.762272 |
| C | 1.239996 | -0.067102 | 0.216016 |
| N | 0.215675 | 0.759764 | -0.217478 |
| C | 2.626307 | 0.440786 | 0.103772 |
| C | 3.763142 | -0.335900 | -0.175511 |
| C | 5.036767 | 0.223800 | -0.253142 |
| C | 5.209362 | 1.589681 | -0.053736 |
| C | 4.100502 | 2.391411 | 0.213083 |
| C | 2.836302 | 1.820902 | 0.284731 |
| N | -2.107249 | 0.903211 | -0.324390 |
| C | -3.455298 | 0.517169 | -0.237782 |
| C | -4.415172 | 1.533305 | -0.098548 |
| C | -5.767960 | 1.220538 | -0.038484 |
| C | -6.192320 | -0.108363 | -0.104166 |
| C | -5.241658 | -1.116112 | -0.249048 |
| C | -3.882029 | -0.814305 | -0.326324 |
| Cl | 3.651725 | -2.071703 | -0.496558 |

| | | | |
|---|---|---|---|
| H | 1.419433 | -2.054271 | 1.172408 |
| H | 5.884164 | -0.411475 | -0.476546 |
| H | 6.202406 | 2.018980 | -0.113465 |
| H | 4.219446 | 3.457491 | 0.366372 |
| H | 1.972807 | 2.440331 | 0.490122 |
| H | -1.936682 | 1.876044 | -0.543663 |
| H | -4.092792 | 2.566940 | -0.033870 |
| H | -6.492579 | 2.019649 | 0.069206 |
| H | -7.246640 | -0.351504 | -0.049000 |
| H | -5.553652 | -2.152065 | -0.318453 |
| H | -3.173663 | -1.615386 | -0.486083 |

Molecule **3e'**

| | | | |
|---|---|---|---|
| C | 2.426400 | 1.366639 | -0.027260 |
| S | 2.195415 | 3.128255 | -0.064684 |
| C | 0.477988 | 2.849304 | -0.049962 |
| C | 0.205627 | 1.511942 | -0.016166 |
| N | 1.320195 | 0.682817 | -0.007913 |
| C | -1.137307 | 0.901694 | -0.001511 |
| C | -2.290100 | 1.665676 | 0.240871 |
| C | -3.553805 | 1.084922 | 0.240118 |
| C | -3.671428 | -0.281692 | -0.001060 |
| C | -2.550263 | -1.069078 | -0.237338 |
| C | -1.291238 | -0.472858 | -0.234561 |
| N | 3.700898 | 0.871375 | -0.017266 |
| C | 3.841364 | -2.858312 | 0.074727 |
| C | 5.219751 | -3.059054 | 0.071249 |
| C | 6.065907 | -1.947952 | 0.039294 |
| C | 5.539763 | -0.662736 | 0.010865 |
| C | 4.148334 | -0.461697 | 0.013622 |
| C | 3.295716 | -1.574035 | 0.046283 |
| Br | -5.415477 | -1.092992 | 0.000415 |
| H | -0.200215 | 3.686197 | -0.083812 |
| H | -2.211111 | 2.726532 | 0.445428 |
| H | -4.430494 | 1.689836 | 0.432003 |
| H | -2.648988 | -2.131028 | -0.422202 |
| H | -0.414875 | -1.079804 | -0.419603 |
| H | 4.438041 | 1.560992 | -0.035381 |
| H | 3.170463 | -3.710011 | 0.099736 |
| H | 5.630562 | -4.061446 | 0.093144 |
| H | 7.141903 | -2.080574 | 0.036293 |
| H | 6.204890 | 0.194439 | -0.013648 |
| H | 2.226965 | -1.428428 | 0.049685 |

Molecule **3e''**

| | | | |
|---|---|---|---|
| C | -2.096310 | 0.018301 | 0.014289 |
| S | -2.289134 | 1.768560 | -0.130478 |
| C | -0.554327 | 1.910472 | -0.157153 |

| | | | |
|---|---|---|---|
| C | 0.030355 | 0.679370 | -0.062083 |
| N | -0.853520 | -0.380892 | 0.041486 |
| C | 1.480773 | 0.405571 | -0.043850 |
| C | 2.422668 | 1.408310 | -0.323319 |
| C | 3.787816 | 1.143965 | -0.295667 |
| C | 4.222568 | -0.142962 | 0.011437 |
| C | 3.316898 | -1.161010 | 0.287309 |
| C | 1.952692 | -0.880514 | 0.256895 |
| N | -3.128340 | -0.880939 | 0.028885 |
| C | -4.519856 | -0.685859 | 0.042585 |
| C | -5.325172 | -1.710036 | -0.482444 |
| C | -6.709909 | -1.592214 | -0.471136 |
| C | -7.320222 | -0.450196 | 0.052058 |
| C | -6.522536 | 0.562786 | 0.579107 |
| C | -5.132243 | 0.449998 | 0.587817 |
| Br | 6.108880 | -0.517291 | 0.048952 |
| H | -0.102277 | 2.887508 | -0.207278 |
| H | 2.098170 | 2.410380 | -0.576054 |
| H | 4.498586 | 1.929821 | -0.515970 |
| H | 3.661938 | -2.159400 | 0.523604 |
| H | 1.241833 | -1.667607 | 0.471031 |
| H | -2.814381 | -1.832662 | -0.109336 |
| H | -4.858226 | -2.594617 | -0.902278 |
| H | -7.313615 | -2.393767 | -0.881339 |
| H | -8.399517 | -0.356032 | 0.053022 |
| H | -6.979556 | 1.449089 | 1.004437 |
| H | -4.543766 | 1.232418 | 1.046818 |

Molecule **3f’**

| | | | |
|---|---|---|---|
| C | -2.069282 | 1.333361 | 0.079114 |
| S | -1.354755 | 2.958656 | 0.148688 |
| C | 0.213250 | 2.208104 | 0.217580 |
| C | 0.099166 | 0.848459 | 0.179512 |
| N | -1.200752 | 0.365341 | 0.106005 |
| C | 1.216074 | -0.115288 | 0.227749 |
| C | 2.535532 | 0.295180 | -0.025943 |
| C | 3.566628 | -0.630541 | 0.044915 |
| C | 3.340082 | -1.969095 | 0.353648 |
| C | 2.027821 | -2.373960 | 0.594614 |
| C | 0.976652 | -1.463347 | 0.531995 |
| N | -3.429208 | 1.219076 | -0.000710 |
| C | -4.607158 | -2.316596 | -0.216813 |
| C | -5.985398 | -2.121361 | -0.268241 |
| C | -6.486691 | -0.818037 | -0.229440 |
| C | -5.623325 | 0.266515 | -0.140016 |
| C | -4.232485 | 0.067701 | -0.087758 |
| C | -3.725265 | -1.238704 | -0.126626 |
| Br | 5.367230 | -0.044654 | -0.309883 |

| | | | |
|---|---|---|---|
| H | 1.096755 | 2.819485 | 0.303350 |
| H | 2.751497 | 1.321690 | -0.290237 |
| H | 4.157972 | -2.675863 | 0.400574 |
| H | 1.828494 | -3.411995 | 0.834800 |
| H | -0.037287 | -1.788222 | 0.723092 |
| H | -3.939390 | 2.090097 | 0.003303 |
| H | -4.201350 | -3.321869 | -0.246380 |
| H | -6.659339 | -2.966958 | -0.337710 |
| H | -7.555834 | -0.642618 | -0.268617 |
| H | -6.023003 | 1.275112 | -0.110852 |
| H | -2.659355 | -1.399743 | -0.087238 |

Molecule **3f''**

| | | | |
|---|---|---|---|
| C | -1.962622 | 0.233857 | -0.063568 |
| S | -1.794780 | -1.523186 | -0.154428 |
| C | -0.068290 | -1.308334 | -0.208947 |
| C | 0.252037 | 0.018149 | -0.153503 |
| N | -0.828103 | 0.880098 | -0.079887 |
| C | 1.616729 | 0.581459 | -0.192194 |
| C | 2.738784 | -0.231049 | 0.039922 |
| C | 4.009737 | 0.322586 | -0.019457 |
| C | 4.217442 | 1.671221 | -0.295582 |
| C | 3.100194 | 2.475791 | -0.515967 |
| C | 1.815116 | 1.942759 | -0.464630 |
| N | -3.152461 | 0.893481 | 0.086862 |
| C | -4.473267 | 0.412510 | 0.088693 |
| C | -5.433182 | 1.147140 | 0.803527 |
| C | -6.762392 | 0.741096 | 0.817888 |
| C | -7.159906 | -0.408594 | 0.132011 |
| C | -6.209332 | -1.134068 | -0.582580 |
| C | -4.875441 | -0.727998 | -0.618235 |
| Br | 5.533155 | -0.811057 | 0.305310 |
| H | 0.572659 | -2.167577 | -0.321074 |
| H | 2.619301 | -1.279591 | 0.277669 |
| H | 5.216810 | 2.084070 | -0.334004 |
| H | 3.238929 | 3.529037 | -0.731262 |
| H | 0.955280 | 2.575582 | -0.639025 |
| H | -3.030329 | 1.860269 | 0.358776 |
| H | -5.128968 | 2.033644 | 1.349516 |
| H | -7.488359 | 1.321795 | 1.375530 |
| H | -8.194776 | -0.728685 | 0.150739 |
| H | -6.503261 | -2.019637 | -1.134541 |
| H | -4.170910 | -1.285143 | -1.220402 |

Molecule **3g'**

| | | | |
|---|---|---|---|
| C | -1.195889 | 1.375269 | -0.032324 |
| S | -0.674040 | 3.072698 | -0.052778 |
| C | 0.973590 | 2.508763 | -0.038241 |

| | | | |
|---|---|---|---|
| C | 1.019362 | 1.144227 | -0.013816 |
| N | -0.220819 | 0.515322 | -0.014524 |
| C | 2.241916 | 0.317548 | -0.000967 |
| C | 3.508396 | 0.877052 | 0.239385 |
| C | 4.651894 | 0.088088 | 0.231347 |
| C | 4.584965 | -1.292108 | -0.009922 |
| C | 3.323807 | -1.847416 | -0.244509 |
| C | 2.172215 | -1.061794 | -0.238911 |
| N | -2.536880 | 1.102736 | -0.032226 |
| C | -3.305337 | -2.547887 | 0.093996 |
| C | -4.697845 | -2.514245 | 0.081321 |
| C | -5.344639 | -1.277039 | 0.030871 |
| C | -4.609909 | -0.098737 | -0.006401 |
| C | -3.204206 | -0.134158 | 0.006161 |
| C | -2.551373 | -1.374049 | 0.056597 |
| C | 5.831064 | -2.142855 | 0.006017 |
| H | 1.782173 | 3.220669 | -0.064910 |
| H | 3.605216 | 1.937165 | 0.443800 |
| H | 5.615289 | 0.550998 | 0.420998 |
| H | 3.236798 | -2.911995 | -0.437370 |
| H | 1.208112 | -1.516778 | -0.426663 |
| H | -3.143871 | 1.908794 | -0.060212 |
| H | -2.787291 | -3.499898 | 0.133101 |
| H | -5.271569 | -3.432982 | 0.110250 |
| H | -6.427609 | -1.226889 | 0.020292 |
| H | -5.122504 | 0.857053 | -0.045257 |
| H | -1.473279 | -1.409680 | 0.066532 |
| H | 6.636685 | -1.678549 | -0.569268 |
| H | 6.201538 | -2.277815 | 1.027866 |
| H | 5.640771 | -3.133621 | -0.411079 |

Molecule **3g''**

| | | | |
|---|---|---|---|
| C | -0.954382 | -0.028609 | -0.001085 |
| S | -1.050405 | 1.731673 | -0.106552 |
| C | 0.691092 | 1.778731 | -0.116593 |
| C | 1.207663 | 0.515614 | -0.048180 |
| N | 0.264423 | -0.496147 | 0.024359 |
| C | 2.642008 | 0.166046 | -0.023758 |
| C | 3.632689 | 1.098646 | -0.372370 |
| C | 4.979977 | 0.760498 | -0.328342 |
| C | 5.397975 | -0.521225 | 0.057470 |
| C | 4.409648 | -1.449117 | 0.400129 |
| C | 3.056787 | -1.116690 | 0.359363 |
| N | -2.033658 | -0.872310 | -0.018965 |
| C | -3.412587 | -0.610148 | 0.014302 |
| C | -4.272781 | -1.594562 | -0.500973 |
| C | -5.649940 | -1.411251 | -0.474314 |
| C | -6.199967 | -0.242030 | 0.055883 |

| | | | |
|---|---|---|---|
| C | -5.348738 | 0.731193 | 0.573759 |
| C | -3.965166 | 0.553352 | 0.565989 |
| C | 6.861722 | -0.887196 | 0.076021 |
| H | 1.197765 | 2.729832 | -0.130461 |
| H | 3.352148 | 2.095865 | -0.691679 |
| H | 5.721441 | 1.503788 | -0.604062 |
| H | 4.699874 | -2.448944 | 0.707182 |
| H | 2.311029 | -1.852495 | 0.632311 |
| H | -1.768469 | -1.835246 | -0.179830 |
| H | -3.853761 | -2.500805 | -0.924982 |
| H | -6.295278 | -2.183393 | -0.877565 |
| H | -7.273482 | -0.096704 | 0.068994 |
| H | -5.758046 | 1.638218 | 1.004079 |
| H | -3.336111 | 1.308287 | 1.016758 |
| H | 7.036704 | -1.795029 | 0.656720 |
| H | 7.467198 | -0.084131 | 0.504304 |
| H | 7.234121 | -1.066324 | -0.938491 |

Molecule **3h’**

| | | | |
|---|---|---|---|
| C | 1.636733 | 1.374410 | -0.030998 |
| S | 1.201626 | 3.095364 | -0.056573 |
| C | -0.473461 | 2.615183 | -0.057780 |
| C | -0.587827 | 1.254636 | -0.031629 |
| N | 0.619492 | 0.564347 | -0.021606 |
| C | -1.847965 | 0.488046 | -0.025805 |
| C | -3.080904 | 1.095057 | 0.244121 |
| C | -4.272243 | 0.372309 | 0.231599 |
| C | -4.249714 | -0.997903 | -0.050869 |
| C | -3.024596 | -1.623339 | -0.314147 |
| C | -1.846855 | -0.891093 | -0.299501 |
| N | 2.962277 | 1.034039 | -0.018082 |
| C | 3.545428 | -2.651441 | 0.084696 |
| C | 4.937911 | -2.687234 | 0.095273 |
| C | 5.646181 | -1.483369 | 0.067996 |
| C | 4.971581 | -0.269670 | 0.030882 |
| C | 3.565862 | -0.234962 | 0.019965 |
| C | 2.851446 | -1.441180 | 0.047045 |
| O | -5.353782 | -1.798154 | -0.087240 |
| C | -6.632597 | -1.212071 | 0.167704 |
| H | -1.245006 | 3.366724 | -0.094638 |
| H | -3.126460 | 2.151464 | 0.482034 |
| H | -5.200104 | 0.882974 | 0.450183 |
| H | -3.014179 | -2.685535 | -0.529541 |
| H | -0.907348 | -1.386848 | -0.506896 |
| H | 3.609769 | 1.808233 | -0.034687 |
| H | 2.980074 | -3.576736 | 0.105547 |
| H | 5.464625 | -3.633708 | 0.124266 |
| H | 6.730331 | -1.487165 | 0.075776 |

| | | | |
|---|---|---|---|
| H | 5.531213 | 0.659953 | 0.010638 |
| H | 1.772929 | -1.423104 | 0.038899 |
| H | -7.352530 | -2.023293 | 0.080847 |
| H | -6.865648 | -0.437179 | -0.568237 |
| H | -6.679678 | -0.789351 | 1.175398 |

Molecule **3h''**

| | | | |
|---|---|---|---|
| C | -1.334055 | 0.039331 | -0.008123 |
| S | -1.517328 | 1.792870 | -0.092414 |
| C | 0.220335 | 1.926283 | -0.111299 |
| C | 0.799632 | 0.689351 | -0.059829 |
| N | -0.093469 | -0.367680 | 0.004453 |
| C | 2.247612 | 0.405627 | -0.051694 |
| C | 3.200040 | 1.411938 | -0.298145 |
| C | 4.557223 | 1.137899 | -0.278974 |
| C | 5.014005 | -0.161796 | -0.014196 |
| C | 4.085416 | -1.176990 | 0.227598 |
| C | 2.720655 | -0.885677 | 0.206177 |
| N | -2.368267 | -0.859244 | -0.024921 |
| C | -3.759061 | -0.677182 | 0.018012 |
| C | -4.562801 | -1.724864 | -0.463598 |
| C | -5.948052 | -1.624478 | -0.428428 |
| C | -6.563773 | -0.477511 | 0.077987 |
| C | -5.768708 | 0.558215 | 0.562470 |
| C | -4.376830 | 0.464876 | 0.544887 |
| O | 6.367075 | -0.332326 | -0.018506 |
| C | 6.891947 | -1.637032 | 0.238638 |
| H | 0.677069 | 2.902271 | -0.125194 |
| H | 2.880054 | 2.423704 | -0.517873 |
| H | 5.283104 | 1.918775 | -0.473311 |
| H | 4.404768 | -2.189672 | 0.433216 |
| H | 2.008685 | -1.678632 | 0.395596 |
| H | -2.051311 | -1.806774 | -0.183433 |
| H | -4.092783 | -2.615083 | -0.867915 |
| H | -6.548541 | -2.444484 | -0.805649 |
| H | -7.643974 | -0.397113 | 0.098252 |
| H | -6.228484 | 1.449861 | 0.973543 |
| H | -3.792204 | 1.268968 | 0.969346 |
| H | 7.974042 | -1.536850 | 0.184575 |
| H | 6.607251 | -1.987733 | 1.234780 |
| H | 6.553627 | -2.354007 | -0.514881 |

Molecule **3i'**

| | | | |
|---|---|---|---|
| C | 2.347919 | 1.375859 | -0.010799 |
| S | 2.097830 | 3.135956 | -0.044920 |
| C | 0.385336 | 2.839195 | -0.042629 |
| C | 0.126551 | 1.498574 | -0.016963 |
| N | 1.248826 | 0.680635 | -0.002666 |

| | | | |
|---|---|---|---|
| C | -1.210211 | 0.876656 | -0.014490 |
| C | -2.376494 | 1.640516 | 0.162244 |
| C | -3.628953 | 1.044178 | 0.150513 |
| C | -3.743272 | -0.336639 | -0.037947 |
| C | -2.596099 | -1.111826 | -0.206043 |
| C | -1.343285 | -0.508411 | -0.194793 |
| N | 3.627274 | 0.895362 | 0.006088 |
| C | 3.810038 | -2.833553 | 0.045601 |
| C | 5.190635 | -3.018336 | 0.044030 |
| C | 6.024176 | -1.897387 | 0.030956 |
| C | 5.483455 | -0.617950 | 0.018845 |
| C | 4.089902 | -0.433172 | 0.019684 |
| C | 3.249903 | -1.555273 | 0.033751 |
| C | -5.094884 | -0.982276 | 0.003112 |
| F | -5.132799 | -2.162588 | -0.658548 |
| F | -6.062140 | -0.199286 | -0.535812 |
| F | -5.501295 | -1.251405 | 1.277864 |
| H | -0.300955 | 3.669653 | -0.072267 |
| H | -2.311327 | 2.710073 | 0.317514 |
| H | -4.514573 | 1.652326 | 0.286780 |
| H | -2.676285 | -2.181598 | -0.352349 |
| H | -0.455391 | -1.110404 | -0.331736 |
| H | 4.356741 | 1.593373 | 0.001900 |
| H | 3.148879 | -3.693109 | 0.056148 |
| H | 5.612799 | -4.016194 | 0.053011 |
| H | 7.101604 | -2.017735 | 0.029607 |
| H | 6.138764 | 0.247049 | 0.008246 |
| H | 2.179577 | -1.422126 | 0.035939 |

Molecule **3i’’**

| | | | |
|---|---|---|---|
| C | 2.023516 | 0.009594 | -0.027890 |
| S | 2.213741 | 1.753713 | 0.190655 |
| C | 0.480168 | 1.892854 | 0.211406 |
| C | -0.103023 | 0.666442 | 0.059956 |
| N | 0.781590 | -0.388720 | -0.079888 |
| C | -1.552758 | 0.394308 | 0.024846 |
| C | -2.498057 | 1.415766 | 0.216662 |
| C | -3.858580 | 1.146111 | 0.179167 |
| C | -4.305620 | -0.157865 | -0.053685 |
| C | -3.381262 | -1.185468 | -0.242089 |
| C | -2.019083 | -0.909038 | -0.204807 |
| N | 3.058120 | -0.884582 | -0.075198 |
| C | 4.449096 | -0.679213 | -0.066522 |
| C | 5.256473 | -1.705068 | 0.450945 |
| C | 6.640330 | -1.574923 | 0.457942 |
| C | 7.246200 | -0.418748 | -0.038317 |
| C | 6.446047 | 0.596266 | -0.557904 |
| C | 5.057186 | 0.470879 | -0.585646 |

| | | | |
|---|---|---|---|
| C | -5.774101 | -0.456838 | -0.039271 |
| F | -6.095041 | -1.541121 | -0.784318 |
| F | -6.520592 | 0.574158 | -0.505266 |
| F | -6.237130 | -0.714583 | 1.218423 |
| H | 0.027240 | 2.865810 | 0.309718 |
| H | -2.175620 | 2.433060 | 0.399139 |
| H | -4.569474 | 1.949485 | 0.326518 |
| H | -3.719413 | -2.197939 | -0.423863 |
| H | -1.303338 | -1.705618 | -0.355921 |
| H | 2.748445 | -1.842419 | 0.025654 |
| H | 4.792384 | -2.600419 | 0.850508 |
| H | 7.246489 | -2.377831 | 0.861789 |
| H | 8.324550 | -0.315126 | -0.024699 |
| H | 6.900314 | 1.493423 | -0.962884 |
| H | 4.466265 | 1.254825 | -1.039150 |

Molecule **3j'**

| | | | |
|---|---|---|---|
| C | 2.725178 | 1.342351 | 0.059572 |
| S | 2.584618 | 3.109992 | 0.175799 |
| C | 0.861095 | 2.929462 | 0.023668 |
| C | 0.521624 | 1.612382 | -0.093022 |
| N | 1.588907 | 0.723741 | -0.074886 |
| C | -0.846207 | 1.080385 | -0.247089 |
| C | -1.976242 | 1.888170 | -0.038867 |
| C | -3.260196 | 1.381165 | -0.201406 |
| C | -3.414328 | 0.049088 | -0.563609 |
| C | -2.323562 | -0.778582 | -0.782065 |
| C | -1.042574 | -0.258552 | -0.618214 |
| N | 3.968625 | 0.777290 | 0.121015 |
| C | 3.908832 | -2.952041 | -0.045557 |
| C | 5.272080 | -3.230019 | 0.021482 |
| C | 6.175421 | -2.168996 | 0.119442 |
| C | 5.720390 | -0.856897 | 0.149012 |
| C | 4.344156 | -0.577805 | 0.081038 |
| C | 3.434366 | -1.639844 | -0.017672 |
| O | -4.718682 | -0.452265 | -0.799084 |
| C | -5.376462 | -1.015782 | 0.227783 |
| F | -6.573337 | -1.415033 | -0.217990 |
| F | -5.578631 | -0.166307 | 1.260383 |
| F | -4.741502 | -2.094275 | 0.739478 |
| H | 0.230459 | 3.803377 | 0.015927 |
| H | -1.861901 | 2.922908 | 0.259216 |
| H | -4.129346 | 2.007772 | -0.047115 |
| H | -2.472131 | -1.808634 | -1.080524 |
| H | -0.182249 | -0.891984 | -0.786315 |
| H | 4.738369 | 1.424828 | 0.207844 |
| H | 3.193897 | -3.763965 | -0.121620 |
| H | 5.627532 | -4.253330 | -0.001730 |

| | | | |
|---|---|---|---|
| H | 7.240942 | -2.361816 | 0.172951 |
| H | 6.430300 | -0.039719 | 0.225139 |
| H | 2.376777 | -1.434778 | -0.070596 |

Molecule **3j''**

| | | | |
|---|---|---|---|
| C | 2.364896 | 0.049957 | -0.075636 |
| S | 2.606737 | 1.765169 | 0.274799 |
| C | 0.880434 | 1.975523 | 0.201548 |
| C | 0.262808 | 0.787158 | -0.066858 |
| N | 1.114096 | -0.291909 | -0.228902 |
| C | -1.191480 | 0.580195 | -0.215868 |
| C | -2.115228 | 1.565858 | 0.167348 |
| C | -3.482115 | 1.367592 | 0.009939 |
| C | -3.927709 | 0.165877 | -0.524936 |
| C | -3.046243 | -0.829639 | -0.918717 |
| C | -1.679648 | -0.618448 | -0.757685 |
| N | 3.364607 | -0.883944 | -0.117543 |
| C | 4.760122 | -0.740673 | -0.033535 |
| C | 5.499656 | -1.837306 | 0.438857 |
| C | 6.885615 | -1.769849 | 0.517660 |
| C | 7.561665 | -0.607986 | 0.139070 |
| C | 6.829363 | 0.477281 | -0.336693 |
| C | 5.439354 | 0.416677 | -0.435498 |
| O | -5.315348 | -0.009864 | -0.752109 |
| C | -6.047489 | -0.589991 | 0.213367 |
| F | -7.315462 | -0.650291 | -0.209887 |
| F | -6.029321 | 0.095139 | 1.378965 |
| F | -5.645113 | -1.847896 | 0.503567 |
| H | 0.458938 | 2.960016 | 0.322818 |
| H | -1.772944 | 2.497323 | 0.600669 |
| H | -4.192465 | 2.131282 | 0.299850 |
| H | -3.420319 | -1.750090 | -1.348752 |
| H | -0.979111 | -1.385154 | -1.060045 |
| H | 3.012216 | -1.831961 | -0.093521 |
| H | 4.980487 | -2.738646 | 0.746524 |
| H | 7.438211 | -2.627041 | 0.885177 |
| H | 8.641453 | -0.553714 | 0.208793 |
| H | 7.338797 | 1.381089 | -0.651177 |
| H | 4.903850 | 1.258163 | -0.853119 |

Molecule **3k'**

| | | | |
|---|---|---|---|
| C | 1.914028 | 1.380226 | -0.018128 |
| S | 1.579817 | 3.128618 | -0.039056 |
| C | -0.112660 | 2.752823 | -0.026836 |
| C | -0.309546 | 1.399577 | -0.007905 |
| N | 0.849036 | 0.634314 | -0.005532 |
| C | -1.613648 | 0.721646 | 0.000417 |
| C | -2.818884 | 1.442332 | 0.105785 |

| | | | |
|---|---|---|---|
| C | -4.041495 | 0.793869 | 0.105227 |
| C | -4.069051 | -0.598211 | 0.000640 |
| C | -2.895353 | -1.342817 | -0.100953 |
| C | -1.677489 | -0.680007 | -0.099015 |
| N | 3.214414 | 0.964790 | -0.015476 |
| C | 3.586160 | -2.749707 | 0.044945 |
| C | 4.974350 | -2.863953 | 0.045449 |
| C | 5.750195 | -1.702454 | 0.026114 |
| C | 5.145517 | -0.451994 | 0.006521 |
| C | 3.744509 | -0.338918 | 0.005651 |
| C | 2.962200 | -1.501488 | 0.025167 |
| N | -5.356219 | -1.288140 | -0.000518 |
| O | -6.381420 | -0.613159 | 0.094625 |
| O | -5.362365 | -2.515405 | -0.097001 |
| H | -0.836456 | 3.551169 | -0.043287 |
| H | -2.806460 | 2.520708 | 0.195047 |
| H | -4.964889 | 1.349337 | 0.187226 |
| H | -2.942033 | -2.419793 | -0.180215 |
| H | -0.759338 | -1.244945 | -0.178320 |
| H | 3.907456 | 1.698780 | -0.028504 |
| H | 2.969383 | -3.641534 | 0.059948 |
| H | 5.446495 | -3.839077 | 0.060707 |
| H | 6.832307 | -1.768208 | 0.026334 |
| H | 5.756589 | 0.444868 | -0.007930 |
| H | 1.886541 | -1.422991 | 0.025115 |

Molecule **3k’’**

| | | | |
|---|---|---|---|
| C | -1.606980 | -0.026577 | 0.031418 |
| S | -1.777913 | 1.723244 | -0.178656 |
| C | -0.046209 | 1.842182 | -0.205924 |
| C | 0.524675 | 0.607355 | -0.061803 |
| N | -0.370080 | -0.439107 | 0.077168 |
| C | 1.967630 | 0.323078 | -0.033769 |
| C | 2.923442 | 1.341605 | -0.209206 |
| C | 4.278773 | 1.061739 | -0.181200 |
| C | 4.693146 | -0.255636 | 0.023487 |
| C | 3.773835 | -1.288182 | 0.199606 |
| C | 2.419302 | -0.993146 | 0.170221 |
| N | -2.654799 | -0.902996 | 0.077982 |
| C | -4.042187 | -0.663986 | 0.068129 |
| C | -4.871049 | -1.639395 | -0.507492 |
| C | -6.251238 | -1.471885 | -0.515333 |
| C | -6.829126 | -0.327034 | 0.036845 |
| C | -6.006697 | 0.637936 | 0.614298 |
| C | -4.622222 | 0.472811 | 0.644531 |
| N | 6.122181 | -0.557657 | 0.052207 |
| O | 6.918564 | 0.368322 | -0.101869 |
| O | 6.470031 | -1.725040 | 0.229363 |

| | | | |
|---|---|---|---|
| H | 0.417762 | 2.810038 | -0.303409 |
| H | 2.610446 | 2.364504 | -0.372746 |
| H | 5.010842 | 1.845070 | -0.316806 |
| H | 4.119144 | -2.300343 | 0.356593 |
| H | 1.693663 | -1.782977 | 0.305908 |
| H | -2.365429 | -1.865392 | -0.039225 |
| H | -4.427468 | -2.524197 | -0.951087 |
| H | -6.875934 | -2.235724 | -0.964126 |
| H | -7.904224 | -0.193852 | 0.022236 |
| H | -6.440432 | 1.524085 | 1.063498 |
| H | -4.012196 | 1.213743 | 1.143356 |

Molecule **3l'**

| | | | |
|---|---|---|---|
| C | 1.466356 | 1.368050 | -0.019607 |
| S | 1.043562 | 3.095589 | -0.033896 |
| C | -0.629807 | 2.632295 | -0.020480 |
| C | -0.755938 | 1.271829 | -0.004421 |
| N | 0.441352 | 0.567854 | -0.006734 |
| C | -2.024077 | 0.523287 | 0.002836 |
| C | -3.263054 | 1.173817 | 0.144814 |
| C | -4.449814 | 0.459960 | 0.140804 |
| C | -4.427067 | -0.937683 | -0.003127 |
| C | -3.198725 | -1.600702 | -0.140457 |
| C | -2.016355 | -0.874823 | -0.135723 |
| N | 2.787125 | 1.019192 | -0.021120 |
| C | 3.347030 | -2.670927 | 0.066082 |
| C | 4.739117 | -2.715422 | 0.049950 |
| C | 5.454936 | -1.516521 | 0.009749 |
| C | 4.787770 | -0.298352 | -0.013843 |
| C | 3.382815 | -0.255349 | 0.002576 |
| C | 2.660692 | -1.455962 | 0.042511 |
| C | -5.647821 | -1.677836 | -0.006869 |
| N | -6.637511 | -2.277523 | -0.010266 |
| H | -1.393863 | 3.392132 | -0.035854 |
| H | -3.306403 | 2.248610 | 0.266793 |
| H | -5.394973 | 0.976251 | 0.252426 |
| H | -3.175051 | -2.677759 | -0.250511 |
| H | -1.070848 | -1.388224 | -0.243202 |
| H | 3.440669 | 1.788341 | -0.041540 |
| H | 2.776150 | -3.592440 | 0.097260 |
| H | 5.260014 | -3.665348 | 0.068350 |
| H | 6.538924 | -1.527619 | -0.003460 |
| H | 5.352772 | 0.627665 | -0.044893 |
| H | 1.582505 | -1.431644 | 0.055027 |

Molecule **3l''**

| | | | |
|---|---|---|---|
| C | -1.175506 | -0.039307 | 0.028973 |
| S | -1.317020 | 1.709379 | -0.198184 |

| | | | |
|---|---|---|---|
| C | 0.418489 | 1.799366 | -0.223678 |
| C | 0.967776 | 0.557439 | -0.066824 |
| N | 0.054529 | -0.472163 | 0.080429 |
| C | 2.407866 | 0.246722 | -0.032159 |
| C | 3.381306 | 1.243502 | -0.222653 |
| C | 4.732123 | 0.939433 | -0.187015 |
| C | 5.147025 | -0.383550 | 0.040773 |
| C | 4.187300 | -1.388582 | 0.230986 |
| C | 2.837031 | -1.071709 | 0.194018 |
| N | -2.236635 | -0.900651 | 0.082526 |
| C | -3.620357 | -0.646289 | 0.072825 |
| C | -4.462577 | -1.630812 | -0.467806 |
| C | -5.840866 | -1.449514 | -0.475231 |
| C | -6.404978 | -0.282044 | 0.043028 |
| C | -5.569868 | 0.692018 | 0.585765 |
| C | -4.186856 | 0.514301 | 0.614790 |
| C | 6.538203 | -0.702201 | 0.077474 |
| N | 7.665808 | -0.960423 | 0.107274 |
| H | 0.898299 | 2.758652 | -0.328217 |
| H | 3.086645 | 2.269209 | -0.404348 |
| H | 5.468761 | 1.718843 | -0.336158 |
| H | 4.501735 | -2.409895 | 0.406429 |
| H | 2.098072 | -1.847373 | 0.341245 |
| H | -1.959341 | -1.868089 | -0.021119 |
| H | -4.030380 | -2.534094 | -0.884717 |
| H | -6.475117 | -2.220883 | -0.896968 |
| H | -7.478722 | -0.138362 | 0.028801 |
| H | -5.992303 | 1.596586 | 1.008302 |
| H | -3.567917 | 1.265362 | 1.086503 |

Molecule **3m’**

| | | | |
|---|---|---|---|
| C | -2.045637 | 1.384536 | 0.037177 |
| S | -1.750748 | 3.133998 | 0.093142 |
| C | -0.041316 | 2.789552 | 0.068160 |
| C | 0.183643 | 1.442269 | 0.017077 |
| N | -0.967564 | 0.659225 | 0.003774 |
| C | 1.497346 | 0.778295 | -0.019757 |
| C | 2.691757 | 1.495618 | -0.194523 |
| C | 3.928704 | 0.869422 | -0.214971 |
| C | 4.044373 | -0.535225 | -0.072257 |
| C | 2.841922 | -1.255233 | 0.115930 |
| C | 1.611190 | -0.611285 | 0.133636 |
| N | -3.340318 | 0.938012 | 0.029534 |
| C | -3.626718 | -2.781956 | -0.078223 |
| C | -5.011951 | -2.929175 | -0.079377 |
| C | -5.814138 | -1.785814 | -0.045652 |
| C | -5.238586 | -0.522082 | -0.011107 |
| C | -3.839986 | -0.374793 | -0.008768 |

| | | | |
|---|---|---|---|
| C | -3.031561 | -1.520038 | -0.043562 |
| N | 5.271854 | -1.170226 | -0.132025 |
| C | 5.369878 | -2.574170 | 0.247644 |
| C | 6.493324 | -0.376157 | -0.075337 |
| H | 0.666316 | 3.601515 | 0.104017 |
| H | 2.665200 | 2.572107 | -0.322256 |
| H | 4.810627 | 1.479741 | -0.350076 |
| H | 2.861996 | -2.327795 | 0.250135 |
| H | 0.713743 | -1.199514 | 0.279106 |
| H | -4.048785 | 1.656473 | 0.056564 |
| H | -2.989342 | -3.659064 | -0.104634 |
| H | -5.461391 | -3.914756 | -0.106171 |
| H | -6.894551 | -1.876407 | -0.046365 |
| H | -5.870442 | 0.360079 | 0.014220 |
| H | -1.957959 | -1.414913 | -0.043410 |
| H | 6.391938 | -2.912800 | 0.086623 |
| H | 5.110329 | -2.745187 | 1.302079 |
| H | 4.715690 | -3.193748 | -0.370339 |
| H | 7.350142 | -1.039630 | -0.178926 |
| H | 6.594508 | 0.174956 | 0.870296 |
| H | 6.532670 | 0.343327 | -0.896671 |

Molecule **3m''**

| | | | |
|---|---|---|---|
| C | 1.725279 | 0.038822 | 0.004927 |
| S | 1.889288 | 1.793459 | 0.090858 |
| C | 0.149062 | 1.908762 | 0.085308 |
| C | -0.418243 | 0.665228 | 0.027907 |
| N | 0.489753 | -0.381598 | -0.023066 |
| C | -1.860023 | 0.364608 | 0.001066 |
| C | -2.832398 | 1.352994 | 0.221444 |
| C | -4.189358 | 1.069444 | 0.186003 |
| C | -4.660206 | -0.238996 | -0.084121 |
| C | -3.679249 | -1.238565 | -0.281515 |
| C | -2.324142 | -0.936242 | -0.243437 |
| N | 2.768517 | -0.850296 | 0.036244 |
| C | 4.157392 | -0.658237 | 0.000029 |
| C | 4.966410 | -1.706263 | 0.472870 |
| C | 6.351030 | -1.596719 | 0.445084 |
| C | 6.961844 | -0.440158 | -0.045427 |
| C | 6.162005 | 0.595885 | -0.521228 |
| C | 4.770622 | 0.493904 | -0.510472 |
| N | -6.012000 | -0.520872 | -0.161349 |
| C | -6.979742 | 0.475749 | 0.280926 |
| C | -6.452419 | -1.910115 | -0.196250 |
| H | -0.317734 | 2.880050 | 0.090413 |
| H | -2.531635 | 2.371362 | 0.441382 |
| H | -4.886763 | 1.873191 | 0.375856 |
| H | -3.971794 | -2.262285 | -0.469151 |

| | | | |
|---|---|---|---|
| H | -1.604304 | -1.729137 | -0.404160 |
| H | 2.458012 | -1.800514 | 0.191086 |
| H | 4.500471 | -2.604338 | 0.864401 |
| H | 6.954980 | -2.417363 | 0.815399 |
| H | 8.041588 | -0.352675 | -0.060050 |
| H | 6.617728 | 1.495312 | -0.919753 |
| H | 4.182920 | 1.299784 | -0.927072 |
| H | -6.867180 | 1.407286 | -0.278795 |
| H | -7.983834 | 0.099220 | 0.093455 |
| H | -6.890324 | 0.704665 | 1.352319 |
| H | -6.183875 | -2.460508 | 0.716552 |
| H | -7.535317 | -1.934179 | -0.305236 |
| H | -6.022629 | -2.436459 | -1.051878 |

Molecule **3n’**

| | | | |
|---|---|---|---|
| C | 1.193821 | 1.706979 | -0.032463 |
| S | 0.589026 | 3.375440 | -0.040600 |
| C | -1.029841 | 2.729888 | -0.043540 |
| C | -1.007444 | 1.364687 | -0.029000 |
| N | 0.262978 | 0.798781 | -0.027871 |
| C | -2.184310 | 0.475724 | -0.028037 |
| C | -3.466769 | 0.948645 | 0.276516 |
| C | -4.580147 | 0.110720 | 0.259119 |
| C | -4.425593 | -1.241930 | -0.063637 |
| C | -3.148688 | -1.734025 | -0.361990 |
| C | -2.049869 | -0.888187 | -0.342035 |
| N | 2.546271 | 1.496950 | -0.025532 |
| C | 3.453153 | -2.125351 | 0.092524 |
| C | 4.829674 | -2.001049 | 0.070806 |
| C | 5.455064 | -0.764950 | 0.017989 |
| C | 4.667046 | 0.378605 | -0.014300 |
| C | 3.264341 | 0.288582 | 0.006270 |
| C | 2.661954 | -0.976445 | 0.059969 |
| H | -3.612398 | 1.988031 | 0.547168 |
| H | -1.873004 | 3.400454 | -0.074679 |
| H | -5.550571 | 0.519315 | 0.505703 |
| H | -3.036038 | -2.783401 | -0.608700 |
| H | -1.069207 | -1.281395 | -0.577115 |
| H | 3.117360 | 2.328802 | -0.047856 |
| H | 2.997076 | -3.106824 | 0.134266 |
| H | 6.535627 | -0.698114 | 0.002131 |
| H | 5.145805 | 1.350736 | -0.055921 |
| H | 1.586557 | -1.056595 | 0.076298 |
| O | -5.444523 | -2.147612 | -0.108070 |
| C | -6.770894 | -1.700668 | 0.183164 |
| H | -7.407506 | -2.577210 | 0.081564 |
| H | -6.842426 | -1.314709 | 1.204080 |
| H | -7.092967 | -0.931846 | -0.525017 |

| | | | |
|---|---|---|---|
| F | 5.599969 | -3.128984 | 0.101992 |

Molecule **3n''**

| | | | |
|---|---|---|---|
| C | -0.958137 | -0.062323 | 0.037290 |
| S | -1.065914 | 1.694897 | -0.098607 |
| C | 0.676734 | 1.751714 | -0.106570 |
| C | 1.200493 | 0.492870 | -0.018139 |
| N | 0.262156 | -0.523276 | 0.072064 |
| C | 2.634998 | 0.148620 | 0.008343 |
| C | 3.619756 | 1.062224 | -0.386575 |
| C | 4.975461 | 0.741532 | -0.345965 |
| C | 5.375514 | -0.525128 | 0.093346 |
| C | 4.402991 | -1.454628 | 0.483673 |
| C | 3.057643 | -1.121195 | 0.439185 |
| N | -2.037199 | -0.907333 | 0.037851 |
| C | -3.413583 | -0.621005 | 0.028336 |
| C | -4.272230 | -1.539157 | -0.596569 |
| C | -5.646897 | -1.333363 | -0.608305 |
| C | -6.153233 | -0.193618 | -0.003544 |
| C | -5.337278 | 0.729560 | 0.624973 |
| C | -3.961183 | 0.507648 | 0.651499 |
| H | 3.336129 | 2.043786 | -0.748452 |
| H | 1.178165 | 2.705308 | -0.134085 |
| H | 5.700515 | 1.477611 | -0.665276 |
| H | 4.720497 | -2.434646 | 0.820625 |
| H | 2.316145 | -1.848029 | 0.745209 |
| H | -1.779011 | -1.871531 | -0.124868 |
| H | -3.859607 | -2.418166 | -1.078469 |
| H | -6.313094 | -2.038970 | -1.088490 |
| H | -5.767277 | 1.600441 | 1.103566 |
| H | -3.332293 | 1.206792 | 1.184922 |
| O | 6.671513 | -0.943256 | 0.170382 |
| C | 7.708010 | -0.034578 | -0.208939 |
| H | 7.699394 | 0.859544 | 0.421010 |
| H | 8.641986 | -0.573083 | -0.061872 |
| H | 7.615365 | 0.253900 | -1.259904 |
| F | -7.500383 | 0.020441 | -0.024545 |

Molecule **3o'**

| | | | |
|---|---|---|---|
| C | -0.141255 | 1.834730 | 0.035893 |
| S | 0.748062 | 3.374297 | 0.053061 |
| C | 2.228984 | 2.456803 | 0.035316 |
| C | 1.966872 | 1.117683 | 0.012503 |
| N | 0.619552 | 0.778173 | 0.017731 |
| C | 2.974330 | 0.038899 | -0.002093 |
| C | 4.322105 | 0.297602 | -0.301199 |
| C | 5.269233 | -0.721053 | -0.297113 |
| C | 4.853250 | -2.009075 | 0.003421 |

| | | | |
|---|---|---|---|
| C | 3.534883 | -2.313296 | 0.295554 |
| C | 2.598991 | -1.281371 | 0.289725 |
| N | -1.505424 | 1.865490 | 0.036771 |
| C | -3.050219 | -1.537247 | -0.060846 |
| C | -4.407672 | -1.204415 | -0.043842 |
| C | -4.774698 | 0.143841 | -0.000581 |
| C | -3.790943 | 1.129643 | 0.025928 |
| C | -2.430362 | 0.800391 | 0.009468 |
| C | -2.067587 | -0.554387 | -0.034834 |
| H | 4.642230 | 1.301076 | -0.553804 |
| H | 3.176660 | 2.969179 | 0.063544 |
| H | 6.308257 | -0.525202 | -0.530880 |
| H | 3.248668 | -3.332722 | 0.522975 |
| H | 1.563908 | -1.497877 | 0.517817 |
| H | -1.921426 | 2.784706 | 0.059540 |
| H | -2.768388 | -2.583329 | -0.094924 |
| H | -5.813572 | 0.443772 | 0.013394 |
| H | -4.093516 | 2.171266 | 0.059544 |
| H | -1.024388 | -0.828820 | -0.049014 |
| O | -5.290015 | -2.250552 | -0.071564 |
| C | -6.687818 | -1.959237 | -0.057943 |
| H | -7.192788 | -2.922893 | -0.084384 |
| H | -6.972754 | -1.424411 | 0.853189 |
| H | -6.979069 | -1.372035 | -0.934154 |
| F | 5.776650 | -3.012194 | 0.004784 |

Molecule **3o''**

| | | | |
|---|---|---|---|
| C | -0.115625 | -0.207966 | 0.047341 |
| S | -0.315124 | 1.524697 | -0.246938 |
| C | 1.423088 | 1.666589 | -0.277781 |
| C | 2.009388 | 0.449866 | -0.075967 |
| N | 1.126723 | -0.601659 | 0.115179 |
| C | 3.460919 | 0.181463 | -0.027281 |
| C | 4.401450 | 1.154559 | -0.403161 |
| C | 5.767135 | 0.897550 | -0.344733 |
| C | 6.186823 | -0.350301 | 0.090321 |
| C | 5.297347 | -1.342438 | 0.464712 |
| C | 3.932787 | -1.068413 | 0.402058 |
| N | -1.162798 | -1.080192 | 0.149250 |
| C | -2.547115 | -0.796673 | 0.063046 |
| C | -3.366387 | -1.636965 | -0.705828 |
| C | -4.732883 | -1.417403 | -0.778422 |
| C | -5.314744 | -0.337258 | -0.100994 |
| C | -4.506206 | 0.502995 | 0.667574 |
| C | -3.133823 | 0.260220 | 0.758853 |
| H | 4.073132 | 2.124482 | -0.755894 |
| H | 1.875058 | 2.636348 | -0.407270 |
| H | 6.494086 | 1.645305 | -0.636217 |

| | | | |
|---|---|---|---|
| H | 5.664169 | -2.305543 | 0.797563 |
| H | 3.220320 | -1.829420 | 0.690944 |
| H | -0.888891 | -2.049364 | 0.057304 |
| H | -2.925611 | -2.465869 | -1.248625 |
| H | -5.364833 | -2.069104 | -1.369964 |
| H | -4.925346 | 1.334843 | 1.216685 |
| H | -2.532957 | 0.895390 | 1.397260 |
| O | -6.664442 | -0.194070 | -0.248699 |
| C | -7.313366 | 0.884075 | 0.428763 |
| H | -6.925445 | 1.851014 | 0.095176 |
| H | -8.366893 | 0.806612 | 0.167974 |
| H | -7.198960 | 0.795424 | 1.513081 |
| F | 7.524389 | -0.608795 | 0.146730 |

Molecule **3p’**

| | | | |
|---|---|---|---|
| C | 0.677115 | 1.666504 | -0.036863 |
| S | -0.061229 | 3.280659 | -0.055968 |
| C | -1.621267 | 2.507261 | -0.039145 |
| C | -1.487253 | 1.149210 | -0.014420 |
| N | -0.177189 | 0.685708 | -0.017944 |
| C | -2.591422 | 0.169875 | -0.000381 |
| C | -3.906600 | 0.552999 | 0.309534 |
| C | -4.946062 | -0.371230 | 0.304712 |
| C | -4.655699 | -1.690438 | -0.007769 |
| C | -3.373867 | -2.115934 | -0.311143 |
| C | -2.344312 | -1.177563 | -0.304266 |
| N | 2.041165 | 1.569590 | -0.037074 |
| C | 3.249526 | -1.962746 | 0.095367 |
| C | 4.610576 | -1.723259 | 0.063239 |
| C | 5.129744 | -0.439385 | -0.000691 |
| C | 4.248266 | 0.633715 | -0.034068 |
| C | 2.858545 | 0.425825 | -0.003168 |
| C | 2.364515 | -0.884561 | 0.061960 |
| H | -4.128044 | 1.580375 | 0.571501 |
| H | -2.516203 | 3.107018 | -0.069325 |
| H | -5.960400 | -0.079682 | 0.546911 |
| H | -3.187074 | -3.156013 | -0.548131 |
| H | -1.336105 | -1.489790 | -0.541749 |
| H | 2.539311 | 2.446812 | -0.068251 |
| H | 2.877869 | -2.978765 | 0.145756 |
| H | 6.200772 | -0.282330 | -0.024372 |
| H | 4.642838 | 1.642682 | -0.084150 |
| H | 1.299838 | -1.054826 | 0.086573 |
| F | -5.669683 | -2.601578 | -0.009944 |
| F | 5.472816 | -2.782285 | 0.095329 |

Molecule **3p’’**

| | | | |
|---|---|---|---|
| C | -0.524953 | -0.067430 | 0.031764 |

| | | | |
|---|---|---|---|
| S | -0.638353 | 1.688454 | -0.125534 |
| C | 1.102680 | 1.749605 | -0.150655 |
| C | 1.629451 | 0.493685 | -0.048379 |
| N | 0.697052 | -0.524325 | 0.062946 |
| C | 3.066433 | 0.154060 | -0.026027 |
| C | 4.046264 | 1.088491 | -0.398446 |
| C | 5.399059 | 0.766591 | -0.362410 |
| C | 5.764258 | -0.507184 | 0.045382 |
| C | 4.833605 | -1.463580 | 0.413502 |
| C | 3.482986 | -1.124718 | 0.373994 |
| N | -1.602123 | -0.912972 | 0.053703 |
| C | -2.978934 | -0.624437 | 0.045566 |
| C | -3.840149 | -1.539703 | -0.579261 |
| C | -5.214407 | -1.330445 | -0.588169 |
| C | -5.716609 | -0.190151 | 0.018956 |
| C | -4.897478 | 0.730322 | 0.647536 |
| C | -3.522077 | 0.504440 | 0.671730 |
| H | 3.758278 | 2.077357 | -0.733634 |
| H | 1.600603 | 2.704215 | -0.198807 |
| H | 6.156876 | 1.483744 | -0.652082 |
| H | 5.158890 | -2.448707 | 0.724248 |
| H | 2.739186 | -1.856857 | 0.658836 |
| H | -1.345312 | -1.879353 | -0.098145 |
| H | -3.430264 | -2.418872 | -1.063151 |
| H | -5.883378 | -2.033585 | -1.068079 |
| H | -5.324655 | 1.601417 | 1.128216 |
| H | -2.890029 | 1.200487 | 1.205636 |
| F | -7.062980 | 0.027275 | 0.000611 |
| F | 7.088333 | -0.829643 | 0.079218 |

Molecule **3q’**

| | | | |
|---|---|---|---|
| C | 0.666705 | 1.931268 | -0.029415 |
| S | -0.087637 | 3.540130 | -0.040562 |
| C | -1.642420 | 2.750129 | -0.043830 |
| C | -1.495684 | 1.392835 | -0.027930 |
| N | -0.179846 | 0.942432 | -0.024805 |
| C | -2.588822 | 0.402433 | -0.026718 |
| C | -3.908183 | 0.759346 | 0.278464 |
| C | -4.942920 | -0.173951 | 0.261285 |
| C | -4.669111 | -1.507542 | -0.061494 |
| C | -3.353715 | -1.884286 | -0.360320 |
| C | -2.334170 | -0.944213 | -0.340787 |
| N | 2.029420 | 1.847759 | -0.020491 |
| C | 3.284252 | -1.671906 | 0.067569 |
| C | 4.664765 | -1.453656 | 0.051156 |
| C | 5.143276 | -0.140750 | 0.010950 |
| C | 4.245441 | 0.923993 | -0.012752 |
| C | 2.861986 | 0.709683 | 0.003100 |

| | | | |
|---|---|---|---|
| C | 2.387142 | -0.610265 | 0.043960 |
| H | -4.145359 | 1.781650 | 0.549469 |
| H | -2.543017 | 3.341148 | -0.076760 |
| H | -5.945644 | 0.147071 | 0.508196 |
| H | -3.148433 | -2.919573 | -0.606995 |
| H | -1.322491 | -1.248723 | -0.576207 |
| H | 2.519406 | 2.729800 | -0.037354 |
| H | 2.916025 | -2.690892 | 0.099097 |
| H | 6.203653 | 0.071010 | -0.002652 |
| H | 4.633709 | 1.936910 | -0.043882 |
| H | 1.324395 | -0.795904 | 0.057116 |
| O | -5.603891 | -2.500209 | -0.105314 |
| C | -6.964253 | -2.172663 | 0.187662 |
| H | -7.520961 | -3.102016 | 0.086071 |
| H | -7.068642 | -1.795166 | 1.208955 |
| H | -7.354017 | -1.434831 | -0.519465 |
| O | 5.456919 | -2.570040 | 0.076267 |
| C | 6.873992 | -2.395864 | 0.061856 |
| H | 7.297233 | -3.398154 | 0.087228 |
| H | 7.201720 | -1.885825 | -0.849109 |
| H | 7.213688 | -1.835474 | 0.938214 |

Molecule **3q''**

| | | | |
|---|---|---|---|
| C | -0.548208 | -0.214564 | 0.043489 |
| S | -0.730548 | 1.527531 | -0.196917 |
| C | 1.010482 | 1.655033 | -0.209396 |
| C | 1.585188 | 0.426745 | -0.045239 |
| N | 0.690025 | -0.621905 | 0.106728 |
| C | 3.032832 | 0.145047 | 0.003543 |
| C | 3.981061 | 1.073723 | -0.442661 |
| C | 5.348782 | 0.813008 | -0.379380 |
| C | 5.799038 | -0.407441 | 0.135632 |
| C | 4.864054 | -1.352058 | 0.577578 |
| C | 3.506178 | -1.078209 | 0.509928 |
| N | -1.602766 | -1.083220 | 0.106442 |
| C | -2.984849 | -0.791562 | 0.029135 |
| C | -3.809743 | -1.617790 | -0.749436 |
| C | -5.175665 | -1.392784 | -0.815190 |
| C | -5.752358 | -0.320828 | -0.120671 |
| C | -4.938756 | 0.505405 | 0.657403 |
| C | -3.566735 | 0.257332 | 0.741279 |
| H | 3.658577 | 2.019317 | -0.863044 |
| H | 1.473743 | 2.624502 | -0.293972 |
| H | 6.044345 | 1.558476 | -0.740000 |
| H | 5.220216 | -2.296367 | 0.972878 |
| H | 2.793764 | -1.815692 | 0.856796 |
| H | -1.333828 | -2.049808 | -0.020086 |
| H | -3.373444 | -2.440439 | -1.305303 |

| | | | |
|---|---|---|---|
| H | -5.811141 | -2.033949 | -1.414450 |
| H | -5.353432 | 1.330769 | 1.219563 |
| H | -2.962784 | 0.881904 | 1.387020 |
| O | 7.111272 | -0.765405 | 0.241422 |
| C | 8.110519 | 0.162474 | -0.187592 |
| H | 8.059412 | 1.091757 | 0.387064 |
| H | 9.065690 | -0.325434 | -0.004393 |
| H | 8.012862 | 0.383105 | -1.254442 |
| O | -7.102361 | -0.171140 | -0.262490 |
| C | -7.745667 | 0.898149 | 0.433819 |
| H | -7.356150 | 1.869131 | 0.114009 |
| H | -8.800372 | 0.827893 | 0.175682 |
| H | -7.627715 | 0.792470 | 1.516262 |

Molecule **3r’**

| | | | |
|---|---|---|---|
| C | 0.398864 | 2.089421 | -0.030874 |
| S | -0.426930 | 3.652087 | -0.029218 |
| C | -1.936789 | 2.790126 | -0.034717 |
| C | -1.727168 | 1.439158 | -0.027822 |
| N | -0.390630 | 1.059391 | -0.031103 |
| C | -2.768709 | 0.395782 | -0.029300 |
| C | -4.099990 | 0.683643 | 0.295371 |
| C | -5.087031 | -0.299776 | 0.276569 |
| C | -4.750933 | -1.613510 | -0.068197 |
| C | -3.421925 | -1.920506 | -0.387409 |
| C | -2.450373 | -0.931654 | -0.365917 |
| N | 1.776812 | 2.070236 | -0.026202 |
| C | 3.173388 | -1.366653 | 0.053339 |
| C | 4.533933 | -1.056572 | 0.039458 |
| C | 4.967136 | 0.273491 | 0.005257 |
| C | 4.031377 | 1.285872 | -0.015886 |
| C | 2.649118 | 0.992951 | -0.003800 |
| C | 2.231201 | -0.352022 | 0.031687 |
| H | -4.384239 | 1.689017 | 0.583845 |
| H | -2.864696 | 3.337725 | -0.061079 |
| H | -6.101432 | -0.032674 | 0.539294 |
| H | -3.168770 | -2.940688 | -0.651510 |
| H | -1.428180 | -1.183121 | -0.618029 |
| H | 2.219811 | 2.978027 | -0.042813 |
| H | 2.858407 | -2.400406 | 0.080864 |
| H | 6.023895 | 0.499510 | -0.004597 |
| H | 4.361909 | 2.317943 | -0.042386 |
| H | 1.178182 | -0.582998 | 0.042014 |
| O | -5.634679 | -2.650357 | -0.115862 |
| C | -7.006655 | -2.396602 | 0.197224 |
| H | -7.516350 | -3.351642 | 0.087949 |
| H | -7.117426 | -2.039724 | 1.225148 |
| H | -7.441432 | -1.669571 | -0.494573 |

| | | | |
|---|---|---|---|
| O | 6.710483 | -1.827519 | 0.048008 |
| N | 5.512829 | -2.125521 | 0.060940 |
| O | 5.112379 | -3.292427 | 0.091356 |

Molecule **3r’’**

| | | | |
|---|---|---|---|
| C | 0.268304 | 0.070543 | 0.106440 |
| S | 0.315580 | 1.812776 | 0.331047 |
| C | -1.422231 | 1.818666 | 0.294615 |
| C | -1.906819 | 0.550528 | 0.123937 |
| N | -0.933395 | -0.427532 | 0.022346 |
| C | -3.327769 | 0.161627 | 0.046478 |
| C | -4.342709 | 1.109031 | -0.133639 |
| C | -5.685450 | 0.741187 | -0.191549 |
| C | -6.041201 | -0.606779 | -0.072205 |
| C | -5.037474 | -1.568397 | 0.101738 |
| C | -3.705426 | -1.187970 | 0.158176 |
| N | 1.361136 | -0.771396 | 0.074719 |
| C | 2.721828 | -0.542376 | 0.013202 |
| C | 3.566430 | -1.667583 | 0.155651 |
| C | 4.937204 | -1.536073 | 0.104723 |
| C | 5.496229 | -0.268023 | -0.091039 |
| C | 4.683445 | 0.854679 | -0.244692 |
| C | 3.306013 | 0.722199 | -0.196163 |
| H | -4.095232 | 2.158363 | -0.244154 |
| H | -1.951568 | 2.750787 | 0.406385 |
| H | -6.435320 | 1.506840 | -0.335929 |
| H | -5.320467 | -2.610696 | 0.191896 |
| H | -2.940428 | -1.941482 | 0.294432 |
| H | 1.079397 | -1.743678 | 0.105469 |
| H | 3.128102 | -2.646287 | 0.312432 |
| H | 5.577875 | -2.398970 | 0.218389 |
| H | 5.130333 | 1.824802 | -0.410181 |
| H | 2.702134 | 1.603763 | -0.347509 |
| O | -7.320499 | -1.075152 | -0.116314 |
| C | -8.387641 | -0.139083 | -0.288277 |
| H | -8.296567 | 0.392934 | -1.239532 |
| H | -9.301544 | -0.729501 | -0.288819 |
| H | -8.418309 | 0.580015 | 0.535229 |
| O | 7.409753 | 1.006973 | -0.306603 |
| N | 6.937201 | -0.120780 | -0.140991 |
| O | 7.635910 | -1.130122 | -0.016051 |

Molecule **3s’**

| | | | |
|---|---|---|---|
| C | 0.949503 | 2.012695 | 0.000009 |
| S | 0.279216 | 3.663950 | -0.000026 |
| C | -1.308001 | 2.963813 | -0.000023 |
| C | -1.236211 | 1.598137 | 0.000006 |
| N | 0.049045 | 1.072874 | 0.000029 |

| | | | |
|---|---|---|---|
| C | -2.382933 | 0.678959 | 0.000021 |
| C | -3.709273 | 1.152622 | -0.000277 |
| C | -4.780859 | 0.277063 | -0.000251 |
| C | -4.532162 | -1.097152 | 0.000045 |
| C | -3.232398 | -1.600199 | 0.000331 |
| C | -2.168374 | -0.711321 | 0.000328 |
| N | 2.302610 | 1.857985 | -0.000009 |
| C | 3.371683 | -1.724716 | -0.000506 |
| C | 4.761843 | -1.577873 | -0.000112 |
| C | 5.308149 | -0.290950 | 0.000369 |
| C | 4.467002 | 0.819154 | 0.000431 |
| C | 3.074786 | 0.675790 | -0.000002 |
| C | 2.531401 | -0.617488 | -0.000461 |
| H | -3.910961 | 2.215773 | -0.000564 |
| H | -2.174316 | 3.604825 | -0.000043 |
| H | -5.797588 | 0.643402 | -0.000492 |
| H | -3.064900 | -2.668044 | 0.000568 |
| H | -1.154982 | -1.087213 | 0.000579 |
| H | 2.840611 | 2.712034 | 0.000029 |
| H | 2.950903 | -2.723558 | -0.000865 |
| H | 6.378040 | -0.134329 | 0.000712 |
| H | 4.907680 | 1.810742 | 0.000835 |
| H | 1.460822 | -0.749147 | -0.000773 |
| O | 5.494441 | -2.733360 | -0.000245 |
| C | 6.919116 | -2.633628 | 0.000110 |
| H | 7.288670 | -3.657190 | -0.000048 |
| H | 7.280462 | -2.116370 | -0.893821 |
| H | 7.280048 | -2.116802 | 0.894458 |
| O | -5.420984 | -3.234238 | 0.000052 |
| N | -5.658366 | -2.026144 | 0.000063 |
| O | -6.799032 | -1.562372 | 0.000086 |

Molecule **3s’’**

| | | | |
|---|---|---|---|
| C | -0.797949 | -0.216283 | 0.059615 |
| S | -1.053905 | 1.506787 | -0.271955 |
| C | 0.672650 | 1.702533 | -0.312383 |
| C | 1.300178 | 0.508503 | -0.084991 |
| N | 0.455464 | -0.568437 | 0.130916 |
| C | 2.754376 | 0.295246 | -0.044278 |
| C | 3.661854 | 1.343300 | -0.291035 |
| C | 5.028605 | 1.129776 | -0.249279 |
| C | 5.504982 | -0.150098 | 0.042228 |
| C | 4.634881 | -1.210338 | 0.290373 |
| C | 3.268059 | -0.981892 | 0.245538 |
| N | -1.825093 | -1.103222 | 0.193062 |
| C | -3.213568 | -0.826059 | 0.087123 |
| C | -4.005381 | -1.597799 | -0.774632 |
| C | -5.370816 | -1.375828 | -0.863825 |

| | | | |
|---|---|---|---|
| C | -5.974230 | -0.359903 | -0.109837 |
| C | -5.190720 | 0.413641 | 0.750540 |
| C | -3.820964 | 0.165368 | 0.856527 |
| H | 3.300907 | 2.337146 | -0.521400 |
| H | 1.093493 | 2.679992 | -0.482550 |
| H | 5.722991 | 1.935684 | -0.439713 |
| H | 5.027581 | -2.192329 | 0.513716 |
| H | 2.579907 | -1.793749 | 0.435632 |
| H | -1.540180 | -2.071664 | 0.129037 |
| H | -3.545264 | -2.373959 | -1.375840 |
| H | -5.985134 | -1.974185 | -1.525980 |
| H | -5.628795 | 1.193371 | 1.358104 |
| H | -3.235207 | 0.743222 | 1.560753 |
| O | -7.319160 | -0.208444 | -0.278972 |
| C | -7.991941 | 0.805959 | 0.470828 |
| H | -7.601305 | 1.799018 | 0.230604 |
| H | -9.037767 | 0.746626 | 0.176700 |
| H | -7.904735 | 0.625316 | 1.546068 |
| O | 7.348697 | -1.516178 | 0.345879 |
| N | 6.945868 | -0.382019 | 0.086565 |
| O | 7.698240 | 0.566611 | -0.137248 |

Molecule **3t'**

| | | | |
|---|---|---|---|
| C | 0.684196 | 2.179203 | -0.000136 |
| S | -0.056292 | 3.788773 | 0.000208 |
| C | -1.605561 | 3.016787 | 0.000297 |
| C | -1.472350 | 1.654724 | 0.000055 |
| N | -0.162456 | 1.196140 | -0.000175 |
| C | -2.573278 | 0.679879 | 0.000014 |
| C | -3.919514 | 1.091286 | 0.000194 |
| C | -4.948954 | 0.165893 | 0.000175 |
| C | -4.635055 | -1.194138 | -0.000014 |
| C | -3.313746 | -1.635991 | -0.000190 |
| C | -2.292519 | -0.697867 | -0.000196 |
| N | 2.055906 | 2.089504 | -0.000267 |
| C | 3.277294 | -1.415884 | 0.000047 |
| C | 4.650916 | -1.173545 | -0.000003 |
| C | 5.151454 | 0.132486 | -0.000186 |
| C | 4.267607 | 1.191325 | -0.000289 |
| C | 2.873582 | 0.967035 | -0.000205 |
| C | 2.387643 | -0.354036 | -0.000065 |
| H | -4.171660 | 2.143558 | 0.000359 |
| H | -2.499745 | 3.618384 | 0.000493 |
| H | -5.981795 | 0.483891 | 0.000327 |
| H | -3.095915 | -2.694660 | -0.000340 |
| H | -1.263274 | -1.027713 | -0.000362 |
| H | 2.544929 | 2.973705 | -0.000330 |
| H | 2.910476 | -2.432723 | 0.000175 |

| | | | |
|---|---|---|---|
| H | 6.218348 | 0.304545 | -0.000239 |
| H | 4.648988 | 2.206014 | -0.000420 |
| H | 1.324770 | -0.532713 | -0.000029 |
| O | -5.422966 | -3.371080 | 0.000214 |
| N | -5.717723 | -2.176422 | -0.000044 |
| O | -6.877951 | -1.766165 | -0.000331 |
| O | 6.786114 | -2.053977 | 0.000065 |
| N | 5.575957 | -2.292285 | 0.000146 |
| O | 5.116488 | -3.436798 | 0.000357 |

Molecule **3t''**

| | | | |
|---|---|---|---|
| C | 0.519885 | 0.141522 | 0.089425 |
| S | 0.620289 | 1.898727 | 0.171018 |
| C | -1.110482 | 1.955964 | 0.137118 |
| C | -1.633885 | 0.692394 | 0.077024 |
| N | -0.696915 | -0.322561 | 0.047762 |
| C | -3.064587 | 0.348996 | 0.031821 |
| C | -4.061081 | 1.336718 | 0.135871 |
| C | -5.404084 | 1.002139 | 0.091916 |
| C | -5.761282 | -0.338497 | -0.056947 |
| C | -4.800063 | -1.341620 | -0.161631 |
| C | -3.458737 | -0.992229 | -0.116184 |
| N | 1.586397 | -0.727934 | 0.133368 |
| C | 2.955153 | -0.540189 | 0.056991 |
| C | 3.768176 | -1.667173 | 0.313238 |
| C | 5.142614 | -1.576149 | 0.258086 |
| C | 5.733901 | -0.347944 | -0.056267 |
| C | 4.952118 | 0.774469 | -0.324835 |
| C | 3.571163 | 0.681789 | -0.272541 |
| H | -3.791816 | 2.377749 | 0.257829 |
| H | -1.608920 | 2.911452 | 0.146869 |
| H | -6.168873 | 1.761282 | 0.173568 |
| H | -5.102537 | -2.372954 | -0.276206 |
| H | -2.701008 | -1.759215 | -0.196007 |
| H | 1.279926 | -1.686229 | 0.249920 |
| H | 3.303848 | -2.614376 | 0.561774 |
| H | 5.760419 | -2.439784 | 0.459127 |
| H | 5.424385 | 1.711971 | -0.582147 |
| H | 2.991577 | 1.559002 | -0.516244 |
| O | -7.475588 | -1.884747 | -0.235118 |
| N | -7.178405 | -0.698423 | -0.101750 |
| O | -8.012109 | 0.201033 | -0.004030 |
| O | 7.681193 | 0.852282 | -0.377696 |
| N | 7.180241 | -0.242320 | -0.110042 |
| O | 7.851043 | -1.252443 | 0.114513 |

**Table S2**. Compounds **3a**-**3t**. The PCM B3LYP/6-311++G** relative energies [$\Delta_i E$ (kcal mol$^{-1}$)] and population fractions [$x_i$ (%)], and the PCM M06/aug-pcS-1 chemical shift in thiazole-5H proton: in $i$-th conformer [$\delta_i$ (ppm)], total calculated [Calcd. $\delta$ (ppm)], and experimental [Exp. $\delta$ (ppm)].

| Conformer | $\Delta_i E$ | $x_i$ | $\delta_i$ | Conformer | $\Delta_i E$ | $x_i$ | $\delta_i$ |
|---|---|---|---|---|---|---|---|
| **3a'** | 0.0000 | 85.59 | 7.38 | **3k'** | 0.0000 | 87.25 | 7.90 |
| **3a''** | 1.0557 | 14.41 | 7.32 | **3k''** | 1.1394 | 12.75 | 7.89 |
| | | Calcd. $\delta$ | 7.38 | | | Calcd. $\delta$ | 7.90 |
| | | Exp. $\delta$ | 7.32 | | | Exp. $\delta$ | 7.75 |
| **3b'** | 0.0000 | 86.06 | 7.39 | **3l'** | 0.0000 | 86.83 | 7.75 |
| **3b''** | 1.0786 | 13.94 | 7.28 | **3l''** | 1.1173 | 13.17 | 7.72 |
| | | Calcd. $\delta$ | 7.38 | | | Calcd. $\delta$ | 7.74 |
| | | Exp. $\delta$ | 7.30 | | | Exp. $\delta$ | 7.64 |
| **3c'** | 0.0000 | 86.25 | 7.49 | **3m'** | 0.0000 | 84.36 | 7.09 |
| **3c''** | 1.0879 | 13.75 | 7.45 | **3m''** | 0.9984 | 15.64 | 7.13 |
| | | Calcd. $\delta$ | 7.48 | | | Calcd. $\delta$ | 7.09 |
| | | Exp. $\delta$ | 7.42 | | | Exp. $\delta$ | 6.97 |
| **3d'** | 0.0000 | 88.24 | 7.54 | **3n'** | 0.0000 | 89.25 | 7.22 |
| **3d''** | 1.1943 | 11.76 | 7.60 | **3n''** | 1.2541 | 10.75 | 7.07 |
| | | Calcd. $\delta$ | 7.55 | | | Calcd. $\delta$ | 7.20 |
| | | Exp. $\delta$ | 7.33 | | | Exp. $\delta$ | 7.13 |
| **3e'** | 0.0000 | 85.89 | 7.48 | **3o'** | 0.0000 | 75.68 | 7.23 |
| **3e''** | 1.0702 | 14.11 | 7.51 | **3o''** | 0.6726 | 24.32 | 7.21 |
| | | Calcd. $\delta$ | 7.48 | | | Calcd. $\delta$ | 7.23 |
| | | Exp. $\delta$ | 7.44 | | | Exp. $\delta$ | 7.22 |
| **3f'** | 0.0000 | 85.73 | 7.43 | **3p'** | 0.0000 | 89.39 | 7.31 |
| **3f''** | 1.0623 | 14.27 | 7.43 | **3p''** | 1.2629 | 10.61 | 7.24 |
| | | Calcd. $\delta$ | 7.43 | | | Calcd. $\delta$ | 7.30 |
| | | Exp. $\delta$ | 7.51 | | | Exp. $\delta$ | 7.30 |
| **3g'** | 0.0000 | 86.29 | 7.38 | **3q'** | 0.0000 | 76.14 | 7.09 |
| **3g''** | 1.0901 | 13.71 | 7.26 | **3q''** | 0.6875 | 23.86 | 7.02 |
| | | Calcd. $\delta$ | 7.37 | | | Calcd. $\delta$ | 7.07 |
| | | Exp. $\delta$ | 7.23 | | | Exp. $\delta$ | 7.06 |
| **3h'** | 0.0000 | 83.47 | 7.22 | **3r'** | 0.0000 | 93.75 | 7.34 |
| **3h''** | 0.9595 | 16.53 | 7.28 | **3r''** | 1.6042 | 6.25 | 7.51 |
| | | Calcd. $\delta$ | 7.23 | | | Calcd. $\delta$ | 7.35 |

| | | | | | | | |
|---|---|---|---|---|---|---|---|
| | | Exp. $\delta$ | 7.14 | | | Exp. $\delta$ | 7.36 |
| **3i'** | 0.0000 | 86.22 | 7.67 | **3s'** | 0.0000 | 75.30 | 7.87 |
| **3i''** | 1.0863 | 13.78 | 7.72 | **3s''** | 0.6605 | 24.70 | 7.73 |
| | | Calcd. $\delta$ | 7.67 | | | Calcd. $\delta$ | 7.83 |
| | | Exp. $\delta$ | 7.57 | | | Exp. $\delta$ | 7.65 |
| **3j'** | 0.0000 | 86.30 | 7.47 | **3t'** | 0.0000 | 94.60 | 8.06 |
| **3j''** | 1.0903 | 13.70 | 7.48 | **3t''** | 1.6959 | 5.40 | 8.12 |
| | | Calcd. $\delta$ | 7.47 | | | Calcd. $\delta$ | 8.06 |
| | | Exp. $\delta$ | 7.41 | | | Exp. $\delta$ | 7.92 |

**Table S3**. The HOMO and LUMO contours, and the MEP surfaces for the investigated 2,4-disubstituted thiazole derivatives **3a**-**3t** calculated at the B3LYP/6-311++G** level of approximation for the lower energy structures. In the MEP surface, red colour denotes the electron-rich regions, and the blue colour denotes the electron-deficient regions.

| | HOMO | LUMO | MEP surface |
|---|---|---|---|
| **3a** | 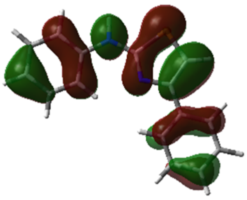 | 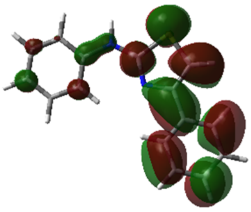 | 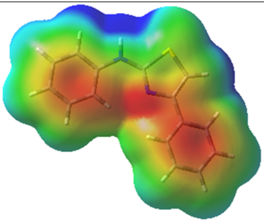 |
| **3b** | 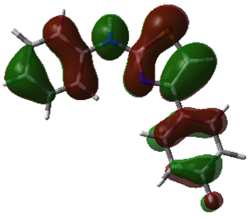 | 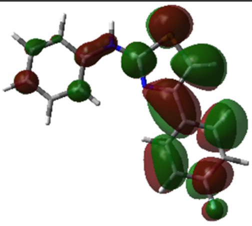 | 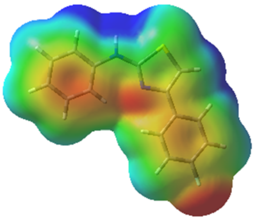 |
| **3c** | 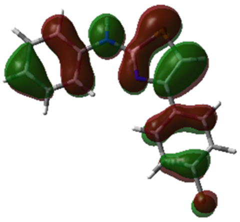 | 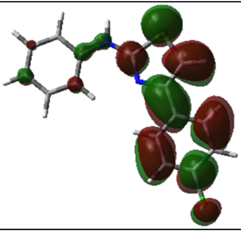 | 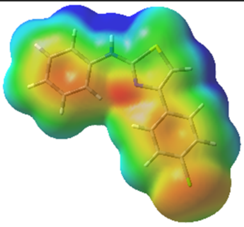 |
| **3d** | 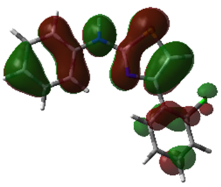 | 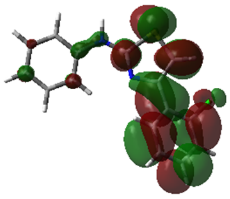 | 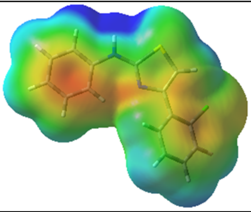 |
| **3e** | 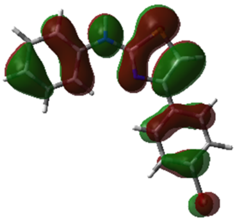 | 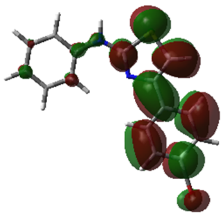 | 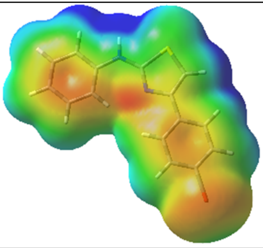 |
| **3f** | 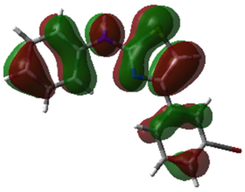 | 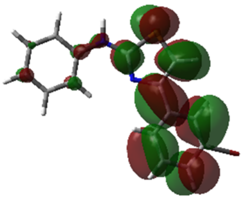 | 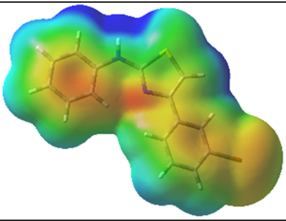 |
| **3g** | 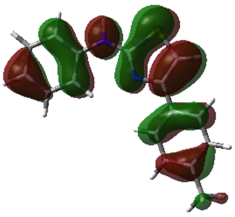 | 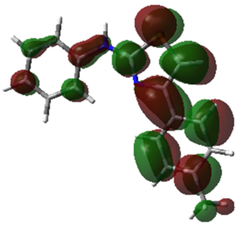 | 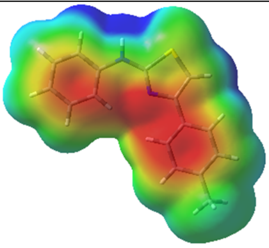 |

| | | | |
|---|---|---|---|
| **3h** | 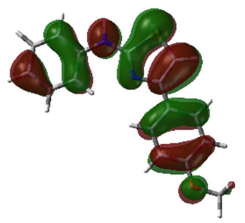 | 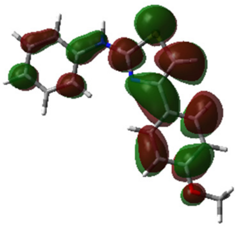 | 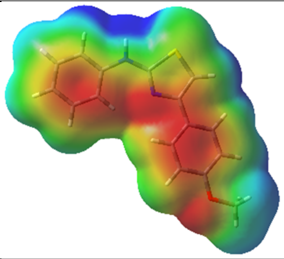 |
| **3i** | 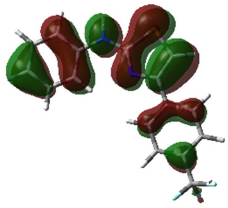 | 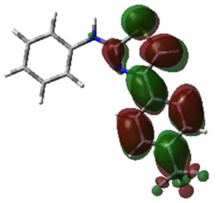 | 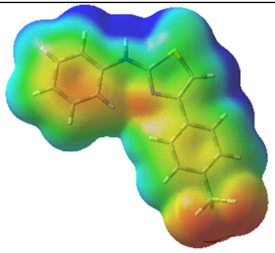 |
| **3j** | 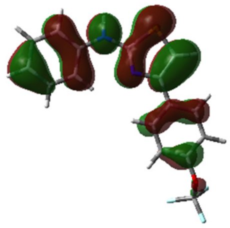 | 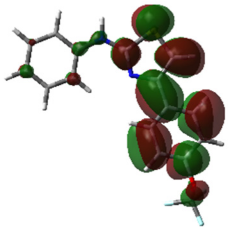 | 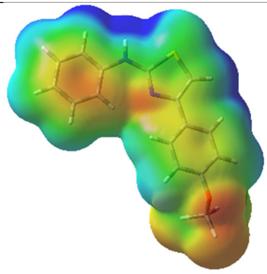 |
| **3k** | 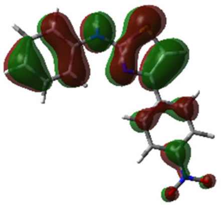 | 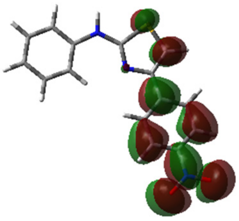 | 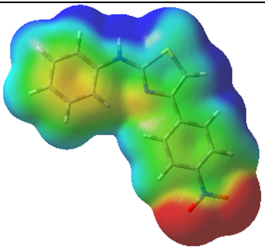 |
| **3l** | 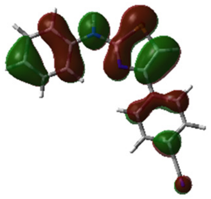 | 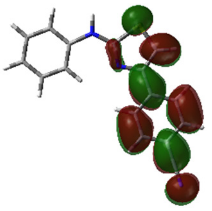 | 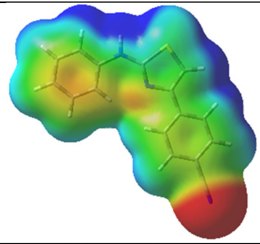 |
| **3m** | 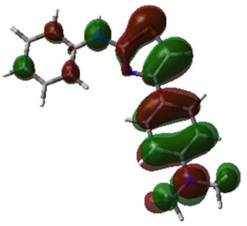 | 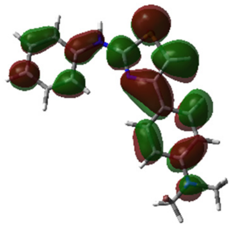 | 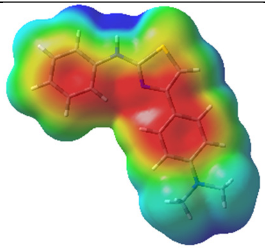 |
| **3n** | 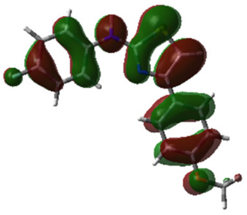 | 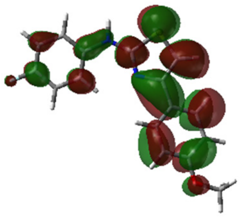 | 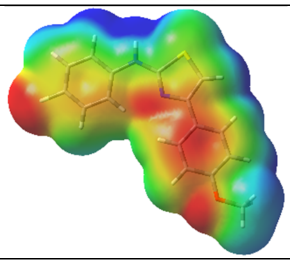 |

| | | | |
|---|---|---|---|
| **3o** | 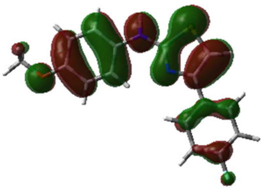 | 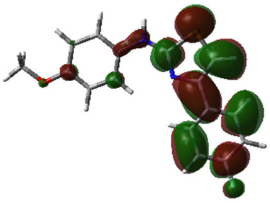 | 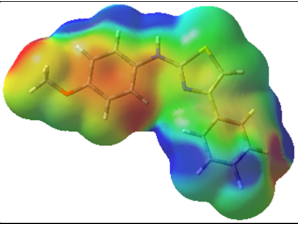 |
| **3p** | 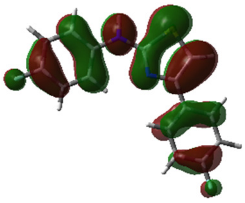 | 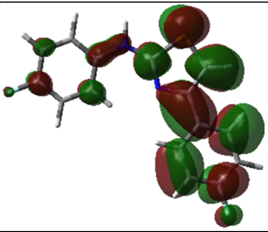 | 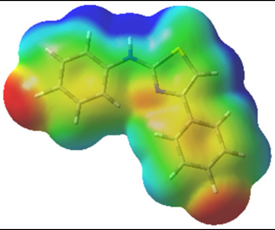 |
| **3q** | 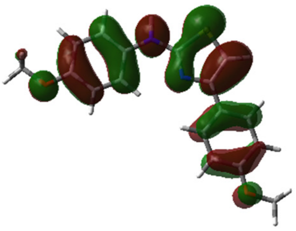 | 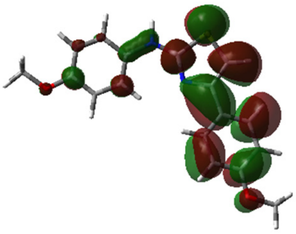 | 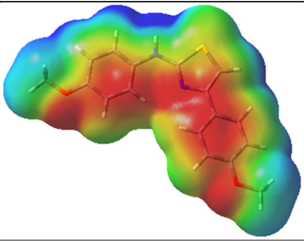 |
| **3r** | 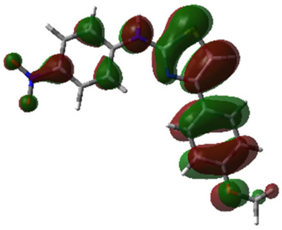 | 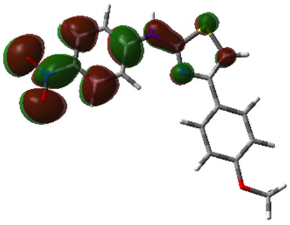 | 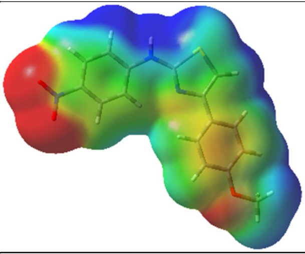 |
| **3s** | 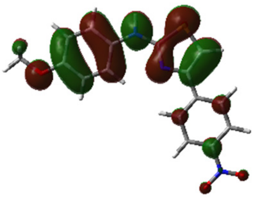 | 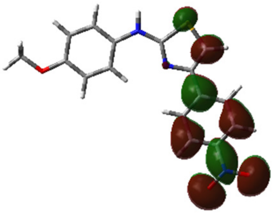 | 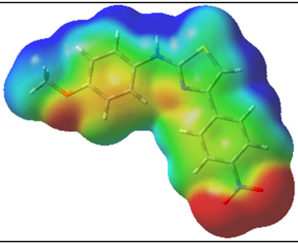 |
| **3t** | 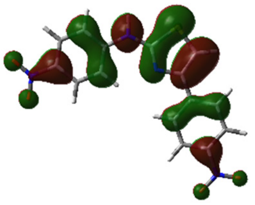 | 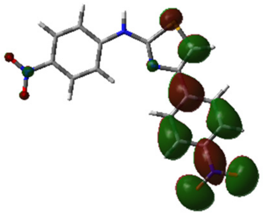 | 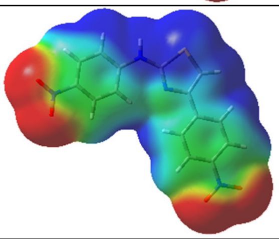 |

**Figure S1.** $^{1}H$ and $^{13}C$ NMR and HRMS spectra of 4-phenylthiazole and investigated compounds **3a**–**3t**.

**4-Phenylthiazole**

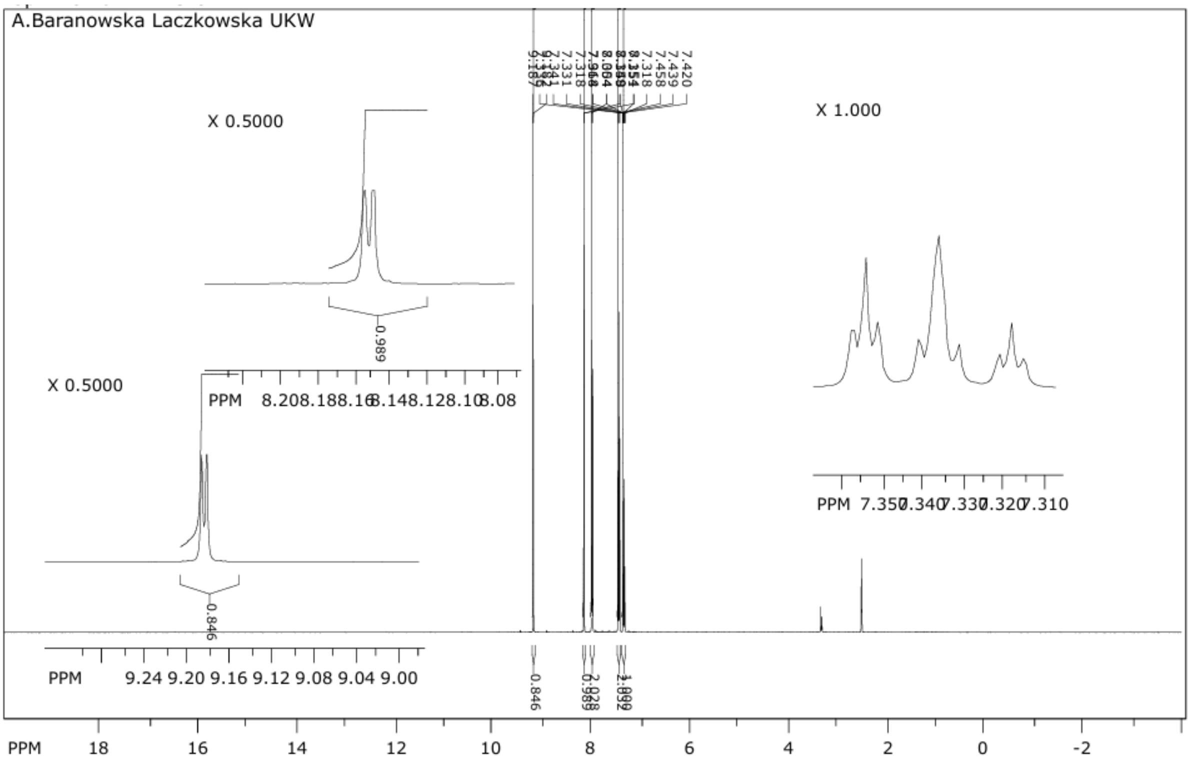


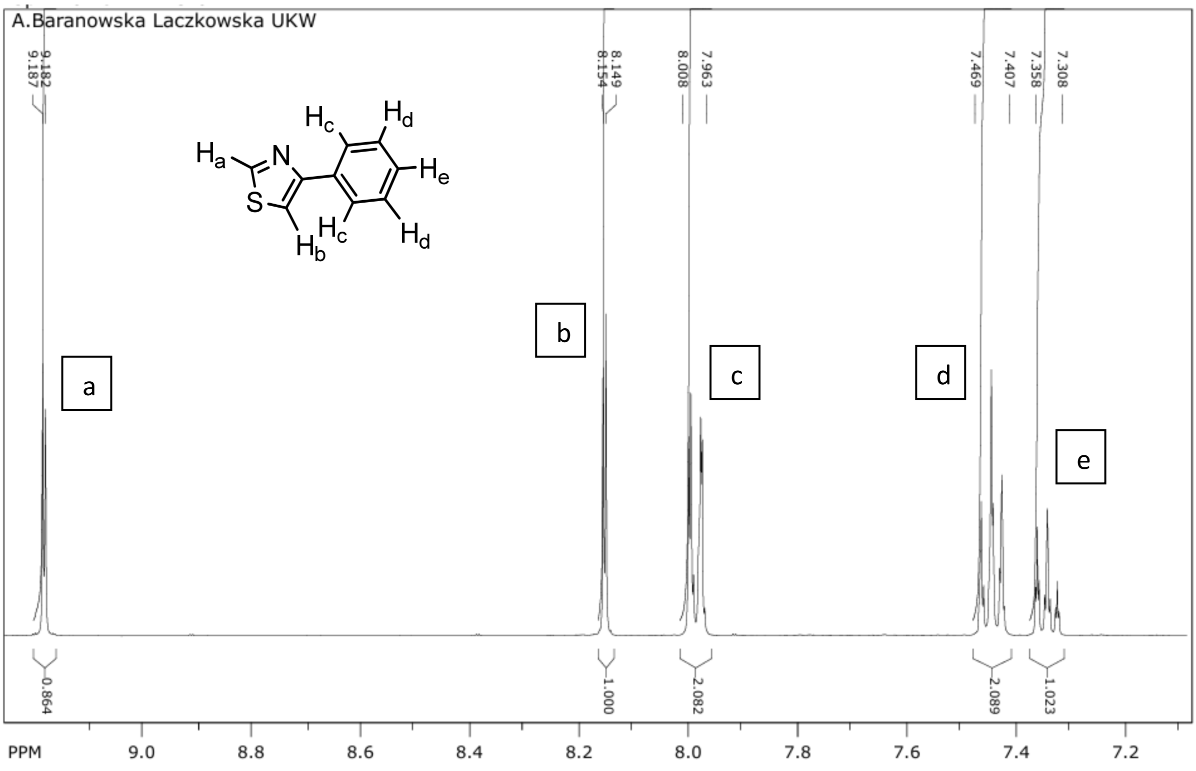

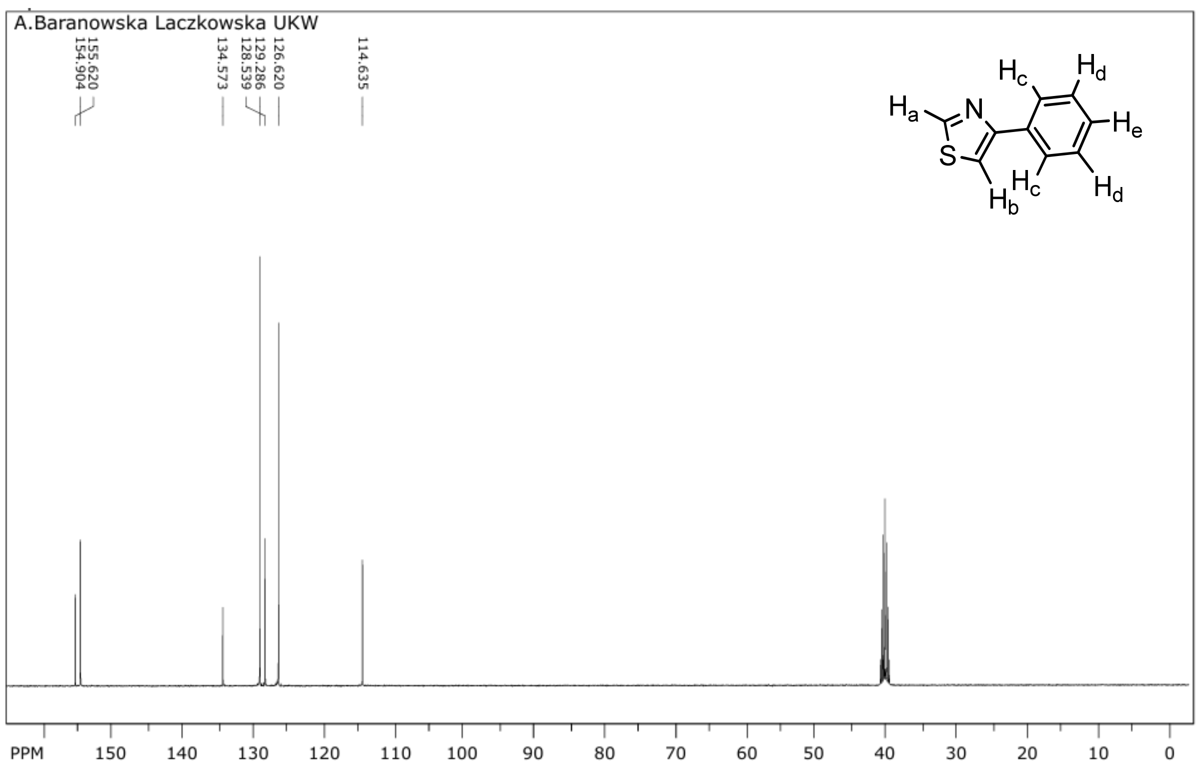
A.Baranowska Laczkowska UKW
155.620
154.904
134.573
129.286
128.539
126.620
114.635
PPM

**Compound 3a**

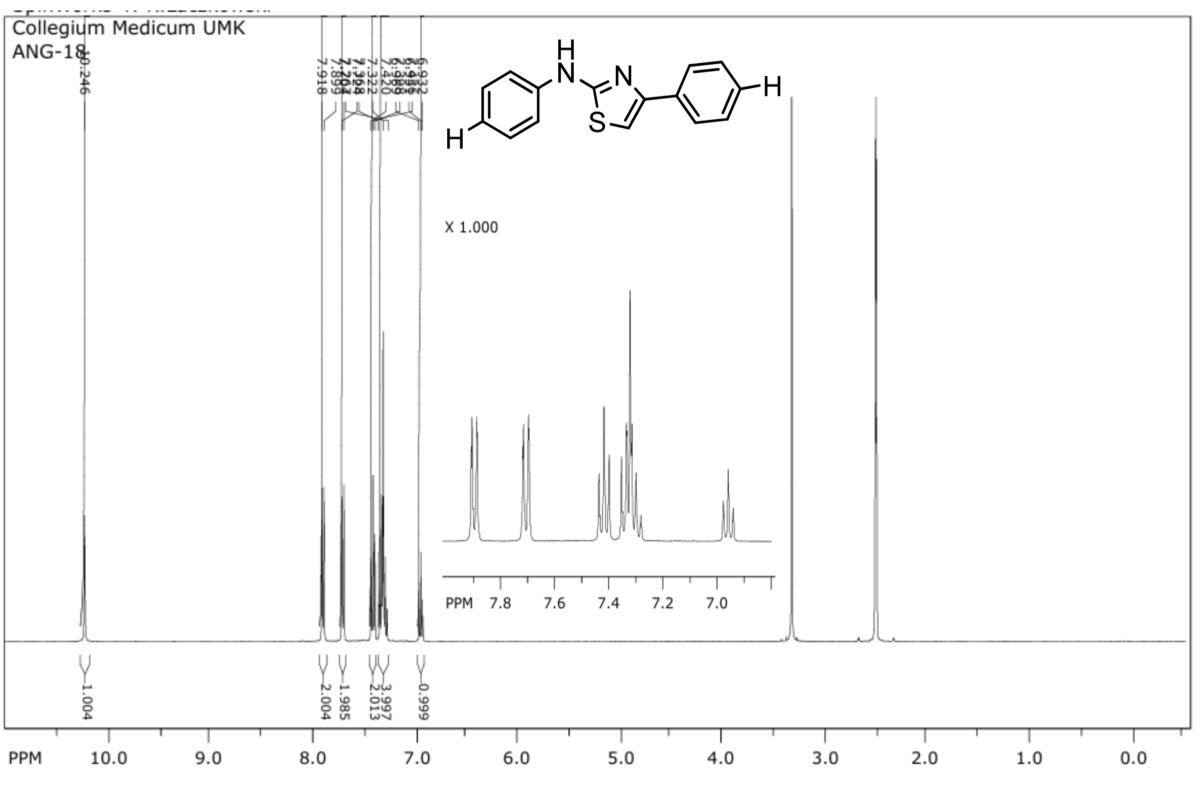
Collegium Medicum UMK
ANG-18
10.246
7.918
7.899
X 1.000
1.004
2.004
1.985
2.013
3.997
0.999
PPM

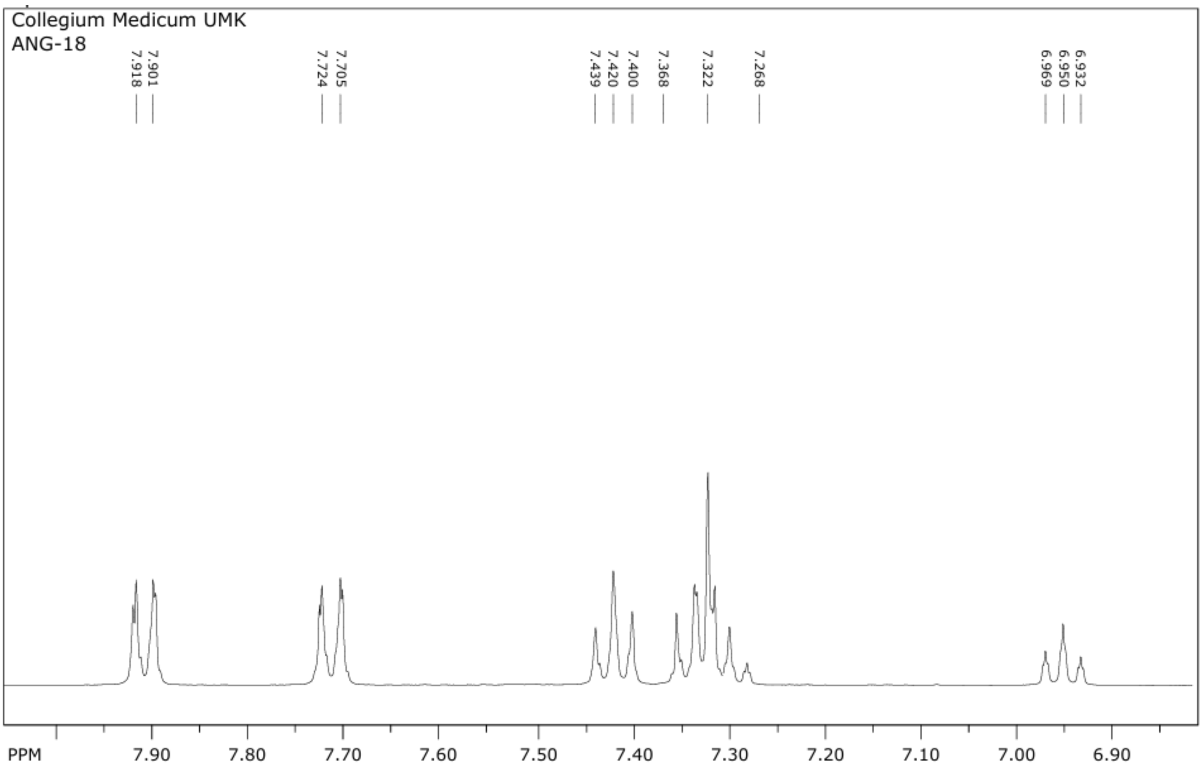
Collegium Medicum UMK
ANG-18
7.918
7.901
7.724
7.705
7.439
7.420
7.400
7.368
7.322
7.268
6.969
6.950
6.932
PPM
7.90
7.80
7.70
7.60
7.50
7.40
7.30
7.20
7.10
7.00
6.90

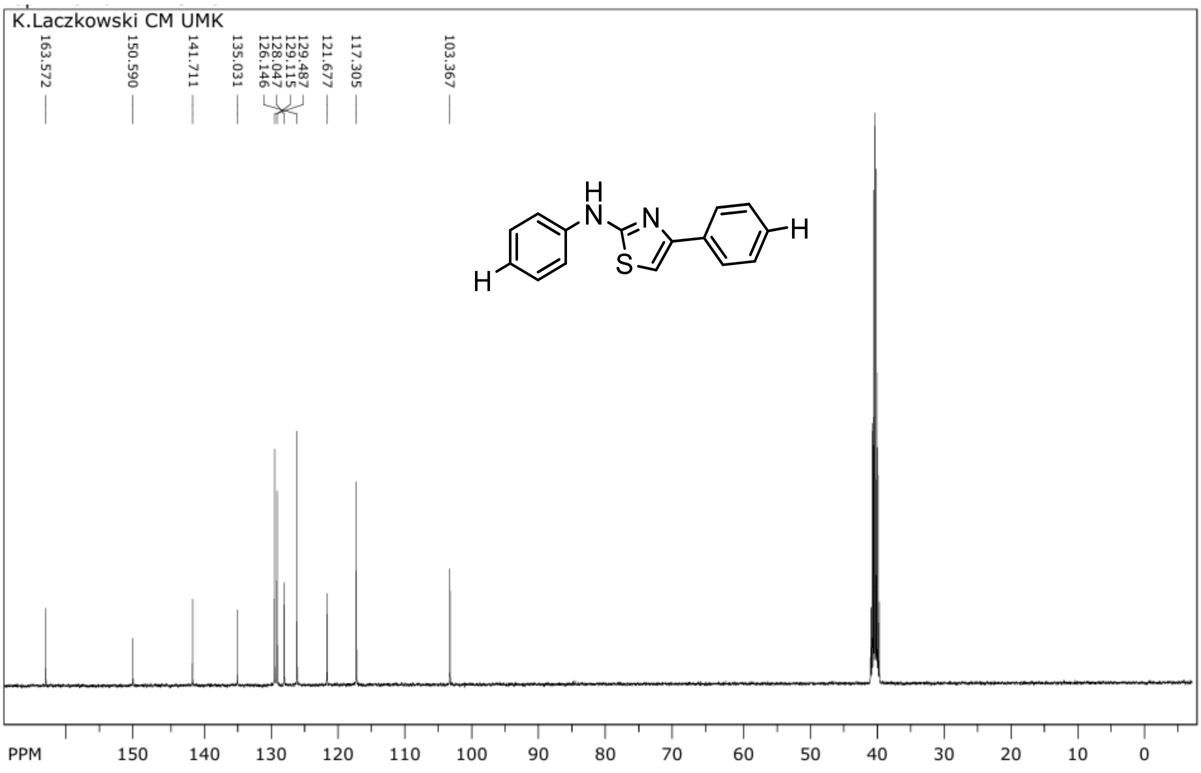
K.Laczkowski CM UMK
163.572
150.590
141.711
135.031
129.487
129.115
128.047
126.146
121.677
117.305
103.367
H
N
N
S
H
H
PPM
150
140
130
120
110
100
90
80
70
60
50
40
30
20
10
0

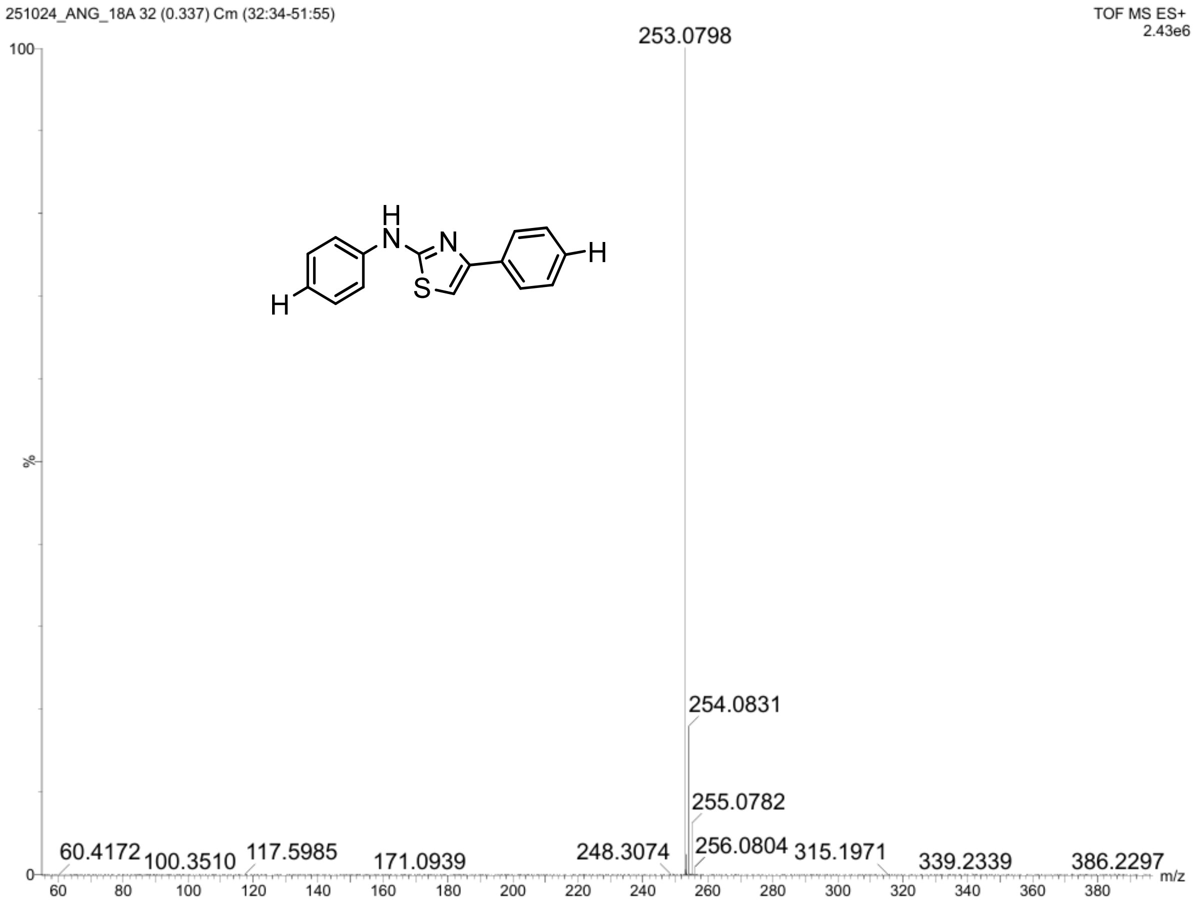

251024_ANG_18A 32 (0.337) Cm (32:34-51:55)
TOF MS ES+
2.43e6
253.0798
254.0831
255.0782
256.0804
248.3074
315.1971
339.2339
386.2297
171.0939
117.5985
100.3510
60.4172
%
m/z


**Compound 3b**

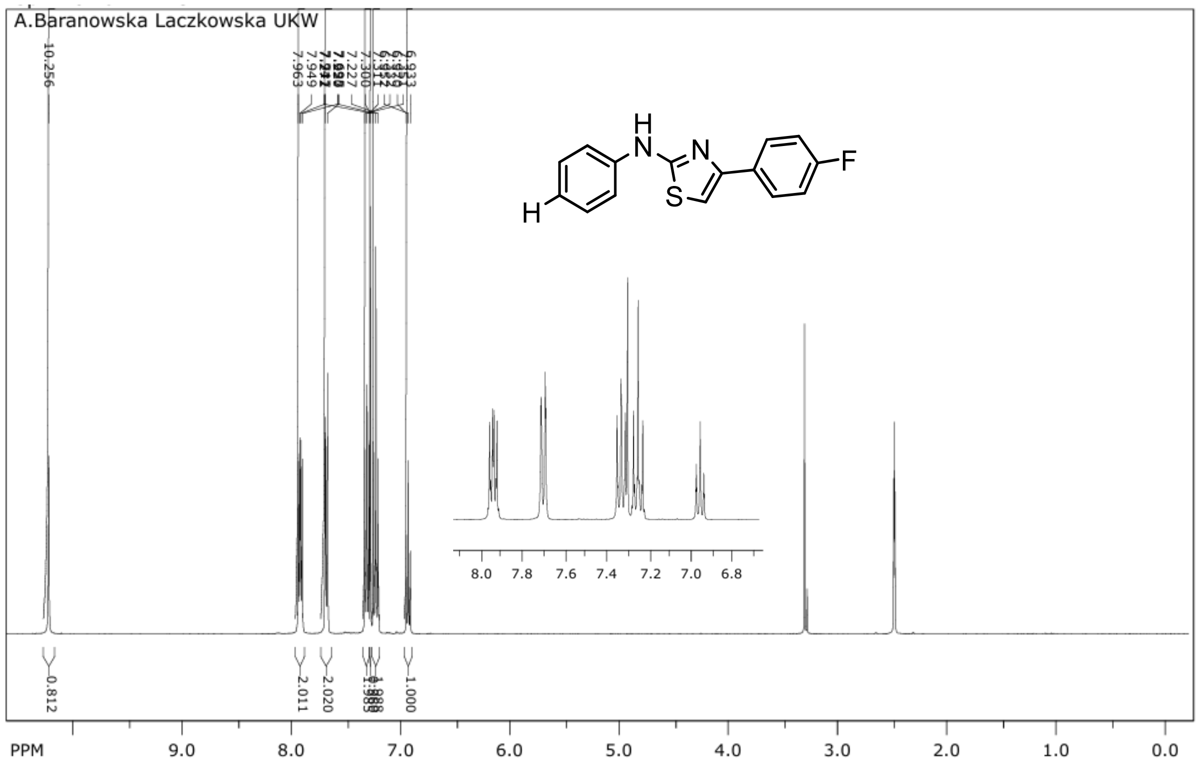

A.Baranowska Laczkowska UKW
10.256
0.812
2.011
2.020
1.000
PPM

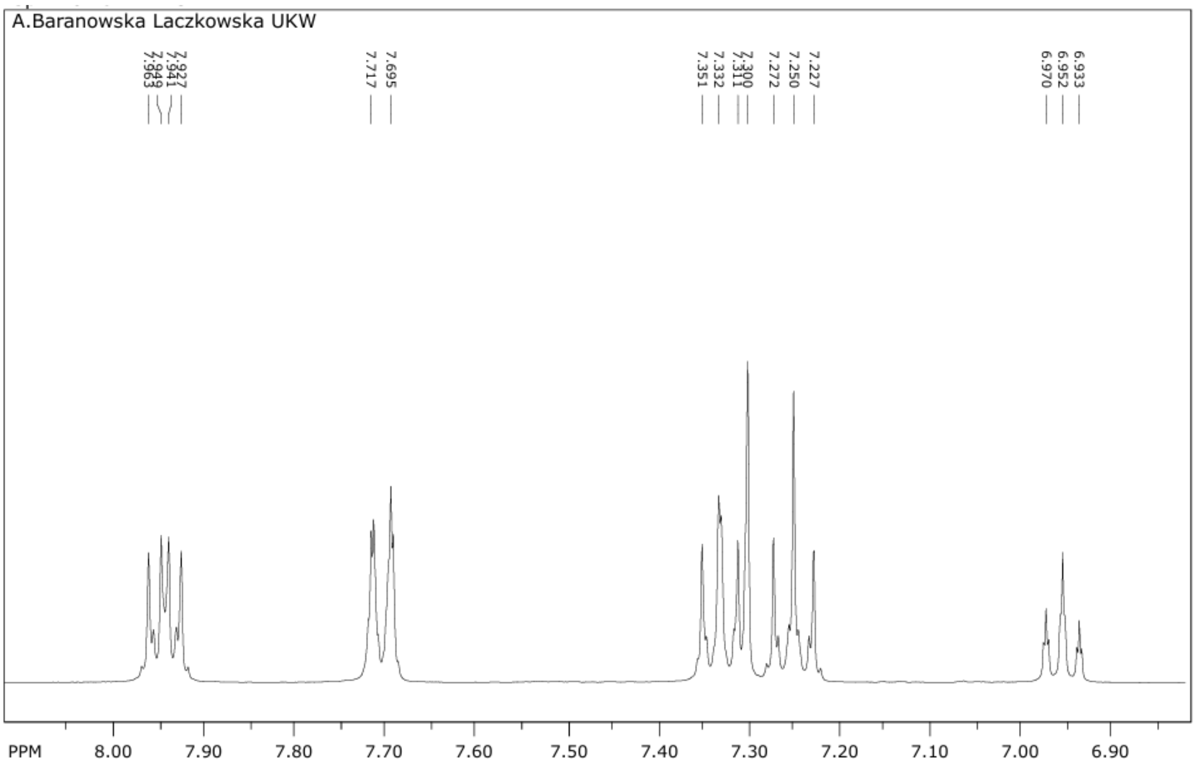
A.Baranowska Laczkowska UKW
7.963
7.949
7.941
7.927
7.717
7.695
7.351
7.332
7.311
7.300
7.272
7.250
7.227
6.970
6.952
6.933
PPM
8.00
7.90
7.80
7.70
7.60
7.50
7.40
7.30
7.20
7.10
7.00
6.90

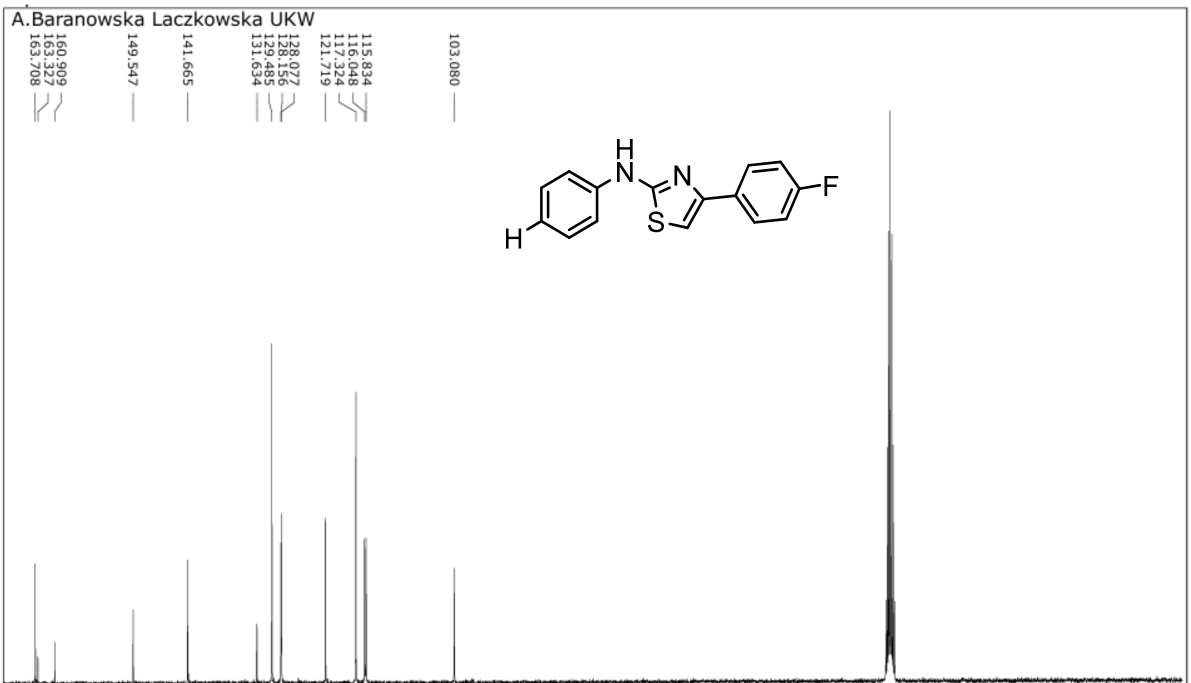
A.Baranowska Laczkowska UKW
163.708
163.327
160.909
149.547
141.665
131.634
129.485
128.156
128.077
121.719
117.324
116.048
115.834
103.080
H
N
N
S
F
H
PPM
150
140
130
120
110
100
90
80
70
60
50
40
30
20
10
0

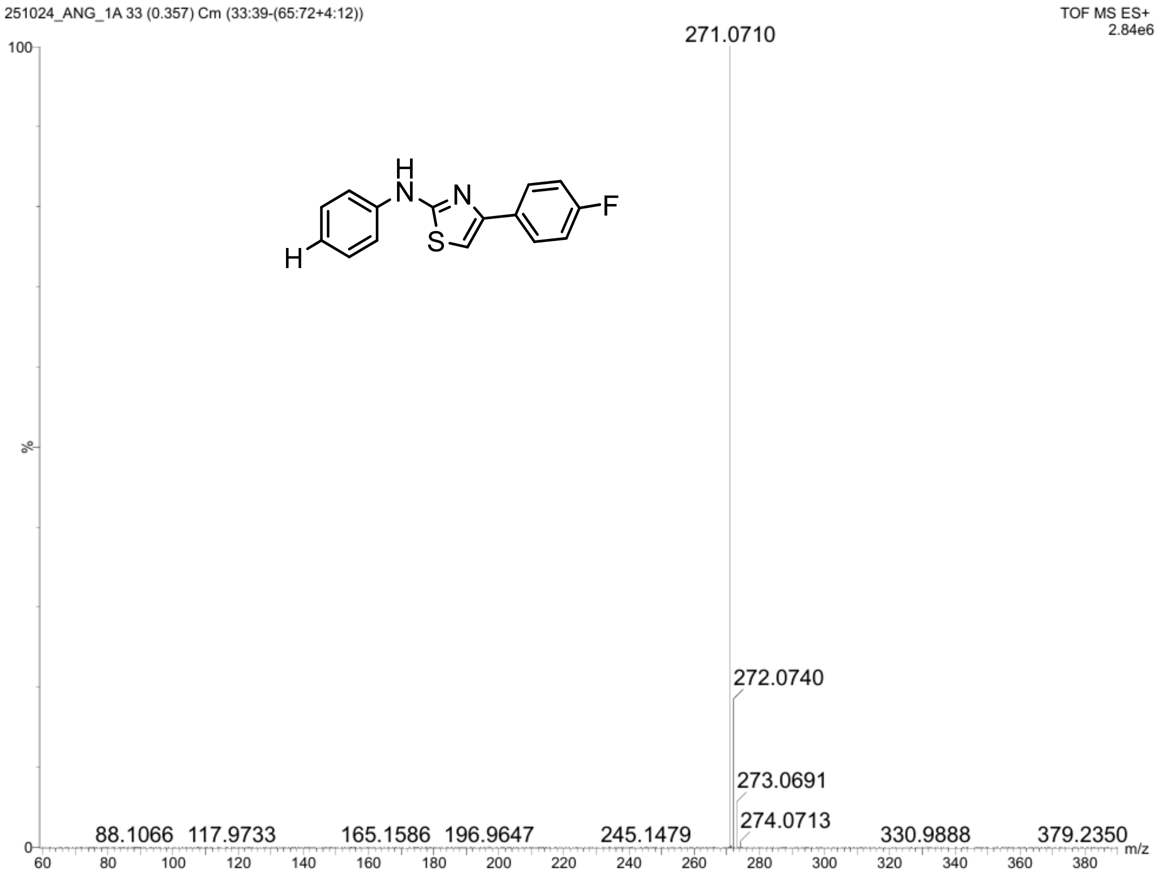
251024_ANG_1A 33 (0.357) Cm (33:39-(65:72+4:12))
TOF MS ES+
2.84e6
271.0710
272.0740
273.0691
274.0713
88.1066
117.9733
165.1586
196.9647
245.1479
330.9888
379.2350
%
m/z
S
N
H
F

**Compound 3c**

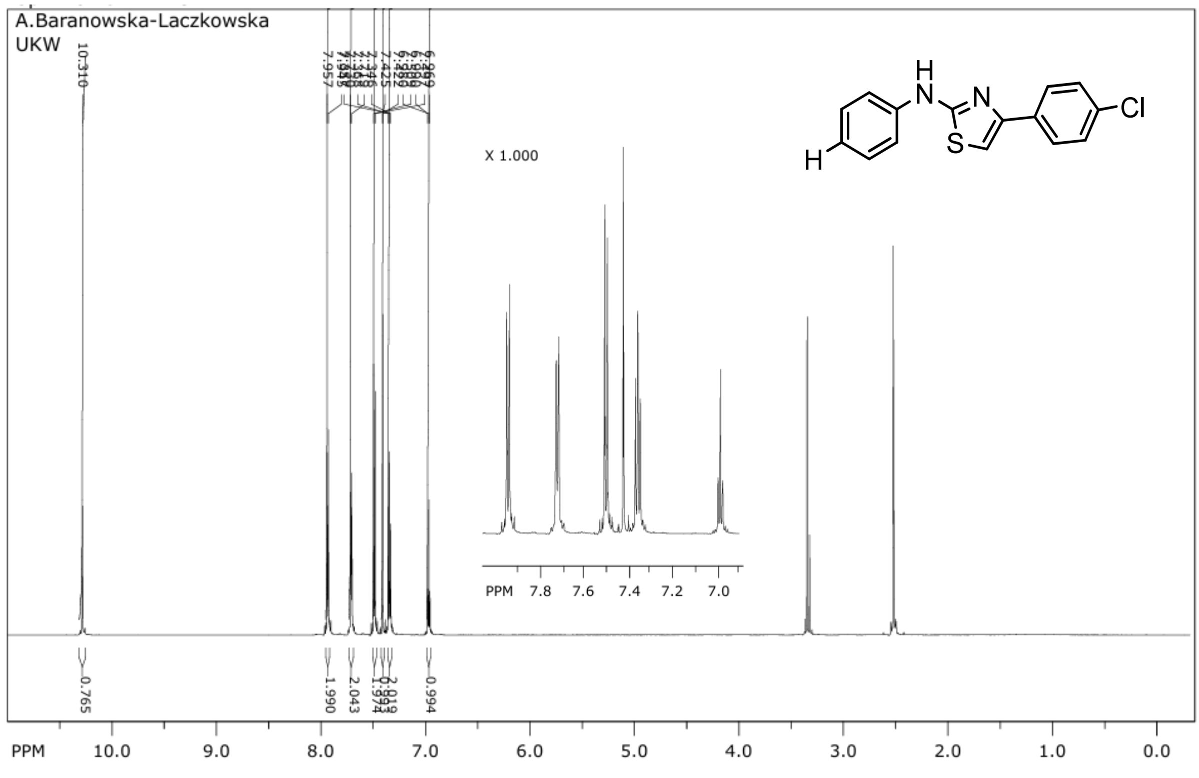
A.Baranowska-Laczkowska
UKW
10.310
X 1.000
PPM
0.765
1.990
2.043
0.994
Cl
S
N
H

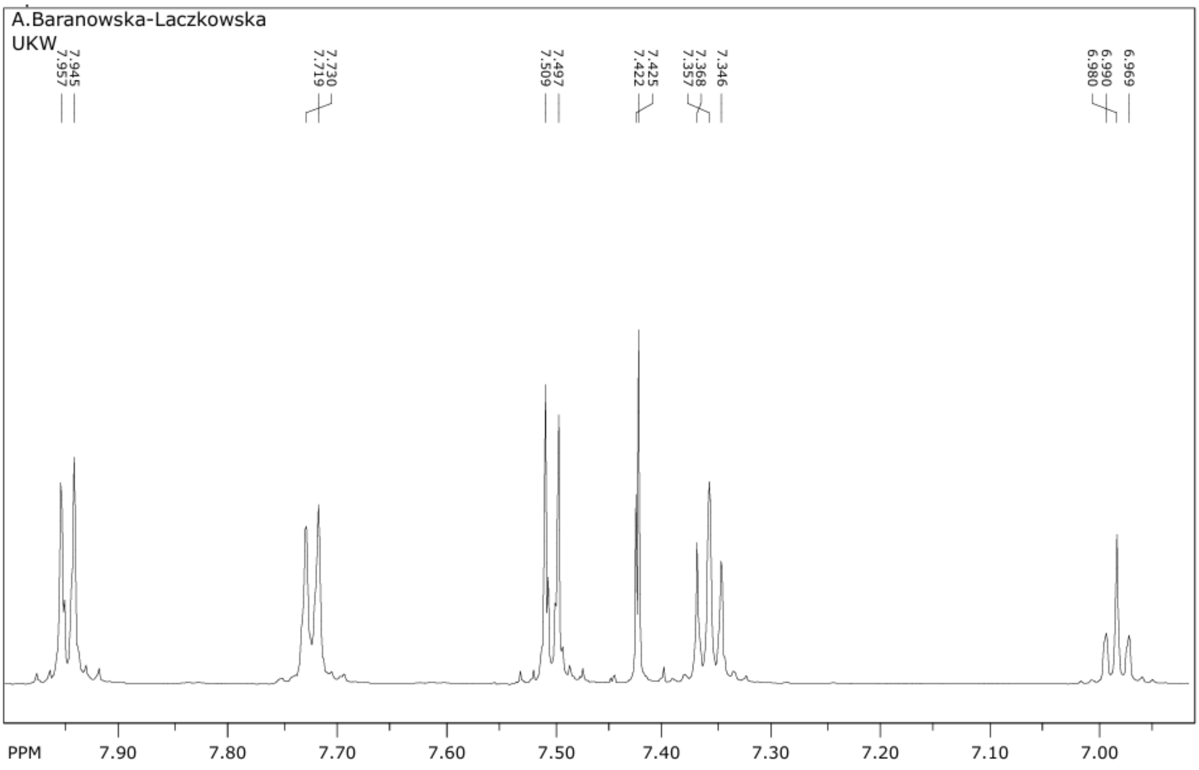
A.Baranowska-Laczkowska
UKW
7.957
7.945
7.730
7.719
7.509
7.497
7.425
7.422
7.368
7.357
7.346
6.990
6.980
6.969
PPM 7.90 7.80 7.70 7.60 7.50 7.40 7.30 7.20 7.10 7.00

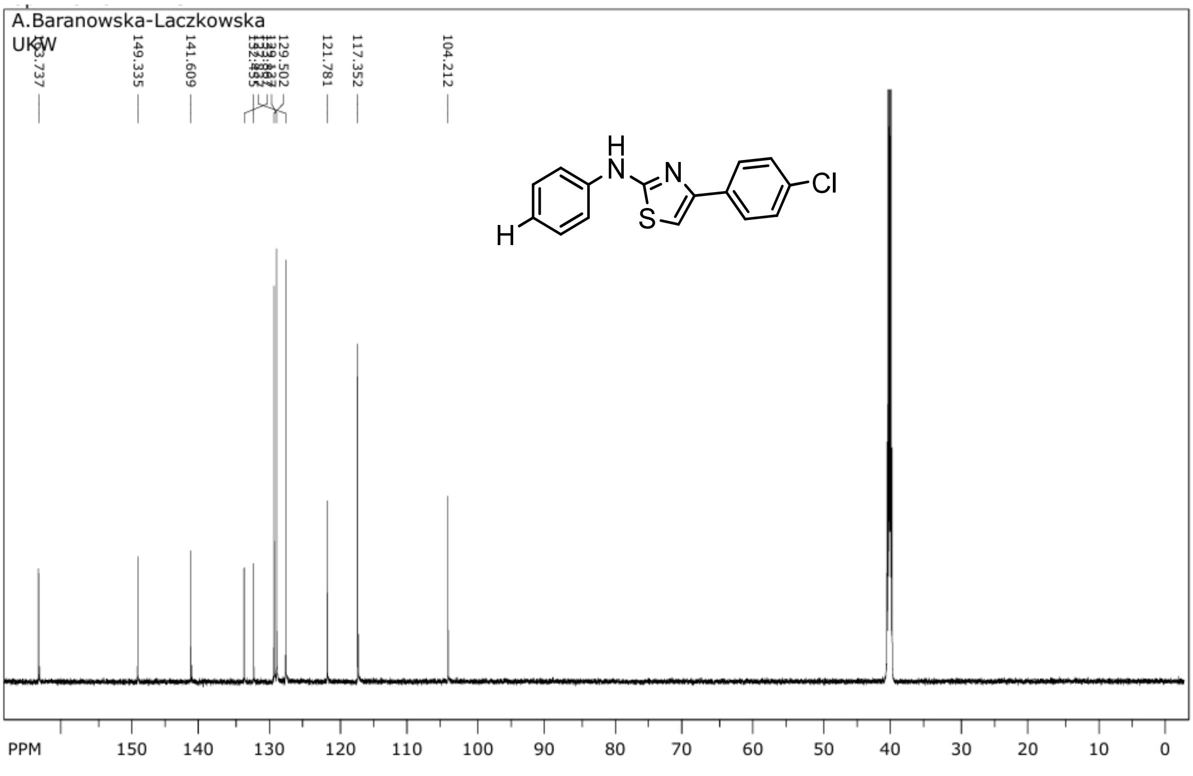
A.Baranowska-Laczkowska
UKW
149.335
141.609
129.502
121.781
117.352
104.212
H
N
N
S
Cl
H
PPM 150 140 130 120 110 100 90 80 70 60 50 40 30 20 10 0

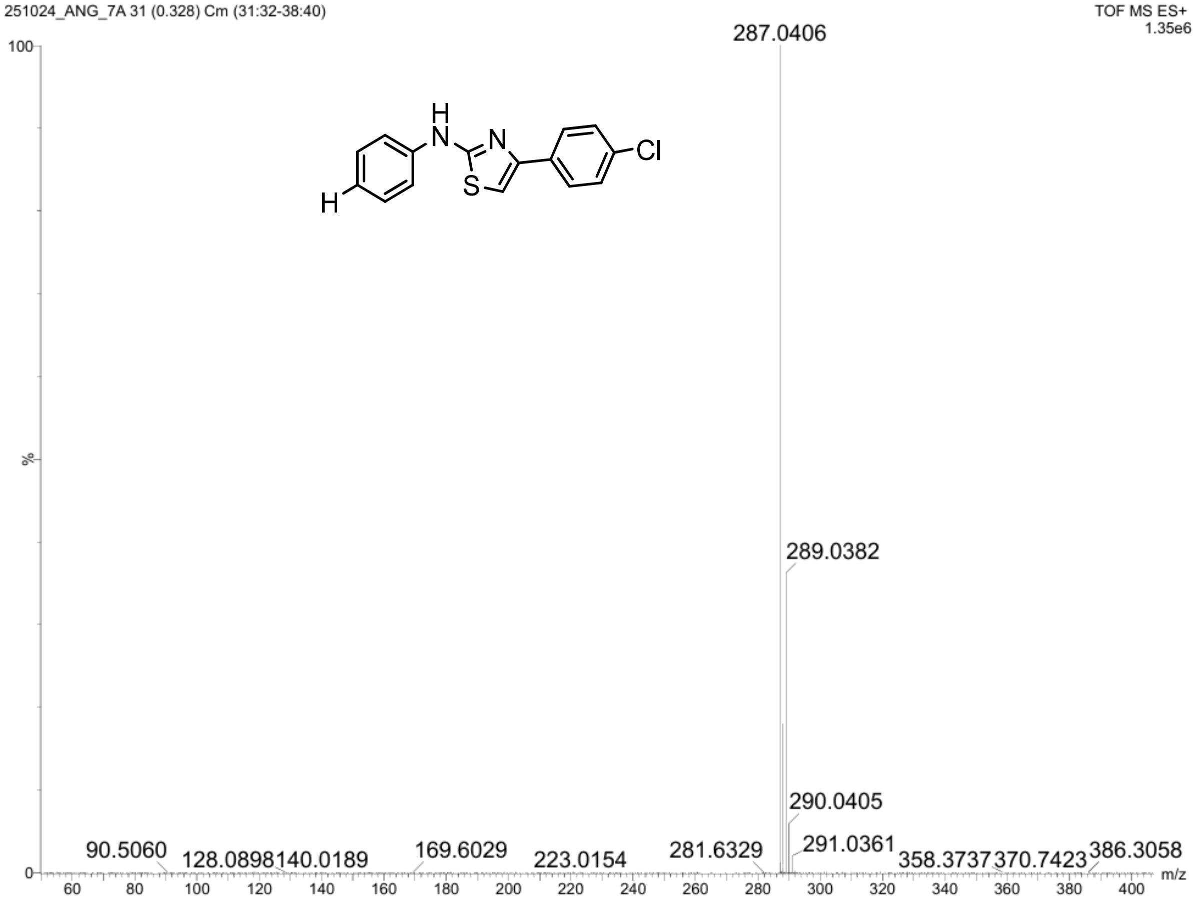
251024_ANG_7A 31 (0.328) Cm (31:32-38:40)
TOF MS ES+
1.35e6
287.0406
289.0382
290.0405
291.0361
90.5060
128.0898
140.0189
169.6029
223.0154
281.6329
358.3737
370.7423
386.3058
%
m/z
H
N
S
Cl

## Compound 3d

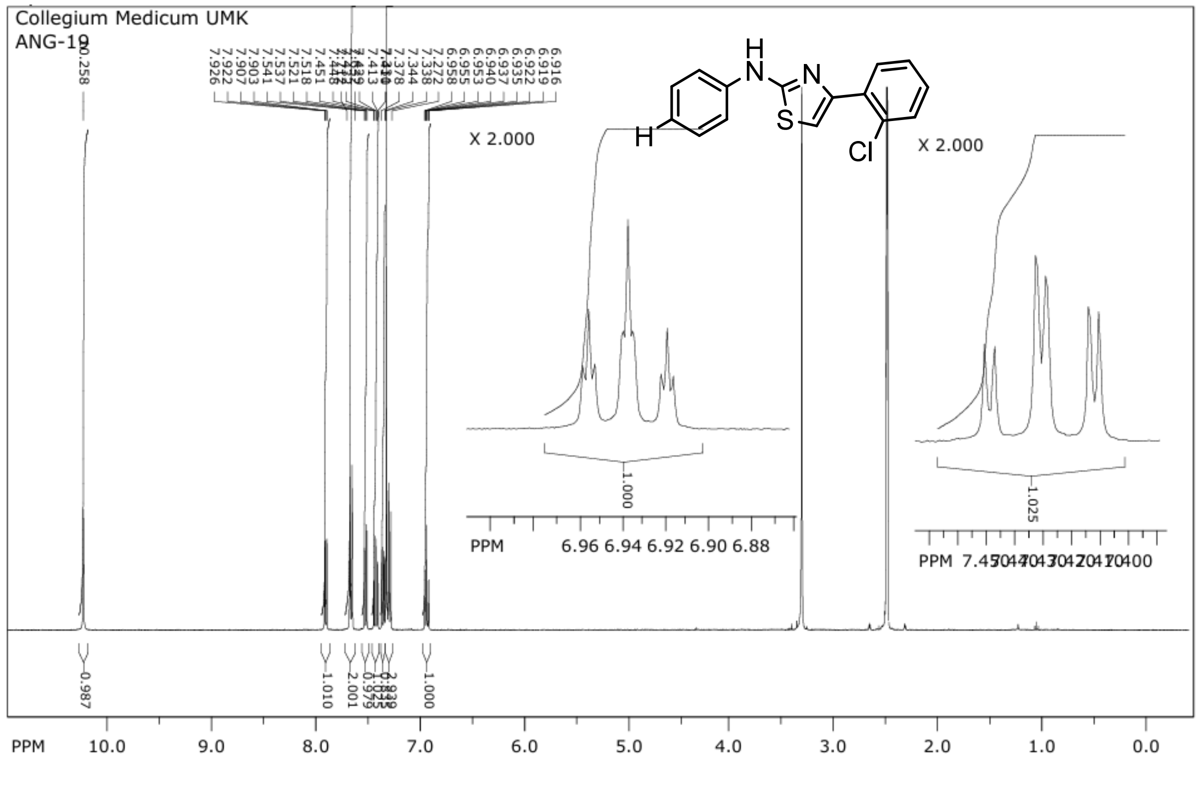
Collegium Medicum UMK
ANG-19
X 2.000
X 2.000
PPM
6.96 6.94 6.92 6.90 6.88
1.000
1.025
0.987
1.010
2.001
0.979
1.000
H
N
S
Cl

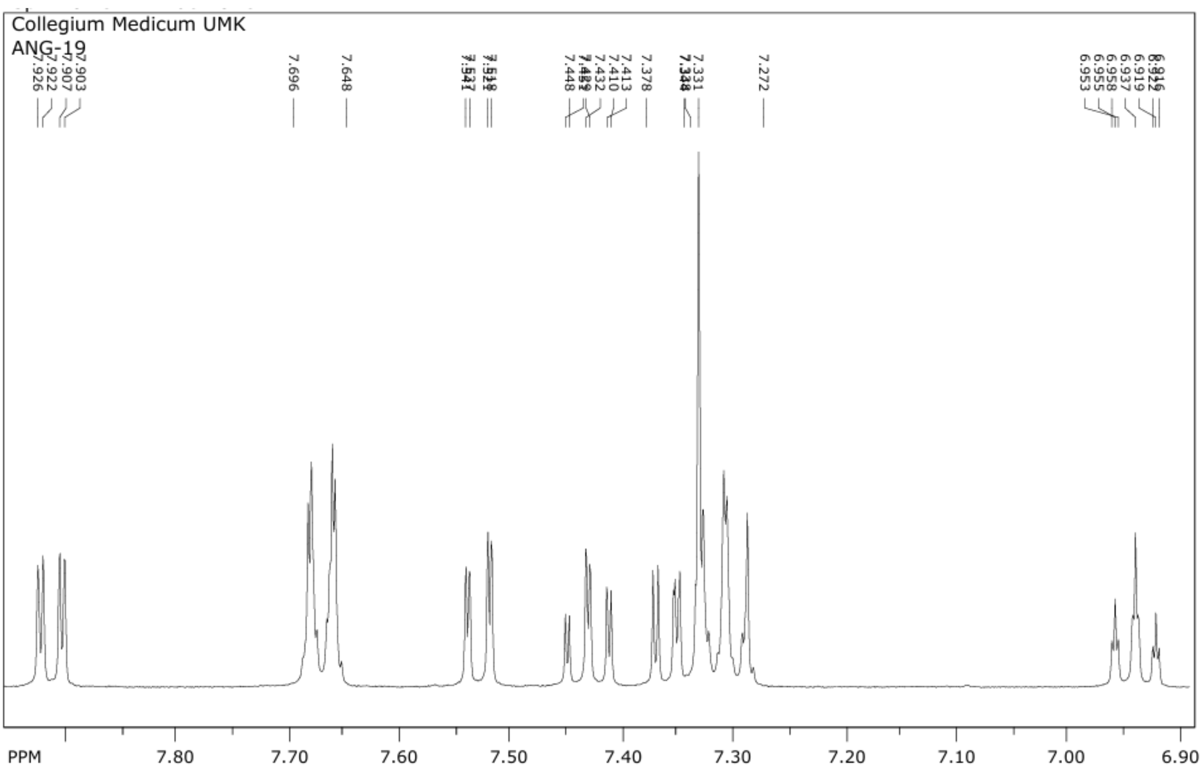
Collegium Medicum UMK
ANG-19
PPM
7.80
7.70
7.60
7.50
7.40
7.30
7.20
7.10
7.00
6.90

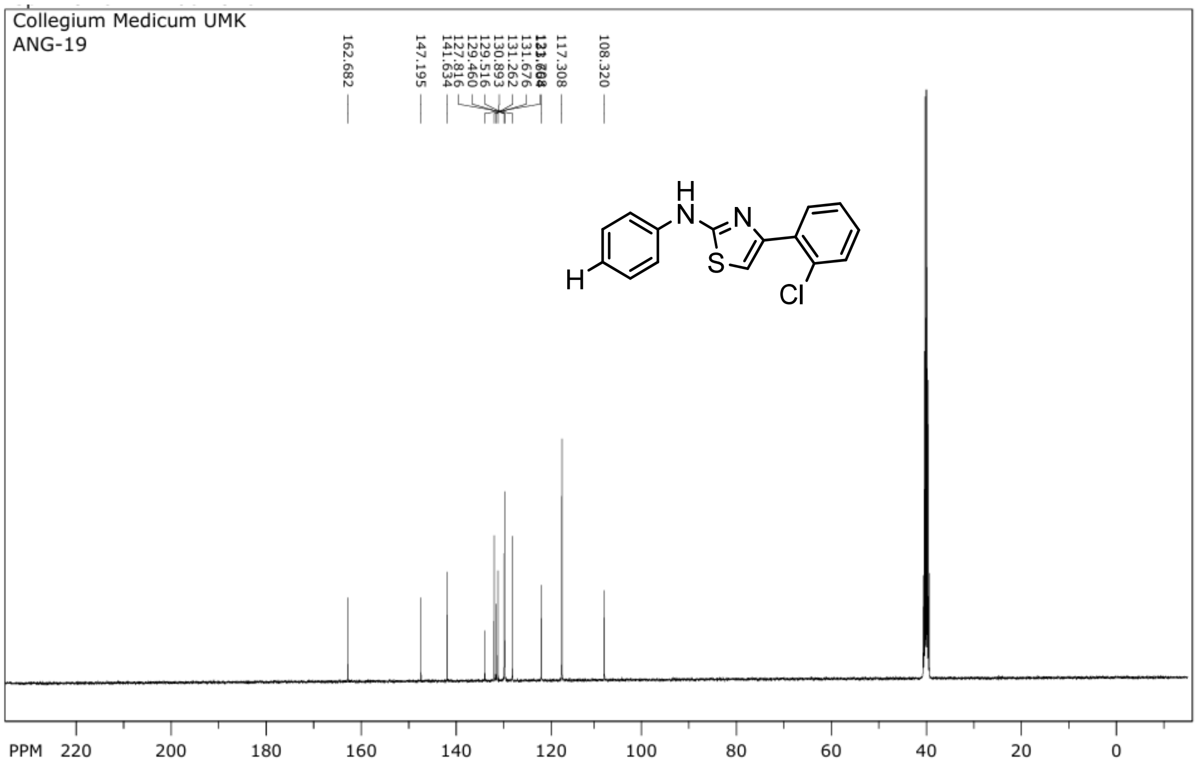
Collegium Medicum UMK
ANG-19
162.682
147.195
141.634
127.816
129.460
129.516
130.893
131.262
131.676
117.308
108.320
H
N
N
S
H
Cl
PPM
220
200
180
160
140
120
100
80
60
40
20
0

**Compound 3e**

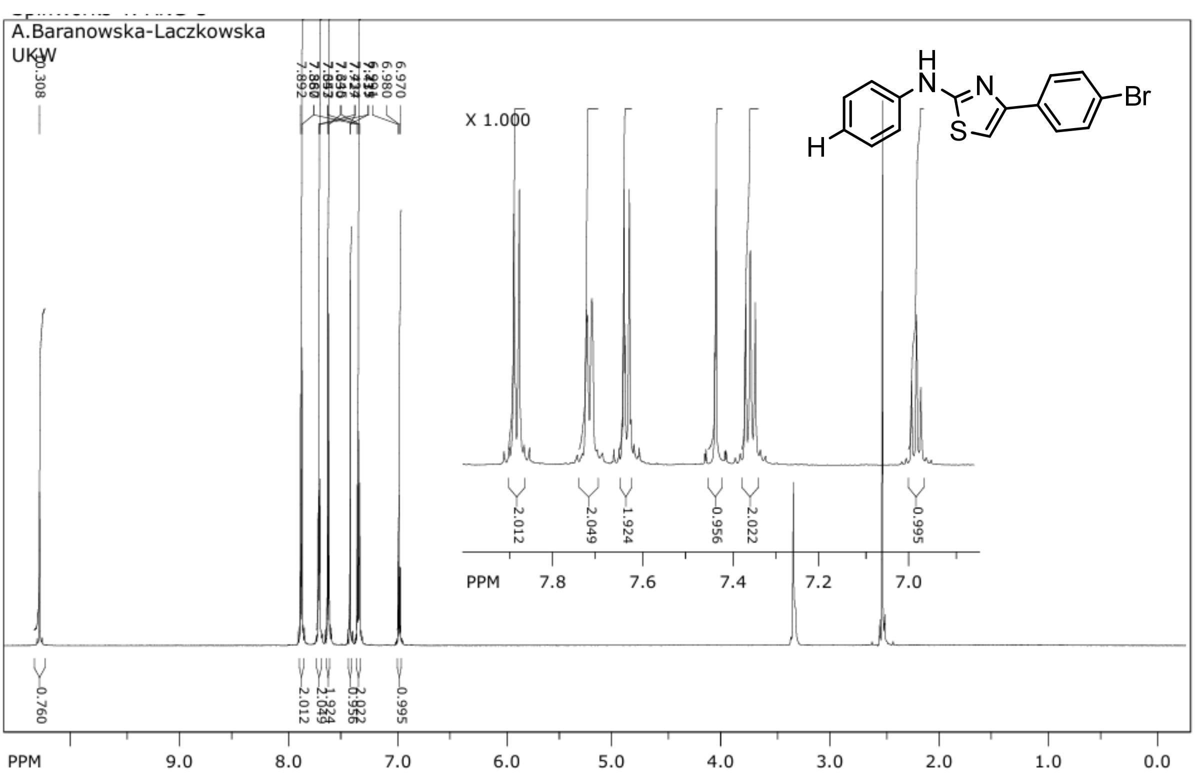
A.Baranowska-Laczkowska
UKW
10.308
7.892
6.980
6.970
X 1.000
H
N
N
S
Br
H
2.012
2.049
1.924
0.956
2.022
0.995
PPM 7.8 7.6 7.4 7.2 7.0
0.760
PPM 9.0 8.0 7.0 6.0 5.0 4.0 3.0 2.0 1.0 0.0

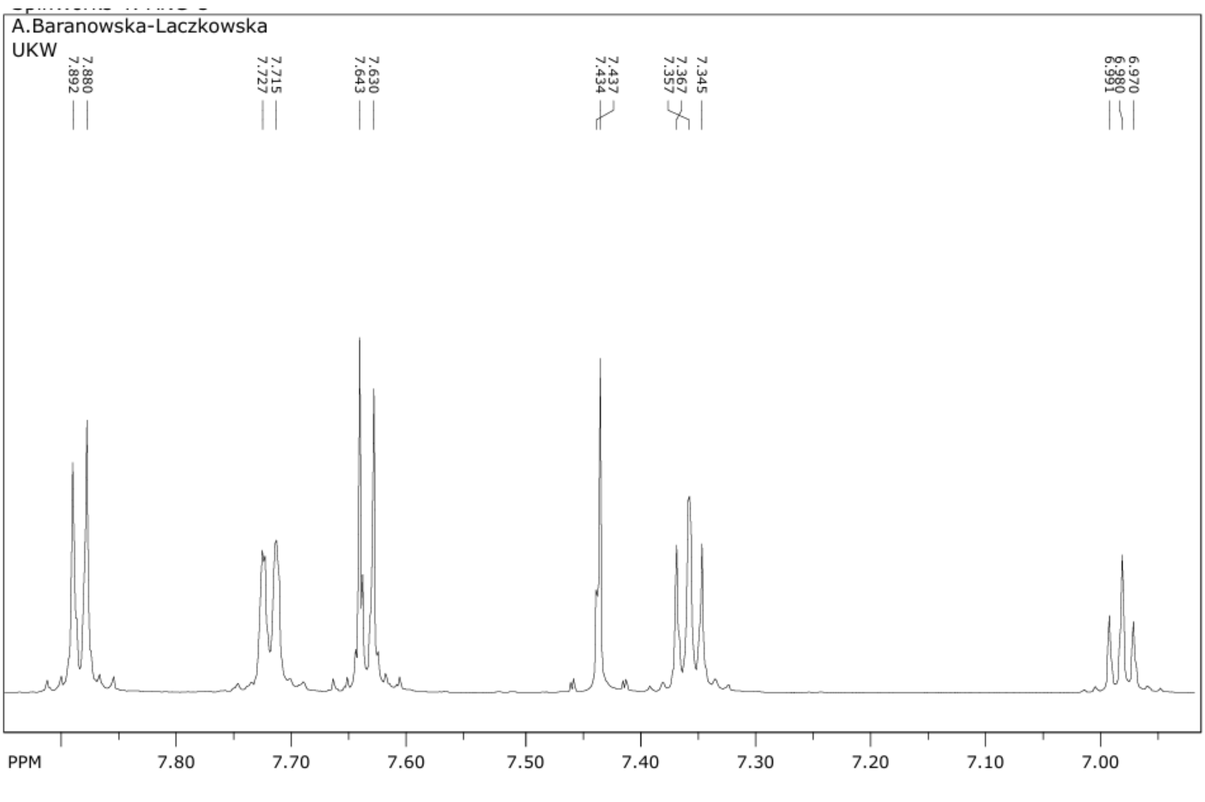
A.Baranowska-Laczkowska
UKW
7.892
7.880
7.727
7.715
7.643
7.630
7.437
7.434
7.367
7.357
7.345
6.991
6.980
6.970
PPM 7.80 7.70 7.60 7.50 7.40 7.30 7.20 7.10 7.00

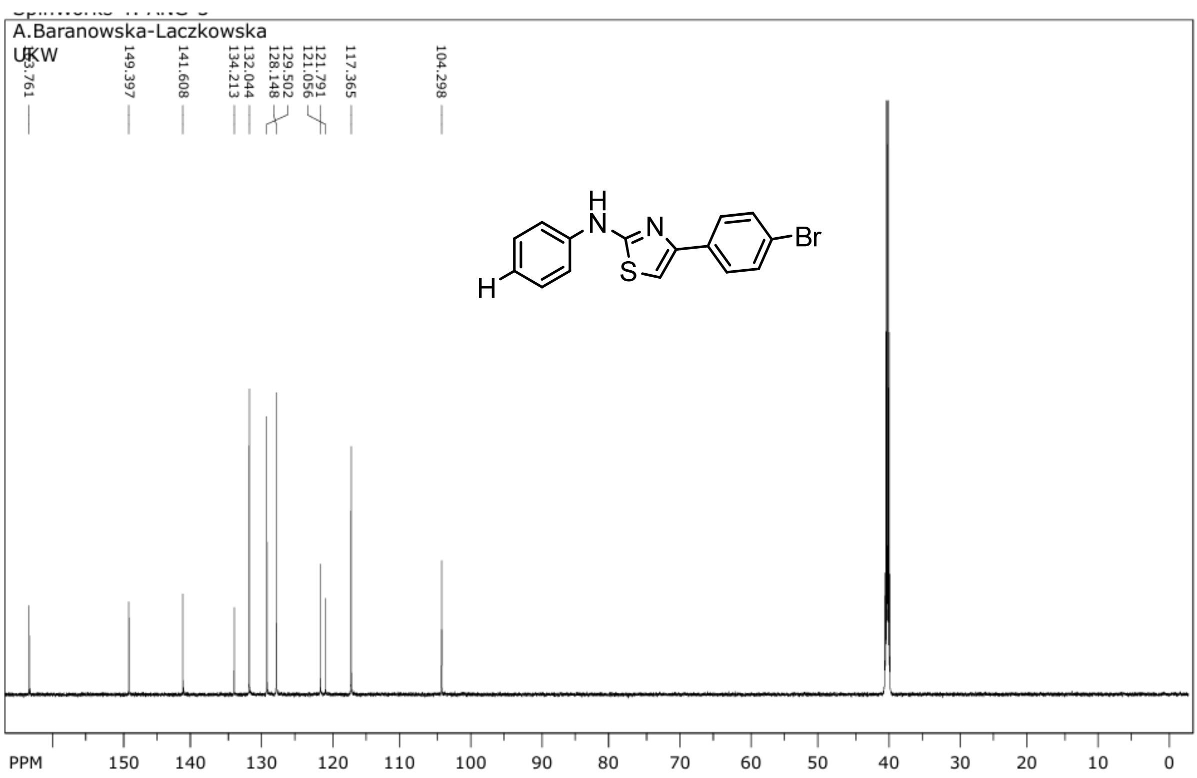
A.Baranowska-Laczkowska
UKW
149.397
141.608
134.213
132.044
129.502
128.148
121.791
121.056
117.365
104.298
Br
H
N
S
PPM
150 140 130 120 110 100 90 80 70 60 50 40 30 20 10 0

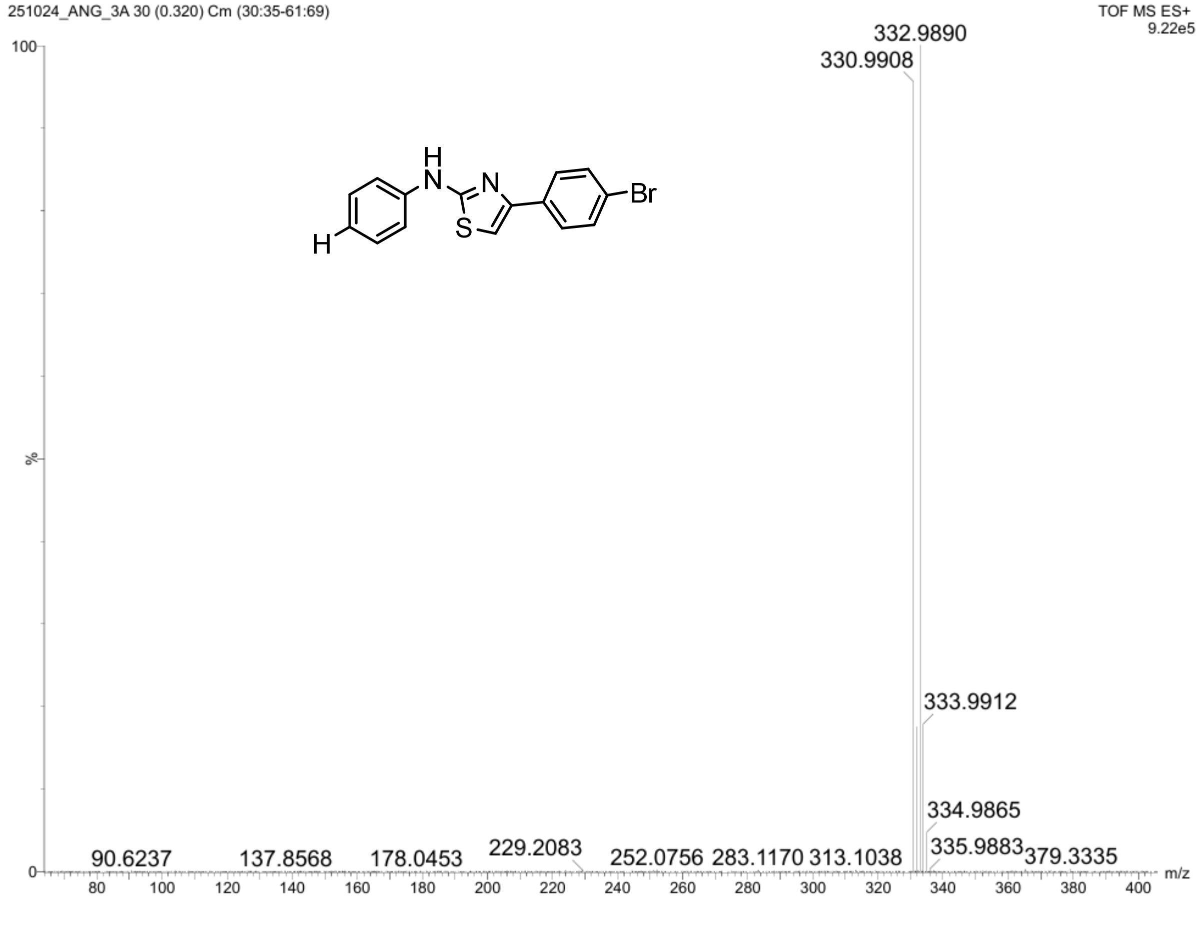
251024_ANG_3A 30 (0.320) Cm (30:35-61:69)
TOF MS ES+
9.22e5
100
%
0
332.9890
330.9908
333.9912
334.9865
335.9883
379.3335
90.6237
137.8568
178.0453
229.2083
252.0756
283.1170
313.1038
Br
H
N
S
m/z
80 100 120 140 160 180 200 220 240 260 280 300 320 340 360 380 400

**Compound 3f**

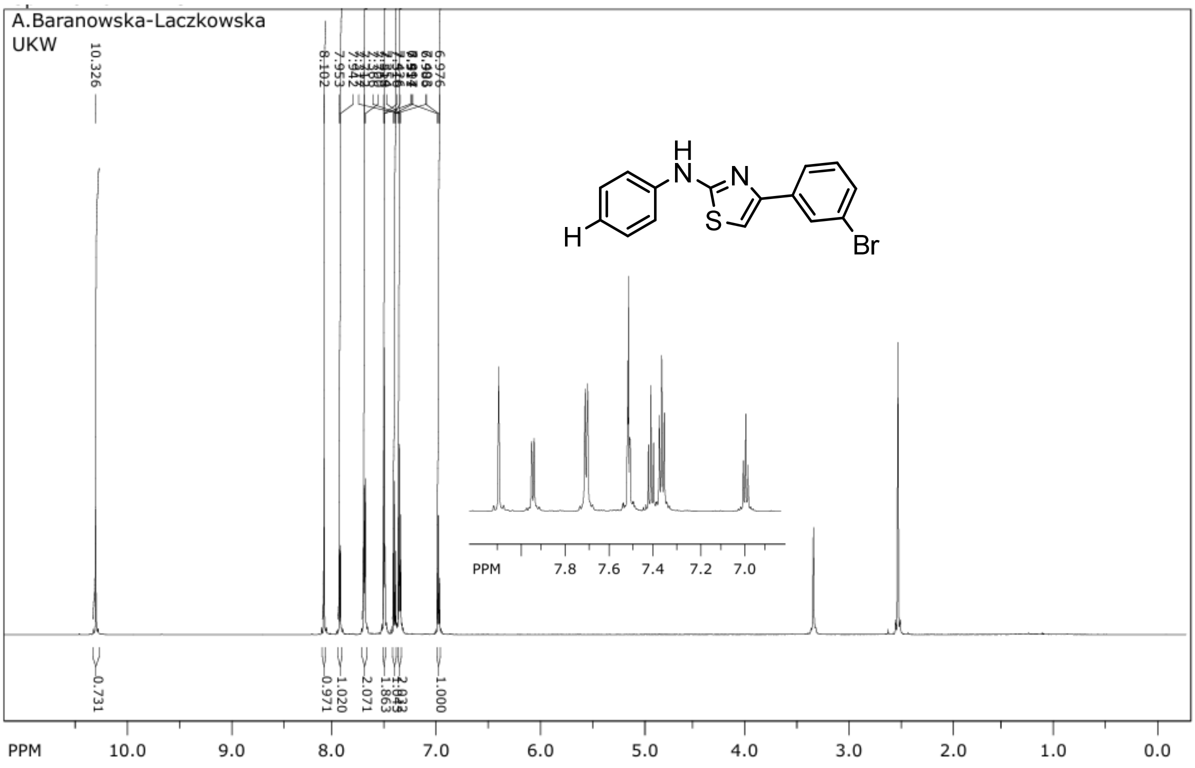
A.Baranowska-Laczkowska
UKW
10.326
8.102
7.953
6.976
H
N
N
S
H
Br
PPM
7.8 7.6 7.4 7.2 7.0
0.731
1.020
0.971
2.071
1.863
1.000
PPM
10.0 9.0 8.0 7.0 6.0 5.0 4.0 3.0 2.0 1.0 0.0

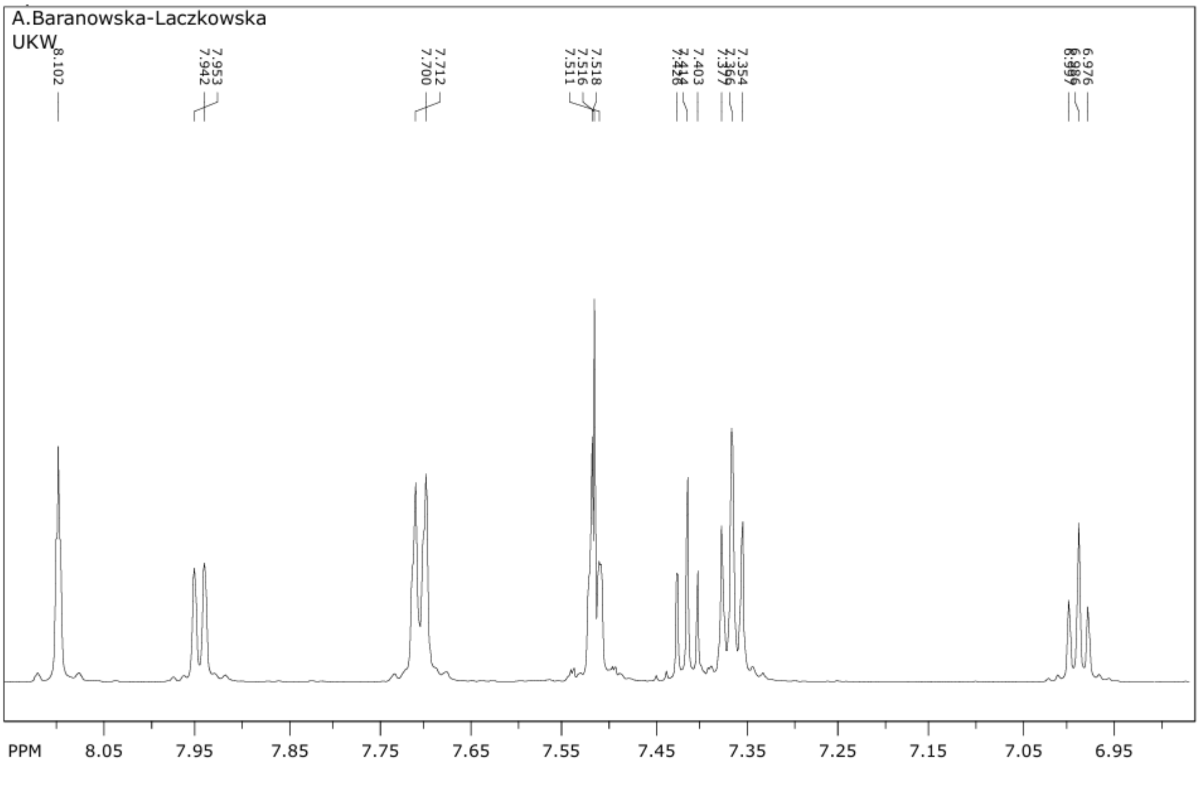
A.Baranowska-Laczkowska
UKW
8.102
7.953
7.942
7.712
7.700
7.518
7.516
7.511
7.403
7.354
6.976
PPM
8.05 7.95 7.85 7.75 7.65 7.55 7.45 7.35 7.25 7.15 7.05 6.95

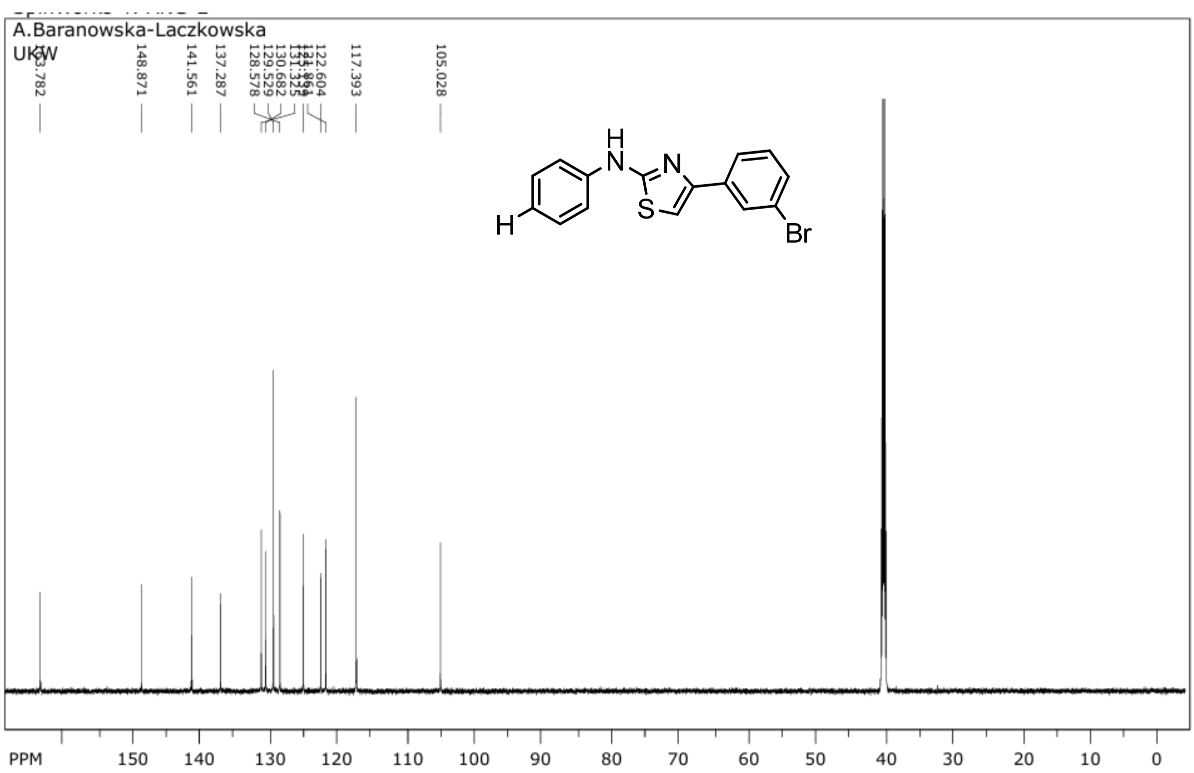
A.Baranowska-Laczkowska
UKW
148.871
141.561
137.287
128.578
129.529
130.682
131.325
122.604
117.393
105.028
H
N
N
S
H
Br
PPM
150
140
130
120
110
100
90
80
70
60
50
40
30
20
10
0

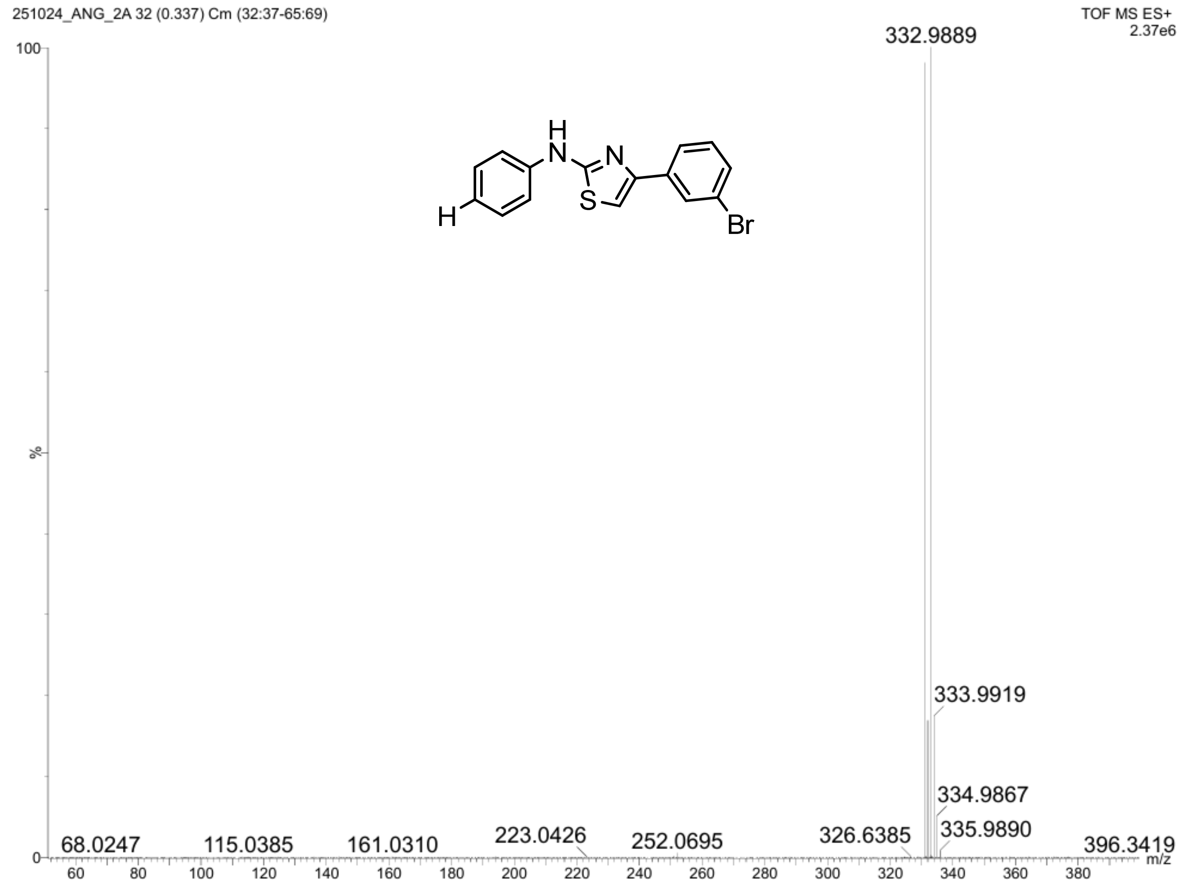
251024_ANG_2A 32 (0.337) Cm (32:37-65:69)
TOF MS ES+
2.37e6
332.9889
100
%
0
H
N
N
S
H
Br
333.9919
334.9867
335.9890
68.0247
115.0385
161.0310
223.0426
252.0695
326.6385
396.3419
m/z
60
80
100
120
140
160
180
200
220
240
260
280
300
320
340
360
380

**Compound 3g**

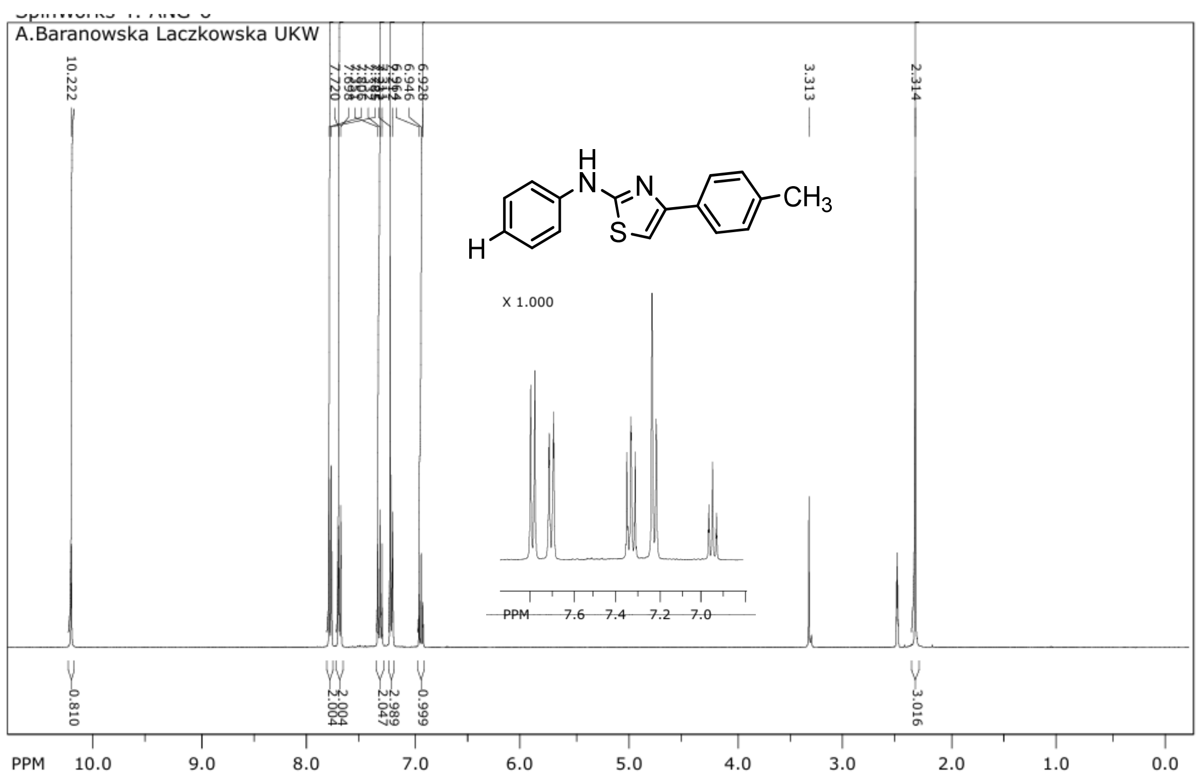
A.Baranowska Laczkowska UKW
10.222
7.720
6.946
6.928
3.313
2.314
X 1.000
0.810
2.004
2.004
2.047
2.989
0.999
3.016
PPM 10.0 9.0 8.0 7.0 6.0 5.0 4.0 3.0 2.0 1.0 0.0

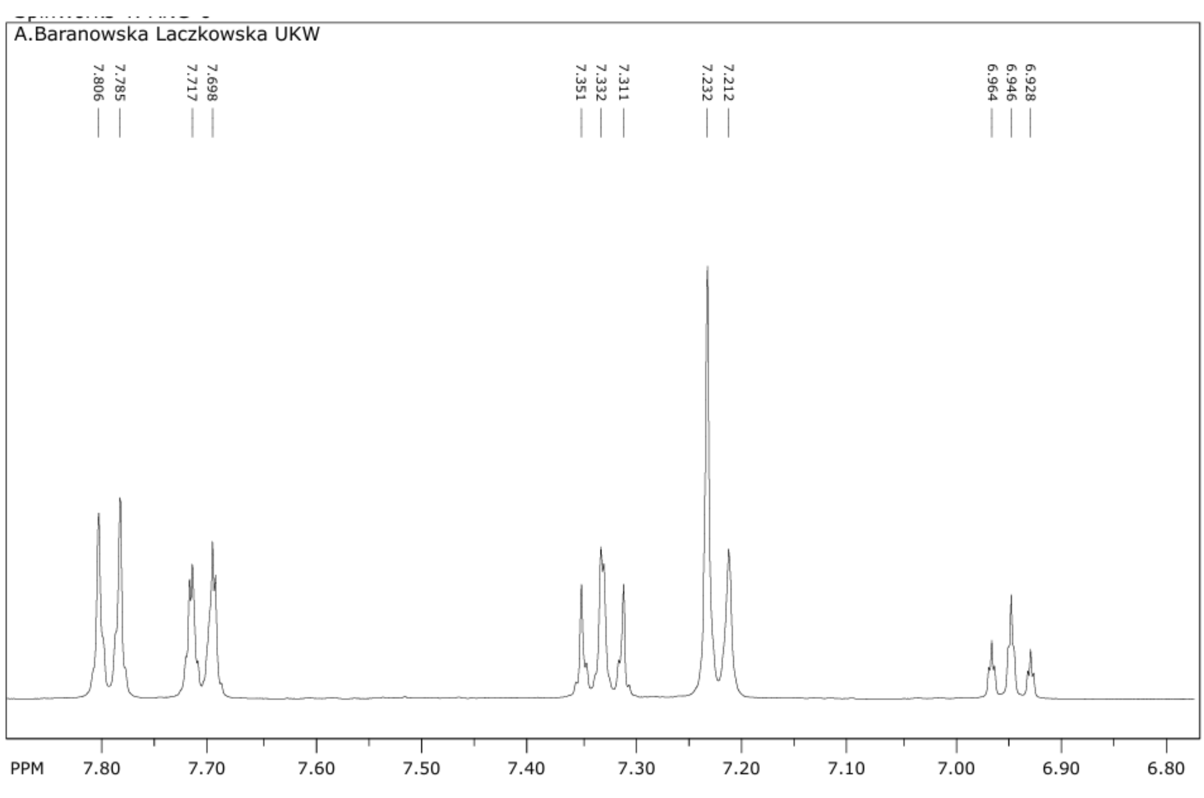
A.Baranowska Laczkowska UKW
7.806
7.785
7.717
7.698
7.351
7.332
7.311
7.232
7.212
6.964
6.946
6.928
PPM 7.80 7.70 7.60 7.50 7.40 7.30 7.20 7.10 7.00 6.90 6.80

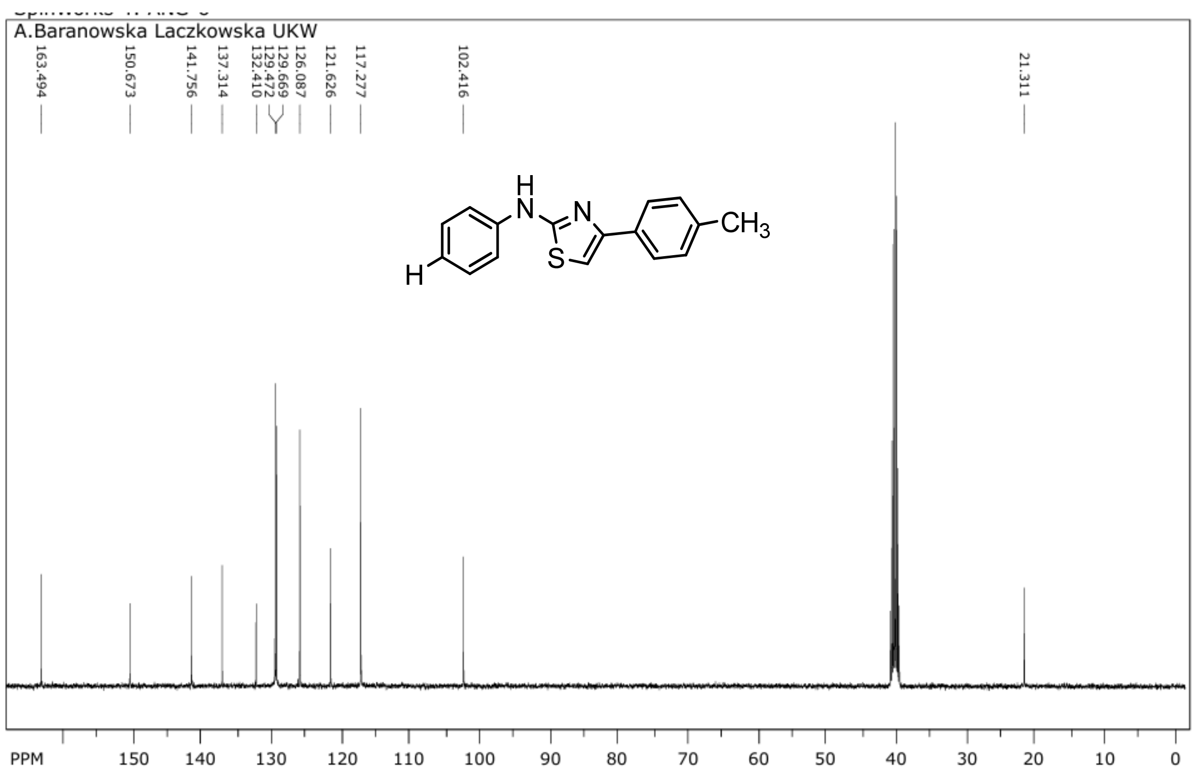
A.Baranowska Laczkowska UKW
163.494
150.673
141.756
137.314
132.410
129.669
129.472
126.087
121.626
117.277
102.416
21.311
PPM
150
140
130
120
110
100
90
80
70
60
50
40
30
20
10
0

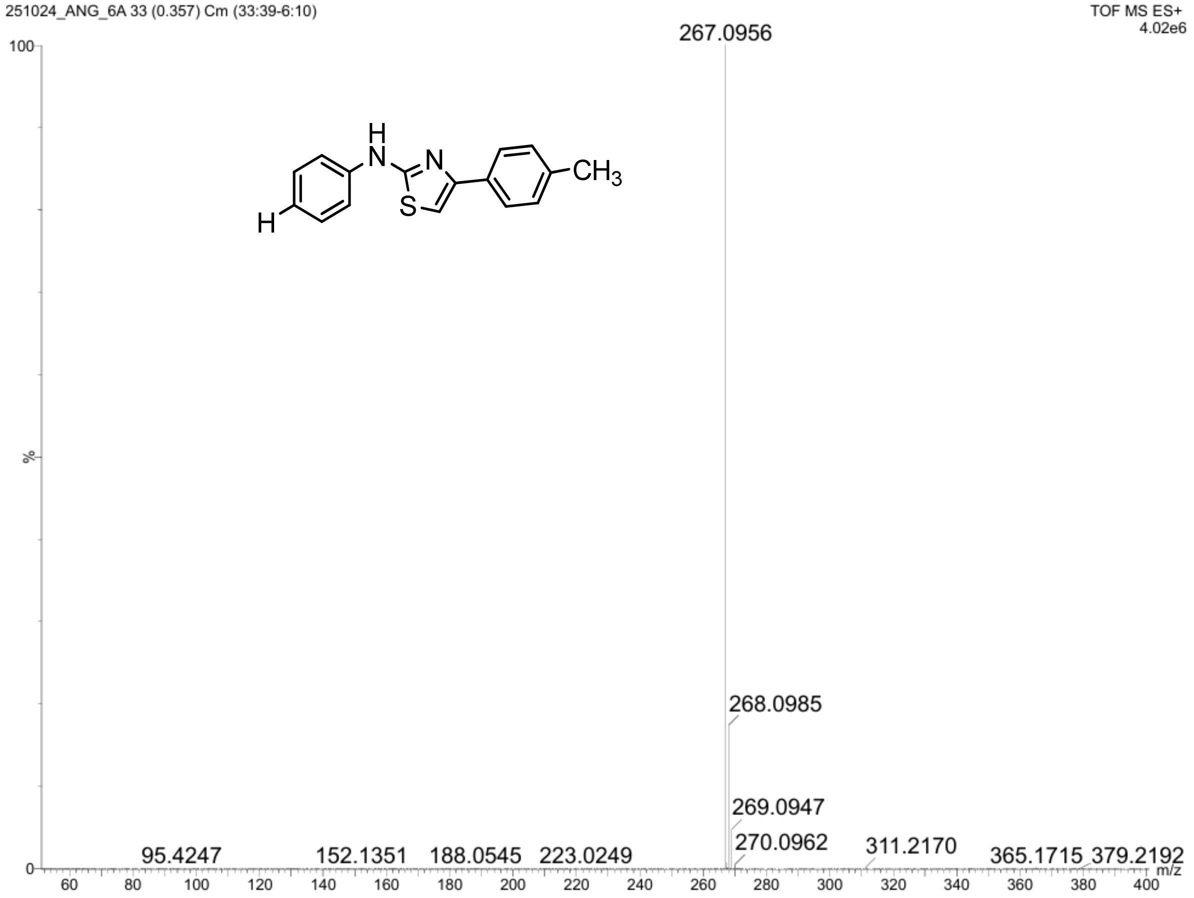
251024_ANG_6A 33 (0.357) Cm (33:39-6:10)
TOF MS ES+
4.02e6
267.0956
268.0985
269.0947
270.0962
311.2170
365.1715
379.2192
95.4247
152.1351
188.0545
223.0249
100
%
0
m/z

**Compound 3h**

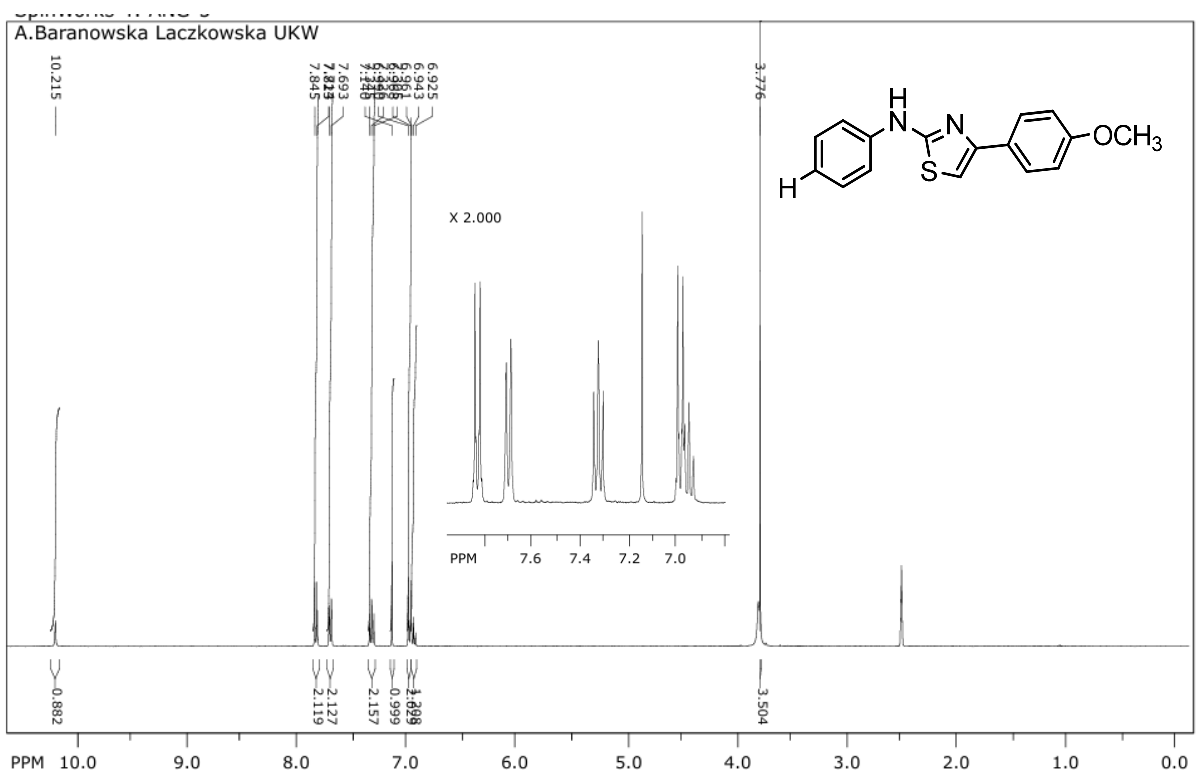
A.Baranowska Laczkowska UKW
10.215
7.845
7.693
6.943
6.925
3.776
X 2.000
0.882
2.119
2.127
2.157
0.999
3.504
PPM 10.0 9.0 8.0 7.0 6.0 5.0 4.0 3.0 2.0 1.0 0.0
PPM 7.6 7.4 7.2 7.0
OCH3

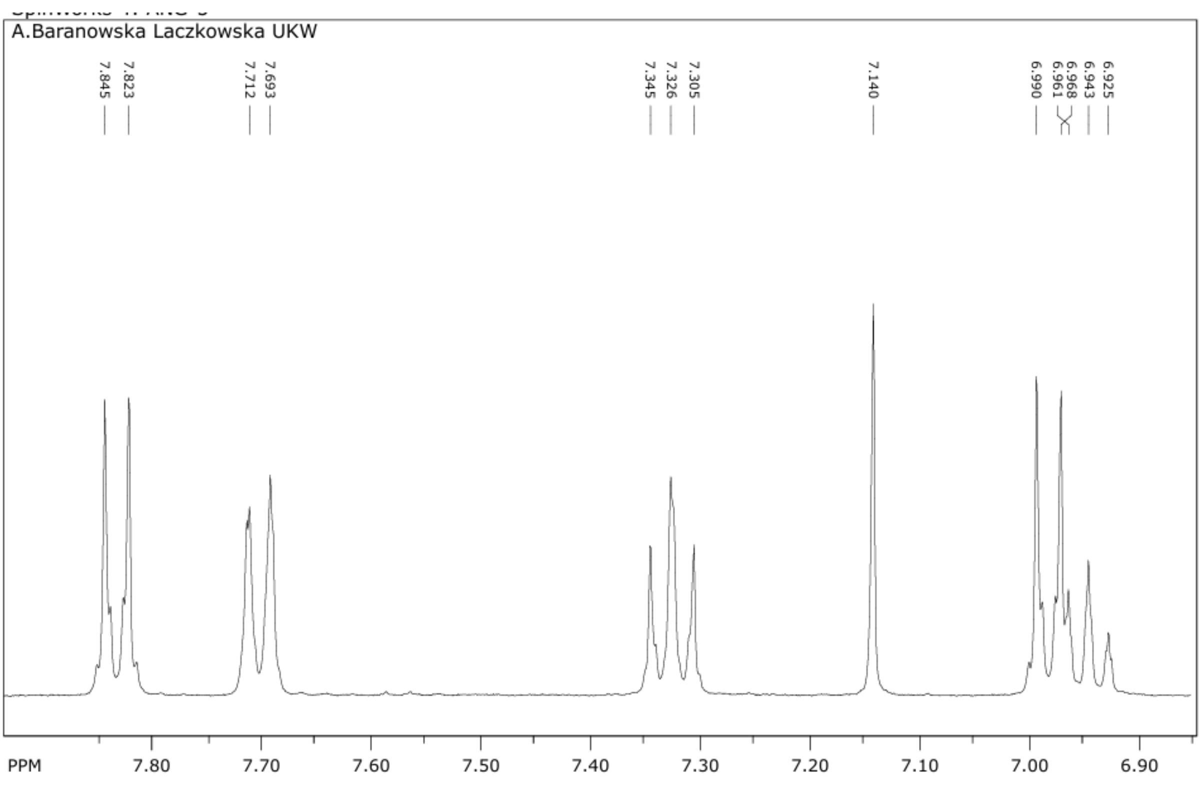
A.Baranowska Laczkowska UKW
7.845
7.823
7.712
7.693
7.345
7.326
7.305
7.140
6.990
6.968
6.961
6.943
6.925
PPM 7.80 7.70 7.60 7.50 7.40 7.30 7.20 7.10 7.00 6.90

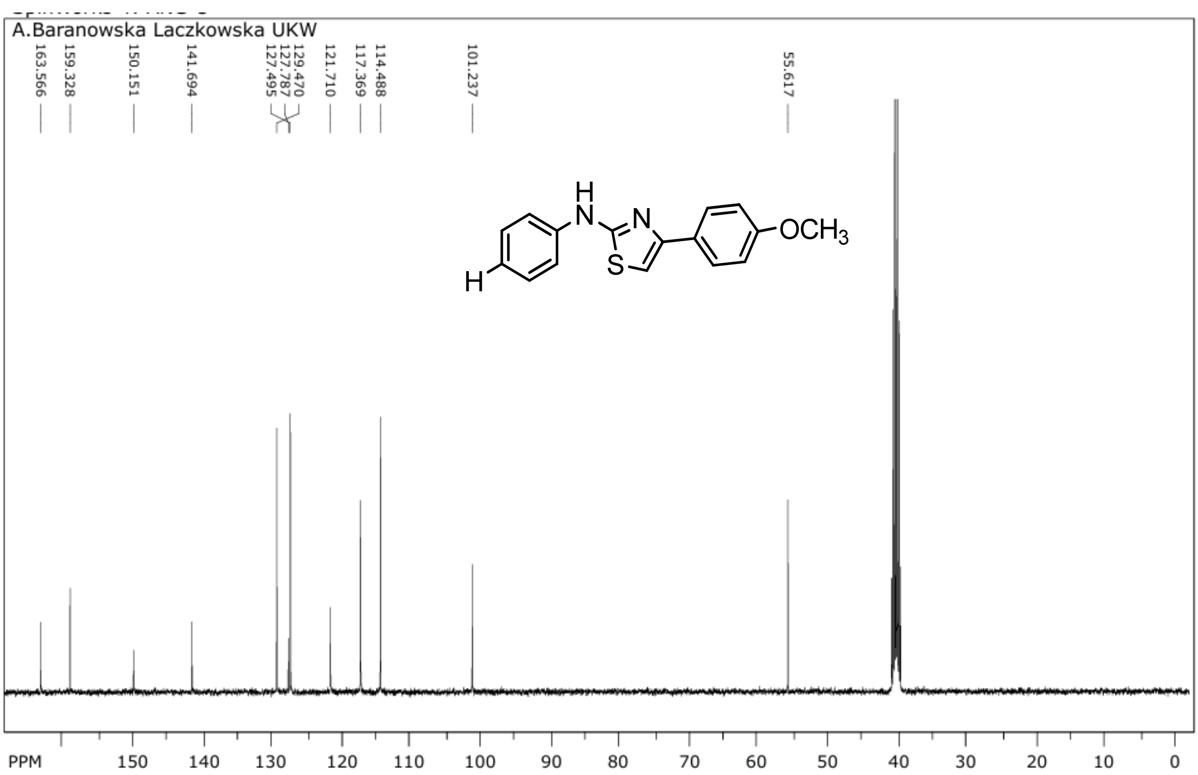
A.Baranowska Laczkowska UKW
163.566
159.328
150.151
141.694
129.470
127.787
127.495
121.710
117.369
114.488
101.237
55.617
H
N
N
S
OCH3
H
PPM
150 140 130 120 110 100 90 80 70 60 50 40 30 20 10 0

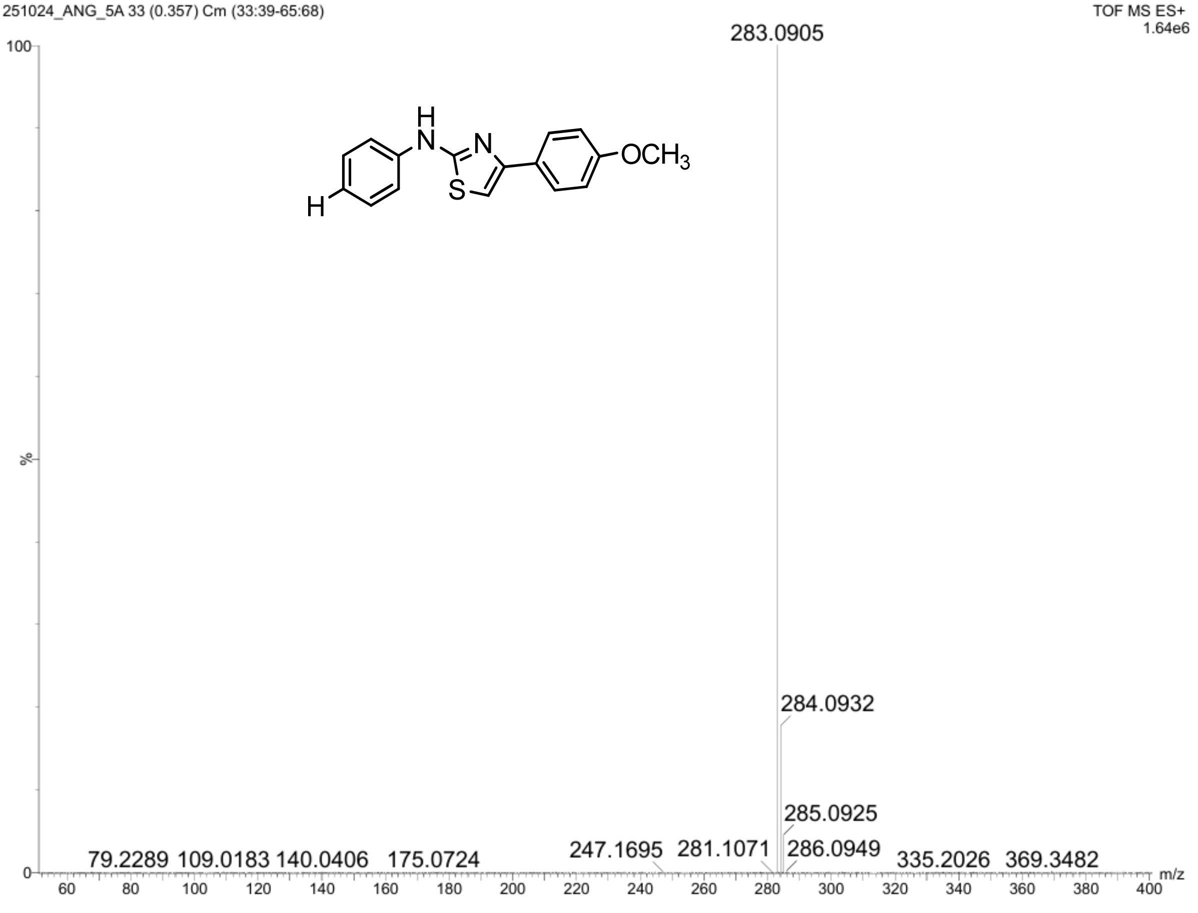
251024_ANG_5A 33 (0.357) Cm (33:39-65:68)
TOF MS ES+
1.64e6
283.0905
100
%
0
H
N
N
S
OCH3
H
284.0932
285.0925
79.2289 109.0183 140.0406 175.0724 247.1695 281.1071 286.0949 335.2026 369.3482
60 80 100 120 140 160 180 200 220 240 260 280 300 320 340 360 380 400
m/z

**Compound 3i**

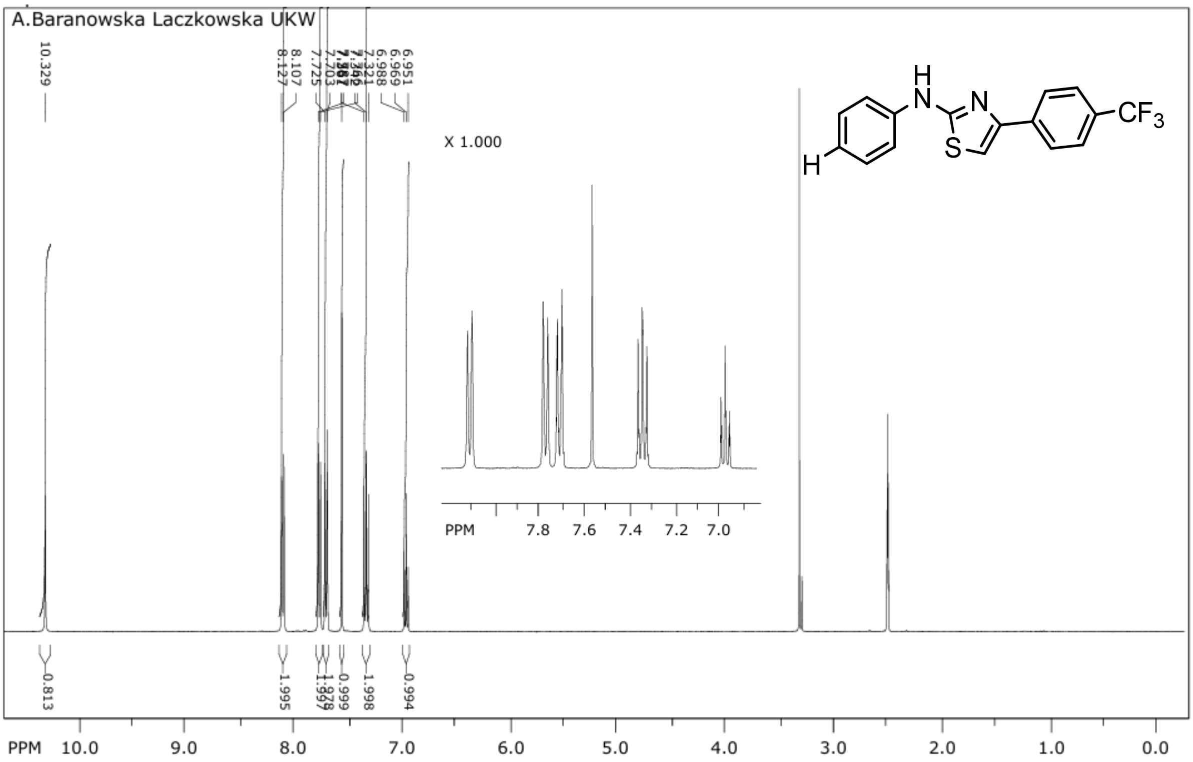
A.Baranowska Laczkowska UKW
10.329
8.127
8.107
7.725
7.703
7.321
6.988
6.969
6.951
X 1.000
H
N
N
S
H
CF3
PPM
7.8
7.6
7.4
7.2
7.0
0.813
1.995
0.999
1.998
0.994
PPM
10.0
9.0
8.0
7.0
6.0
5.0
4.0
3.0
2.0
1.0
0.0

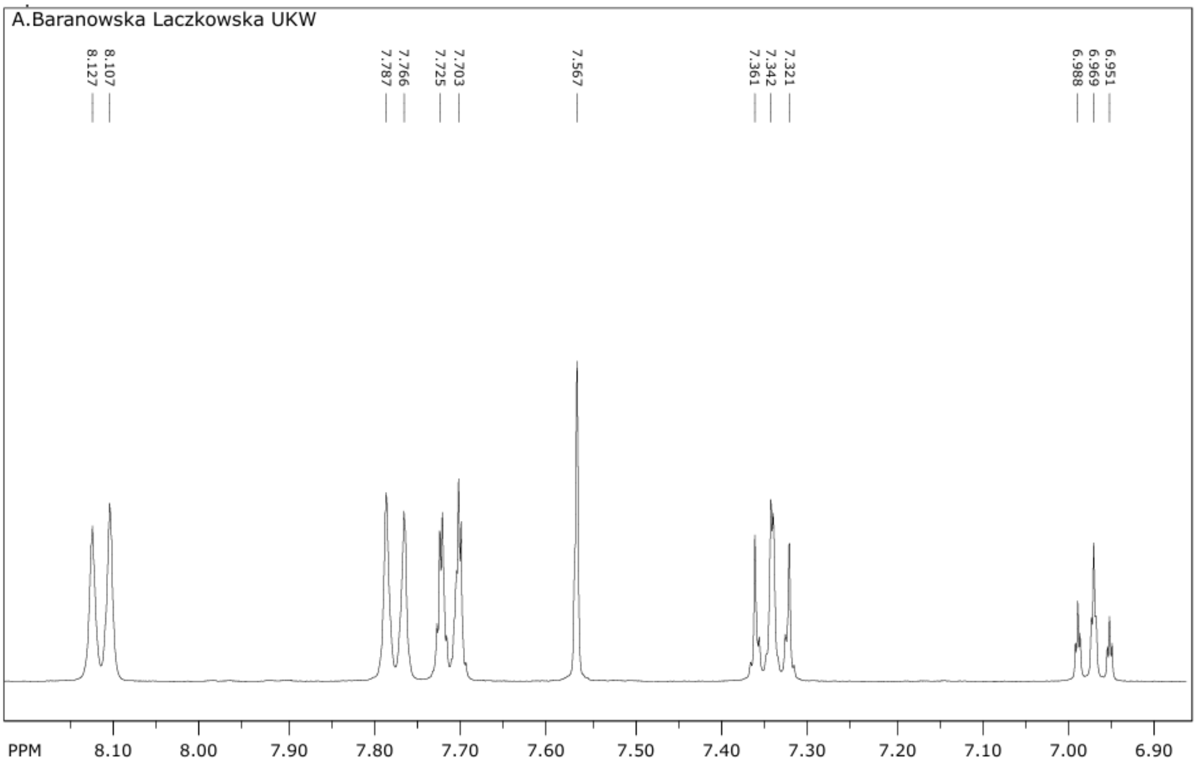
A.Baranowska Laczkowska UKW
8.127
8.107
7.787
7.766
7.725
7.703
7.567
7.361
7.342
7.321
6.988
6.969
6.951
PPM
8.10
8.00
7.90
7.80
7.70
7.60
7.50
7.40
7.30
7.20
7.10
7.00
6.90

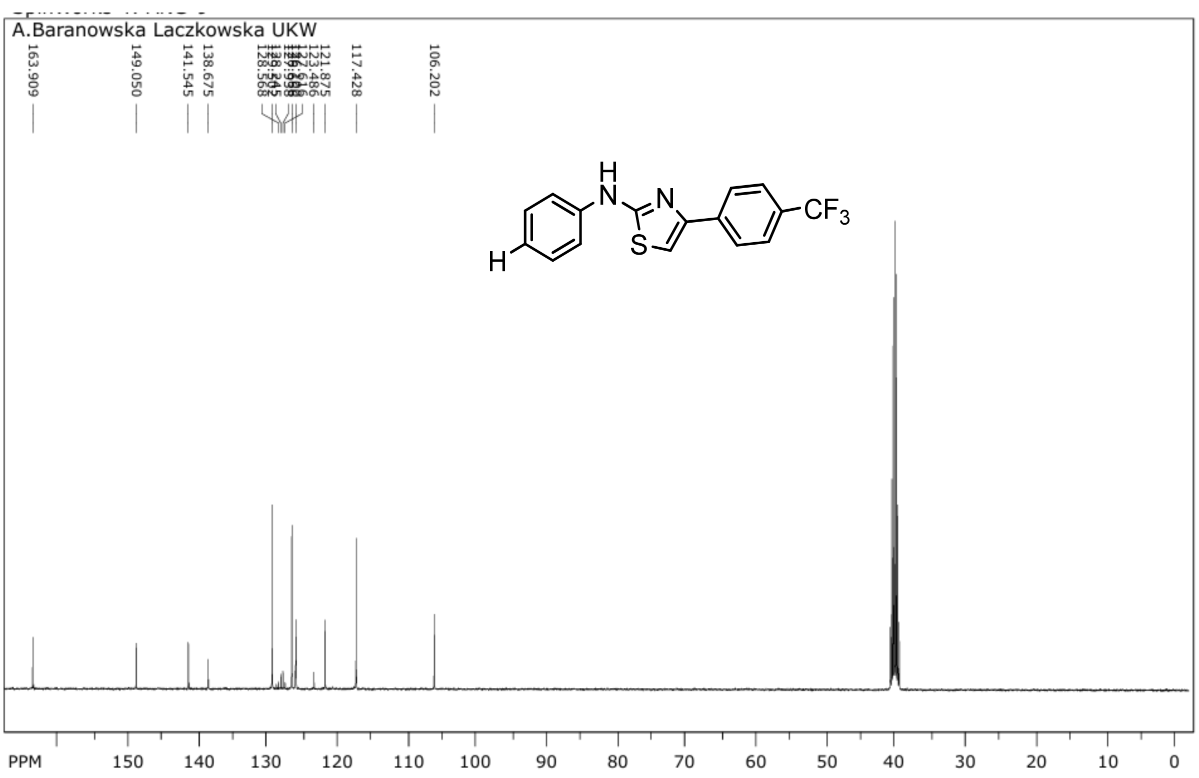
A.Baranowska Laczkowska UKW
163.909
149.050
141.545
138.675
128.568
121.875
117.428
106.202
PPM
150
140
130
120
110
100
90
80
70
60
50
40
30
20
10
0

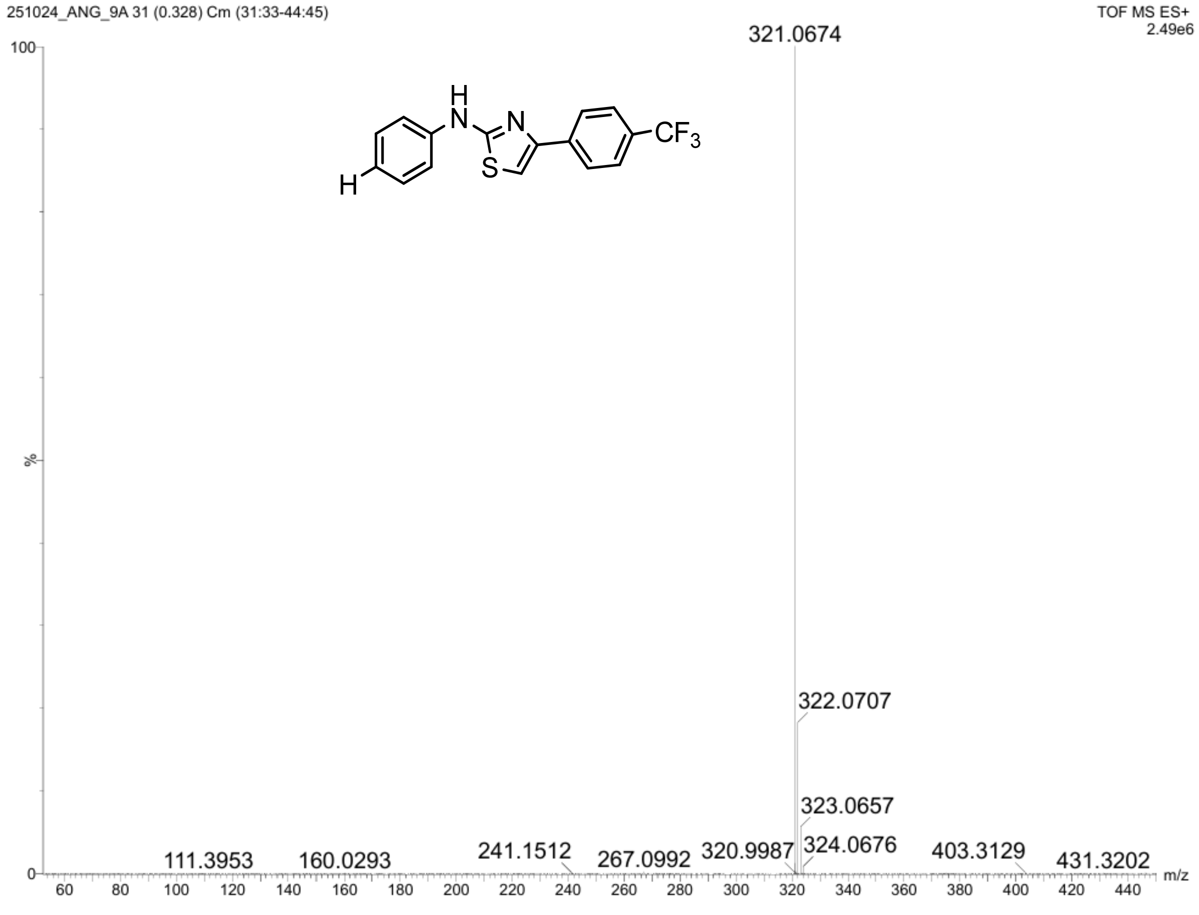
251024_ANG_9A 31 (0.328) Cm (31:33-44:45)
TOF MS ES+
2.49e6
321.0674
322.0707
323.0657
324.0676
111.3953
160.0293
241.1512
267.0992
320.9987
403.3129
431.3202
100
%
0
m/z

**Compound 3j**

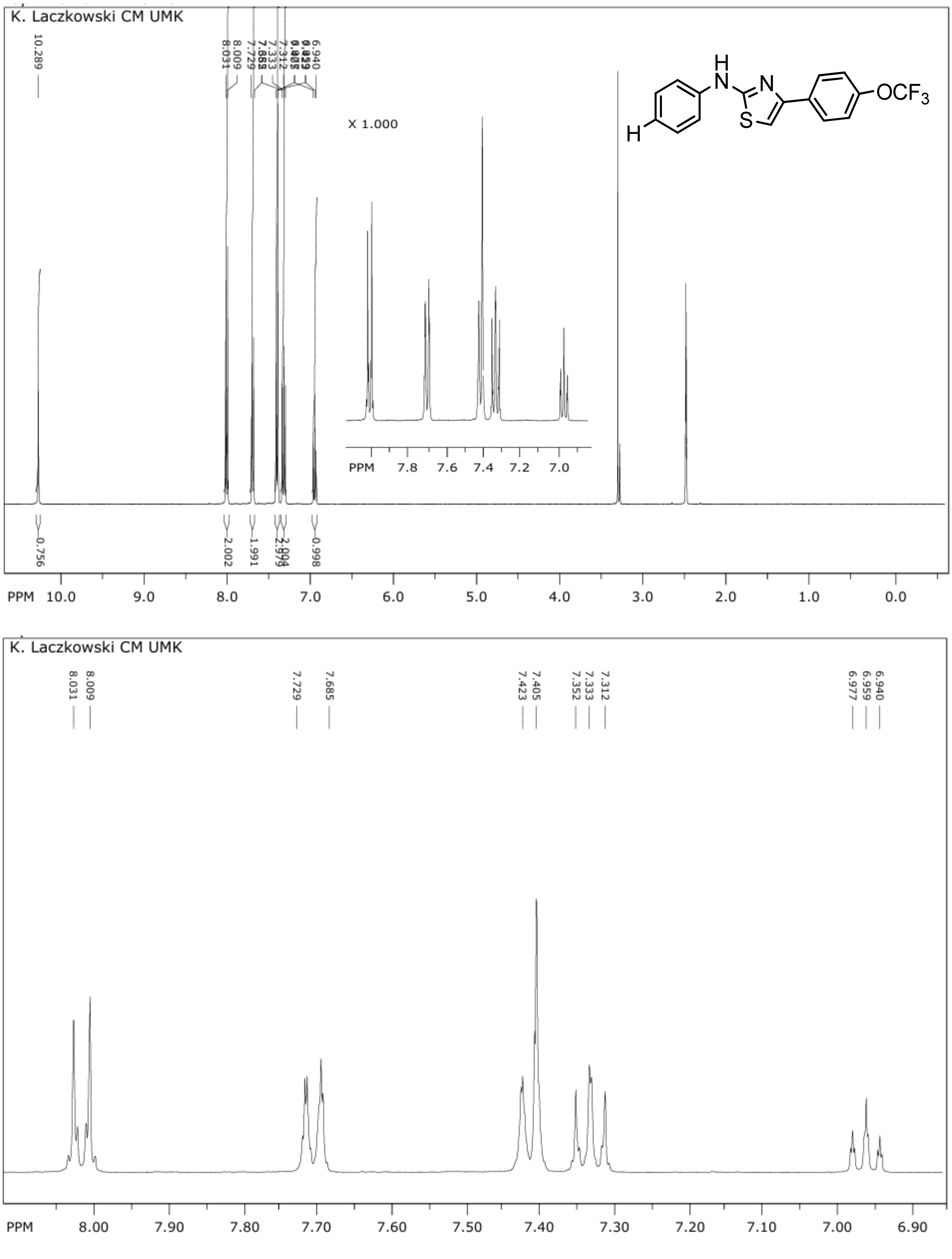

K. Laczkowski CM UMK
10.289
X 1.000
PPM
K. Laczkowski CM UMK
8.031
8.009
7.729
7.685
7.423
7.405
7.352
7.333
7.312
6.977
6.959
6.940
OCF3

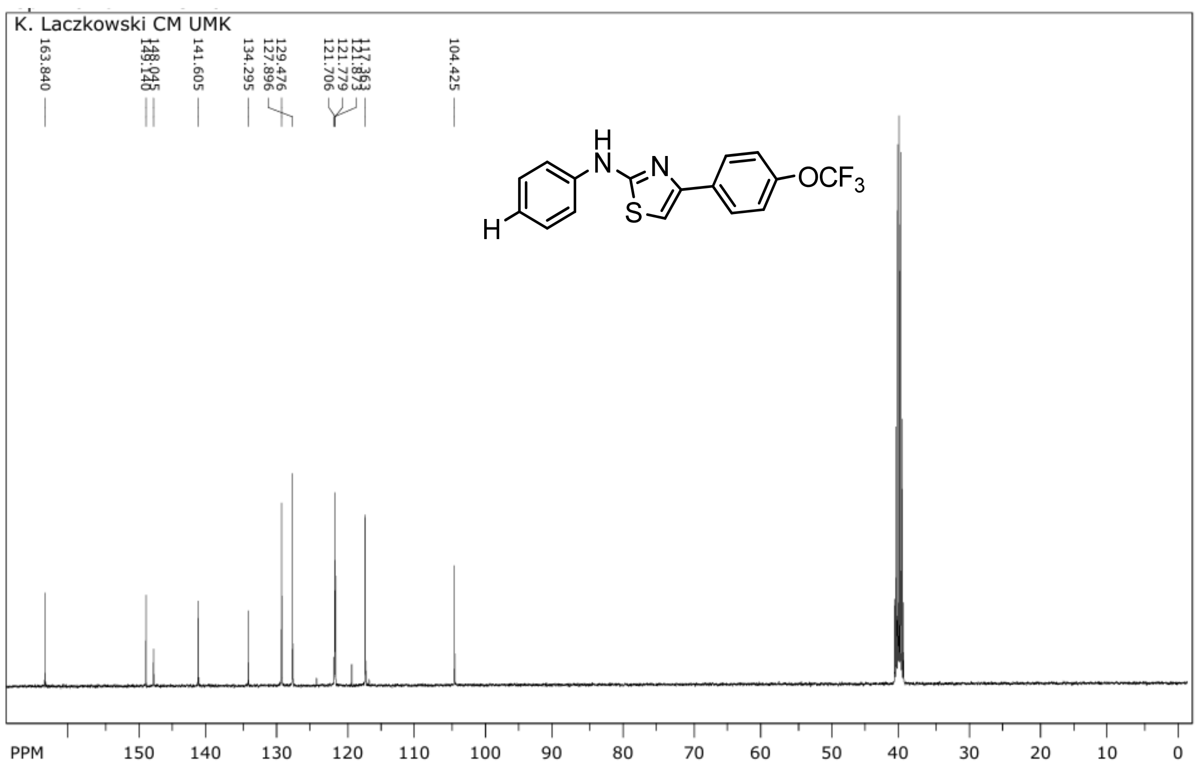
K. Laczkowski CM UMK
163.840
148.945
148.140
141.605
134.295
129.476
127.896
121.779
121.706
117.363
104.425
OCF3
PPM
150
140
130
120
110
100
90
80
70
60
50
40
30
20
10
0

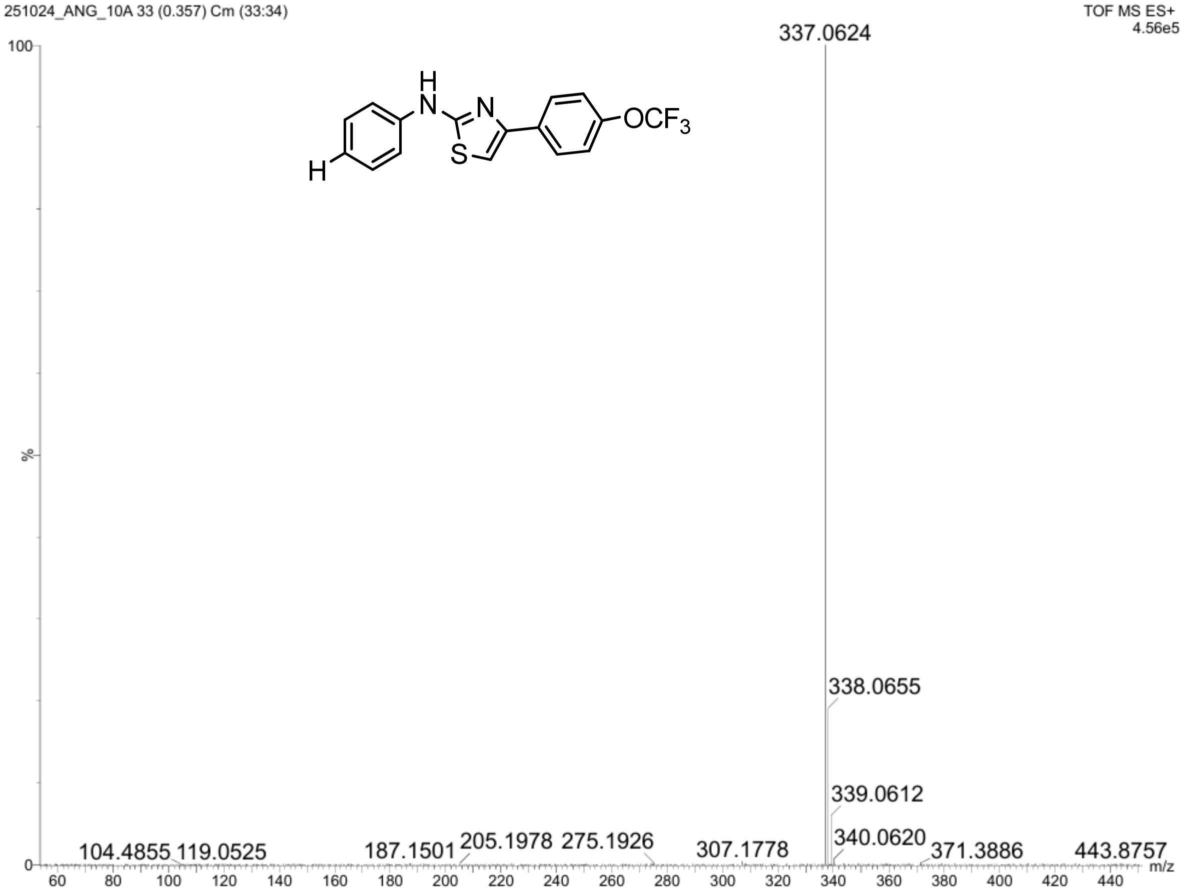
251024_ANG_10A 33 (0.357) Cm (33:34)
TOF MS ES+
4.56e5
337.0624
338.0655
339.0612
340.0620
104.4855
119.0525
187.1501
205.1978
275.1926
307.1778
371.3886
443.8757
%
m/z
OCF3

**Compound 3k**

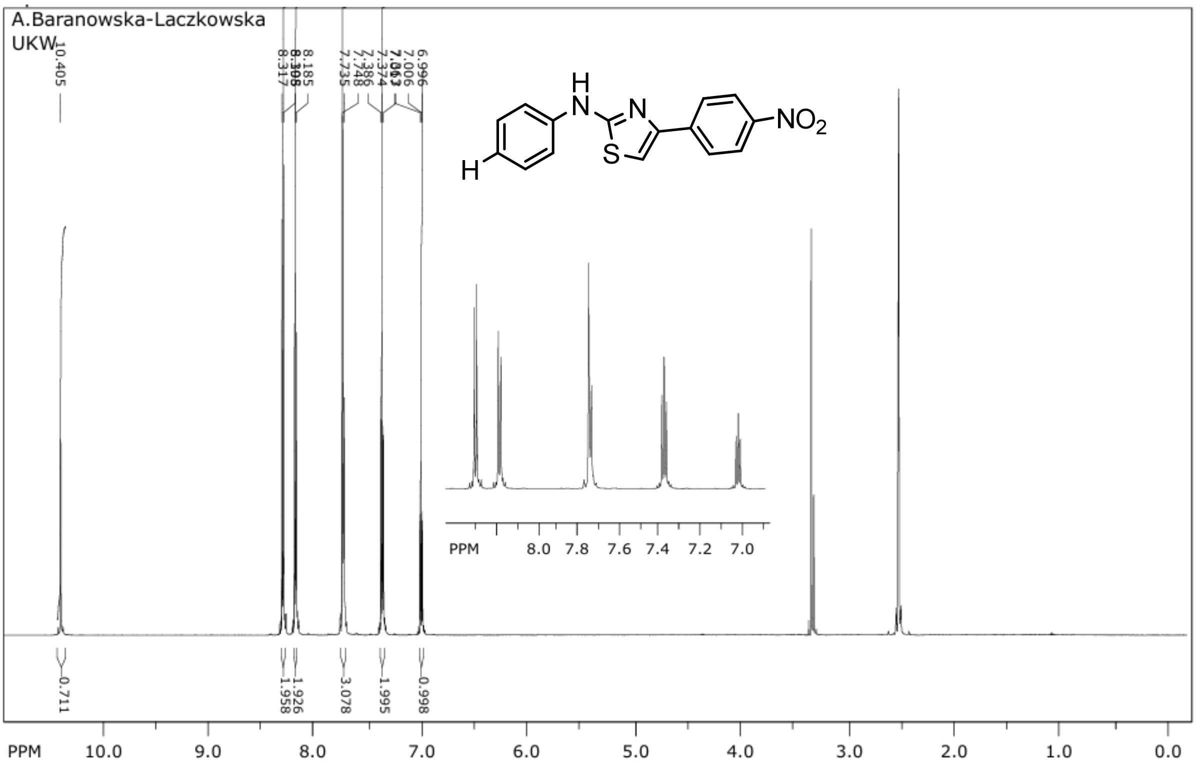
A.Baranowska-Laczkowska
UKW
10.405
8.317
8.185
7.735
7.748
7.386
7.374
7.006
6.996
H
N
N
S
H
NO2
0.711
1.958
1.926
3.078
1.995
0.998
PPM
8.0 7.8 7.6 7.4 7.2 7.0
PPM 10.0 9.0 8.0 7.0 6.0 5.0 4.0 3.0 2.0 1.0 0.0

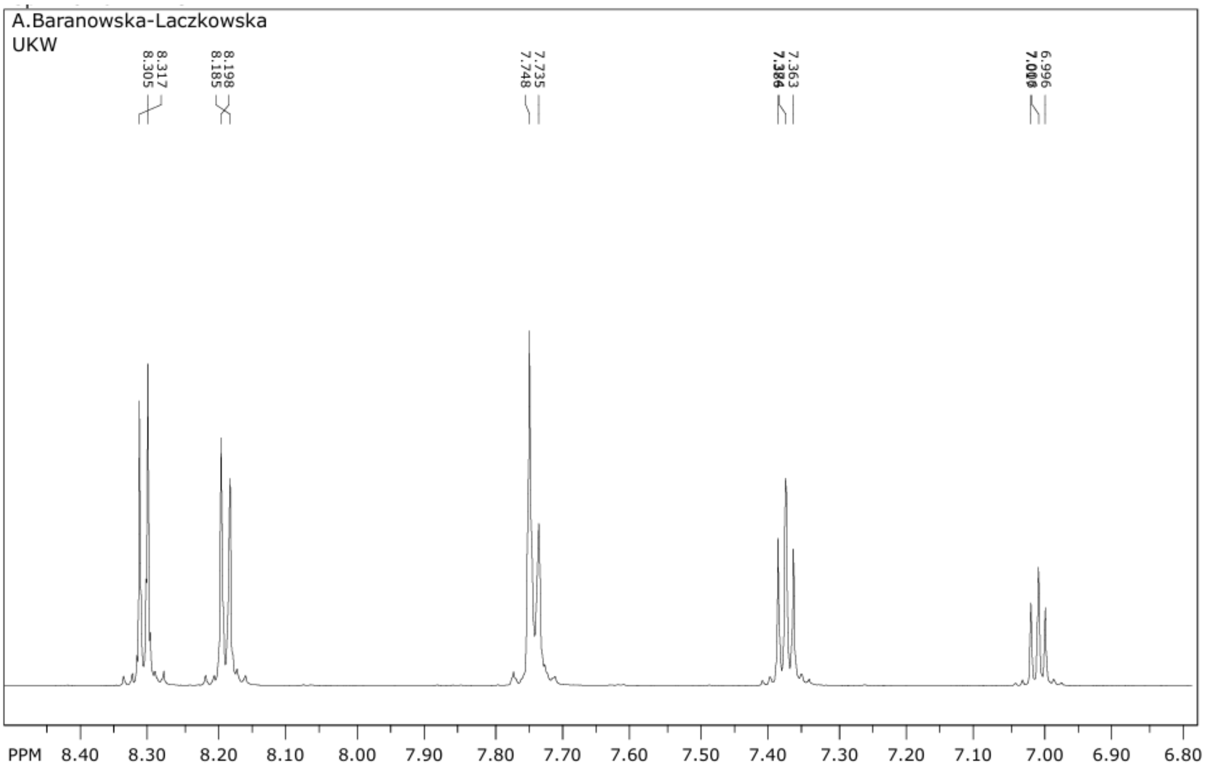
A.Baranowska-Laczkowska
UKW
8.317
8.305
8.198
8.185
7.735
7.748
7.363
6.996
PPM 8.40 8.30 8.20 8.10 8.00 7.90 7.80 7.70 7.60 7.50 7.40 7.30 7.20 7.10 7.00 6.90 6.80

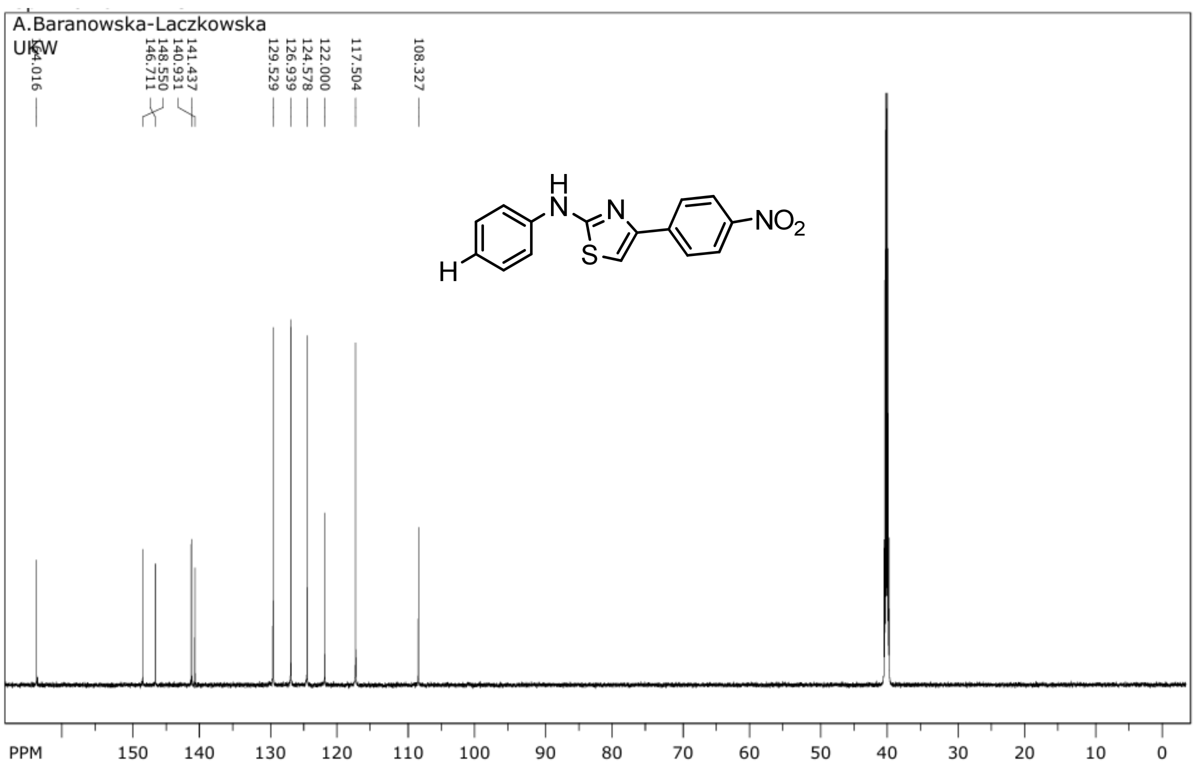
A.Baranowska-Laczkowska
UKW
164.016
148.550
146.711
141.437
140.931
129.529
126.939
124.578
122.000
117.504
108.327
H
N
N
S
NO2
H
PPM
150
140
130
120
110
100
90
80
70
60
50
40
30
20
10
0

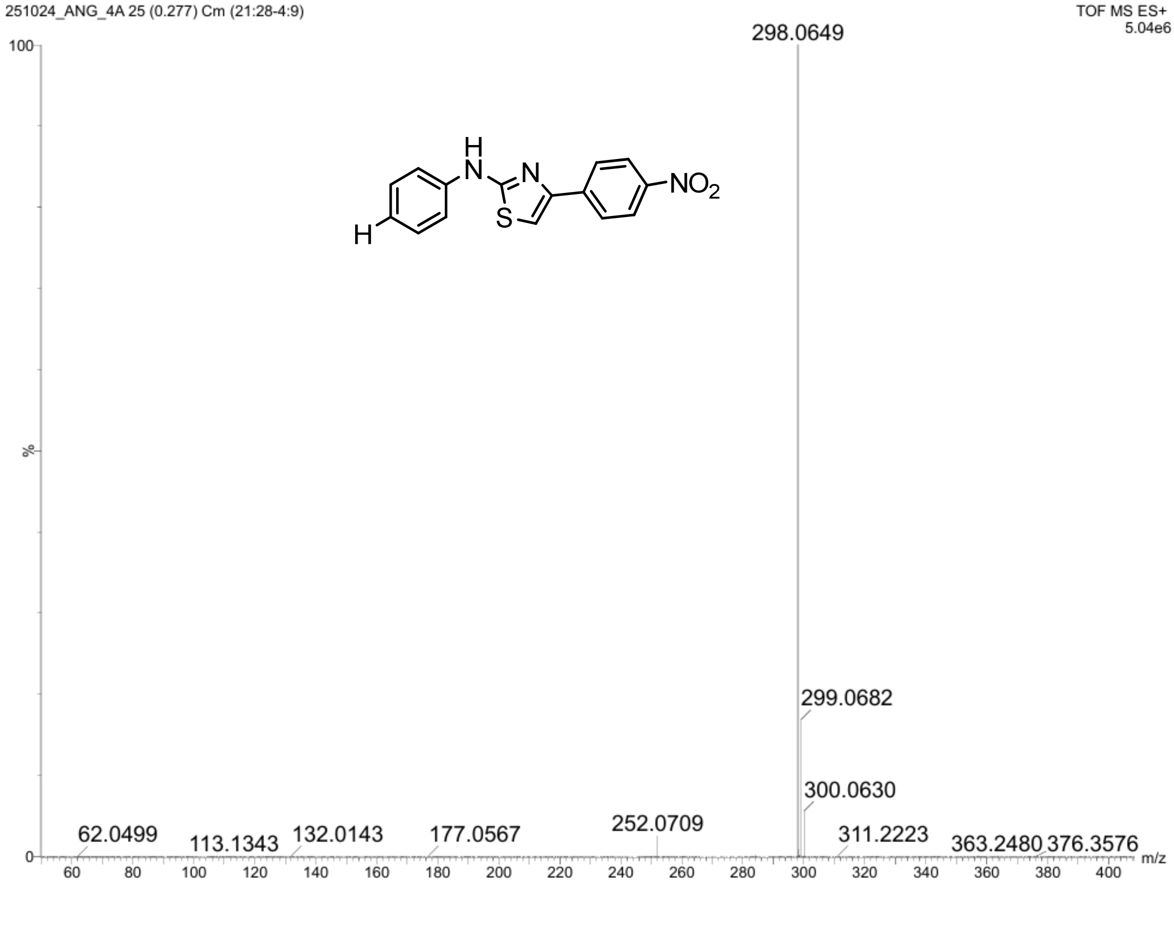
251024_ANG_4A 25 (0.277) Cm (21:28-4:9)
TOF MS ES+
5.04e6
298.0649
100
%
0
H
N
N
S
NO2
H
299.0682
300.0630
62.0499
113.1343
132.0143
177.0567
252.0709
311.2223
363.2480
376.3576
m/z
60
80
100
120
140
160
180
200
220
240
260
280
300
320
340
360
380
400

**Compound 3l**

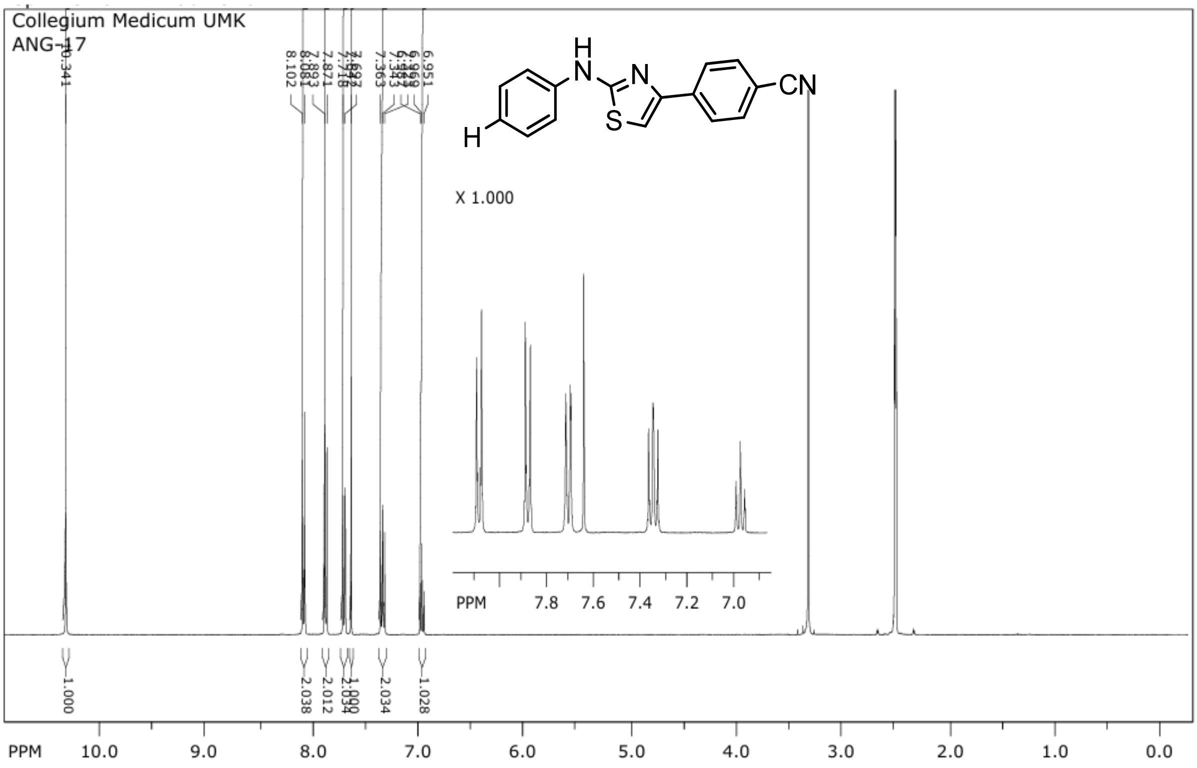
Collegium Medicum UMK
ANG-17
X 1.000
PPM 10.0 9.0 8.0 7.0 6.0 5.0 4.0 3.0 2.0 1.0 0.0

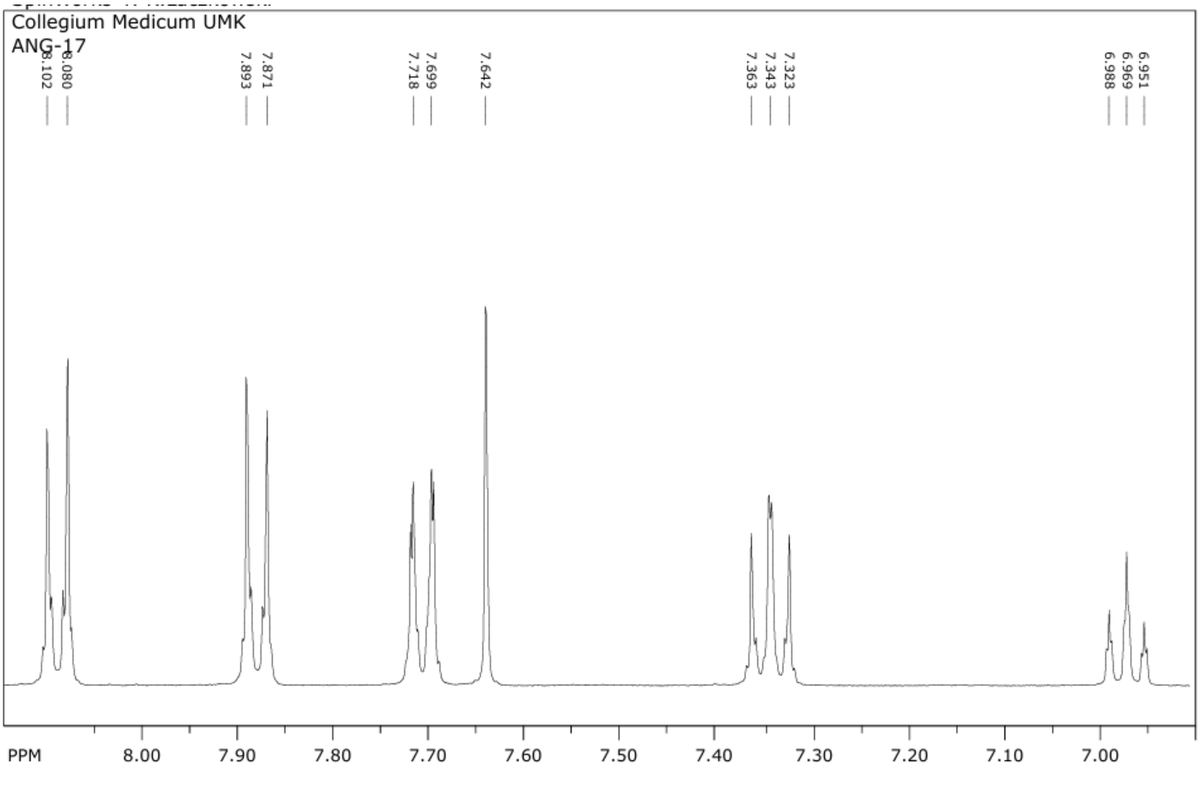
Collegium Medicum UMK
ANG-17
8.102
8.080
7.893
7.871
7.718
7.699
7.642
7.363
7.343
7.323
6.988
6.969
6.951
PPM 8.00 7.90 7.80 7.70 7.60 7.50 7.40 7.30 7.20 7.10 7.00

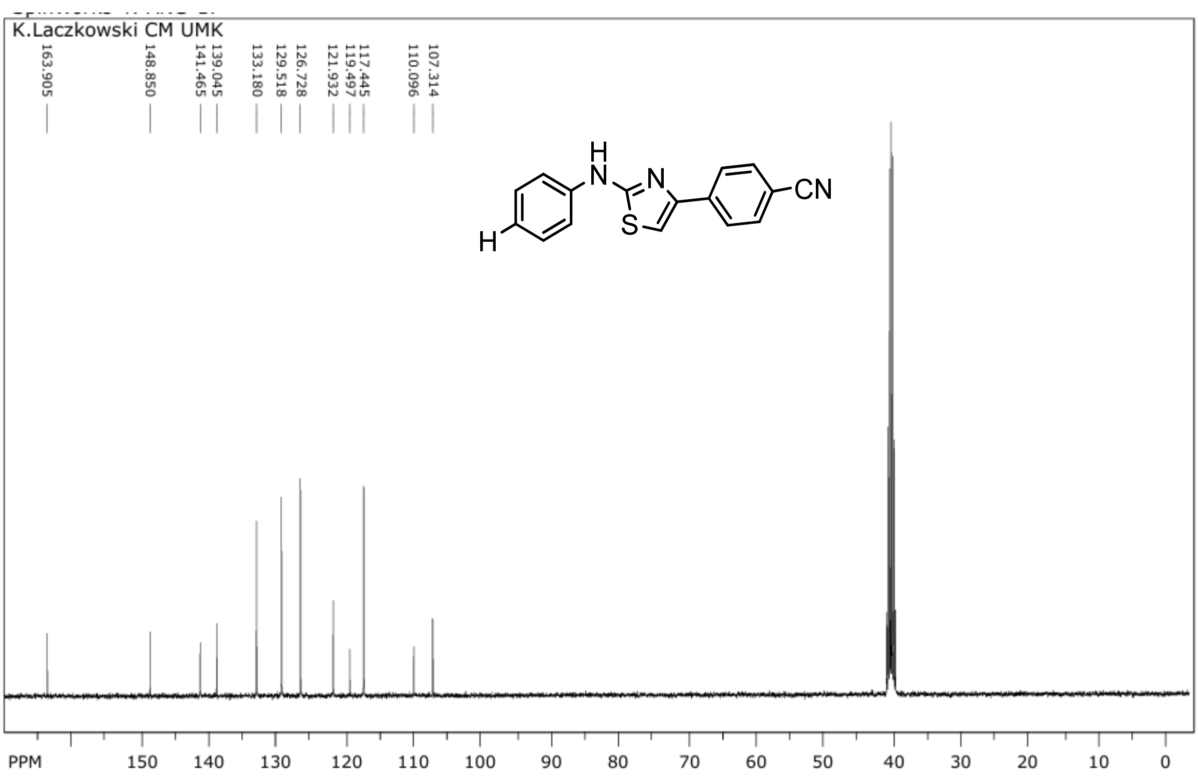
K.Laczkowski CM UMK
163.905
148.850
141.465
139.045
133.180
129.518
126.728
121.932
119.497
117.445
110.096
107.314
H
N
N
S
CN
H
PPM
150
140
130
120
110
100
90
80
70
60
50
40
30
20
10
0

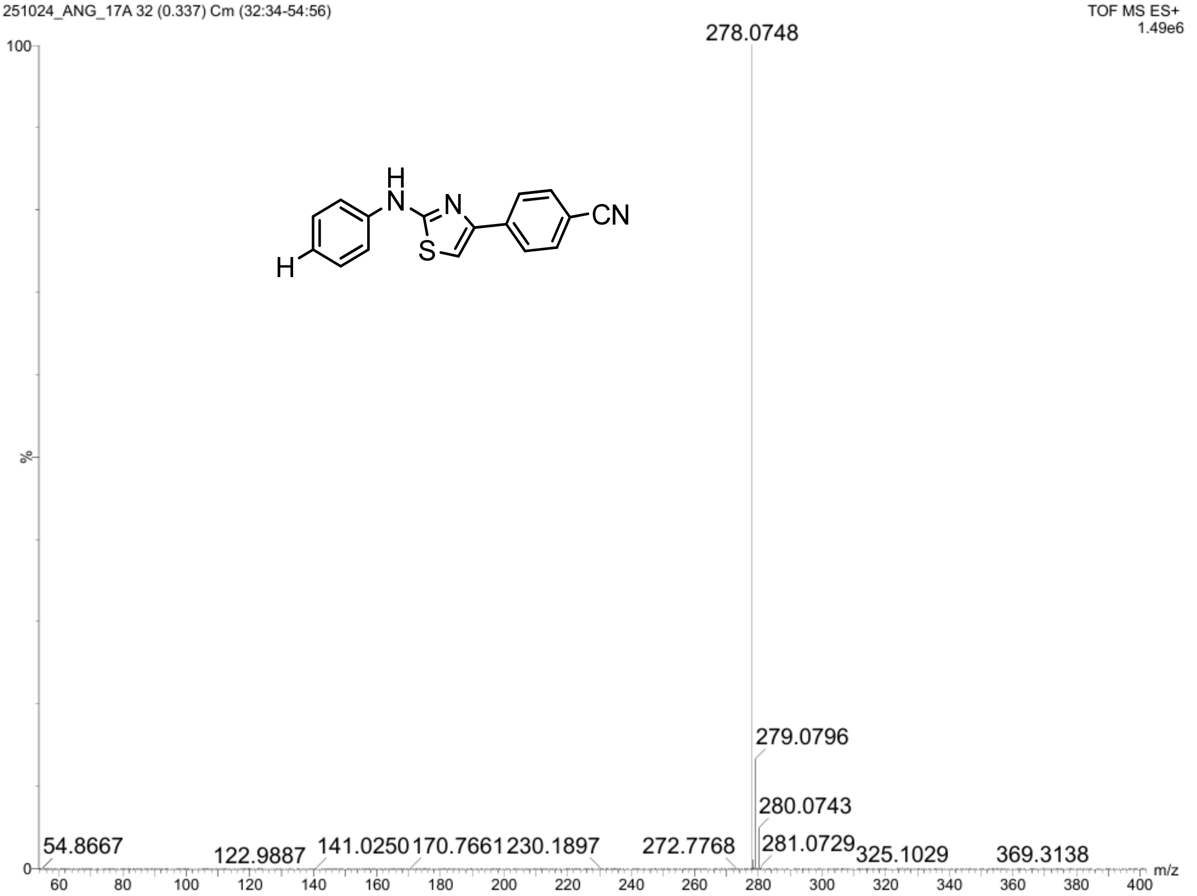
251024_ANG_17A 32 (0.337) Cm (32:34-54:56)
TOF MS ES+
1.49e6
278.0748
100
%
0
279.0796
280.0743
54.8667
122.9887
141.0250
170.7661
230.1897
272.7768
281.0729
325.1029
369.3138
m/z
H
N
N
S
CN
H

**Compound 3m**

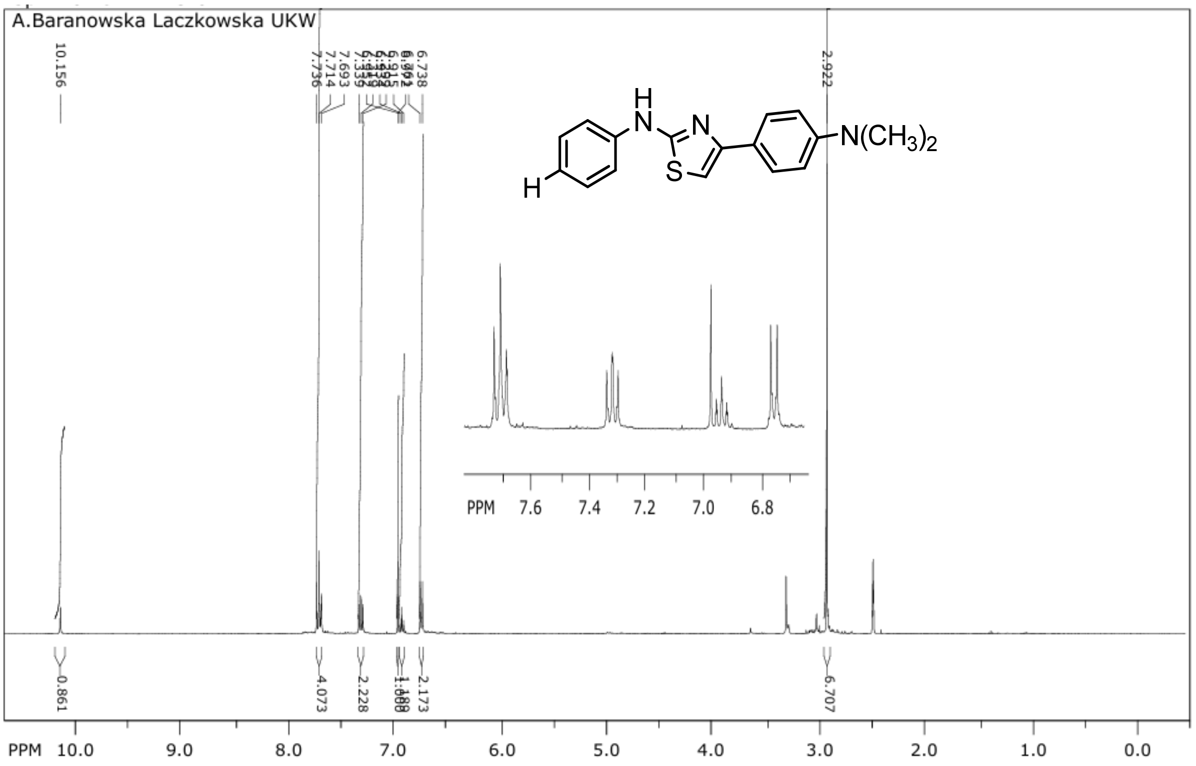
A.Baranowska Laczkowska UKW
10.156
7.736
7.714
7.693
2.922
6.738
N(CH3)2
H
N
S
0.861
4.073
2.228
2.173
6.707
PPM 7.6 7.4 7.2 7.0 6.8
PPM 10.0 9.0 8.0 7.0 6.0 5.0 4.0 3.0 2.0 1.0 0.0

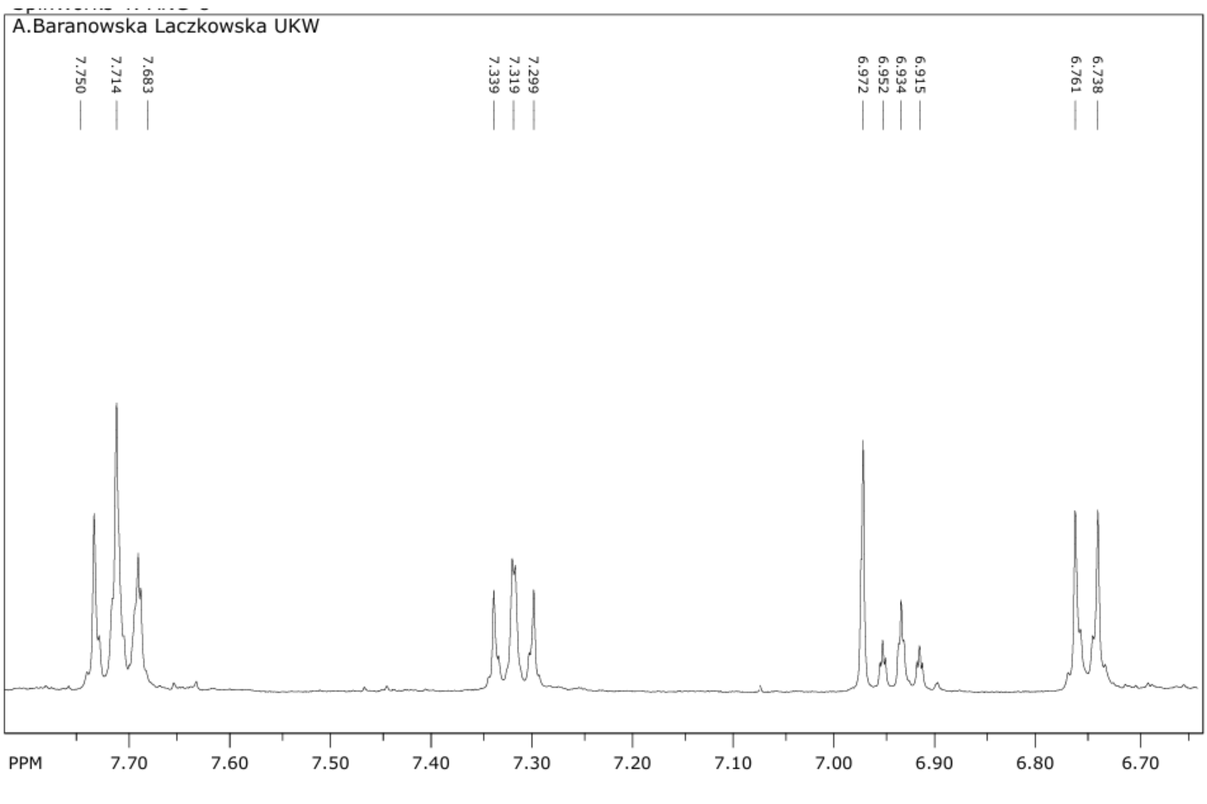
A.Baranowska Laczkowska UKW
7.750
7.714
7.683
7.339
7.319
7.299
6.972
6.952
6.934
6.915
6.761
6.738
PPM 7.70 7.60 7.50 7.40 7.30 7.20 7.10 7.00 6.90 6.80 6.70

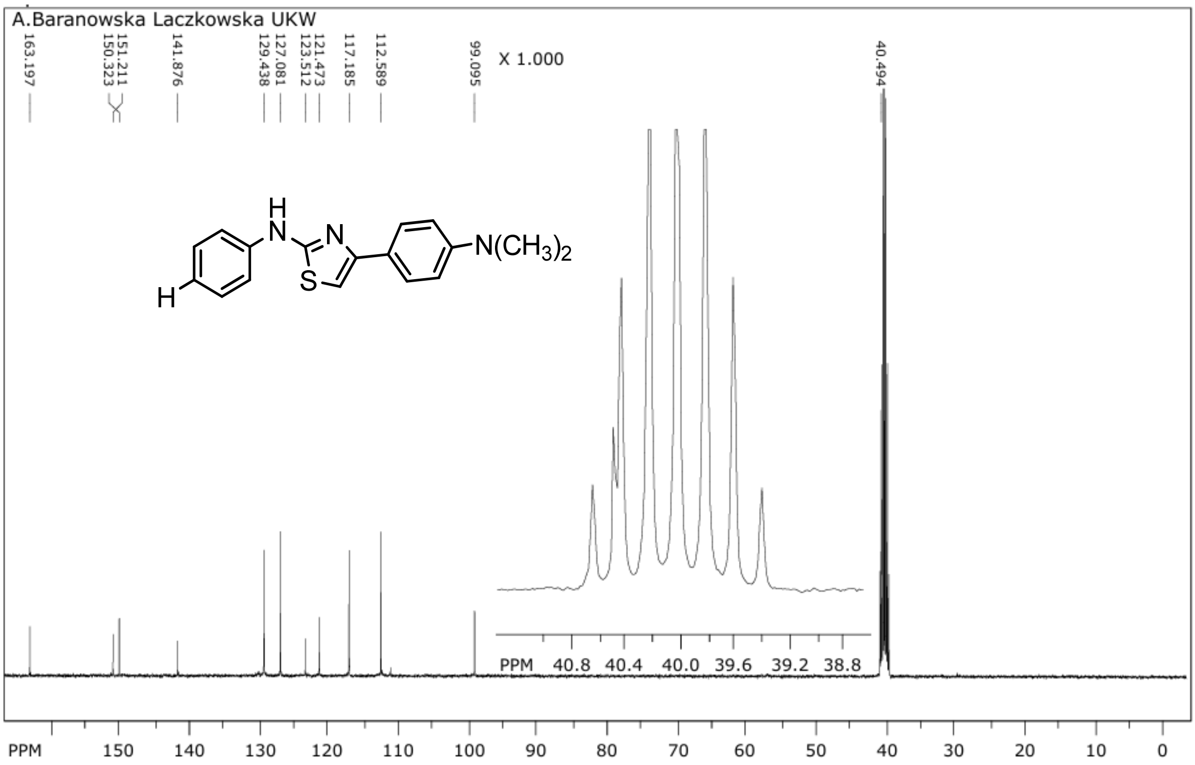

A.Baranowska Laczkowska UKW
163.197
151.211
150.323
141.876
129.438
127.081
123.512
121.473
117.185
112.589
99.095
X 1.000
40.494
N(CH3)2
H
S
N
PPM 40.8 40.4 40.0 39.6 39.2 38.8
PPM 150 140 130 120 110 100 90 80 70 60 50 40 30 20 10 0


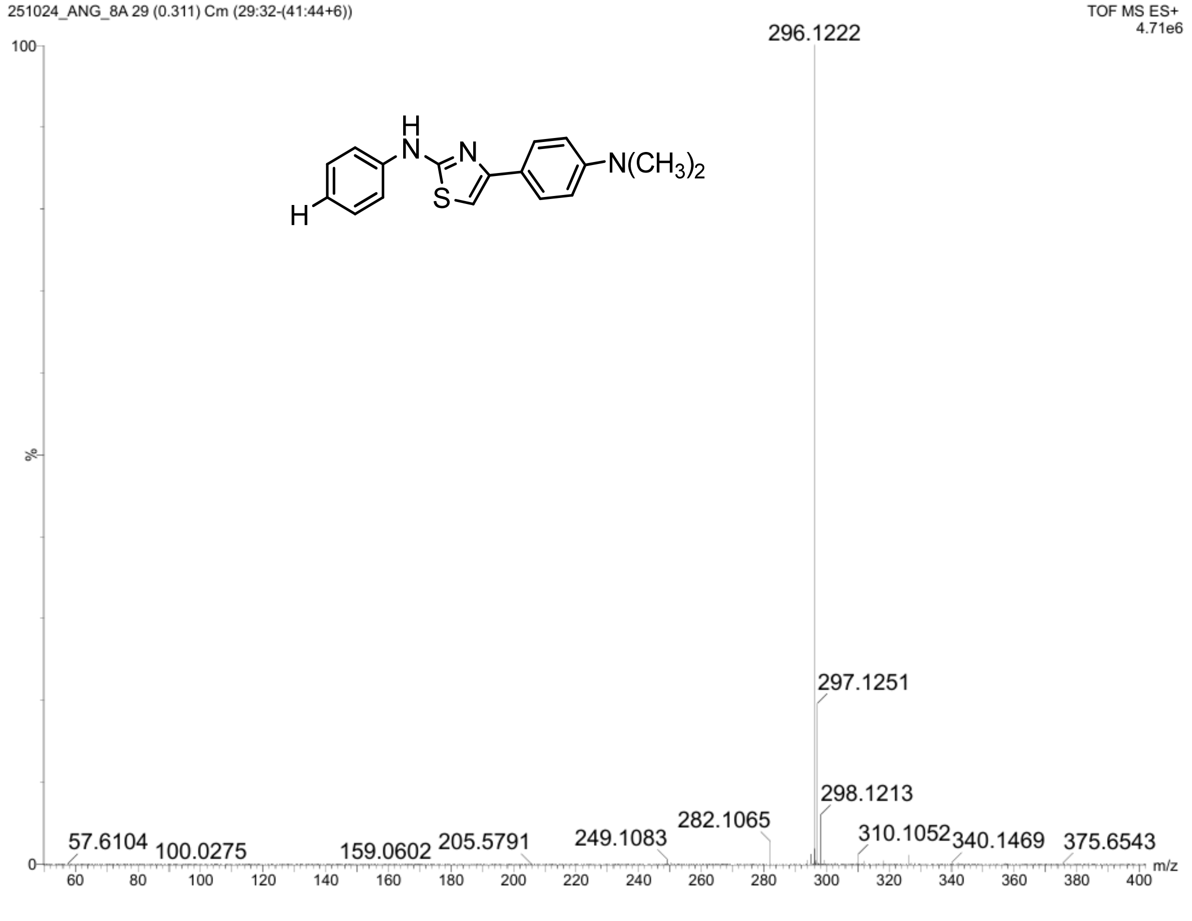

251024_ANG_8A 29 (0.311) Cm (29:32-(41:44+6))
TOF MS ES+
4.71e6
296.1222
297.1251
298.1213
57.6104
100.0275
159.0602
205.5791
249.1083
282.1065
310.1052
340.1469
375.6543
%
m/z
N(CH3)2

**Compound 3n**

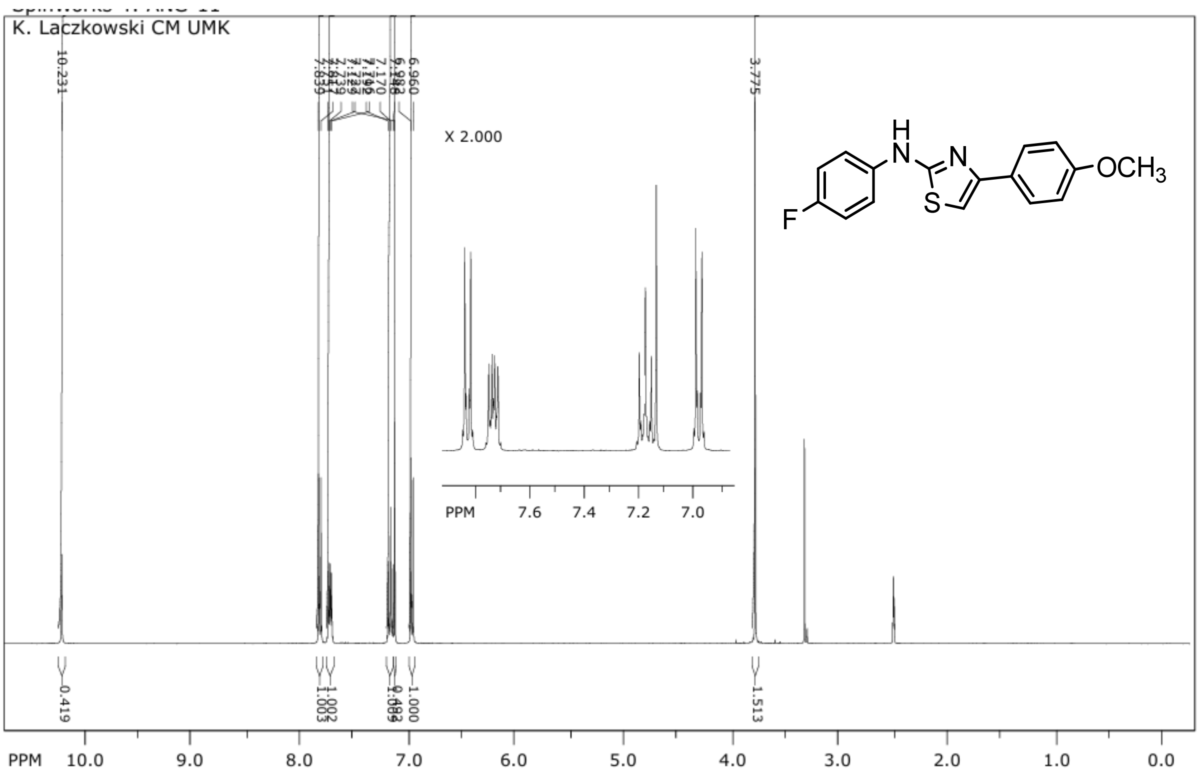
K. Laczkowski CM UMK
10.231
6.960
3.775
X 2.000
PPM
7.6
7.4
7.2
7.0
F
H
N
N
S
$OCH_3$
0.419
1.000
1.513
PPM
10.0
9.0
8.0
7.0
6.0
5.0
4.0
3.0
2.0
1.0
0.0

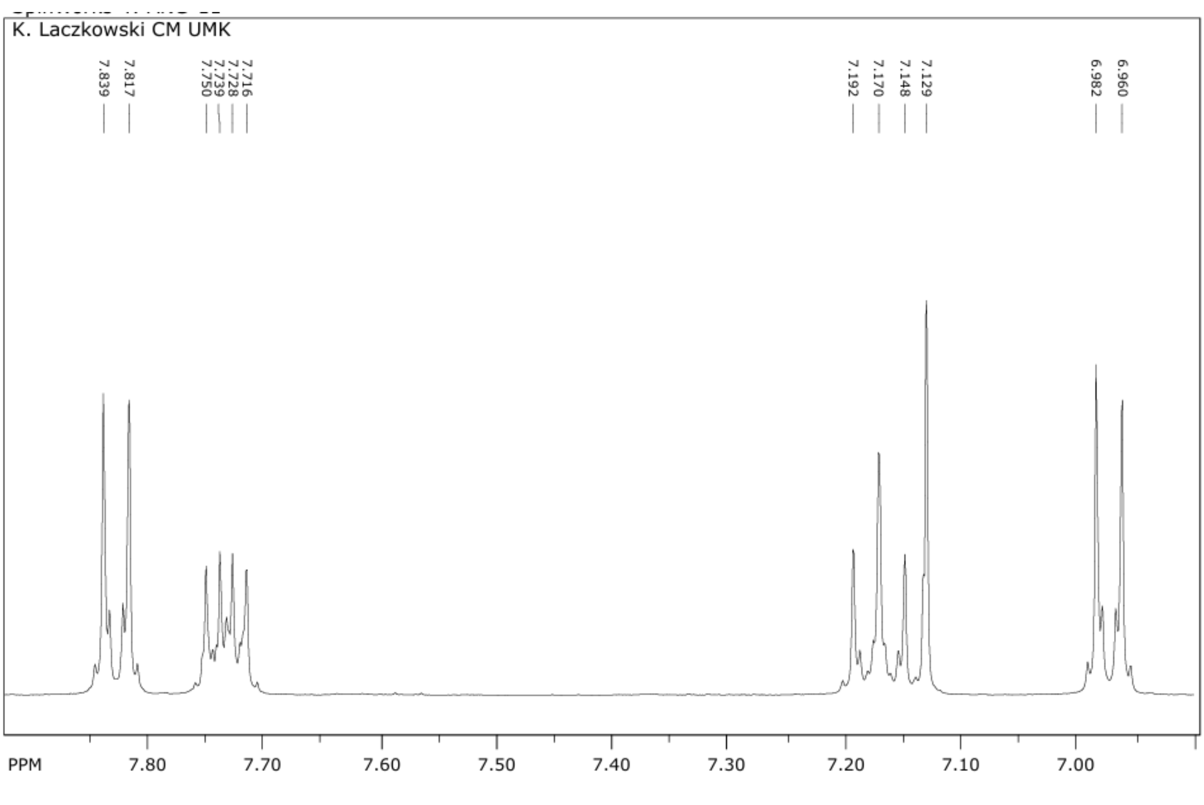
K. Laczkowski CM UMK
7.839
7.817
7.750
7.739
7.728
7.716
7.192
7.170
7.148
7.129
6.982
6.960
PPM
7.80
7.70
7.60
7.50
7.40
7.30
7.20
7.10
7.00

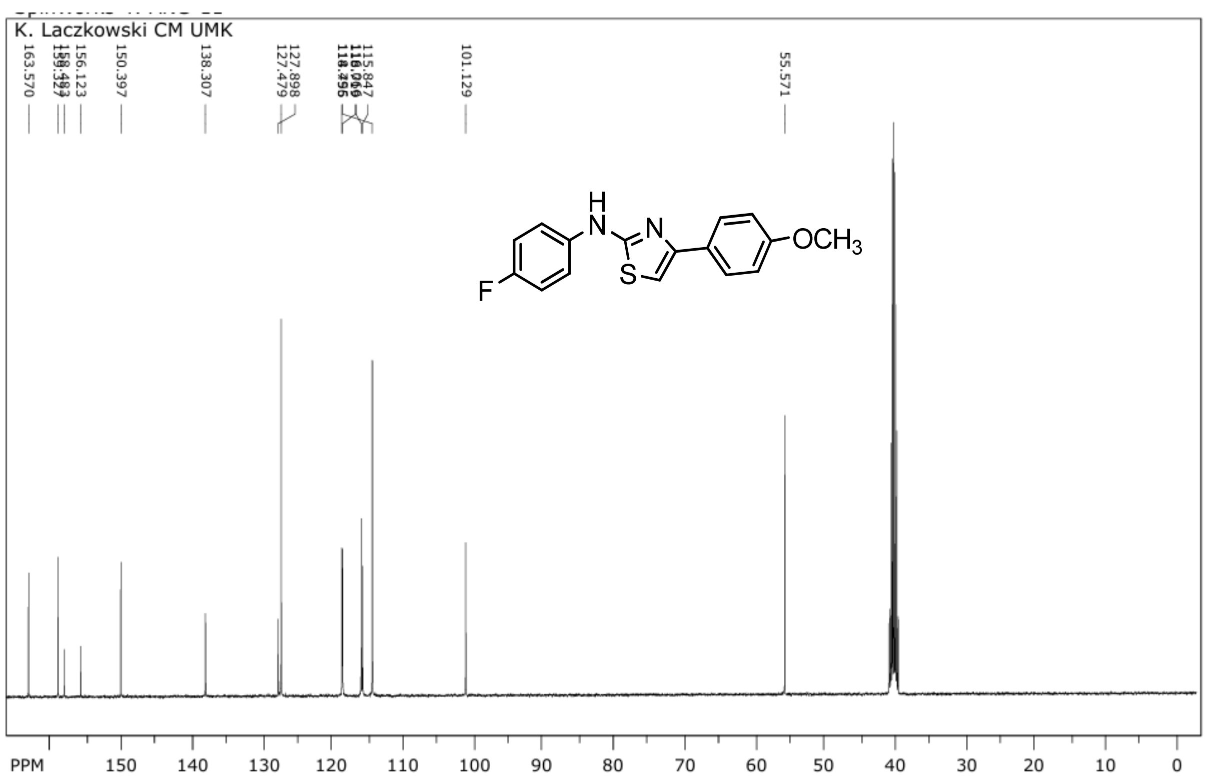
K. Laczkowski CM UMK
163.570
156.123
150.397
138.307
127.898
127.479
115.847
101.129
55.571
OCH3
F
H
N
S
PPM
150
140
130
120
110
100
90
80
70
60
50
40
30
20
10
0

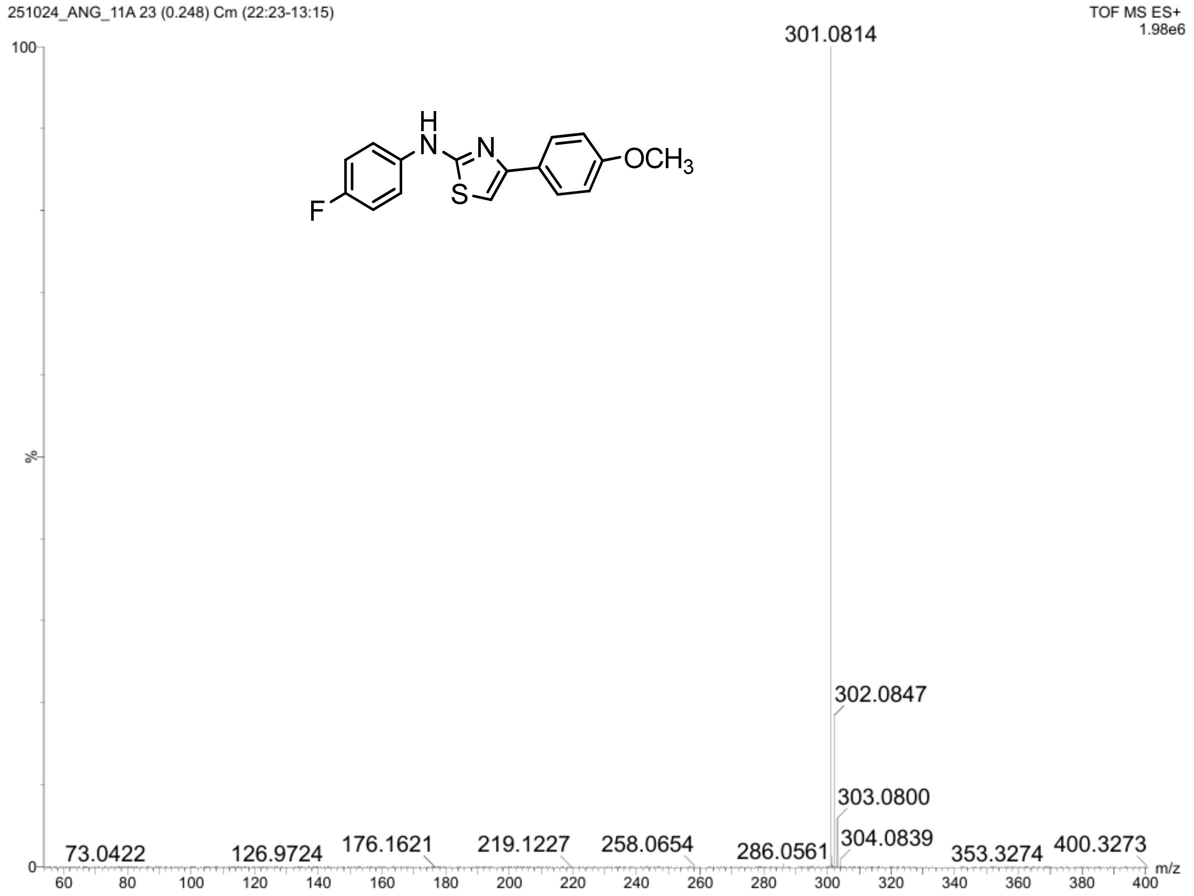
251024_ANG_11A 23 (0.248) Cm (22:23-13:15)
TOF MS ES+
1.98e6
301.0814
302.0847
303.0800
304.0839
73.0422
126.9724
176.1621
219.1227
258.0654
286.0561
353.3274
400.3273
100
%
0
m/z
OCH3
F
H
N
S

**Compound 3o**

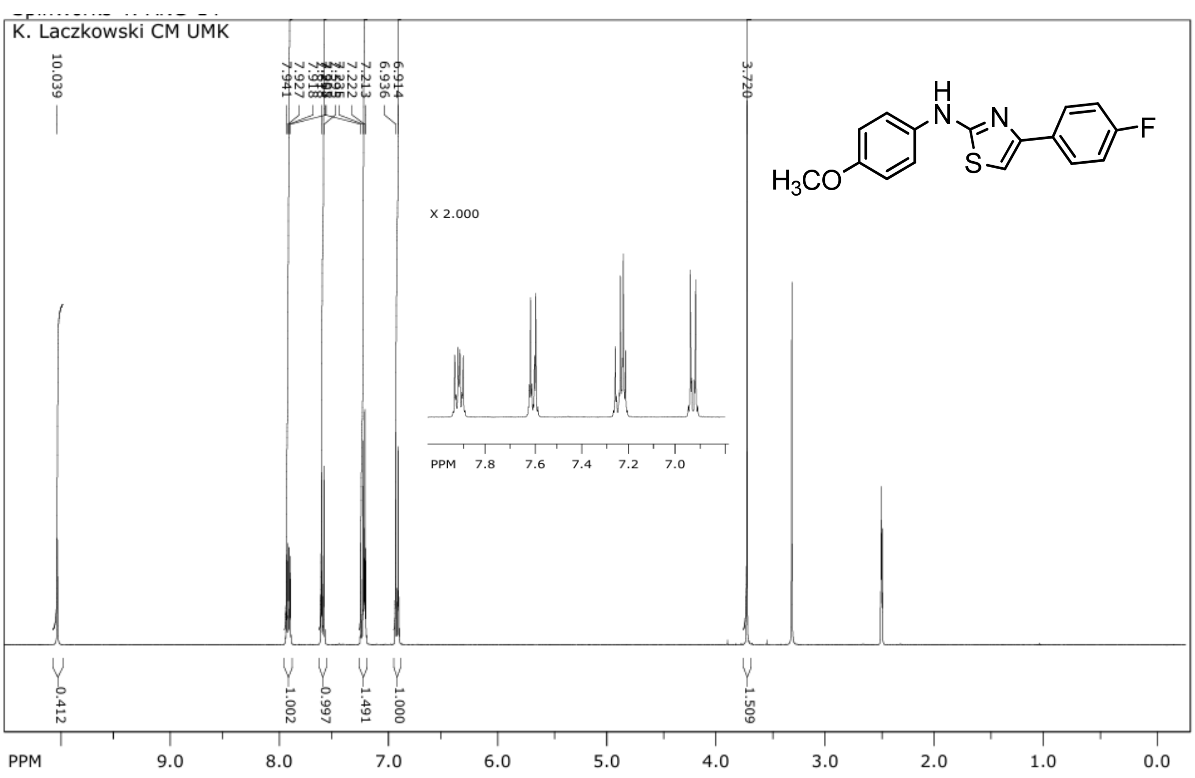
K. Laczkowski CM UMK
10.039
7.941
7.927
7.918
7.222
7.213
6.936
6.914
3.720
H
N
N
S
F
H3CO
X 2.000
PPM 7.8 7.6 7.4 7.2 7.0
0.412
1.002
0.997
1.491
1.000
1.509
PPM 9.0 8.0 7.0 6.0 5.0 4.0 3.0 2.0 1.0 0.0

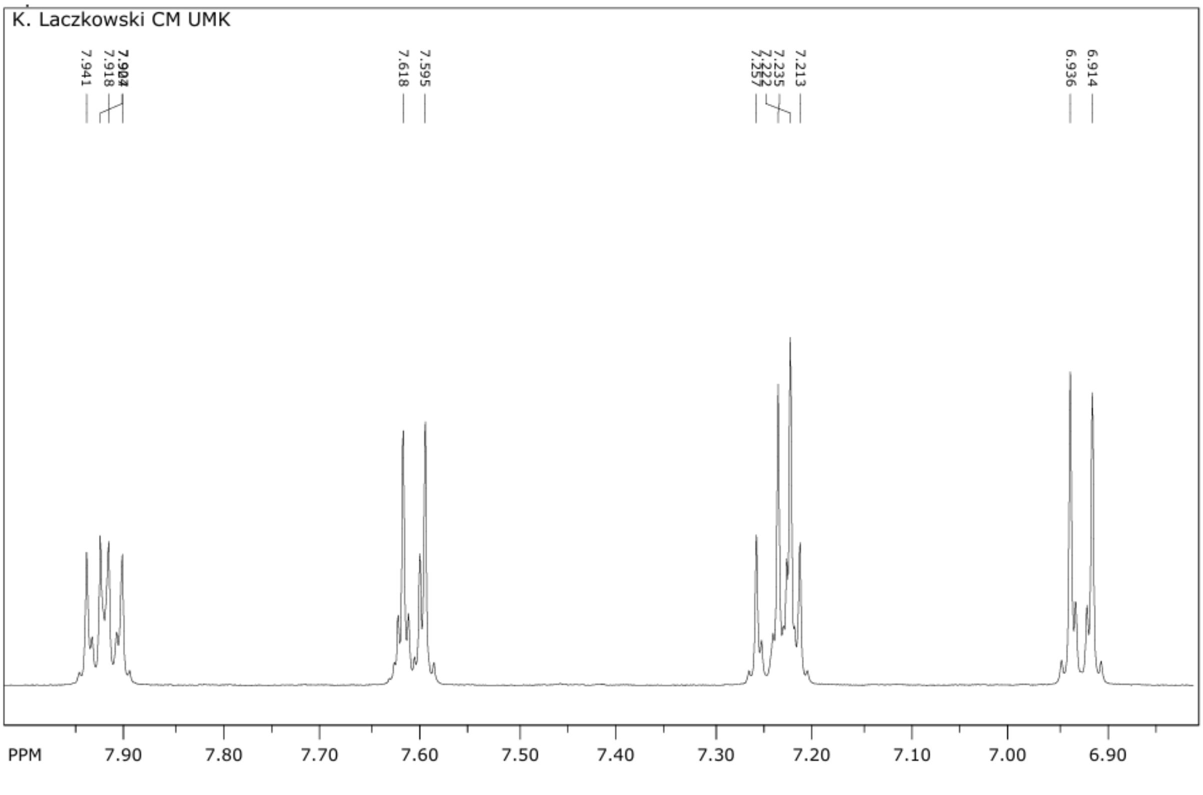
K. Laczkowski CM UMK
7.941
7.918
7.618
7.595
7.235
7.213
6.936
6.914
PPM 7.90 7.80 7.70 7.60 7.50 7.40 7.30 7.20 7.10 7.00 6.90

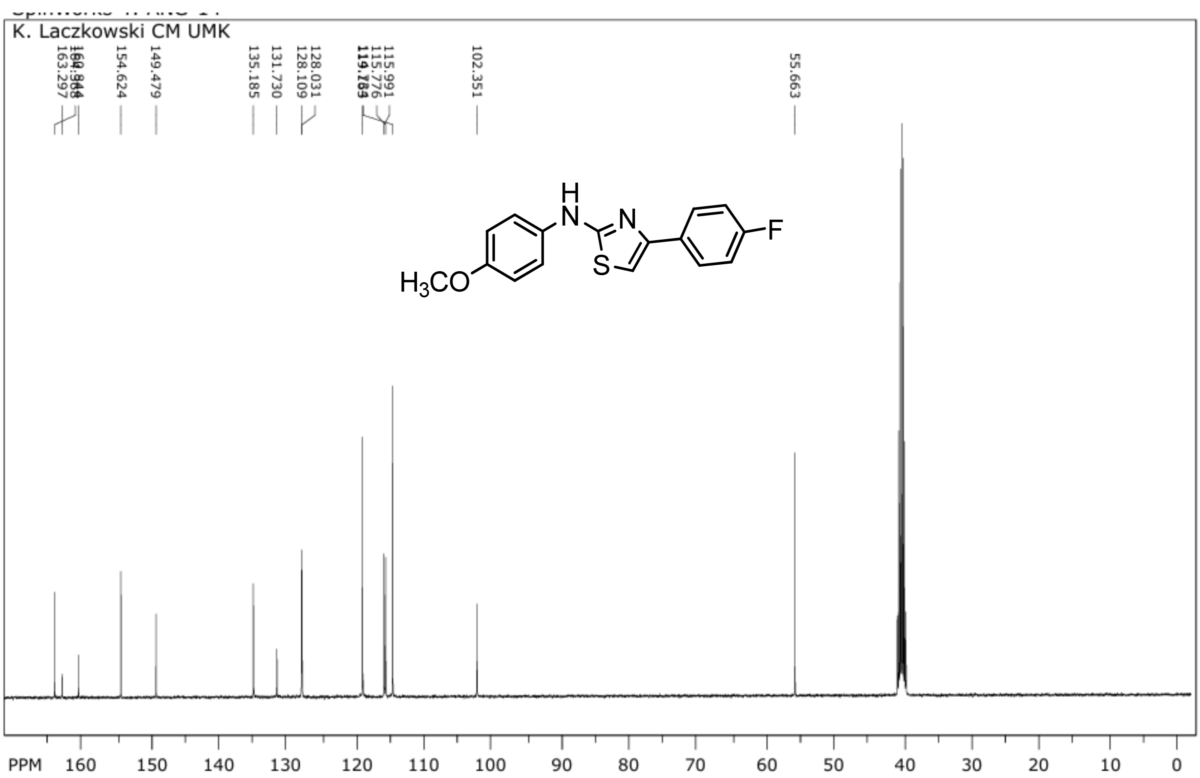

K. Laczkowski CM UMK
163.297
154.624
149.479
135.185
131.730
128.109
128.031
115.991
115.776
102.351
55.663
H
N
N
S
F
$H_3CO$
PPM 160 150 140 130 120 110 100 90 80 70 60 50 40 30 20 10 0


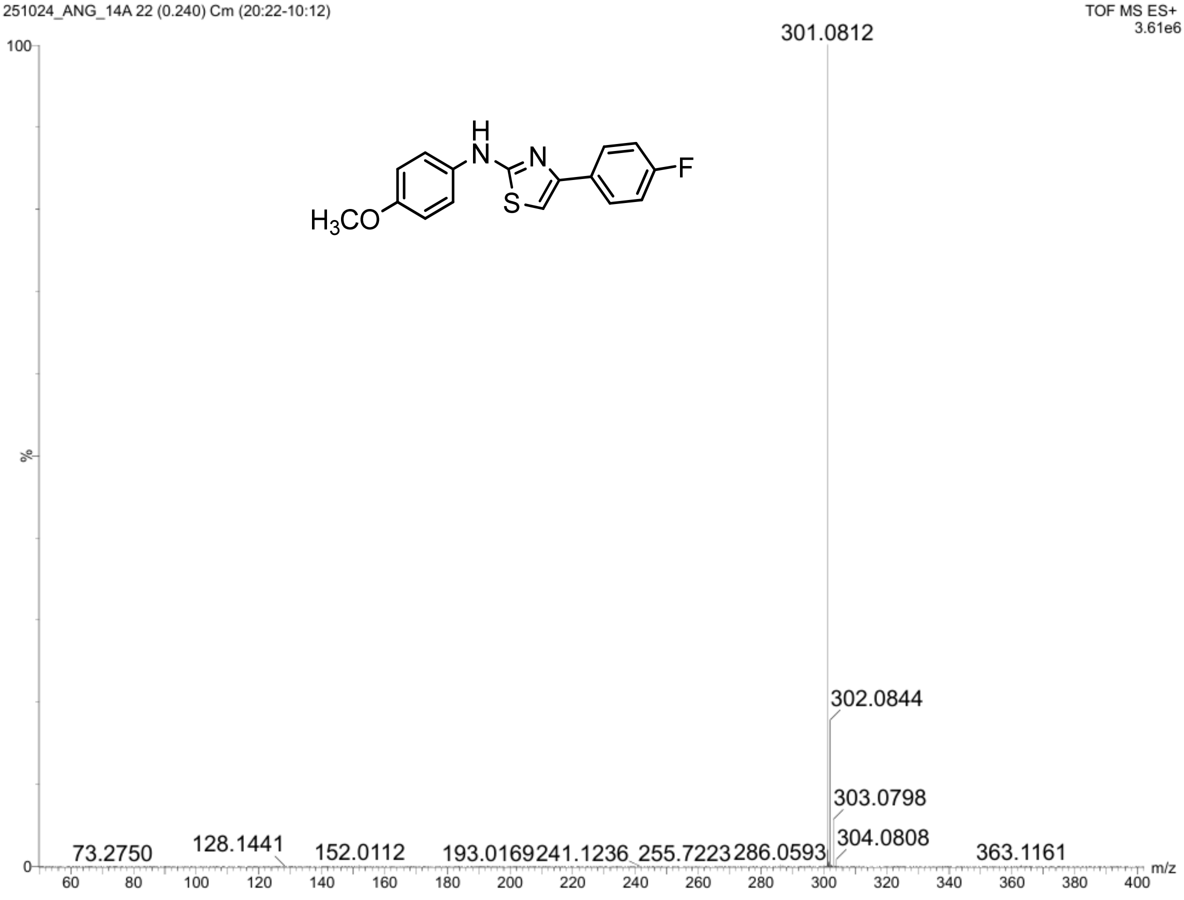

251024_ANG_14A 22 (0.240) Cm (20:22-10:12)
TOF MS ES+
3.61e6
301.0812
H
N
N
S
F
$H_3CO$
%
302.0844
303.0798
304.0808
73.2750
128.1441
152.0112
193.0169241.1236 255.7223 286.0593
363.1161
0
100
60 80 100 120 140 160 180 200 220 240 260 280 300 320 340 360 380 400 m/z

**Compound 3p**

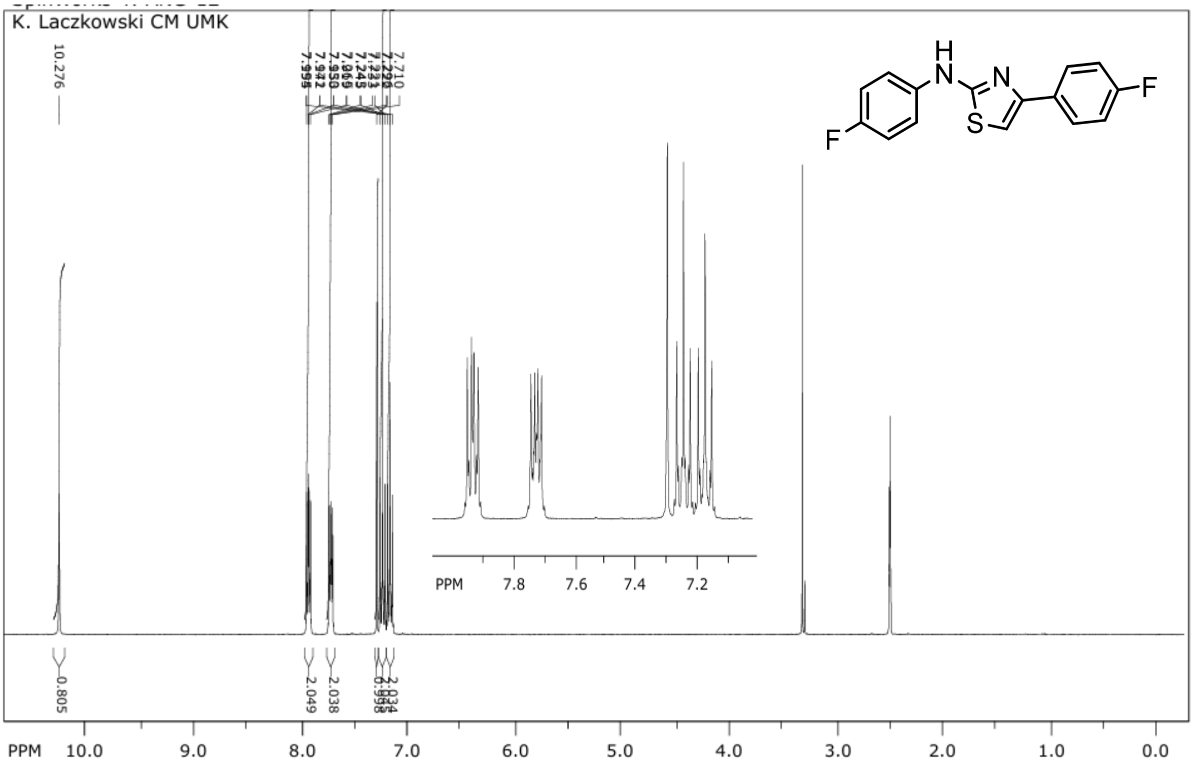
K. Laczkowski CM UMK
10.276
7.955
7.942
7.950
7.969
7.245
7.233
7.226
7.222
7.710
0.805
2.049
2.038
2.034
PPM 10.0 9.0 8.0 7.0 6.0 5.0 4.0 3.0 2.0 1.0 0.0
PPM 7.8 7.6 7.4 7.2
F
H
N
N
S
F

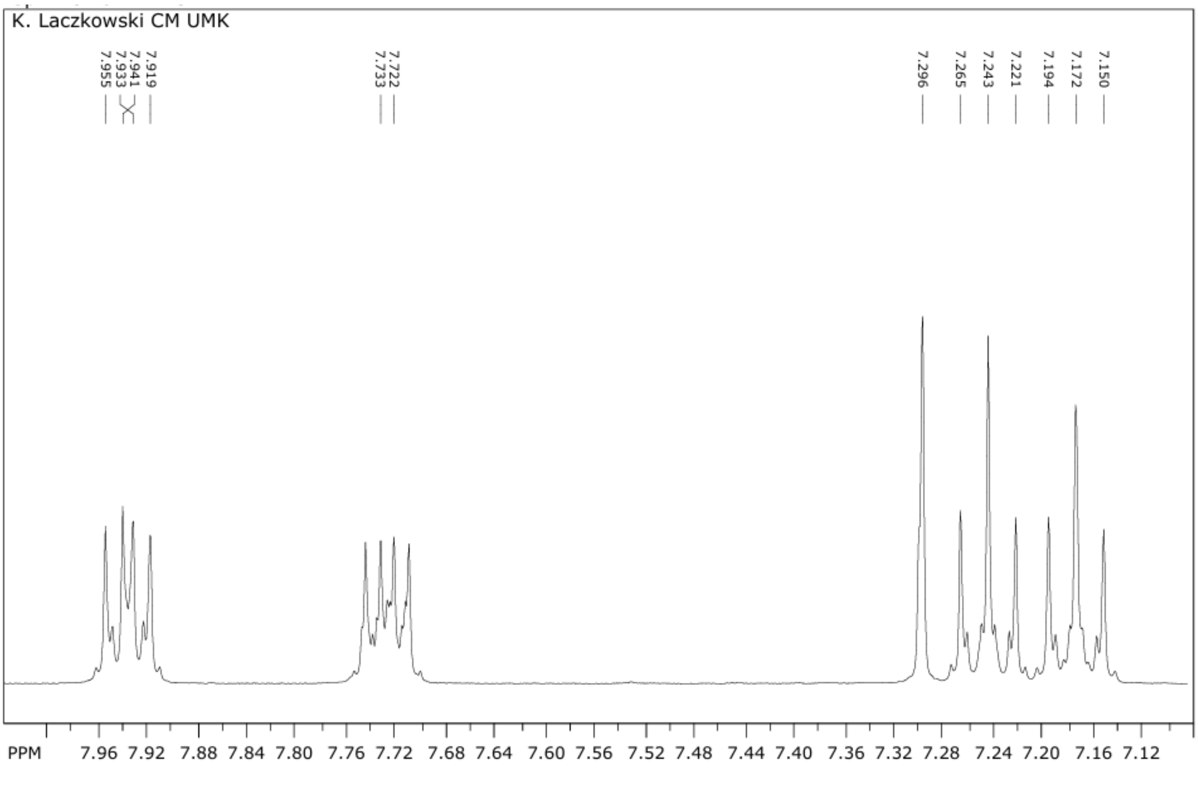
K. Laczkowski CM UMK
7.955
7.941
7.933
7.919
7.733
7.722
7.296
7.265
7.243
7.221
7.194
7.172
7.150
PPM 7.96 7.92 7.88 7.84 7.80 7.76 7.72 7.68 7.64 7.60 7.56 7.52 7.48 7.44 7.40 7.36 7.32 7.28 7.24 7.20 7.16 7.12

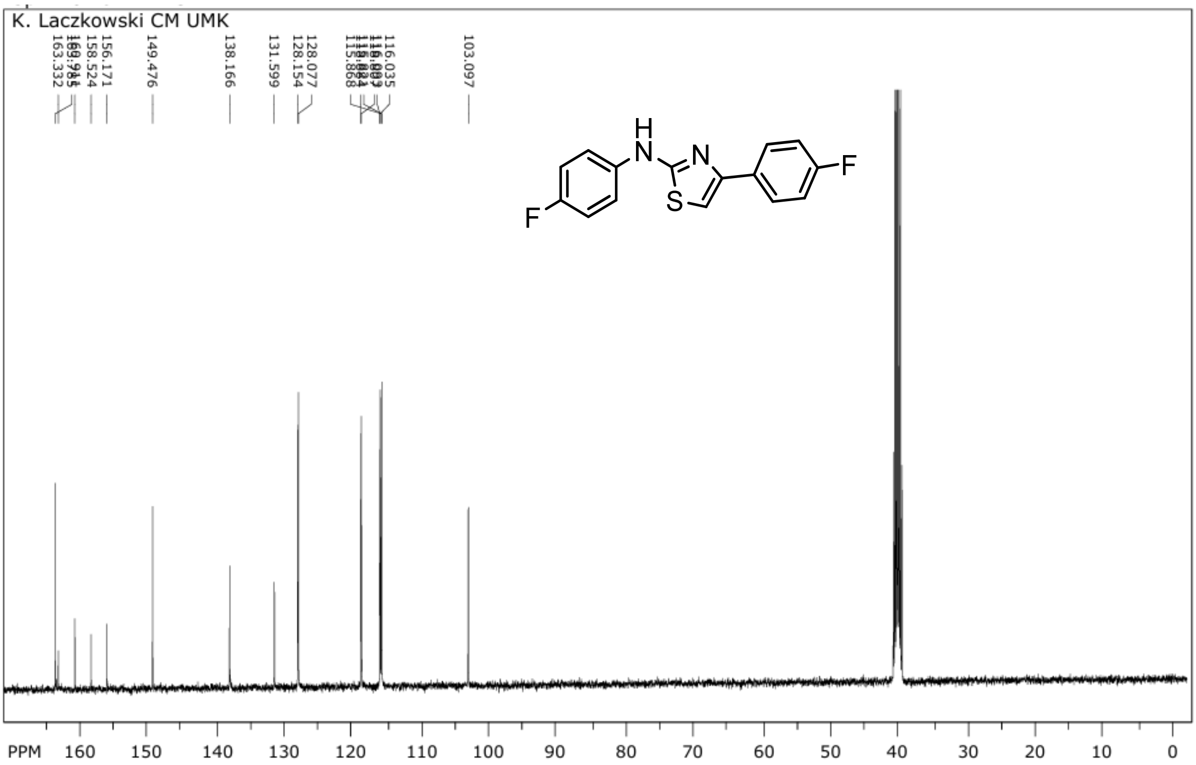
K. Laczkowski CM UMK
163.332
158.524
156.171
149.476
138.166
131.599
128.077
128.154
115.868
116.035
103.097
H
N
N
S
F
F
PPM 160 150 140 130 120 110 100 90 80 70 60 50 40 30 20 10 0

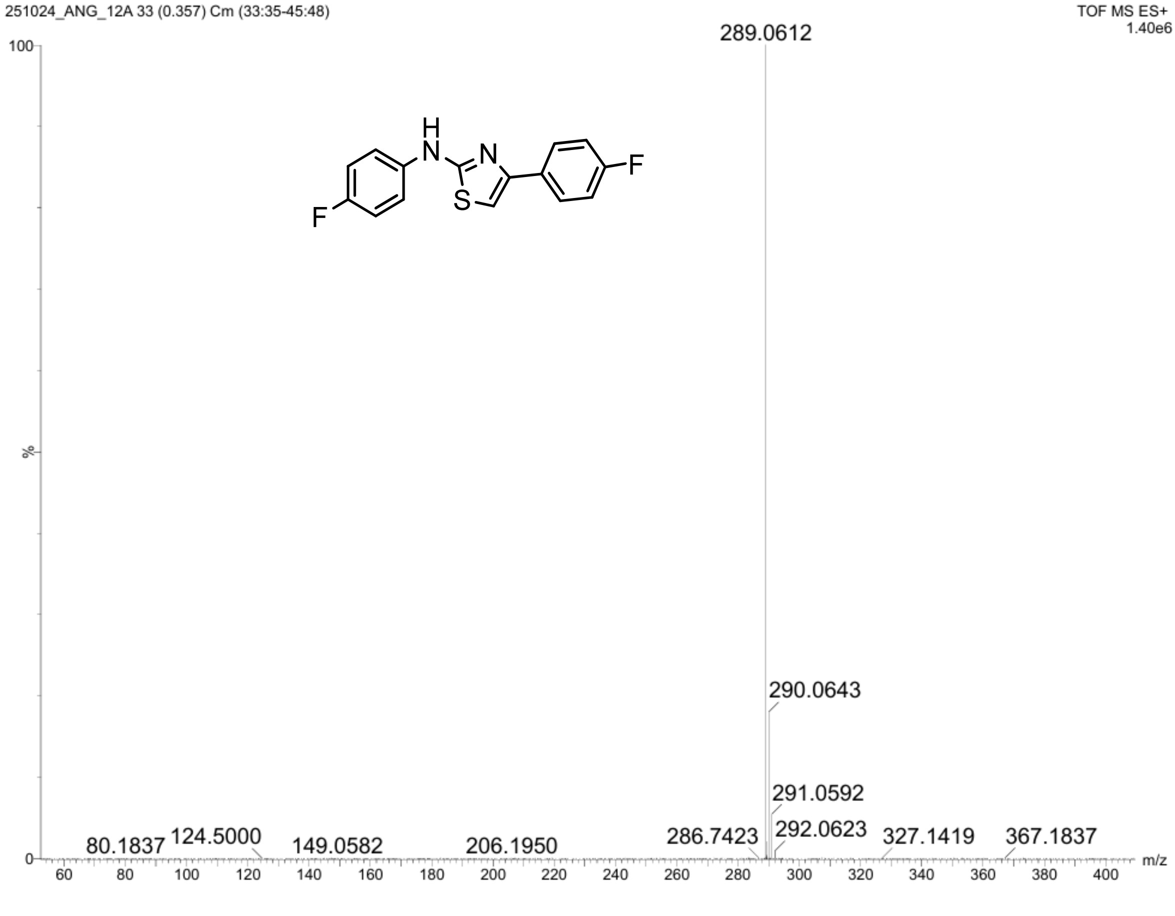
251024_ANG_12A 33 (0.357) Cm (33:35-45:48)
TOF MS ES+
1.40e6
289.0612
290.0643
291.0592
292.0623
80.1837
124.5000
149.0582
206.1950
286.7423
327.1419
367.1837
100
%
0
m/z
60 80 100 120 140 160 180 200 220 240 260 280 300 320 340 360 380 400
H
N
N
S
F
F

**Compound 3q**

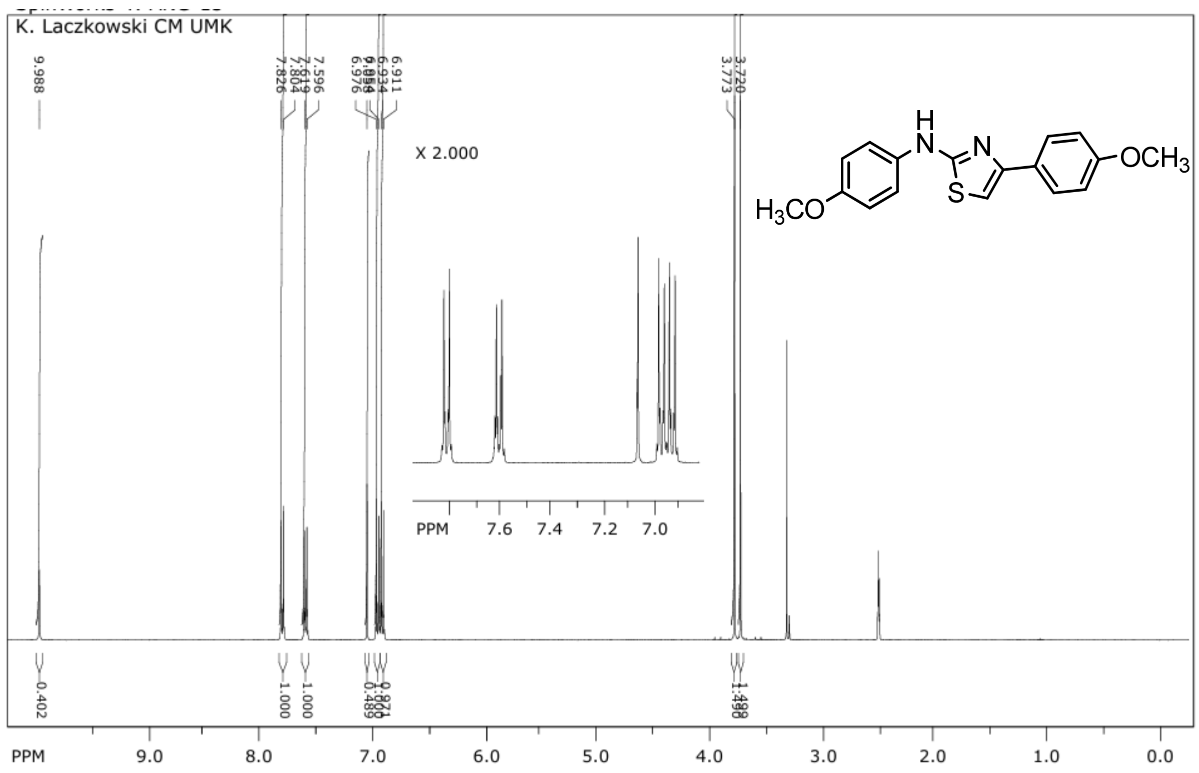
K. Laczkowski CM UMK
9.988
7.826
7.804
7.619
7.596
6.976
6.954
6.934
6.911
3.773
3.720
X 2.000
PPM 7.6 7.4 7.2 7.0
0.402
1.000
1.000
0.489
1.490
PPM 9.0 8.0 7.0 6.0 5.0 4.0 3.0 2.0 1.0 0.0
H
N
N
S
OCH3
H3CO

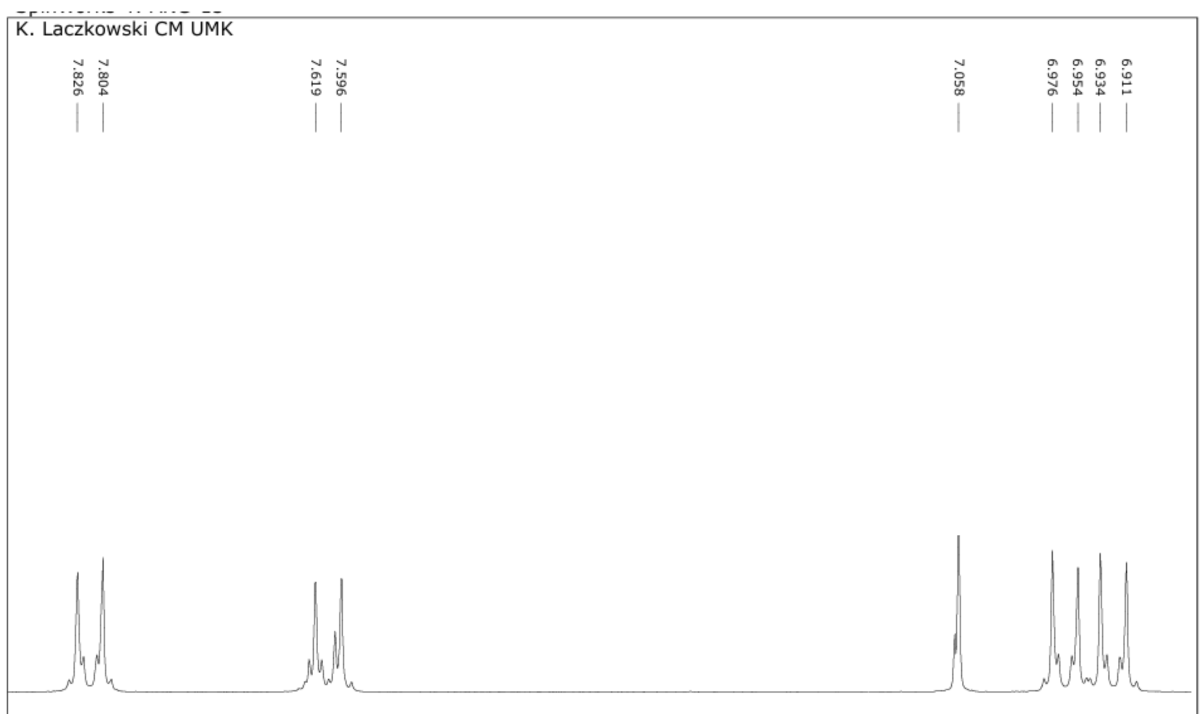
K. Laczkowski CM UMK
7.826
7.804
7.619
7.596
7.058
6.976
6.954
6.934
6.911
PPM 7.80 7.70 7.60 7.50 7.40 7.30 7.20 7.10 7.00 6.90

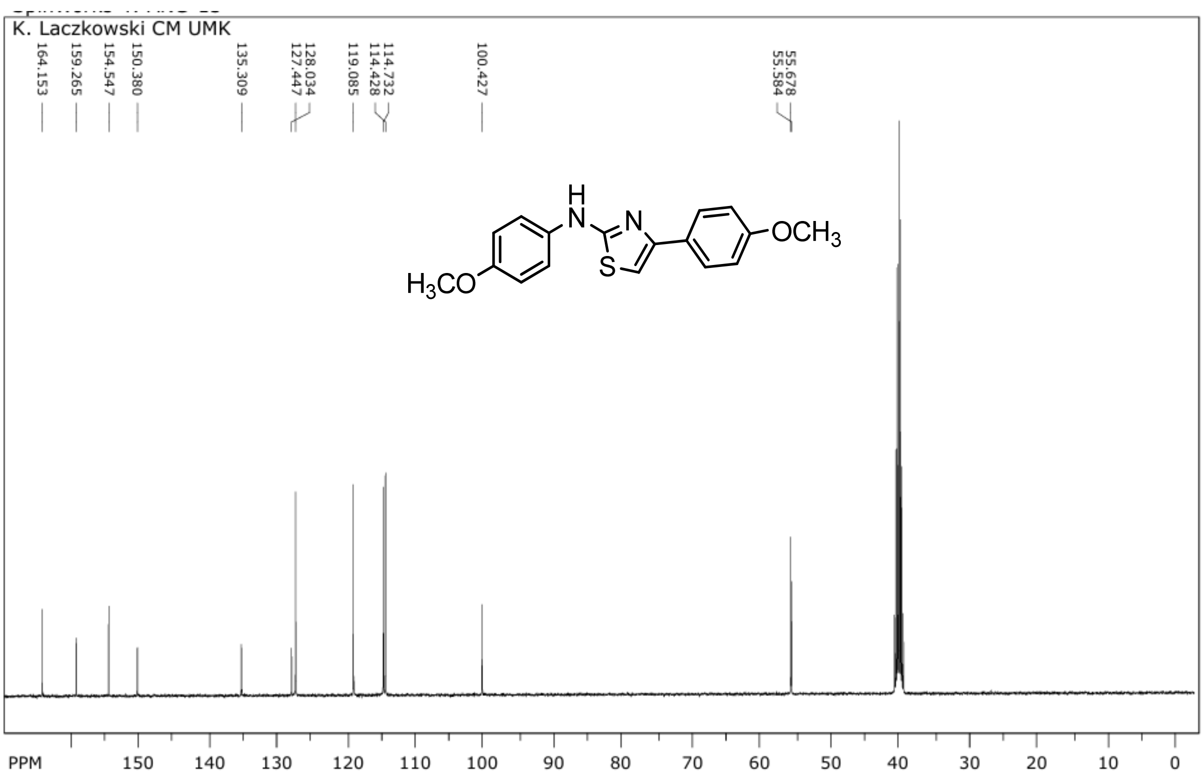
K. Laczkowski CM UMK
164.153
159.265
154.547
150.380
135.309
128.034
127.447
119.085
114.732
114.428
100.427
55.678
55.584
H
N
N
S
OCH3
H3CO
PPM
150
140
130
120
110
100
90
80
70
60
50
40
30
20
10
0

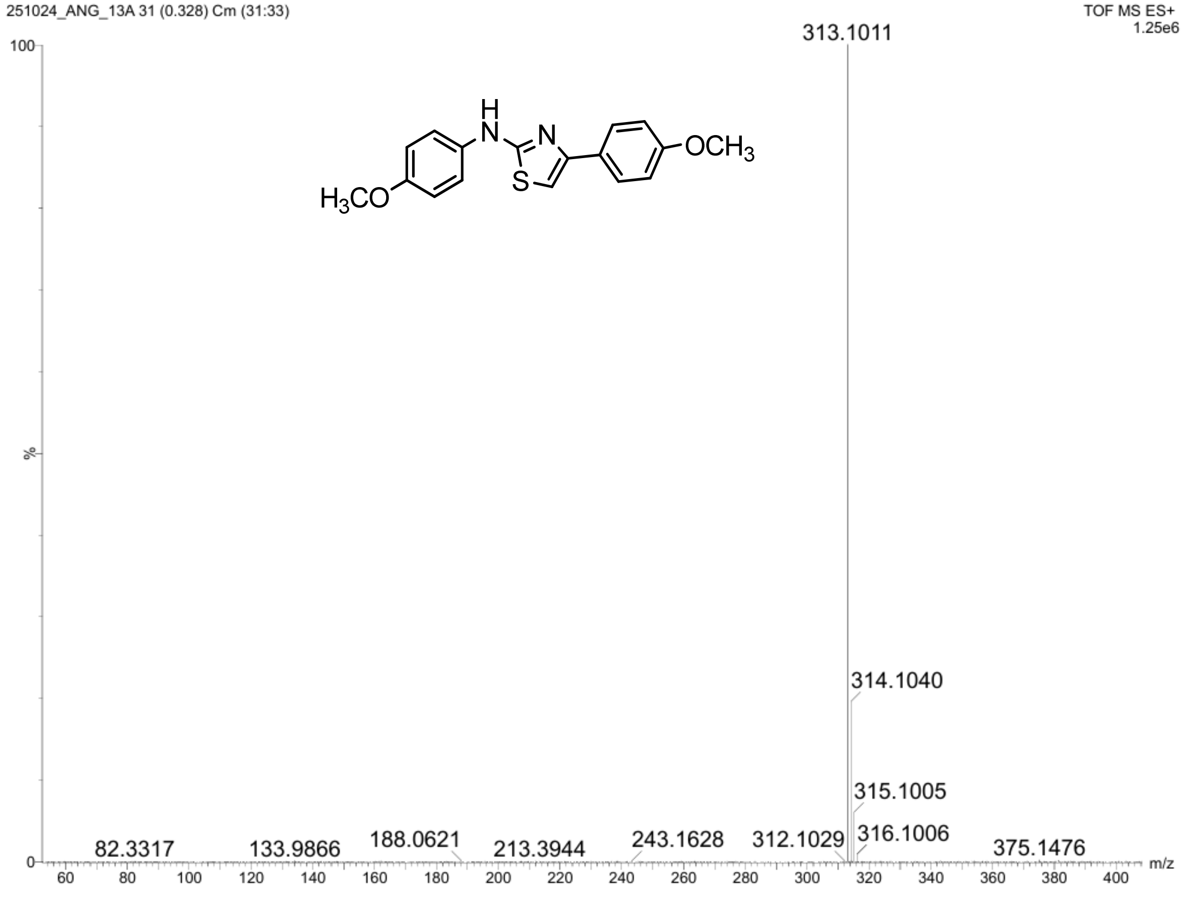
251024_ANG_13A 31 (0.328) Cm (31:33)
TOF MS ES+
1.25e6
313.1011
100
%
0
H
N
N
S
OCH3
H3CO
314.1040
315.1005
316.1006
82.3317
133.9866
188.0621
213.3944
243.1628
312.1029
375.1476
60
80
100
120
140
160
180
200
220
240
260
280
300
320
340
360
380
400
m/z

**Compound 3r**

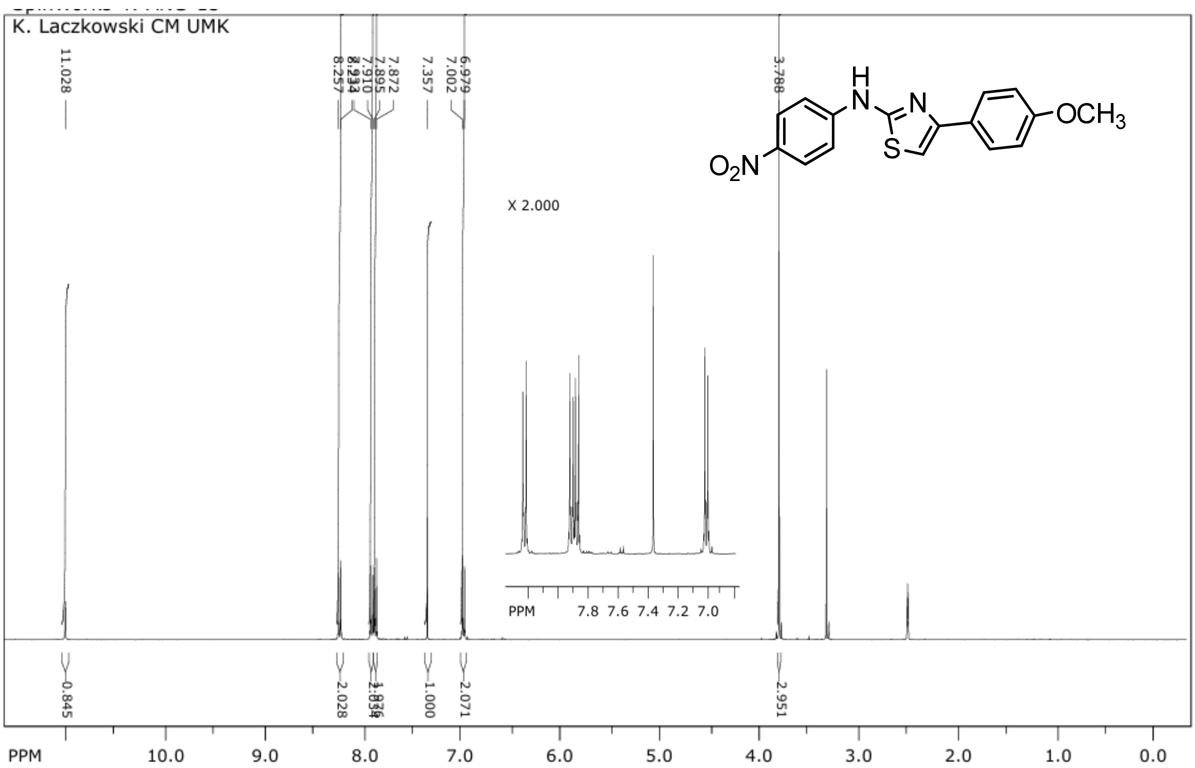
K. Laczkowski CM UMK
11.028
8.257
8.234
7.933
7.910
7.895
7.872
7.357
7.002
6.979
3.788
O2N
H
N
S
OCH3
X 2.000
PPM 7.8 7.6 7.4 7.2 7.0
0.845
2.028
1.000
2.071
2.951
PPM 10.0 9.0 8.0 7.0 6.0 5.0 4.0 3.0 2.0 1.0 0.0

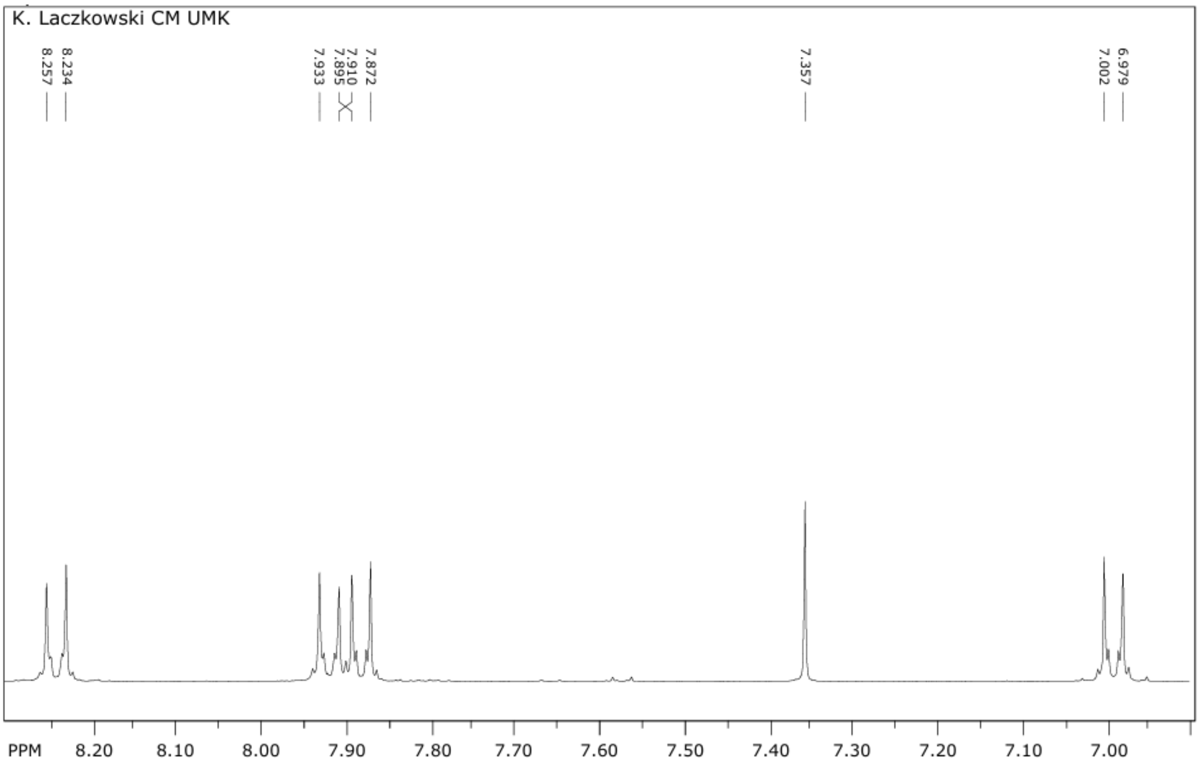
K. Laczkowski CM UMK
8.257
8.234
7.933
7.910
7.895
7.872
7.357
7.002
6.979
PPM 8.20 8.10 8.00 7.90 7.80 7.70 7.60 7.50 7.40 7.30 7.20 7.10 7.00

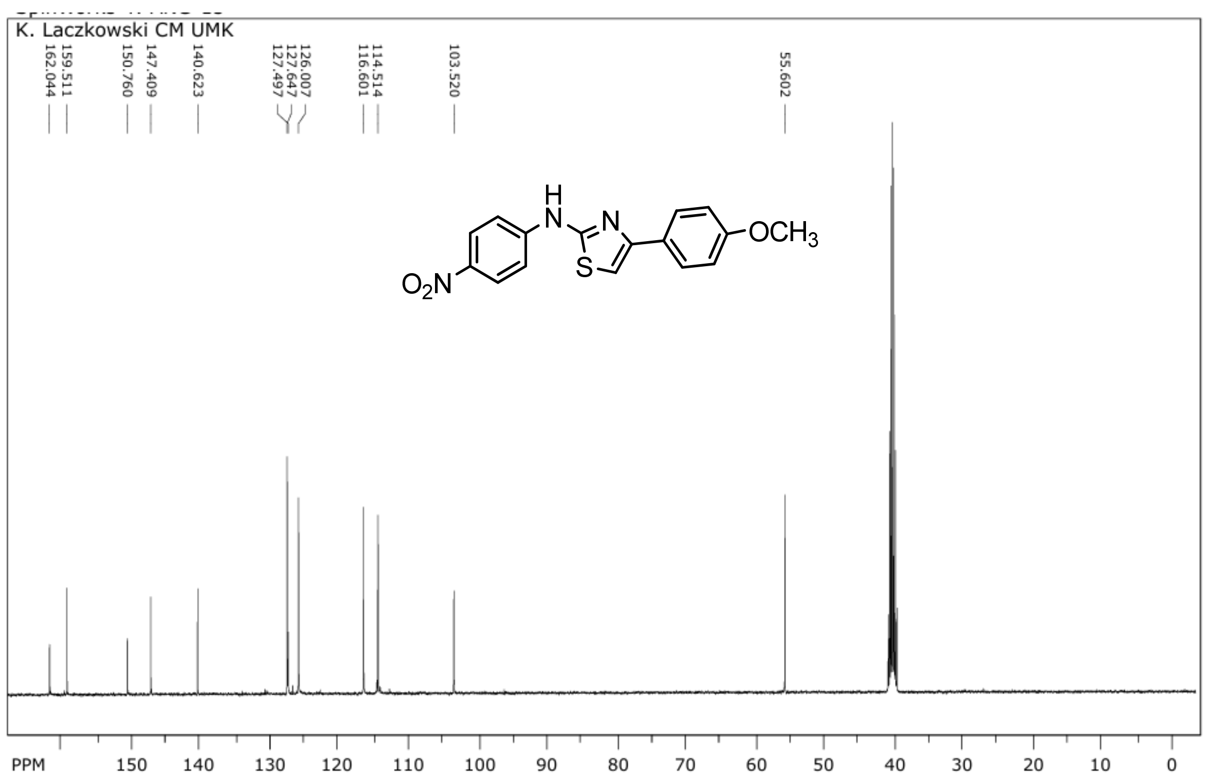
K. Laczkowski CM UMK
162.044
159.511
150.760
147.409
140.623
127.647
127.497
126.007
116.601
114.514
103.520
55.602
PPM
OCH3
O2N
H
N
S

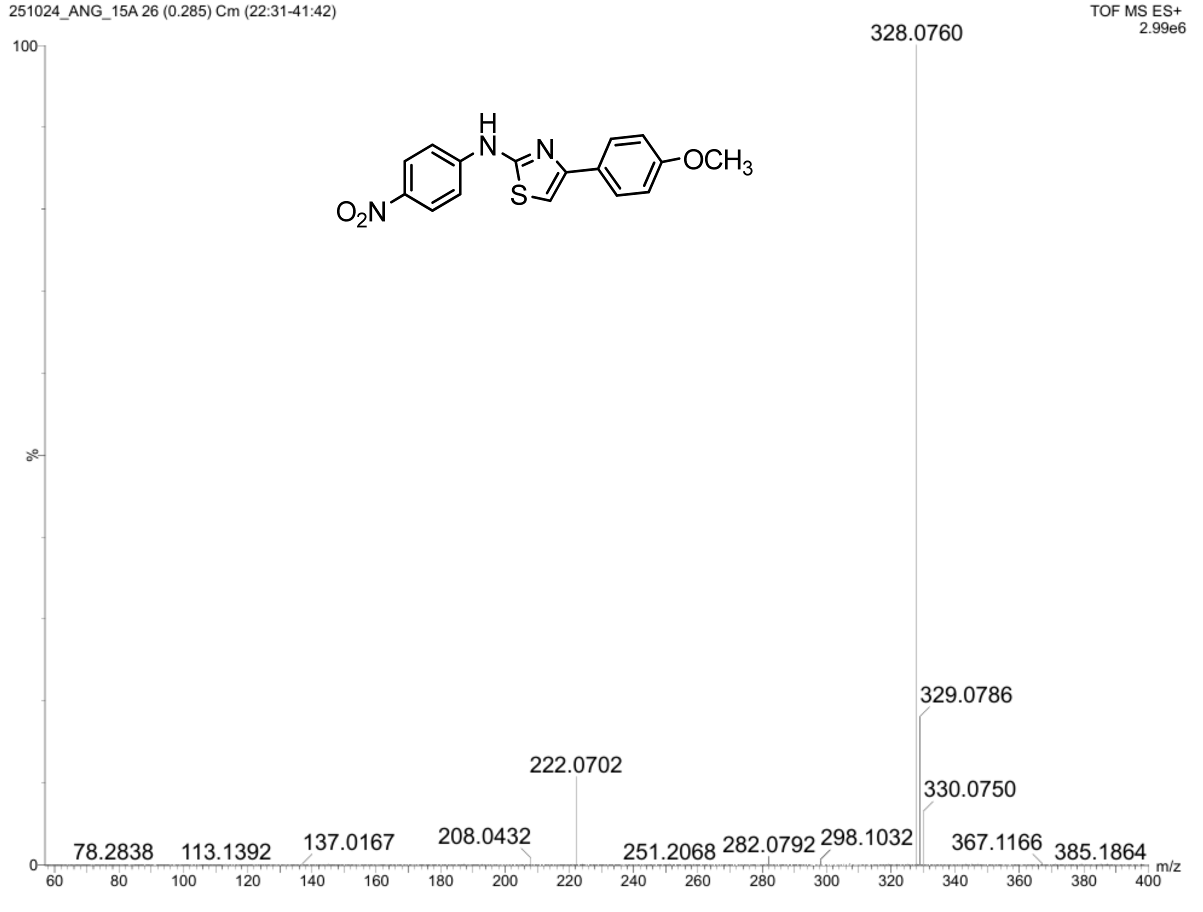
251024_ANG_15A 26 (0.285) Cm (22:31-41:42)
TOF MS ES+
2.99e6
328.0760
329.0786
330.0750
222.0702
78.2838
113.1392
137.0167
208.0432
251.2068
282.0792
298.1032
367.1166
385.1864
m/z
%
OCH3
O2N

**Compound 3s**

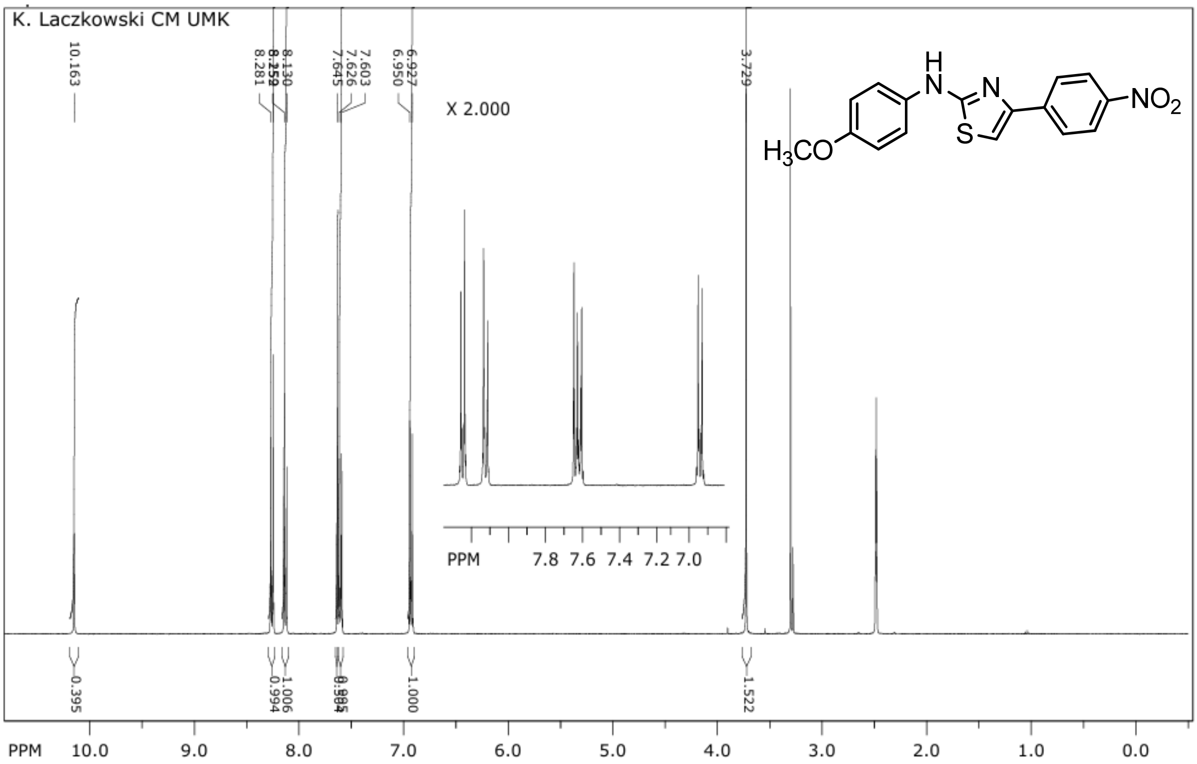
K. Laczkowski CM UMK
10.163
8.281
8.259
8.130
7.645
7.626
7.603
6.950
6.927
3.729
X 2.000
PPM 7.8 7.6 7.4 7.2 7.0
$H_3CO$
H
N
N
S
$NO_2$
0.395
0.994
1.006
1.000
1.522
PPM 10.0 9.0 8.0 7.0 6.0 5.0 4.0 3.0 2.0 1.0 0.0

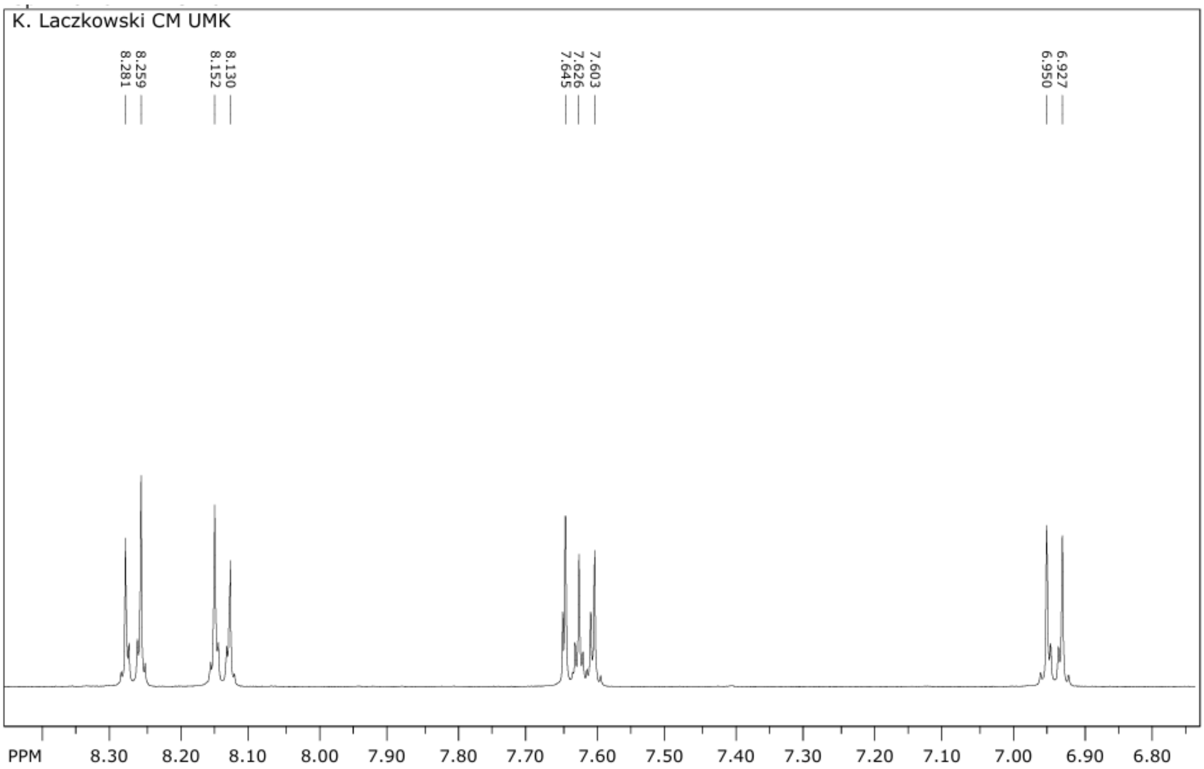
K. Laczkowski CM UMK
8.281
8.259
8.152
8.130
7.645
7.626
7.603
6.950
6.927
PPM 8.30 8.20 8.10 8.00 7.90 7.80 7.70 7.60 7.50 7.40 7.30 7.20 7.10 7.00 6.90 6.80

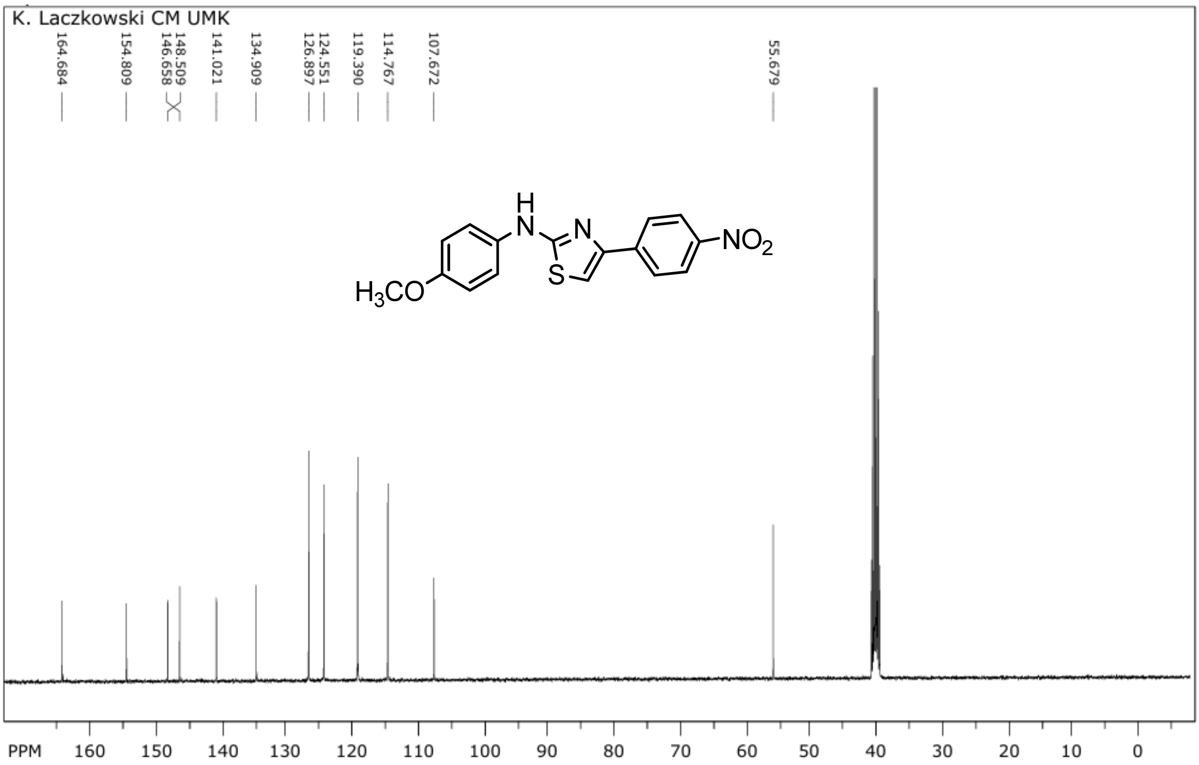
K. Laczkowski CM UMK
164.684
154.809
148.509
146.658
141.021
134.909
126.897
124.551
119.390
114.767
107.672
55.679
H
N
N
S
NO2
H3CO
PPM
160
150
140
130
120
110
100
90
80
70
60
50
40
30
20
10
0

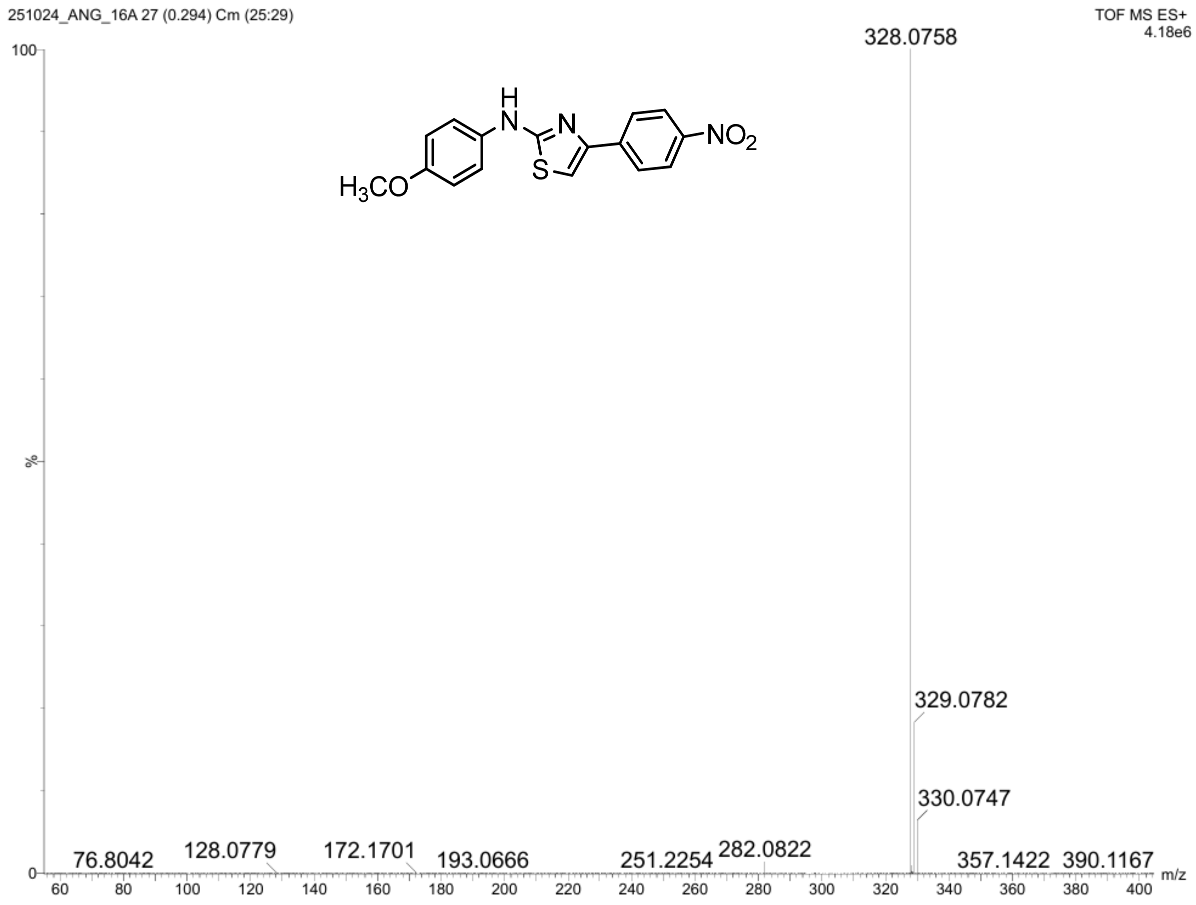
251024_ANG_16A 27 (0.294) Cm (25:29)
TOF MS ES+
4.18e6
328.0758
100
%
0
H
N
N
S
NO2
H3CO
329.0782
330.0747
76.8042
128.0779
172.1701
193.0666
251.2254
282.0822
357.1422
390.1167
m/z
60
80
100
120
140
160
180
200
220
240
260
280
300
320
340
360
380
400

**Compound 3t**

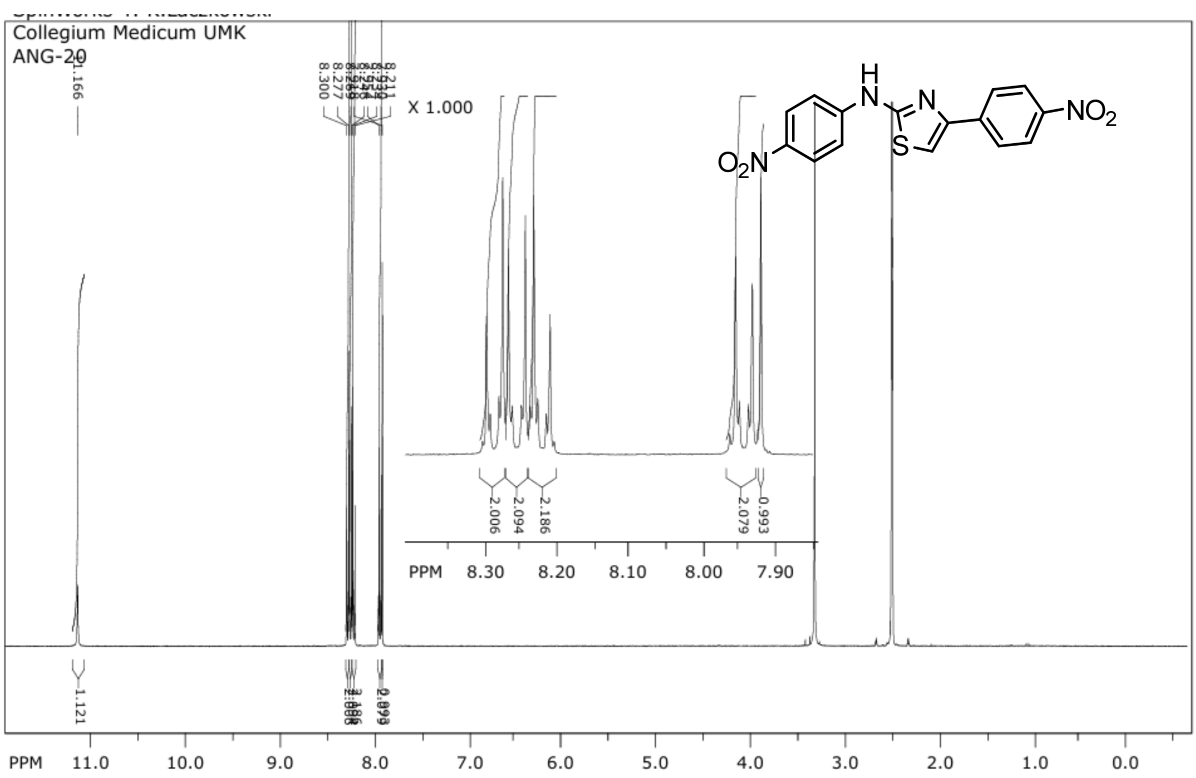

Collegium Medicum UMK
ANG-20
11.166
8.300
8.277
8.211
X 1.000
2.006
2.094
2.186
2.079
0.993
PPM 8.30 8.20 8.10 8.00 7.90
1.121
PPM 11.0 10.0 9.0 8.0 7.0 6.0 5.0 4.0 3.0 2.0 1.0 0.0
H
N
S
$NO_2$
$O_2N$


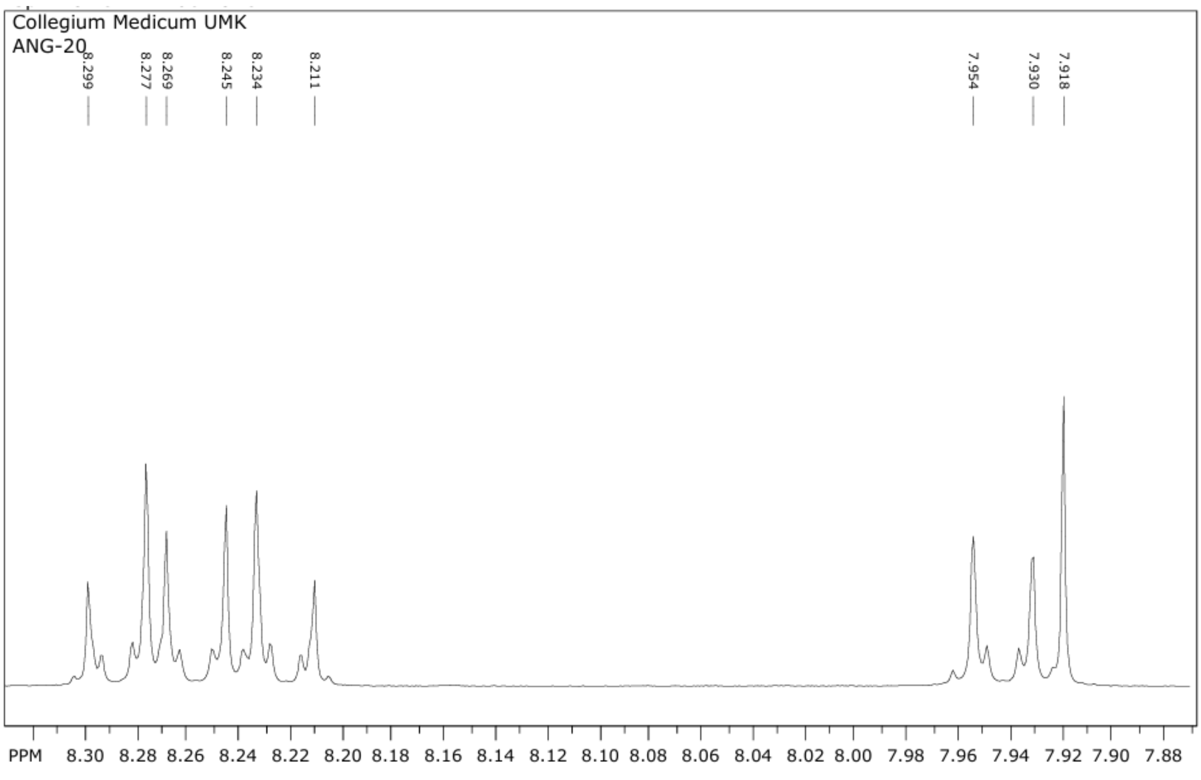

Collegium Medicum UMK
ANG-20
8.299
8.277
8.269
8.245
8.234
8.211
7.954
7.930
7.918
PPM 8.30 8.28 8.26 8.24 8.22 8.20 8.18 8.16 8.14 8.12 8.10 8.08 8.06 8.04 8.02 8.00 7.98 7.96 7.94 7.92 7.90 7.88

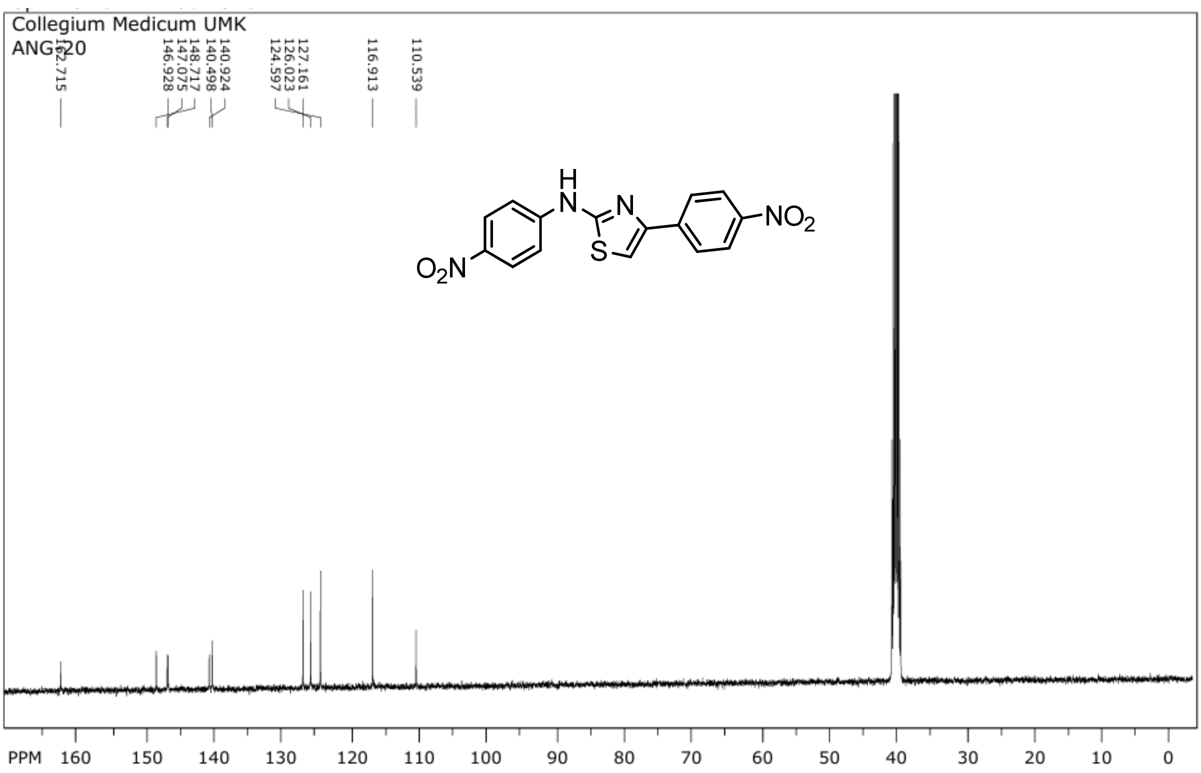

Collegium Medicum UMK
ANG120
162.715
148.717
147.075
146.928
140.924
140.498
127.161
126.023
124.597
116.913
110.539
H
N
N
S
NO2
O2N
PPM 160 150 140 130 120 110 100 90 80 70 60 50 40 30 20 10 0

**Figure S2.** Correlation between calculated molecular properties and the experimental chemical shifts $\delta$ of thiazole-5H proton in compounds **3a**-**3m**. Symbol $\Delta E$ denotes HOMO–LUMO energy gap, $IP$—ionization potential, $EA$—electron affinity, $\chi$—electronegativity, and $\eta$—chemical hardness.

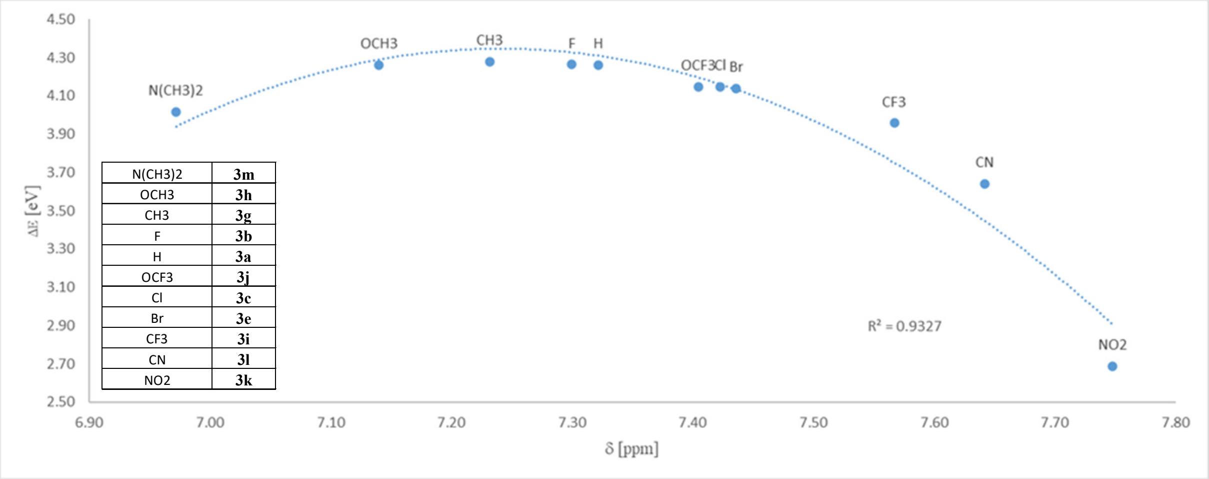


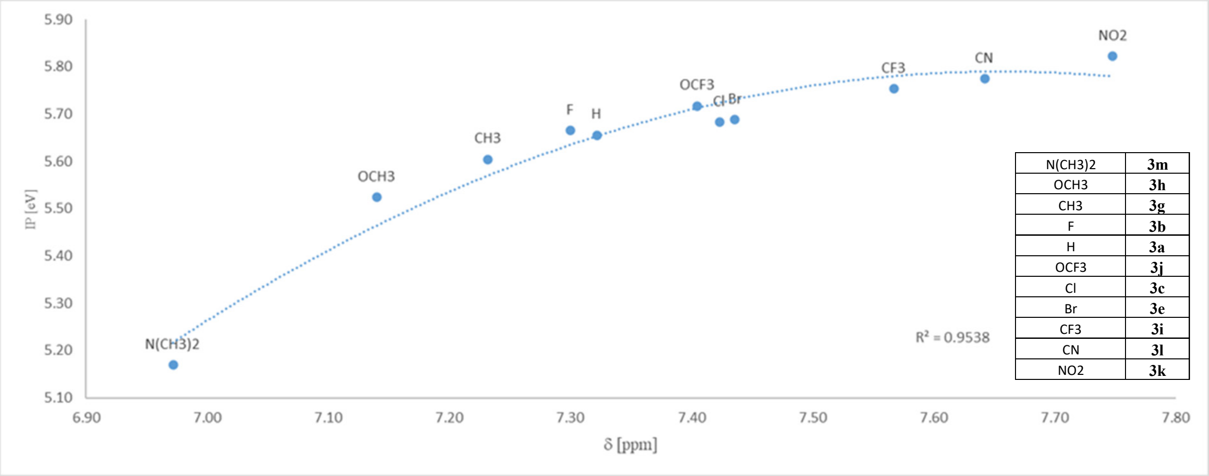


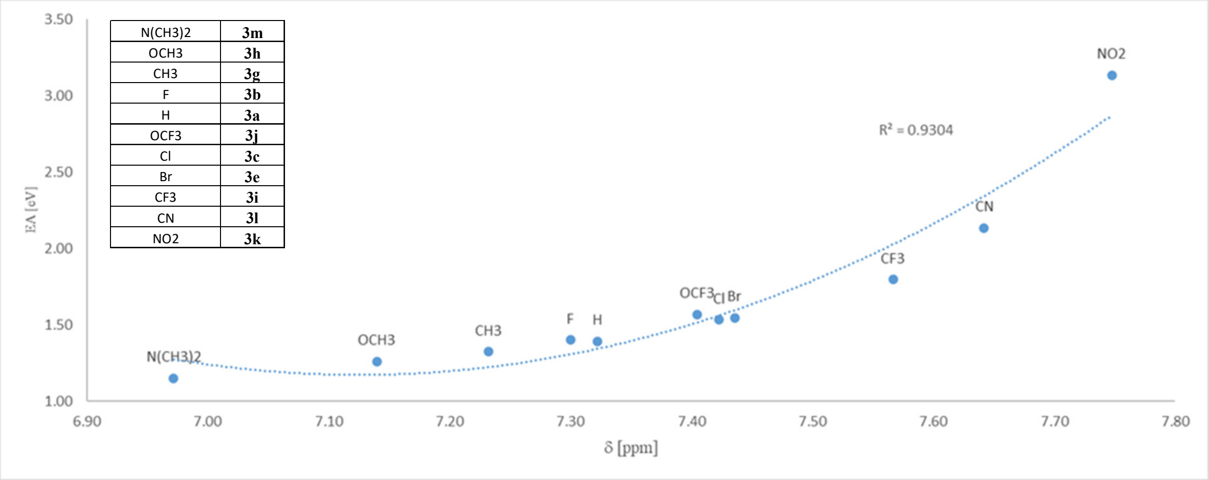

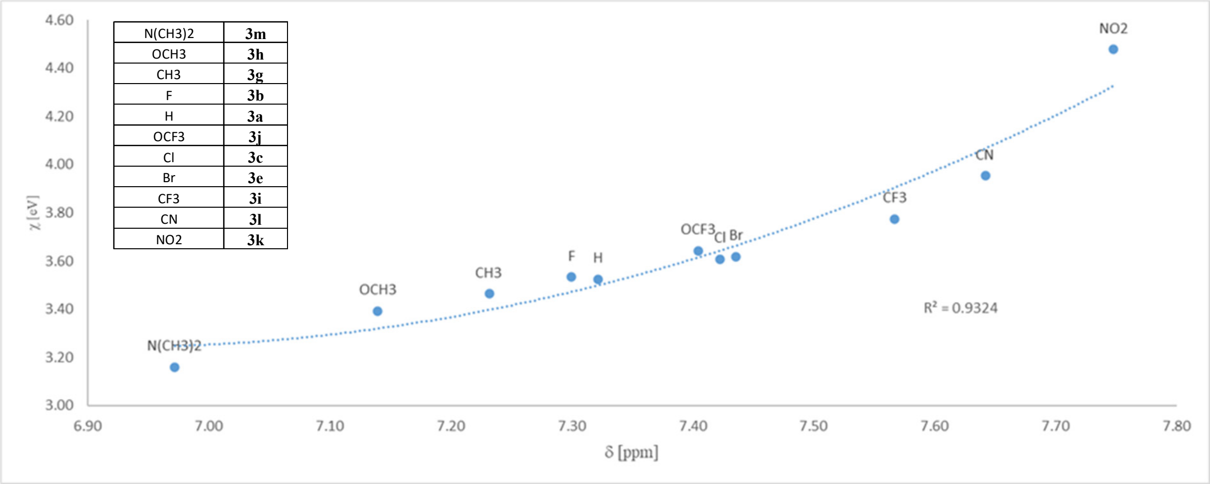

N(CH3)2 3m
OCH3 3h
CH3 3g
F 3b
H 3a
OCF3 3j
Cl 3c
Br 3e
CF3 3i
CN 3l
NO2 3k
NO2
CN
CF3
OCF3
Cl
Br
F
H
CH3
OCH3
N(CH3)2
R² = 0.9324
χ [eV]
δ [ppm]
4.60
4.40
4.20
4.00
3.80
3.60
3.40
3.20
3.00
6.90
7.00
7.10
7.20
7.30
7.40
7.50
7.60
7.70
7.80


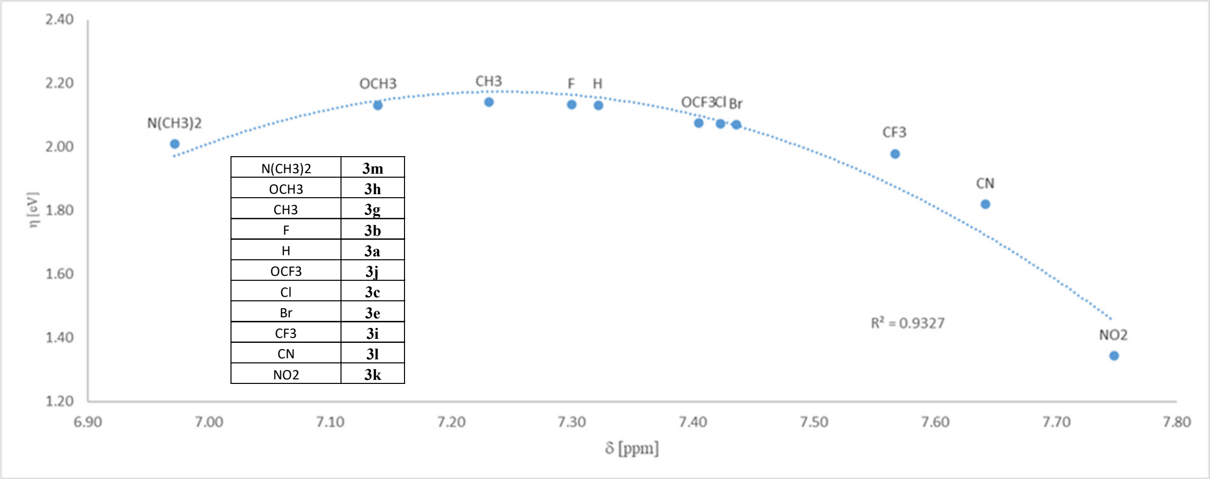

N(CH3)2 3m
OCH3 3h
CH3 3g
F 3b
H 3a
OCF3 3j
Cl 3c
Br 3e
CF3 3i
CN 3l
NO2 3k
N(CH3)2
OCH3
CH3
F
H
OCF3
Cl
Br
CF3
CN
NO2
R² = 0.9327
η [eV]
δ [ppm]
2.40
2.20
2.00
1.80
1.60
1.40
1.20
6.90
7.00
7.10
7.20
7.30
7.40
7.50
7.60
7.70
7.80
